# Nitrogen Insertion via Asymmetric Condensation and Chirality Transfer: A Stereodivergent Entry to Cyanocyclopropanes

**DOI:** 10.1002/anie.202503056

**Published:** 2025-04-14

**Authors:** Marlene Arnold, Jasmin Hammes, Mike Ong, Christian Mück‐Lichtenfeld, Johannes M. Wahl

**Affiliations:** ^1^ Department Chemie Johannes Gutenberg‐Universität Duesbergweg 10–14 55128 Mainz Germany; ^2^ Organisch‐Chemisches Institut Universität Münster Corrensstraße 36 48149 Münster Germany

**Keywords:** Asymmetric catalysis, Chirality transfer, Desymmetrization, Nitrogen insertion, Ring contraction

## Abstract

The condensation of prochiral cyclobutanones and diphenylphosphinyl hydroxylamine is achieved under Brønsted acid catalysis. Interestingly, the competing aza‐Baeyer–Villiger reaction is completely suppressed and the axially chiral oxime esters can be isolated in excellent yield and selectivity (up to 96% yield, up to 97:3 er). Computational analysis highlights the crucial role of the Brønsted acid in facilitating a successful condensation. Building on the inherent reactivity of the corresponding oxime esters, a one‐pot protocol toward cyanocyclopropanes was discovered, which establishes two consecutive stereocenters. This unusual ring contraction is triggered by strong base and permits an axial‐to‐point chirality transfer with good enantiospecificity (up to 98% es). Fine‐tuning the reaction parameters enables stereodivergent access to both diastereomers of the cyanocyclopropanes, and the utility of this method is demonstrated through the formal synthesis of the drug tasimelteon.

## Graphical abstract

The synthesis of cyanocyclopropanes is achieved via asymmetric nitrogen insertion into prochiral cyclobutanones. Key mechanistic steps involve an organocatalyzed asymmetric condensation followed by chirality transfer via an intercepted Neber rearrangement. The reaction can be conducted in a one‐pot, stereodivergent manner, allowing access to all possible cyclopropane isomers with precise control of stereochemistry.

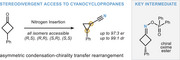

## Introduction

**Scheme 1:**
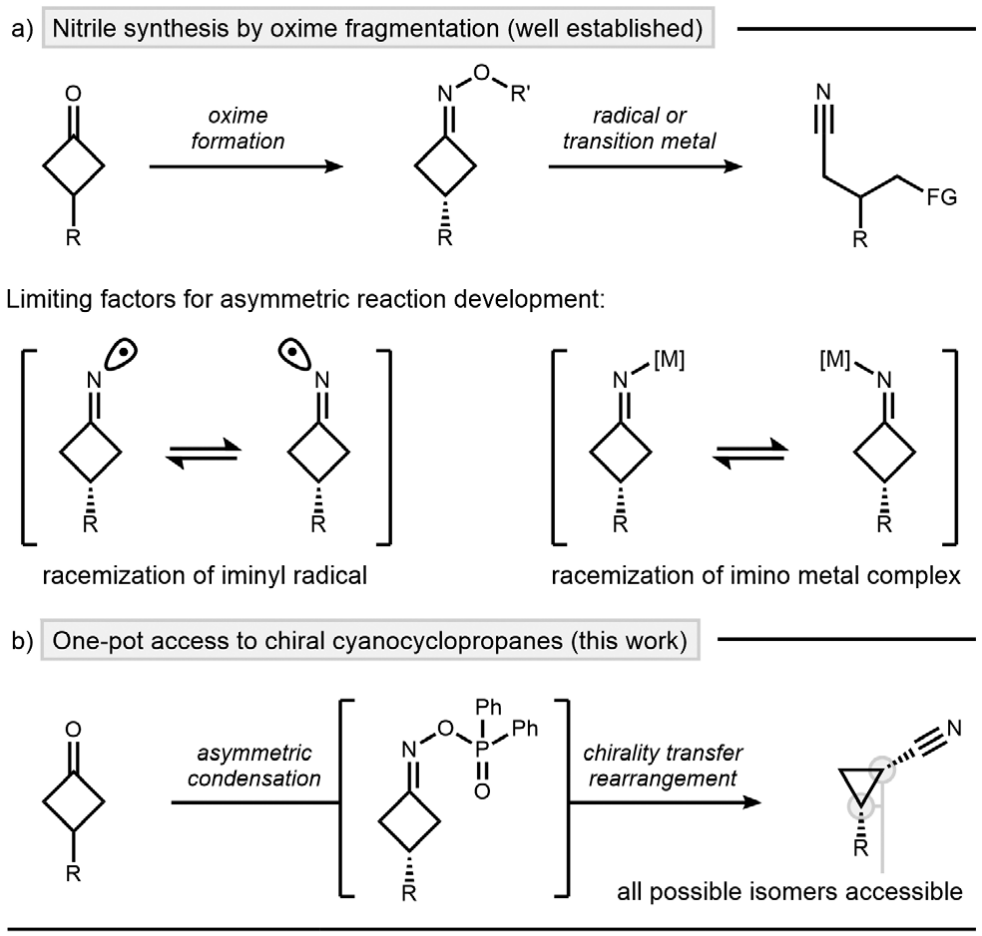
Nitrile synthesis via fragmentation. a) Racemic strategies and their limitations. b) Asymmetric version using enantioselective condensation and chirality transfer as key design elements. FG, functional group.

Oxime esters represent versatile intermediates in synthesis.^[^ ^1^ ^]^ Among their transformations, methods that convert oxime esters to nitriles have attracted considerable attention, owing to the important role nitrile functionality plays in natural products^[^ ^2^ ^]^ and pharmaceutical compounds.^[^ ^3^ ^]^ In particular, C─C bond cleavage of cyclobutanone‐derived oxime esters has enabled new pathways to access valuable nitrile‐containing building blocks (Scheme 1a, top).^[^ ^4^ ^]^ In contrast to classical Beckmann fragmentation strategies,^[^ ^5^, ^6^, ^7^, ^8^, ^9^, ^10^ ^]^ current endeavors hinge on the susceptibility of oxime esters to undergo single electron transfer (SET) and oxidative addition (OA) processes.^[^ ^11^, ^12^, ^13^, ^14^, ^15^, ^16^, ^17^ ^]^ However, the key intermediates in such transformations—namely, the iminyl radical^[^ ^18^, ^19^ ^]^ or imino‐metal complex^[^ ^20^ ^]^—rapidly lose their inherent stereointegrity, significantly hindering the development of asymmetric protocols (Scheme 1a, bottom). As a consequence, catalytic methods that establish and transfer chirality from oxime C═N double bonds remain largely unexplored and only very recently first studies were disclosed. In 2017, Antilla and coworkers showed that chiral phosphoric acids (CPAs) can act as catalysts to access optically active oximes via condensation.^[^ ^21^, ^22^, ^23^ ^]^ Although effective, this method did not disclose any chirality‐transfer reactions, possibly due to the relatively unreactive nature of the targeted oxime ethers. Recently, the groups of Tan^[^ ^24^ ^]^ and Shi^[^ ^25^ ^]^ succeeded in transferring oxime chirality by the development of a stereoretentive deprotection–activation protocol for chiral oxime ethers, enabling stereoselective Beckmann rearrangements. However, the necessity of this multistep sequence remains a limitation. Palladium catalysis has also shown potential for stereospecific transformations of oxime esters through ligand adjustments, yet its application to chiral precursors remains unknown.^[^ ^26^, ^27^ ^]^

In this work, we address previous limitations by first enabling enantioselective synthesis of axially chiral cyclobutanone oxime esters and subsequently exploring their transformations via chirality transfer (Scheme 1b). Our approach leverages a site‐selective deprotonation strategy leading to cyanocyclopropanes via ring‐contraction, avoiding selectivity issues common to iminyl radical and transition metal chemistry. Mechanistic studies shine light on the synergy between the stereoselective condensation and stereospecific ring contraction, ultimately leading to stereodivergent access to chiral *cis* and *trans* configured cyclopropanes using commercially available diphenylphosphinyl hydroxylamine (DPPH) as a nitrogen source.^[^ ^28^, ^29^, ^30^, ^31^, ^32^, ^33^ ^]^

## Results and Discussion

**Scheme 2:**
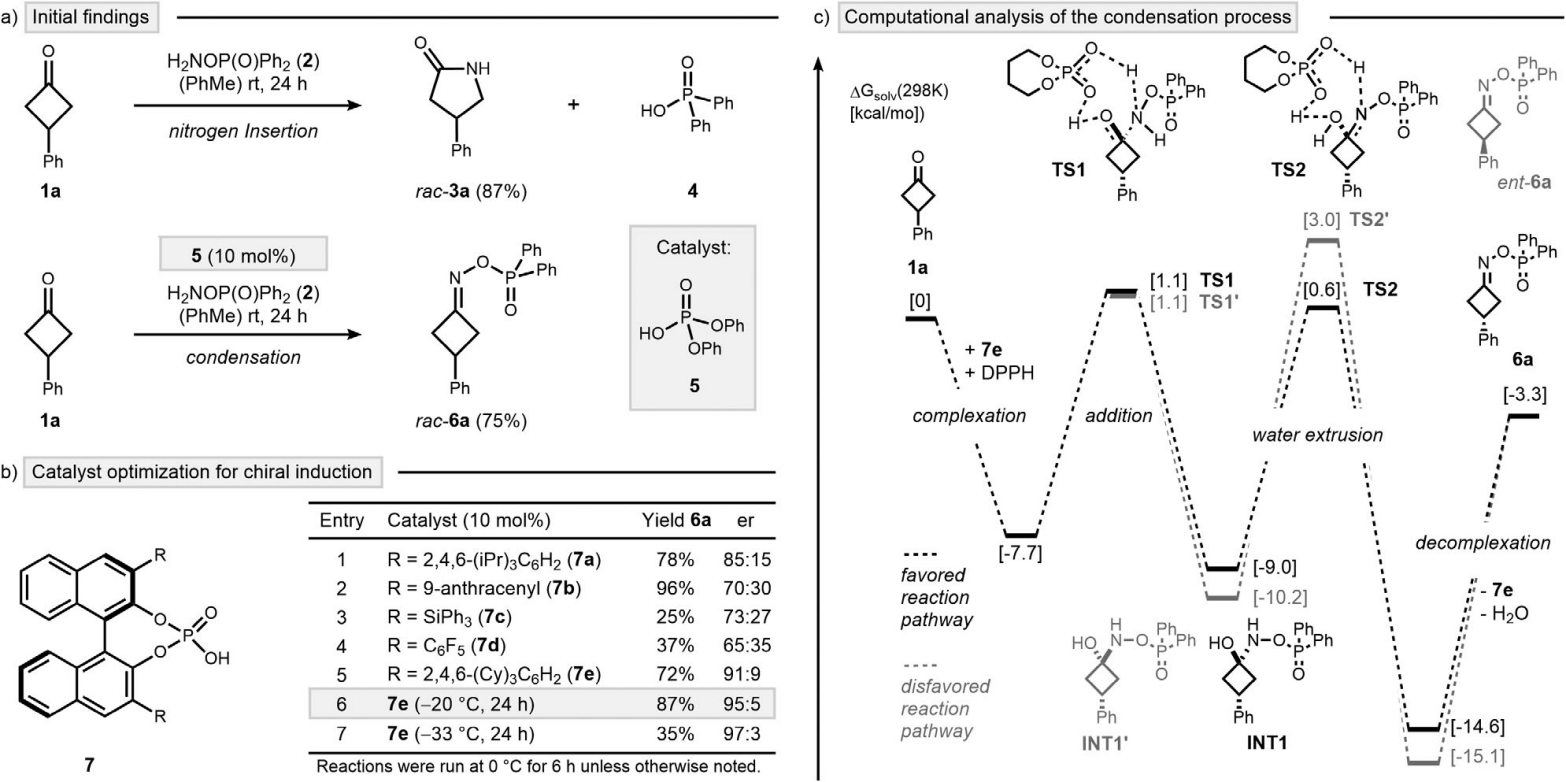
Access to cyclobutanone‐derived oxime esters by condensation. a) Initial findings on the effect of phosphoric acid **5** as catalyst. b) Establishing enantioselectivity through CPA catalyst optimization. Yields were determined by NMR using dibromomethane as an internal standard. Enantiomeric ratios were determined by HPLC using a chiral column. c) Computational assessment of the condensation reaction. Relative free energies (kcal mol^−1^) were determined in toluene and are shown in brackets. For the sake of clarity, CPA (**7e**) is not presented in its entirety.

This study originated from a surprising observation during our recent optimization of the aza‐Baeyer–Villiger nitrogen insertion at cyclobutanone **1a** with DPPH (**2**) (Scheme 2a, top).^[^ ^34^ ^]^ Upon introducing a catalytic amount of diphenylphosphoric acid (**5**), the reaction's outcome shifted markedly, yielding the condensation product *rac*‐**6a** instead of the expected nitrogen insertion product *rac*‐**3a** (Scheme 2a, bottom). This finding was particularly intriguing as diphenylphosphinic acid (**4**), a stoichiometric byproduct of the insertion reaction, failed to catalyze the condensation effectively.^[^ ^35^ ^]^ We hypothesized that oxime esters, such as *rac*‐**6a**, could serve as valuable intermediates for discovering novel nitrogen insertion reactions due to their relatively weak N─O bond and axial chirality. To explore this, we first looked for an asymmetric synthetic route to **6a** with an aim to subsequently enable chirality transfer reactions. As an initial attempt, 10 mol% of CPA **7a** was employed instead of phosphoric acid **5** to probe asymmetric induction at 0 °C (Scheme 2b, Entry 1). Gratifyingly, oxime ester **6a** was isolated in good yield, with a notable enantiomeric ratio (er) of 85:15. Subsequent modification of the CPA scaffold (Entries 2–5) revealed CPA **7e** as the optimal catalyst, achieving a superior enantioselectivity of 91:9 er. Further refinement demonstrated that performing the reaction at −20 °C enhanced the selectivity to 95:5 er (Entry 6). Lowering the reaction temperature further improved the enantiomeric ratio to 97:3; however, incomplete conversions were observed under these conditions (Entry 7). Further modifications involving the hydroxylamine reagent, reaction additives, and applied solvent did not result in additional improvements (see Supporting Information for a comprehensive optimization study).

To gain deeper insights into the critical interactions leading to high induction during the condensation process, we performed computational studies using density functional theory (DFT) (Scheme 2c).^[^ ^36^ ^]^ Our findings reveal that CPA **7e** not only catalyzes the extrusion of water but also facilitates the addition of DPPH (**2**) to the ketone moiety of cyclobutanone **1a** via transition state (TS) **TS1**. However, the addition process was found to be unspecific leading to the reversible formation of both hemiaminal intermediates **Int1** and **Int1’**. The stereoselectivity‐determining condensation preferentially proceeds from hemiaminal **Int1** via **TS2** to afford oxime ester **6a** (shown in black). The competing pathway leading to the opposite enantiomer *ent*‐**6a** was found to be 2.4 kcal mol^−1^ higher in energy (shown in grey, see Supporting Information for a full discussion of all stereochemically distinct pathways). These results provide a compelling explanation for the formation of oxime ester **6a** as the major enantiomer and highlight the dual catalytic role of the CPA in this transformation.

**Scheme 3:**
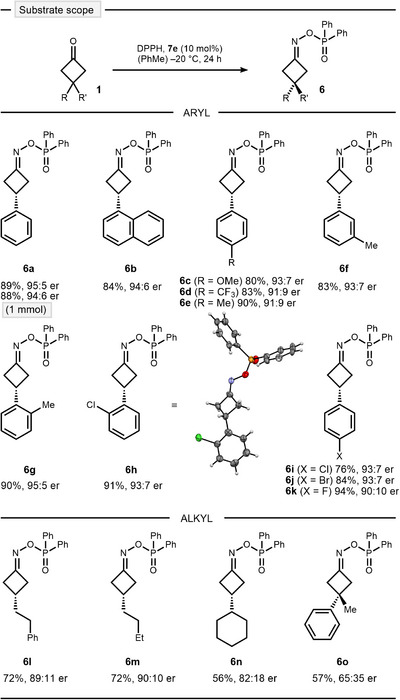
Substrate scope of the asymmetric condensation.

With the optimal conditions in hand, we briefly explored the scope of the condensation (Scheme 3). The reaction was easily scalable without any loss of reactivity or selectivity (**6a**). We were able to reisolate CPA **7e** in 91% yield and reuse it with no detectable reduction of the catalytic activity.^[^ ^37^ ^]^ The condensation tolerated a variety of electronic (**6b–d**) and steric perturbations (**6e–g**) on the aromatic ring, including halogen substitution (**6h–k**). Absolute configuration was established by X‐ray analysis of *ortho*‐chloro oxime ester **6h**. Interestingly, alkyl substituted cyclobutanones resulted in excellent yield and good induction under the optimized conditions (**6l–n**). However, less selective results were obtained for 3,3‐disubstitution (**6o**).

**Scheme 4:**
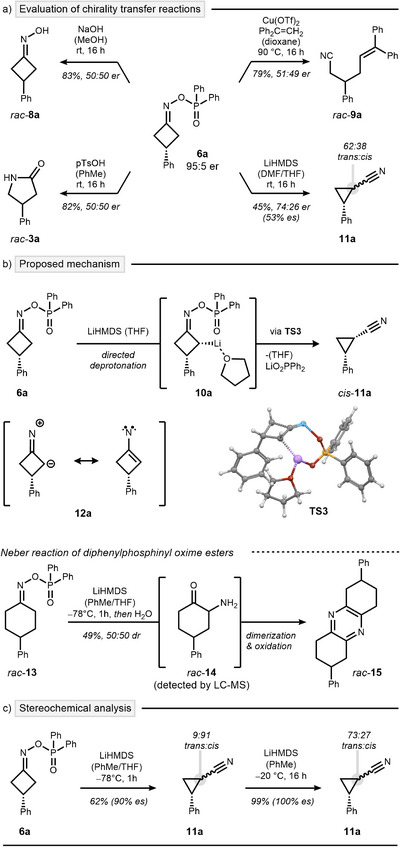
Development of a chirality transfer sequence. a) Identification of a stereospecific reaction. b) Mechanistic analysis of the ring‐contraction. c) Stereochemical analysis under optimized conditions.

The development of chirality transfer reactions from chiral oxime ester **6a** proved to be challenging. Established reaction sequences resulted in a complete loss of the stereochemical information inherent to the oxime ester. Specifically, treatment under alkaline^[^ ^34^ ^]^ and acidic^[^ ^38^ ^]^ conditions led to the formation of oxime *rac‐* **8a** and lactam *rac‐* **3a**, respectively, whereas nitrile *rac‐* **9a** was generated via a radical cascade (Scheme 4a).^[^ ^39^ ^]^ To overcome racemization, we adopted a strategy based on site‐selective, irreversible deprotonation using a non‐nucleophilic base. We hypothesized that this process could be stereospecific, with the phosphonic ester selectively directing the deprotonation. The resulting anion **10a** was then expected to undergo a Neber rearrangement.^[^ ^40^, ^41^ ^]^

However, upon testing this sequence, a mixture of cyanocyclopropane diastereomers *trans*‐**11a** and *cis*‐**11a** was obtained (Scheme 4a, bottom right).^[^ ^42^, ^43^ ^]^ This outcome was not only unexpected but also particularly exciting due to the small, yet measurable, enantiospecificity (es) of 53%.

Mechanistically, the ring contraction is proposed to proceed through a Beckmann fragmentation coupled with concurrent C─C bond formation (Scheme 4b). DFT analysis revealed a transition structure for the synchronous ring contraction and elimination of Li(O_2_PPh_2_) (**TS3**) from the preferred configurational isomer **10a·**THF, with a TS energy of 19.9 kcal mol^−1^. A singlet nitrene intermediate **12a**, which has been proposed in other Neber reactions,^[^ ^40^, ^41^ ^]^ is not a stable intermediate but rearranges to **11a** (see Supporting Information for a discussion of all considered mechanistic pathways). Mechanistic divergence from the classical Neber reaction appears to be enabled by the strained character of the cyclobutane ring. To test this hypothesis, we examined the reaction with less strained cyclohexanone‐derived oxime ester *rac*‐**13**. This led to the formation of pyrazine *rac*‐**15** through a conventional Neber rearrangement to *rac*‐**14**, followed by a condensation–oxidation sequence. (Scheme 4b, bottom).^[^ ^44^ ^]^

As predicted by our DFT analysis, a concerted mechanism should allow for a fully stereospecific reaction protocol toward *cis*‐**11a**. Further optimization of the reaction conditions revealed a striking effect of solvent and temperature on stereocontrol. When the reaction was performed in toluene/THF mixtures at low temperature, both stereospecificity and diastereoselectivity improved significantly (Scheme 4c, left). This underscores the pivotal role of ethereal solvents in LiHMDS disaggregation,^[^ ^45^, ^46^ ^]^ facilitating directed deprotonation at ‒78 °C. We also studied the interconversion of the isomers *cis*‐**11a** and *trans*‐**11a** under basic conditions at ‒20 °C, identifying a thermodynamic preference for the *trans*‐isomer (Scheme 4c, right).^[^ ^47^, ^48^ ^]^

Based on these results, we were wondering whether the reaction sequence can be performed in a stereodivergent one‐pot fashion, enabling efficient syntheses of cyanocyclopropanes from simple prochiral cyclobutanones.

**Scheme 5:**
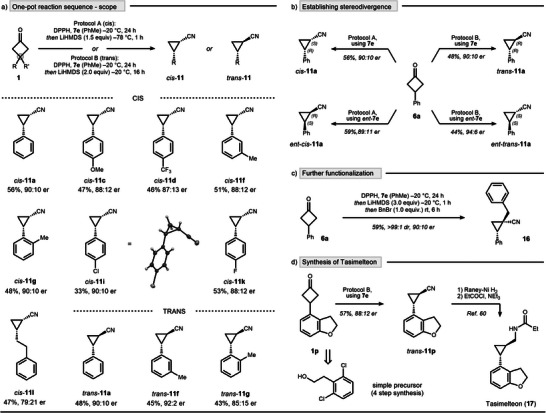
Development of a stereodivergent one‐pot nitrogen insertion protocol. a) Scope of *cis*‐ and *trans*‐selective method. For combined NMR yield and diastereoselectivity see Supporting Information. b) Stereodivergent syntheses of all four possible isomers of **11a**. c) Further functionalization by trapping the lithiated cyanocyclopropane intermediate. d) Application through the synthesis of tasimelteon.

In this connection, two protocols A and B were developed, differing in base equivalents (equiv), temperature, and applied time for rearrangement, which allowed one‐pot nitrogen insertions in a highly stereospecific manner. Recognizing the inherent synthetic challenges of achieving *cis*‐selective access to optically‐active cyanocyclopropanes,^[^ ^49^, ^50^, ^51^, ^52^, ^53^, ^54^, ^55^ ^]^ we commenced our scope explorations using protocol A (Scheme 5a, top). A range of substrates with diverse electronic and steric perturbations at the aromatic core was well‐tolerated, affording cyanocyclopropanes **11a–l** with high selectivity and in synthetically useful yields. Absolute configuration was established by X‐ray analysis of *para*‐chloro cyanocyclopropane *cis*‐**11i**. The protocol was also easily adjustable to access *trans*‐isomers by using protocol B, as demonstrated by the exemplarily entries *trans*‐**11a**, *trans*‐**11f**, and *trans*‐**11g** (Scheme 5a, bottom). Furthermore, all four possible cyclopropane isomers can be accessed from phenylcyclobutanone **1a** using a divergent reaction set‐up. Specifically, when using CPA **7e** as the catalyst, *cis*‐**11a** and *trans*‐**11a** were accessible in (*R,S*) and (*R,R*)‐configuration, respectively. Analogously, CPA *ent*‐**7e** enabled the synthesis of the (*S,R*) and (*S,S*)‐isomers (Scheme 5b). We also explored further functionalization of the corresponding cyanocyclopropanes. In this context, we realized that in cases when functionalization occurs via deprotonation followed by electrophilic trapping, it can be seamlessly integrated into our reaction protocol. This is exemplified by benzylation, which proceeded with complete diastereoselectivity, yielding cyanocyclopropane **16** in 59% yield and 90:10 er (Scheme 5c).

To further highlight the synthetic utility of our methodology, we applied it to the formal synthesis of tasimelteon, an FDA‐approved drug for treating non‐24‐hour sleep–wake disorder (Scheme 5d).^[^ ^56^, ^57^, ^58^ ^]^ Therefore, easily accessible cyclobutanone **1p** was subjected to the *trans*‐selective protocol B using CPA **7e** as the catalyst. Gratifyingly, we were able to isolate cyclopropane *trans*‐**11p** in 57% yield and with precise control over the two newly formed stereocenters. As previously described, cyanocyclopropane *trans*‐**11p** can be converted to tasimelteon (**17**) by reduction and propanylation steps.^[^ ^59^, ^60^ ^]^

## Conclusion

This study presents a novel one‐pot sequence that converts prochiral cyclobutanones to cyanocyclopropanes bearing two consecutive stereocenters. Enabled by a CPA organocatalyst, this transformation initially achieves selective condensation with DPPH. Upon the addition of LiHMDS a consecutive rearrangement is triggered, which occurs stereospecifically, enabling the transfer of chirality from the oxime ester intermediate to the cyanocyclopropane product. A detailed investigation of both key steps provided valuable mechanistic insights, underscoring the significant potential of oxime esters in asymmetric synthesis. These results not only advance axial‐to‐point chirality transfer reactions but also open up new possibilities for the synthesis of chiral nitriles as valuable building blocks.

## Conflict of Interests

The authors declare no conflict of interest.

## Supporting information



Supporting Information

Supporting Information

Supporting Information

Supporting Information

## Data Availability

The data that support the findings of this study are available in the Supporting Information of this article.
