# Supplementary material for: Nitrogen Insertion via Asymmetric Condensation and Chirality Transfer: A Stereodivergent Entry to Cyanocyclopropanes
Source: Angew Chem Int Ed Engl. 2025 Apr 14;64(24):e202503056. doi: 10.1002/anie.202503056 (PMC12144887; doi:10.1002/anie.202503056)

Nitrogen Insertion via Asymmetric Condensation and Chirality Transfer: A Stereodivergent Entry to Cyanocyclopropanes

Marlene Arnold, Jasmin Hammes, Mike Ong, Christian Mück-Lichtenfeld, Johannes M. Wahl*

Department Chemie, Johannes Gutenberg-Universität Duesbergweg 10-14, 55128 Mainz, Germany

Supporting Information

Contents

1. General 1
2. Synthesis of starting materials..3
3. Optimization of the enantioselective condensation 17
4. DFT calculations for the asymmetric condensation 25
5. Substrate scope cyclobutanone oxime esters 35
6. Test Experiments Application 51
7. Optimization of the ring contraction reaction.....................................54
8. Mechanistic Investigation for the Ring Contraction Reaction 57
9. Substrate Scope Cyclopropanes 63
10. Crystallographic data 73
11. References 77
12. NMR Spectra and HPLC Traces 79

1. General

Reaction Set-up: Chemicals were purchased from *Alfa Aesar*, *Acros Organics*, *Sigma Aldrich*, *BLDpharm*, *FluoroChem*, *Carbolution* or *ABCR* and (unless otherwise stated) used as received. All reactions involving air or moisture sensitive reagents were carried out in oven- (125 °C) and flame-dried glassware under nitrogen atmosphere using standard *Schlenk* techniques. Dry solvents were collected from an *MBraun MB SPS-800* (Et_2O: MB-KOL-A and MB-KOL MT2-250, THF: 2 × MB-KOL MT2-150°C, CH_2Cl_2: 2 × MB-KOL-A). A positive argon pressure was used to pass the solvents through the columns. Unless otherwise noted, all work-up and purification procedures were carried out with pre-distilled technical grade solvents. Purification was performed either with standard column chromatography techniques using *Geduran*® Si 60 silica gel (0.063-0.200 mm, *Merck*), on an automated flash chromatography system *Biotage Isolera One* utilizing *Biotage Sfär Silica D-Duo* 60 µm columns (5 g, 25 g, 100 g) or with preparative thin layer chromatography (TLC) using glass plates coated with SiO_2-60 F254 and 0.5 mm thickness from *Merck*. Glass silica gel plates 60 F254 (*Merck*) were used for analytic thin layer chromatography applying either UV light (254/366 nm), $KMnO_4$ (1.5 g $KMnO_4$, 5 g $NaHCO_3$ and 5 mL NaOH 10% in 200 mL H_2O), CAM (0.5g $Ce(NH_4)_2(NO_3)_6$ and 24.0 g of $(NH_4)_6Mo_7O_{24}\cdot 4H_2O$, 28 mL H_2SO_4 in 200 mL H_2O) for detection.

Analytical methods: Melting points (**M.P.**) were measured on a *Büchi* B-540 melting-point apparatus and are reported uncorrected. Infrared (**IR**) spectra were obtained on a Tensor 27 spectrometer (*Bruker*) using a diamond ATR unit and are reported in wavenumbers (cm^{-1}). Bands are characterized as broad (br), strong (s), medium (m), and weak (w). Nuclear magnetic resonance (**NMR**) spectra were recorded by the analytical department of the Department Chemie at Johannes Gutenberg-Universität Mainz. The following spectrometers were used: Avance III HD 300 (*Bruker*), Avance II 400 (*Bruker*), Avance III HD 400 (*Bruker*), and Avance III 600 equipped with a cryo-probe head (Bruker). Spectra were recorded at 26 °C (unless otherwise noted). Chemical shifts are reported in ppm with the solvent resonance as the internal standard (^1H NMR $CHCl_3$: δ = 7.26 ppm, C_6HD_5: δ = 7.16 ppm, $(CHD_2)(CD_3)SO$: δ = 2.50 ppm; ^{13}C NMR $CDCl_3$: δ = 77.16 ppm, C_6D_6 δ = 128.06 ppm, $(CD_3)_2SO$: δ = 39.5 ppm). Chemical shifts of ^{19}F NMR are referenced to internal or external standards according to *Togni* and coworkers.[1] The data is reported as follows: chemical shift, multiplicity (s = singlet, d = doublet, t = triplet, q = quartet, p = pentet, br = broad, m = multiplet or combinations of these), coupling constants (Hz) and integration. Apparent multiplicity, which occurs as a result of accidental equality of coupling constants to magnetically non-equivalent protons, is marked as *app.* High Resolution Mass Spectrometry (**HRMS**) was performed by the analytical department of the Department Chemie at Johannes Gutenberg-Universität Mainz. Spectra were recorded on a *Thermo-Fisher Scientific* DFS (GC-MS, ionization via electron ionization (EI) or chemical ionization (CI)) or on an *Agilent* 6545 Q-ToF (LC-MS, ionization via electron spray ionization (ESI), atmospheric-pressure chemical ionization (APCI)). Signals are reported as mass to charge ratio *m/z.*

Optical rotations were measured on a Perkin-Elmer 241 polarimeter at 589 nm wavelength (Na D-line) using a standard 10 cm cell (1 mL). Specific rotations, $[\alpha]_D^T$, are reported in °·mL/(g·dm) at the specific temperature. Concentrations (c) are given in grams per 100 mL of the specific solvent. Analytical high-performance liquid chromatography (**HPLC**) measurements were performed on the following systems: *Knauer* HPLC Pump Smartline 1000 with degassing unit, *Knauer* Autosampler Smartline 3950, *Knauer* UV-detector Smartline 2550, *Knauer* RI-detector Smartline 2300 or *Agilent Technologies* 1260 Infinity II HPLC-System with a binary pump, high performance degassing unit, automized liquid sampler, thermostatic column oven and diode array detector. Separation was performed using Lux® iCellulose-5 (4.6 × 250 nm × 5 µm, Phenomenex Ltd.), Lux® Cellulose-1 (4.6 x 250 nm x 5 µm, Phenomenex Ltd.), Lux® Amylose-1 (4.6 x 250 nm x 5 µm, Phenomenex Ltd.), Lux® i-Amylose-3 (4.6 x 250 nm x 5 µm, Phenomenex Ltd.), or Reprosil Chiral-AMS (4.6 x 250 nm x 5 µm, Dr Maisch GmBH.).

X-Ray diffraction: Data sets for compounds **[6h]**, **[6m]** **[*cis*-11a]** and **[*cis*-11i]** were collected by *D. Schollmeyer* with a STOE Diffractometer system. Programs used: data col-lection X-Area WinXpose 2.022.0 (X-RED and X-AREA, Stoe & Cie, 2019), cell-refinement: X-Area Recipe 1.36.0 (X-RED and X-AREA, Stoe & Cie, 2019), structure solution SHELXT-2014 (G.M. Sheldrick Acta Cryst., 2015, A71, 3-8); structure refinement SHELXT-2018/3 (A. L. Spek Acta Cryst., 2015, C71, 3-8) and graphics Platon (A. L. Spek Acta Cryst., 2009, D65, 148-155). R-values are given for observed reflections, and ωR2 values are given for all reflections.

2. Synthesis of starting materials

2.1. Substrate synthesis

Cyclobutanones **[1b], [1f-j]** were prepared according to the methods reported in the literature.[2,3]

General procedure A (GP-A) for the [2+2] cycloaddition of keteneiminium salt:

A Schlenk tube was charged with dimethylacetamide (1.20 eq.) in 1,2-dichloroethane (0.5 M). The reaction solution was cooled to room temperature with a water bath. Tf_2O (2.00 eq.) was added dropwise, and the reaction mixture was stirred at room temperature for 10 min. A solution of the corresponding alkene (1.00 eq.) and 2,6-lutidine (2.00 eq.) in 1,2-dichloroethane (2.0 M) was added dropwise to the reaction mixture. The solution was stirred at 90 °C for 8 h. After cooling to room temperature water (20 mL) was added. The reaction mixture was stirred at 90 °C for 16 h. The mixture was allowed to cool to room temperature and the layers were separated. The aqueous layer was extracted with CH_2Cl_2 (4 × 50 mL). The combined organic layers were dried over $MgSO_4$, filtered and the solvent was removed under reduced pressure. The product was separated *via* flash column chromatography with the conditions given in the corresponding entry.

General procedure B (GP-B) for the [2+2] cycloaddtion of dichloroketene via ultrasound irradiation and subsequent dehalogenation:

According to a procedure of *Ong et al*.[3], in a Schlenk flask under inert atmosphere, Zn powder (2.00 eq.) was suspended in anhydrous Et_2O (1.3 M). Olefin (1.00 eq.) was added to the suspension and the flask was placed in a sonication bath. The mixture was sonicated while a solution of trichloroacetyl chloride (1.50 eq.) in Et_2O (2.0 M) was added dropwise over a period of 40 min. The sonication bath was cooled by the addition of ice to maintain the temperature <25 °C. After complete addition, the mixture was sonicated until TLC showed full conversion. The mixture was filtered through a pad of Celite® and washed with Et_2O. The filtrate was washed with water (2 × 50 mL), aq. sat. $NaHCO_3$ solution (4 × 50 mL) and brine (2 × 50 mL). The organic phase was dried over $MgSO_4$, filtered and concentrated under reduced pressure. The crude reaction mixture was used without further purification.

In a round bottom flask, the crude mixture was dissolved in glacial acetic acid (0.5 M). The solution was cooled with a water bath and Zn dust (4.00 eq.) was slowly added. The reaction mixture was heated to 80 °C for 16 h. After cooling to room temperature, the mixture was filtered through a pad of celite and washed with CH_2Cl_2. The solvent was removed under reduced pressure and the residue was redissolved in Et_2O. The organic phase was washed with water (3 × 50 mL), aq. sat. $NaHCO_3$ solution (3 × 50 mL) and brine (2 × 50 mL). The organic layer was dried over $MgSO_4$, filtered and the solvent was removed under reduced pressure. The product was isolated *via* flash column chromatography with the conditions given in the corresponding entry.

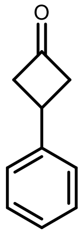

3-Phenylcyclobutan-1-one [1a]

Following **GP-A** using styrene (3.12 g, 30.0 mmol, 1.00 eq.) the product (3.48 g, 23.8 mmol, 79%) was obtained *via* flash column chromatography (SiO_2, *n*-pentane:EtOAc, 95:5 → 90:10, stained with $KMnO_4$) and bulb to bulb distillation (83 °C, 0.11 mbar) as a colorless oil.

^{1}H NMR (400 MHz, $CDCl_3$): δ = 7.34 – 7.28 (m, 2H), 7.27 – 7.18 (m, 3H), 3.63 (*app.* dq, J ≈ 9.2, 7.5 Hz, 1H), 3.51 – 3.38 (m, 2H), 3.27 – 3.13 (m, 2H). **^{13}C NMR (101 MHz, $CDCl_3$)**: δ = 207.0, 143.7, 128.8, 126.8, 126.6, 54.8, 28.6. The spectroscopic data was in agreement to those previously reported.[4]

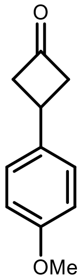

3-(4-Methoxyphenyl)cyclobutan-1-one [1c]

Following **GP-A** using 4-methoxystyrene (1.34 g, 10.0 mmol, 1.00 eq.) the product (156 mg, 880 µmol, 9%) was obtained *via* flash column chromatography (SiO_2, *n*-pentane:EtOAc, 70:30, stained with $KMnO_4$) as a colorless oil.

^{1}H NMR (600 MHz, $CDCl_3$): δ = 7.24 – 7.20 (m, 2H), 6.92 – 6.87 (m, 2H), 3.81 (s, 3H), 3.68 – 3.59 (m, 1H), 3.52 – 3.42 (m, 2H), 3.25 – 3.16 (m, 2H). **^{13}C NMR (151 MHz, $CDCl_3$)**: δ = 207.3, 158.5, 135.8, 127.7, 114.2, 55.5, 55.0, 27.9. The spectroscopic data was in agreement to those previously reported.[5]

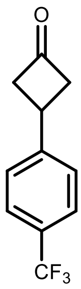

3-(4-(Trifluoromethyl)phenyl)cyclobutan-1-one [1d]

Following **GP-A** using 4-(trifluoromethyl)styrene (1.72 g, 10.0 mmol, 1.00 eq.) the product (561 mg, 2.62 mmol, 26%) was obtained *via* flash column chromatography (SiO_2, *n*-pentane:EtOAc, 90:10, stained with $KMnO_4$) and bulb-to-bulb distillation (85 °C, 0.20 mbar) as a colorless oil.

^{1}H NMR (600 MHz, $CDCl_3$): δ = 7.67 – 7.42 (m, 4H), 3.78 – 3.68 (m, 1H), 3.59 – 3.53 (m, 2H), 3.30 – 3.20 (m, 2H). **^{13}C NMR (151 MHz, $CDCl_3$)**: δ = 205.8, 147.7, 129.2 (q, $^2J_{C\text{-}F}$ = 32.5 Hz), 127.1, 125.8 (q, $^3J_{C\text{-}F}$ = 3.8 Hz), 124.2 (q, $^1J_{C\text{-}F}$ = 271.8), 54.8, 28.5. **^{19}F-NMR (376 MHz, $CDCl_3$):** δ = –62.4. The spectroscopic data was in agreement to those previously reported.[6]

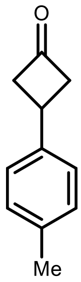

3-(*p*-Tolyl)cyclobutan-1-one [1e]

Following **GP-A** using 4-methylstyrene (1.18 g, 10.0 mmol, 1.00 eq.) the product (1.37 g, 8.55 mmol, 86%) was obtained *via* flash column chromatography (SiO_2, *n*-pentane:EtOAc, 90:10, stained with $KMnO_4$) as a colorless oil.

^{1}H NMR (400 MHz, $CDCl_3$): δ = 7.23 – 7.10 (m, 4H), 3.71 – 3.58 (m, 1H), 3.56 – 3.41 (m, 2H), 3.31 – 3.16 (m, 2H), 2.35 (s, 3H). **^{13}C NMR (101 MHz, $CDCl_3$)**: δ = 207.3, 140.7, 136.4, 129.5, 126.5, 54.9, 28.2, 21.1. The spectroscopic data was in agreement to those previously reported.[4]

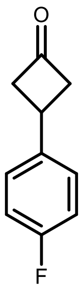

3-(4-Fluorophenyl)cyclobutan-1-one [1k]

Following **GP-A** using 1-ethenyl-4-fluorobenzene (1.22 g, 10.0 mmol, 1.00 eq.) the product (1.28 g, 7.79 mmol, 78%) was obtained *via* automated flash column chromatography (SiO_2, *n*-pentane:EtOAc, 95:5, stained with $KMnO_4$) as a colorless oil.

^{1}H NMR (400 MHz, $CDCl_3$): δ = 7.34 – 7.20 (m, 2H), 7.13 – 6.97 (m, 2H), 3.77 – 3.59 (m, 1H), 3.58 – 3.43 (m, 2H), 3.31 – 3.13 (m, 2H). **^{13}C NMR (101 MHz, $CDCl_3$)**: δ = 206.4, 161.7 (d, $^{1}J_{C\text{-}F}$ = 245.1 Hz), 139.4 (d, $^{4}J_{C\text{-}F}$ = 3.2 Hz), 128.1 (d, $^{3}J_{C\text{-}F}$ = 8.0 Hz), 115.6 (d, $^{2}J_{C\text{-}F}$ = 21.4 Hz), 55.0, 28.0. **^{19}F NMR (282 MHz, $CDCl_3$)**: δ = –116.11 (tt, *J* = 8.7, 5.2 Hz). The spectroscopic data was in agreement to those previously reported.[7]

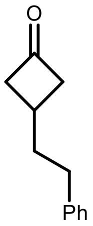

3-Phenethylcyclobutan-1-one [1l]

Following **GP-B** with minor alterations[a] using 4-phenyl-1-butene (1.10 g, 10.0 mmol, 1.00 eq.) the product (513 mg, 2.93 mmol, 29%) was obtained *via* flash column chromatography (SiO_2, *n*-pentane:EtOAc, 95:5, stained with $KMnO_4$) as a colorless oil.

^{1}H NMR (600 MHz, $CDCl_3$): δ = 7.38 – 7.11 (m, 5H), 3.28 – 3.04 (m, 2H), 2.84 – 2.58 (m, 4H), 2.43 – 2.32 (m, 1H), 1.96 – 1.90 (m, 2H). **^{13}C NMR (151 MHz, $CDCl_3$)**: δ = 208.3, 141.5, 128.6, 128.5, 126.2, 52.6, 38.1, 34.7, 23.5. The spectroscopic data was in agreement to those previously reported.[8]

[a] Reduction was performed at 90 °C for 96 h.

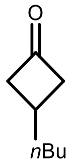

3-Butylcyclobutan-1-one [1m]

Following **GP-B** using 1-hexene (2.52 g, 30.0 mmol, 1.00 eq.) the product (1.57 g, 12.4 mmol, 41%) was obtained *via* flash column chromatography (SiO_2, *n*-pentane:EtOAc, 97:3, stained with $KMnO_4$) as a colorless oil.

^{1}H NMR (400 MHz, CDCl$_3$): δ = 3.19 – 3.06 (m, 2H), 2.72 – 2.57 (m, 2H), 2.41 – 2.26 (m, 1H), 1.63 – 1.52 (m, 2H), 1.39 – 1.22 (m, 4H), 0.91 (t, J = 7.0 Hz, 3H). **^{13}C NMR (101 MHz, CDCl$_3$)**: δ = 209.1, 52.7, 36.2, 30.6, 24.0, 22.6, 14.2. The spectroscopic data was in agreement to those previously reported.[9]

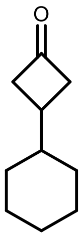

3-Cyclohexylcyclobutan-1-one [1n]

Following **GP-B** using vinylcyclohexane (1.10 g, 10.0 mmol, 1.00 eq.) the product (1.89 g, 8.40 mmol, 84%) was obtained *via* flash column chromatography (SiO_2, *n*-pentane:EtOAc, 95:5, stained with $KMnO_4$) as a colorless oil.

^{1}H NMR (600 MHz, CDCl$_3$): δ = 3.14 – 2.94 (m, 2H), 2.84 – 2.65 (m, 2H), 2.12 – 2.00 (m, 1H), 1.80 – 1.63 (m, 5H), 1.36 – 1.06 (m, 4H), 1.01 – 0.81 (m, 2H). **^{13}C NMR (151 MHz, CDCl$_3$)**: δ = 208.7, 50.9, 43.9, 31.0, 30.1, 26.3, 26.2. The spectroscopic data was in agreement to those previously reported.[7]

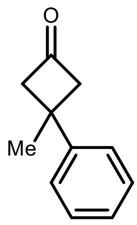

3-Methyl-3-phenylcyclobutan-1-one [1o]

Following **GP-B** using prop-1-en-2-ylbenzene (1.77 g, 15.0 mmol, 1.00 eq.) the product (1.08 g, 6.72 mmol, 45%) was obtained *via* automated flash column chromatography (SiO_2, cyclohexane:EtOAc, 97:3, stained with $KMnO_4$) as a colorless oil.

^{1}H NMR (600 MHz, $CDCl_3$): δ = 7.41 – 7.35 (m, 2H), 7.34 – 7.30 (m, 2H), 7.28 – 7.22 (m, 1H), 3.53 – 3.42 (m, 2H), 3.18 – 3.07 (m, 2H), 1.61 (s, 3H). **^{13}C NMR (151 MHz, $CDCl_3$)**: δ = 206.9, 148.4, 128.7, 126.4, 125.8, 59.4, 34.1, 31.2. The spectroscopic data was in agreement to those previously reported.[10]

3-(2,3-Dihydrobenzofuran-4-yl)cyclobutan-1-one [1p]

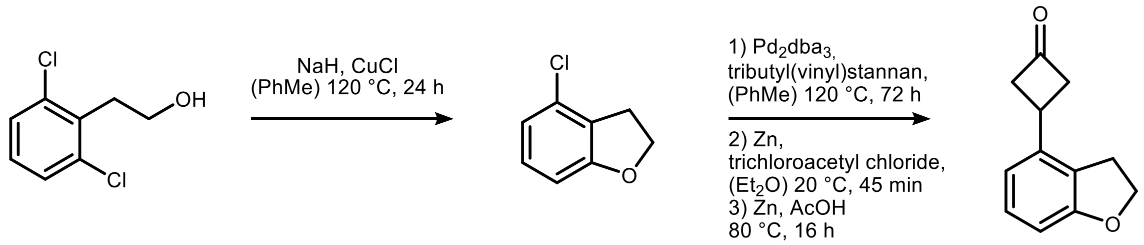

Following a procedure by *Zhu et al.*[11], 2,6-dichlorophenyl ethanol (3.44 g, 18.0 mmol, 1.00 eq.) was dissolved in dry toluene (70 mL) in a Schlenk flask. Sodium hydride (864 mg, 21.6 mmol, 1.20 eq.) was added and the reaction mixture was stirred at 40 °C for 15 minutes. After cooling to room temperature, CuCl (89.1 mg, 5.0 mol%) and EtOAc (0.1 mL) was added. The resulting mixture was heated under reflux for 24 h. After cooling to room temperature, water was added (25 mL) and the phases were separated. The aqueous layer was extracted with EtOAc (3 × 25 mL) and the combined organic phase was washed with HCl (1.0 M, 20 mL), aq. sat. $NaHCO_3$ solution (20 mL) and brine (20 mL). The organic phase was dried over $MgSO_4$, filtered and concentrated under reduced pressure. 4-Chloro-2,3-dihydrobenzofuran (2.10 g, 13.6 mmol, 75%) was isolated *via* automated flash column chromatography (SiO_2, cyclohexane:EtOAc, 95:5, stained with $KMnO_4$) as a yellow oil.

^{1}H NMR (400 MHz, $CDCl_3$): δ = 7.04 (*app.* tt, J ≈ 8.0, 0.8 Hz, 1H), 6.83 (dd, J = 8.1, 0.8 Hz, 1H), 6.67 (dd, J = 7.9, 0.7 Hz, 1H), 4.61 (t, J = 8.8 Hz, 2H), 3.29 – 3.21 (m, 2H). **^{13}C NMR (101 MHz, $CDCl_3$)**: δ = 161.1, 130.8, 129.3, 126.1, 120.6, 107.8, 71.2, 29.6. The spectroscopic data was in agreement to those previously reported.[11]

Following a procedure by *Littke et al.*[12], $Pd_2(dba)_3$ (147 mg, 0.160 mmol, 1.5 mol%) and tri-*tert*-butlyphosphine (121 mg, 0.146 mL, 0.600 mmol, 6.0 mol%) were dissolved in dry 1,4-dioxane (5 mL) under N_2 atmosphere. A solution of 4-chloro-2,3-dihydro-1-benzofuran (1.55 g, 10.0 mmol, 1.00 eq.) in dry 1,4-dioxane (5 mL) and cesium fluoride

(3.34 g, 2.20 mmol, 2.20 eq.) were added. Tributyl(ethenyl)stannane (3.33 g, 3.07 mL, 1.05 mmol, 1.05 eq.) was added and the reaction mixture was stirred at 100 °C for 24 h. After cooling to room temperature, water (20 mL) was added, and the aqueous phase was extracted with Et_2O (3 × 20 mL). The combined organic phase was washed with water (3 × 20 mL), aq. sat. NH_4Cl solution (3 × 20 mL) and brine (3 × 20 mL), dried over $MgSO_4$ and concentrated under reduced pressure. The mixture was filtered through a short silica plug (SiO_2, cyclohexane:EtOAc, 95:5, stained with $KMnO_4$) and used without further purification.

Following **GP-B** using 4-vinyl-2,3-dihydrobenzofuran (375 mg, 2.57 mmol, 1.00 eq.) the product (173 mg, 910 µmol, 35%) was obtained *via* automated flash column chromatography (SiO_2, cyclohexane:EtOAc, 97:3, stained with $KMnO_4$) as a yellow solid.

M.P. 80 – 82 °C. **IR (neat)**: $\tilde{\nu}$ = 2922 (w), 1782 (s), 1588 (m), 1478 (w), 1454 (m), 1379 (w), 1235 (m), 1166 (w), 1107 (w), 1035 (w), 985 (m), 946 (w), 839 (w), 777 (m), 718 (w), 445 (w). **^{1}H NMR (600 MHz, $CDCl_3$)**: δ = 7.15 (t, J = 7.9 Hz, 1H, CH_{arom}), 6.80 (d, J = 8.0 Hz, 1H, CH_{arom}), 6.72 (d, J = 8.0 Hz, 1H, CH_{arom}), 4.60 (t, J = 8.7 Hz, 2H, CH_2), 3.64 (*app.* p, J ≈ 8.2 Hz, 1H, C*H*), 3.49 – 3.42 (m, 2H, CH_2), 3.32 – 3.25 (m, 2H, CH_2), 3.17 (t, J = 8.7 Hz, 2H, CH_2). **^{13}C NMR (151 MHz, $CDCl_3$)**: δ = 206.6 (C_q), 160.3 (C_q), 139.9 (C_q), 128.7 (*C*H), 125.4 (C_q), 116.9 (*C*H), 108.0 (*C*H), 71.1 (*C*H_2), 53.5 (2x*C*H_2), 28.9 (*C*H_2), 26.6 (*C*H). **HRMS (ESI):** Calculated for $C_{12}H_{13}O_2$ $[M+H]^+$: 189.0910, Found: 189.0904.

2.2. Hydroxylamine reagents

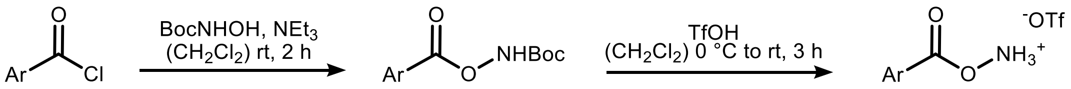

General procedure C (GP-C) for the synthesis of Boc-protected amino carbamates:

According to a procedure from *Liu et al.*[13], a solution of triethylamine (1.20 eq.) and arylchloride (1.00 eq.) in CH_2Cl_2 (0.7 M) was added dropwise to a solution of *N*-Boc-hydroxylamine (1.00 eq.) in CH_2Cl_2 (0.5 M) while cooling with an ice bath. The reaction mixture was stirred at room temperature for 2 h. Water was added, and the aqueous phase was extracted with CH_2Cl_2. The combined organic phase was dried over $MgSO_4$, filtered and the solvent was removed under reduced pressure. The product was separated *via* flash column chromatography with the conditions given in the corresponding entry.

General procedure D (GP-D) for the Boc-deprotection with triflic acid:

Following a modified procedure by *Liu et al.*[14], Boc-protected carbamate (1.00 eq.) was dissolved in CH_2Cl_2 (0.2 M). The solution was cooled with an ice bath and triflic acid (1.50 eq.) was added dropwise. The reaction mixture was stirred at room temperature for 3 h. The formed precipitate was filtered and washed with cold *n*-pentane to obtain the corresponding product.

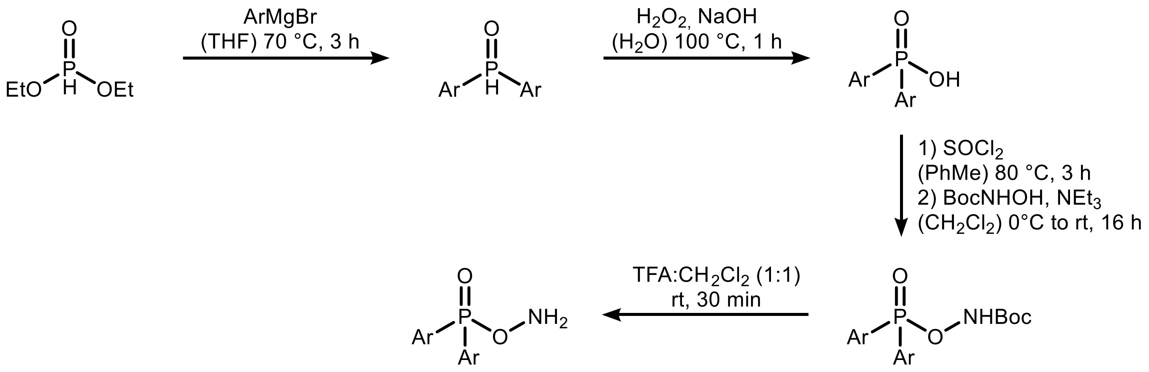

General procedure E (GP-E) for the Grignard addition to diethylphosphite:

Arylmagnesiumbromide (1.0 M in THF, 3.00 eq.) was added to a Schlenk flask under N_2-atmosphere. The solution was cooled to 0 °C and a solution of diethylphosphite (1.00 eq.) in dry THF (1.0 M) was added dropwise. The reaction mixture was stirred for 3 h under reflux and 16 h at room temperature. The mixture was cooled to 0 °C and HCl (1.0 M, 30 mL) was slowly added. The solution was extracted with EtOAc (3 × 20 mL), dried over $MgSO_4$, filtered and concentrated under reduced pressure. The product was obtained by recrystallization with the conditions given in the corresponding entry.

General procedure F (GP-F) for the oxidation with sodium hydroxide and hydrogen peroxide:

Hydrogen peroxide (35%, 40.0 eq.) was added dropwise to a suspension of phosphite (1.00 eq.) in aqueous NaOH-solution (5.0 N, 1.0 M). The reaction mixture was stirred at 100 °C for 1 h. After cooling to 0 °C, conc. HCl was added dropwise until precipitation was completed. The precipitate was collected by filtration and washed with water and Et_2O to obtain the corresponding phosphinic acid.

General procedure G (GP-G) for the synthesis of Boc-protected *O*-phosphorylated hydroxylamines:

According to a procedure from *Smulik et al.*[15], to a suspension of phosphinic acid (1.00 eq.) in dry toluene (20 mL) was added thionyl chloride (3.00 eq.) under N_2-atmosphere. The reaction mixture was stirred at 80 °C for 3 h. After cooling to room temperature, the solvent was removed under reduced pressure (Schlenkline, cooling trap). The crude phosphinic acid chloride was redissolved in dry CH_2Cl_2 (3.0 M) and used without further purification.

In an oven dried Schlenk flask *N*-Boc hydroxylamine (1.10 eq.) was dissolved in dry CH_2Cl_2 (1.0 M) under N_2-atmosphere. The mixture was cooled with an ice bath and NEt_3 (1.25 eq.) was added dropwise over a period of 10 minutes. The solution of phosphinic acid chloride (1.00 eq.) in CH_2Cl_2 (3.0 M) was added dropwise to the reaction flask over a period of 10 minutes. The mixture was allowed to warm to room temperature over night. Water (10 mL) was added, and the mixture was extracted with CH_2Cl_2. The organic phase was dried over $MgSO_4$, filtered and concentrated under reduced pressure. The product was separated *via* flash column chromatography with the conditions given in the corresponding entry.

General procedure H (GP-H) for the Boc-deprotection with TFA:

Boc-protected hydroxylamine (1.00 eq.) was dissolved in CH_2Cl_2 (1.0 M). The solution was cooled with an ice bath. TFA (1.0 M) was added and the solution was stirred at room temperature for 30 minutes. The solution was diluted with CH_2Cl_2 and water. $NaHCO_3$ was added until gas evolution stopped. The aqueous layer was extracted with CH_2Cl_2 and the combined organic phase was dried over $MgSO_4$, filtered and concentrated under reduced pressure. The residue was redissolved in CH_2Cl_2 (20 mL) and hexane was added. The product was obtained by filtration of the precipitate formed.

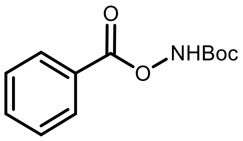

tert-Butyl (benzoyloxy)carbamate [S1]

Following **GP-C** using benzoyl chloride (1.41 g, 10.0 mmol, 1.00 eq.), *N*-Boc-hydroxylamine (1.33 g, 10.0 mmol, 1.00 eq.) and NEt_3 (1.21 g, 12.0 mmol, 1.20 eq.)

the product (1.96 g, 8.26 mmol, 83%) was obtained *via* column chromatography (SiO_2, EtOAc, stained with $KMnO_4$) as a colorless solid.

^{1}H NMR (400 MHz, CDCl3): δ = 8.16 (s, 1H), 8.14 – 8.07 (m, 2H), 7.69 – 7.57 (m, 1H), 7.54 – 7.42 (m, 2H), 1.52 (s, 9H). **^{13}C NMR (101 MHz, $CDCl_3$):** δ = 166.3, 155.7, 134.3, 130.1, 128.8, 127.0, 83.6, 28.2. The spectroscopic data was in agreement to those previously reported.[13]

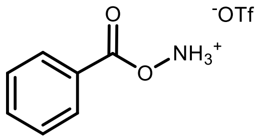

O-Benzoylhydroxylammonium trifluoromethanesulfonate [S2]

Following **GP-D** using *tert*-butyl (benzoyloxy)carbamate [**S1**] (949 mg, 4.00 mmol, 1.00 eq.) and triflic acid (900 mg, 6.00 mmol, 1.50 eq.) the product 888 mg, 3.09 mmol, 77%) was obtained after precipitation as a colorless solid.

^{1}H NMR (400 MHz, DMSO): δ = 7.96 (m, 3H), 7.80 – 7.70 (m, 1H), 7.67 – 7.54 (m, 3H), 7.50 (m, 1H). **^{13}C NMR** (101 MHz, DMSO): δ = 167.4, 164.8, 134.4, 132.9, 129.3, 128.62, 124.7. The spectroscopic data was in agreement to those previously reported.[14]

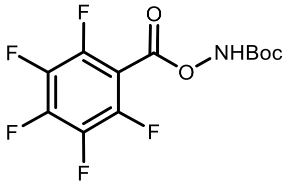

***tert*-Butyl ((perfluorobenzoyl)oxy)carbamate [S3]**

Following **GP-C** using pentafluorobenzoyl chloride (1.66 g, 7.22 mmol, 1.00 eq.), *N*-Boc-hydroxylamine (961 mg, 7.22 mmol, 1.00 eq.) and NEt_3 (730 mg, 7.22 mmol, 1.00 eq.) the product (2.11 g, 6.45 mmol, 89%) was obtained *via* column chromatography (SiO_2, pentane:EtOAc 90:10, stained with $KMnO_4$) as a colorless solid.

^{1}H NMR (400 MHz, $CDCl_3$): δ = 8.06 (s, 1H), 1.52 (s, 9H). **^{13}C NMR (101 MHz, $CDCl_3$):** δ = 158.9, 154.9, 146.0 (d, J = 260.1 Hz), 144.3 (d, J = 261.9 Hz), 138.0 (d, J = 247.0 Hz), 105.1, 84.4, 28.1. **^{19}F NMR (376 MHz, $CDCl_3$):** δ = –133.64 (dq, J = 19.2, 6.9 Hz), –143.63 (tt, J = 21.1, 5.6 Hz), –157.37 - –157.58 (m). The spectroscopic data was in agreement to those previously reported.[16]

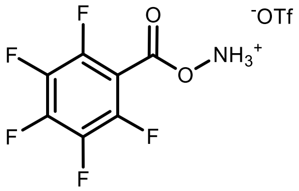

O-(Perfluorobenzoyl)hydroxylammonium trifluoromethanesulfonate [S4]

Following **GP-D** using *tert*-butyl ((perfluorobenzoyl)oxy)carbamate [**S3**] (327 mg, 1.00 mmol, 1.00 eq.) and triflic acid (225 mg, 1.50 mmol, 1.50 eq.) the product (276 mg, 0.732 mmol, 73%) was obtained after precipitation as a colorless solid.

^{13}C NMR (101 MHz, DMSO): δ = 159.9, 145.7 (d, J = 4.3 Hz), 143.1 (dd, J = 7.8, 4.0 Hz), 140.9, 138.6, 136.1 (d, J = 5.0 Hz), 120.7 (q, J = 322.1 Hz), 109.3 (d, J = 20.4 Hz). **^{19}F NMR (376 MHz, DMSO):** δ = -77.56, -139.90 – -140.64 (m), -150.59 (tq, J = 21.0, 3.6 Hz), -161.13 (tt, J = 22.0, 5.6 Hz). The spectroscopic data was in agreement to those previously reported.[14]

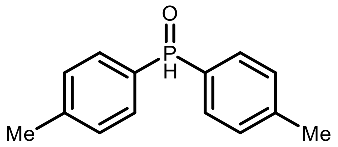

Di-*p*-tolylphosphine oxide [S5]

Following **GP-E** using 4-methylphenylmagnesiumbromide (1.0 M in THF, 60 mL, 60.0 mmol, 3.00 eq.) and diethylphosphite (2.76 g, 20.0 mmol, 1.00 eq.) the product (1.97 g, 8.55 mmol, 43%) was obtained was obtained by recrystallisation from Et_2O (30 mL) as a colorless powder.

^{1}H NMR (400 MHz, CDCl$_3$): δ = 8.02 (d, J = 478.0 Hz, 1H), 7.61 – 7.53 (m, 4H), 7.29 (dd, J = 8.0, 2.6 Hz, 4H), 2.39 (s, 6H). **^{13}C NMR (101 MHz, CDCl$_3$)**: δ = 143.2 (d, J = 2.9 Hz), 130.9 (d, J = 11.8 Hz), 129.7 (d, J = 13.2 Hz), 128.5 (d, J = 103.9 Hz), 21.8. **^{31}P NMR (162 MHz, CDCl$_3$):** δ = 22.28. The spectroscopic data was in agreement to those previously reported.[17]

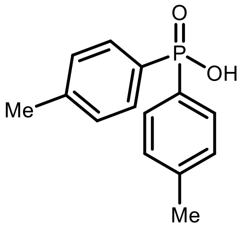

Di-*p*-tolylphosphinic acid [S6]

Following **GP-F** using di-*p*-tolylphosphine oxide [**S5**] (1.97 g, 8.55 mmol, 1.00 eq.) the product (2.07 g, 8.39 mmol, 98%) was obtained as a colorless powder.

^{1}H NMR (400 MHz, CDCl$_3$): δ = 8.86 (s, 1H), 7.71 – 7.45 (m, 4H), 7.12 (dd, J = 8.0, 3.1 Hz, 4H, C$H_{arom.}$), 2.34 (s, 6H). **^{13}C NMR (101 MHz, CDCl$_3$)**: δ = 142.1 (d, J = 2.9 Hz), 131.34 (d, J = 10.9 Hz), 130.0 (d, J = 142.4 Hz), 129.06 (d, J = 13.7 Hz), 21.7.

^{31}P NMR (162 MHz, $CDCl_3$): δ = 33.88. The spectroscopic data was in agreement to those previously reported.[18]

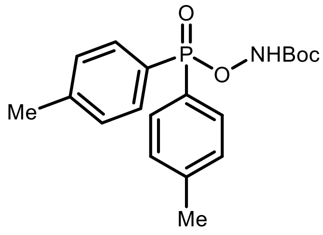

***tert*-Butyl ((di-*p*-tolylphosphoryl)oxy)carbamate [S7]**

Following **GP-G** using di-*p*-tolylphosphinic acid [**S6**] (2.65 g, 10.0 mmol, 1.00 eq.) the product (3.00 g, 8.30 mmol, 83%) was obtained *via* automated flash column chromatography (SiO_2, cyclohexane:EtOAc (95:5 → 75:25)) as a colorless powder.

M.P.: 164 – 169 °C. **IR (neat)**: $\tilde{\nu}$ = 3090 (w), 2980 (w), 2895 (w), 1750 (m), 1719 (m), 1603 (w), 1368 (m), 1273 (w), 1253 (m), 1225 (s), 1209 (m), 1163 (m), 1129 (s), 1111 (m), 1082 (m), 836 (m), 810 (m), 770 (w), 744 (m), 710 (m), 671 (s), 622 (w), 544 (m), 527 (s), 483 (m), 465 (m), 452 (m), 430 (m), 414 (m). **^{1}H NMR (400 MHz, $CDCl_3$):** δ = 8.78 (d, *J* = 6.1 Hz, 1H, N*H*), 7.87 – 7.77 (m, 4H, C$H_{arom.}$), 7.24 (dddd, *J* = 7.6, 3.5, 1.6, 0.9 Hz, 4H, C$H_{arom.}$), 2.37 (s, *J* = 0.9 Hz, 6H, CH_3), 1.39 (s, 9H, CH_3). **^{13}C NMR (101 MHz, $CDCl_3$):** δ = 156.2 (d, J = 5.3 Hz, C_q), 143.4 (d, J = 2.9 Hz, C_q), 132.5 (d, J = 10.7 Hz, $CH_{arom.}$), 129.3 (d, J = 13.8 Hz, $CH_{arom.}$), 125.9 (d, J = 138.2 Hz, C_q), 82.7 (C_q), 28.2 (CH_3), 21.8 (CH_3). **^{31}P NMR (162 MHz, $CDCl_3$):** δ = 41.54. **HRMS (ESI):** Calculated for $C_{19}H_{24}NO_6PNa$ [M+Na]$^+$: 416.1233, Found: 416.1227.

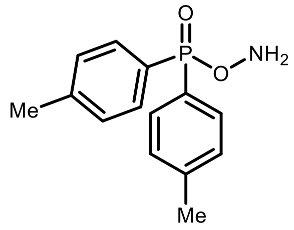

***O*-(di-*p*-tolylphosphinyl))hydroxylamine [S8]**

Following **GP-H** using *tert*-butyl ((di-*p*-tolylphosphoryl)oxy)carbamate [**S7**] (361 mg, 1.00 mmol, 1.00 eq.) the product (229 mg, 880 µmol, 88%) was obtained as a colorless powder.

^{1}H NMR (400 MHz, $CDCl_3$): δ = 7.77 – 7.66 (m, 4H), 7.31 – 7.24 (m, 4H), 2.39 (s, 6H). **^{13}C NMR (101 MHz, $CDCl_3$)**: δ = 143.7 (d, J = 2.9 Hz, C_q), 132.0 (d, J = 10.3 Hz, $CH_{arom.}$), 130.2 (d, J = 13.7 Hz, $CH_{arom.}$), 126.9 (d, J = 138.3 Hz, C_q), 21.8 (CH_3). **^{31}P NMR (162 MHz, $CDCl_3$):** δ = 36.99. The spectroscopic data was in agreement to those previously reported.[18]

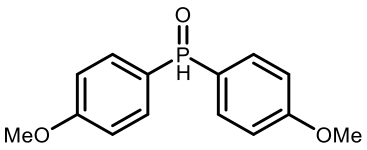

Bis(*p*-methoxyphenyl)phosphine oxide [S9]

Following **GP-E** using 4-methoxyphenylmagnesiumbromide (1.0 M in THF, 30 mL, 30.0 mmol, 3.00 eq.) the product (2.02 g, 7.70 mmol, 77%) was obtained by recrystallisation from EtOAc (30 mL) as a colorless powder.

^{1}H NMR (400 MHz, $CDCl_3$): δ = 8.02 (d, J = 477.8 Hz, 1H), 7.65 – 7.52 (m, 4H), 7.05 – 6.93 (m, 4H), 3.84 (s, 6H). **^{13}C NMR (101 MHz, $CDCl_3$)**: δ = 163.0 (d, J = 2.8 Hz), 132.8 (d, J = 12.9 Hz), 123.1 (d, J = 108.1 Hz), 114.5 (d, J = 13.9 Hz), 55.5. **^{31}P NMR (162 MHz, $CDCl_3$):** δ = 21.24. The spectroscopic data was in agreement to those previously reported.[17]

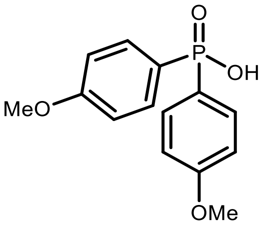

Bis-(*p*-methoxyphenyl)phosphinic acid [S10]

Following **GP-F** using bis(*p*-methoxyphenyl)phosphine oxide [**S9**] (1.97 g, 7.5 mmol, 1.00 eq.) the product (1.97 g, 7.06 mmol, 94%) was obtained as colorless powder.

^{1}H NMR (400 MHz, $CDCl_3$): δ = 9.49 (s, 1H), 7.71 – 7.44 (m, 4H), 6.83 (dq, J = 9.3, 2.6 Hz, 4H), 3.79 (s, 6H). **^{13}C NMR (101 MHz, $CDCl_3$)**: δ = 162.3 (d, J = 3.0 Hz), 133.2 (d, J = 12.0 Hz), 124.7 (d, J = 147.4 Hz), 113.9 (d, J = 14.4 Hz), 55.3. **^{31}P NMR (162 MHz, $CDCl_3$):** δ = 35.16. The spectroscopic data was in agreement to those previously reported.[18]

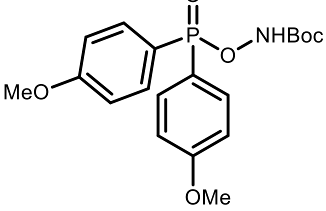

***tert*-Butyl ((bis-*p*-methoxyphenylphosphoryl)oxy)carbamate [S11]**

Following **GP-G** using bis-(*p*-methoxyphenyl)phosphinic acid [**S10**] (1.48 g, 5.0 mmol, 1.00 eq.) the product (1.65 g, 4.20 mmol, 84%) was obtained *via* automated flash column chromatography (SiO_2, cyclohexane:EtOAc (95:5 → 75:25)) as colorless powder.

M.P.: 166 – 171 °C. **IR (neat)**: $\tilde{\nu}$ = 3079 (w), 2927 (w), 1748 (m), 1598 (s), 1505 (m), 1297 (m), 1254 (s), 1217 (m), 1180 (m), 1161 (m), 1130 (s), 1082 (m), 1026 (m), 829

(m), 805 (m), 733 (m), 677 (m), 552 (s), 542 (s), 458 (m). **^{1}H NMR (600 MHz, CDCl$_3$)**: δ = 8.84 (s, 1H, N*H*), 7.92 – 7.78 (m, 4H, C$H_{arom.}$), 6.95 – 6.91 (m, 4H, C$H_{arom.}$), 3.82 (s, 6H, 2 x CH_3), 1.39 (s, 9H, 3 x CH_3).**^{13}C NMR (151 MHz, CDCl$_3$)**: δ = 163.1 (d, J = 2.9 Hz, C_q), 156.2 (d, J = 5.3 Hz, C_q), 134.4 (d, J = 11.4 Hz, *C*H), 121.0, 120.5 (d, J = 143.5 Hz, C_q), 114.0 (d, J = 14.6 Hz, *C*H), 82.6 (C_q), 55.5 (CH$_3$), 28.1 (CH$_3$). **^{31}P NMR (162 MHz, CDCl$_3$)** δ = 41.59. **HRMS (ESI):** Calculated for $C_{19}H_{25}NO_6P$ [M+H]$^+$: 395.1447, Found: 395.14487.

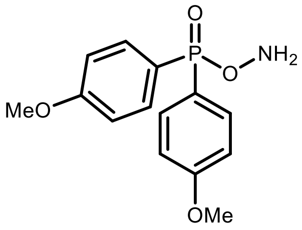

***O*-(bis-*p*-methoxyphenylphosphinyl)hydroxylamine [S12]**

Following **GP-H** using *tert*-butyl ((bis-*p*-methoxyphenylphosphoryl)oxy)carbamate [**S11**] (1.57 g, 4.0 mmol, 1.00 eq.) the product (956 mg, 3.20 mmol, 81%) was obtained as a colorless powder.

^{1}H NMR (400 MHz, CDCl$_3$): δ = 7.80 – 7.71 (m, 4H), 6.97 (dq, J = 9.3, 2.6 Hz, 4H), 3.84 (s, 6H). **^{13}C NMR (101 MHz, CDCl$_3$):** δ = 162.9 (d, J = 3.0 Hz), 133.9 (d, J = 11.3 Hz), 122.8 (d, J = 148.3 Hz), 114.3 (d, J = 14.1 Hz), 55.5. **^{31}P NMR (162 MHz, CDCl$_3$)**: δ = 39.36. The spectroscopic data was in agreement to those previously reported.[18]

3. Optimization of the enantioselective condensation

Table S1: Hydroxylamine reagent optimization, reactions were carried out on a 0.05 mmol scale, yield based on ^{1}H NMR experiments using CH_2Br_2 as an internal standard.

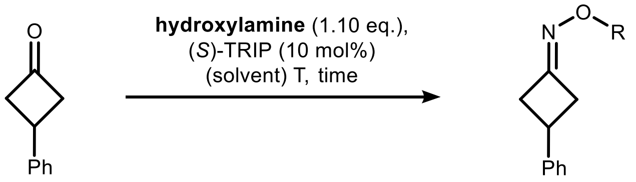

Entry	hydroxylamine regent	solvent	concentration	temperature	time	yield	er
1	2,4-$C_6H_3(NO_2)_2$-ONH_2	CH_2Cl_2	0.1 M	0 °C	6 h	90%	32:68
2	4-$C_6H_4(NO_2)CO$-ONH_2	CH_2Cl_2	0.1 M	0 °C	6 h	94%	59:41
3	C_6H_5CO-ONH_3OTf	CH_2Cl_2	0.1 M	0 °C	6 h	83%	50:50
4	C_6F_5CO-ONH_3OTf	CH_2Cl_2	0.1 M	0 °C	6 h	84%	49:51
5	Mes-SO_2-ONH_2	CH_2Cl_2	0.1 M	0 °C	6 h	0%	-
6	Ph_2PO-ONH_2	CH_2Cl_2	0.1 M	0 °C	6 h	82%	20:80
7	Ph_2PO-ONH_2	PhMe	0.05 M	0 °C	6 h	78%	15:85
8	$(4\text{-}C_6H_4Me)_2PO$-ONH_2	PhMe	0.05 M	0 °C	6 h	96%	17:83
9	$(4\text{-}C_6H_4(OMe))_2PO$-$ONH_2$	PhMe	0.05 M	0 °C	6 h	77%	19:81
10	$(4\text{-}C_6H_4(OMe))_2PO$-$ONH_2$	PhMe	0.05 M	–78 °C	24 h	64%	15:85

(*S*)-TRIP = (*S*)-3,3′-Bis(2,4,6-triisopropylphenyl)-1,1′-binaphthyl-2,2′-diyl hydrogenphosphate, Ph = Phenyl, Mes = mesityl, rt = room temperature, PhMe = toluene

Table S2: Solvent and concentration optimization, reactions were carried out on a 0.05 mmol scale, yield based on ^1H NMR experiments using CH_2Br_2 as an internal standard.

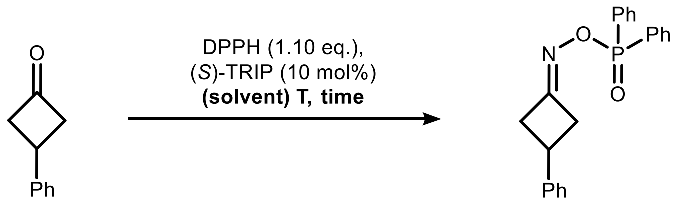

Entry	solvent	additive	concentration	time	temperature	yield	er
1	CH_2Cl_2	-	0.1 M	24 h	rt	88 %	39:61
2	CH_2Cl_2	4 Å MS	0.1 M	24 h	rt	82%	43:57
3	CH_2Cl_2	-	0.1 M	24 h	0 °C	87%	19:81
4	CH_2Cl_2	-	0.1 M	6 h	0 °C	82%	20:80
5	*n*-hexane	-	0.1 M	6 h	0 °C	44%	13:87
6	PhMe	-	0.1 M	6 h	0 °C	79%	15:85
7	PhMe	4 Å MS	0.1 M	6 h	0 °C	74%	15:85
8	PhMe	-	0.05 M	6 h	0 °C	78%	15:85
9	PhMe	-	0.01 M	6 h	0 °C	61%	16:84
10	$PhCF_3$	-	0.1 M	6 h	0 °C	35%	87:13
11	THF	-	0.1 M	6 h	0 °C	85%	70:30
12	MeCN	-	0.1 M	6 h	0 °C	90%	61:39
13	MeOH	-	0.1 M	6 h	0 °C	61%	55:45

DPPH = *O*-Diphenylphosphinylhydroxylamine, (*S*)-TRIP = (*S*)-3,3′-Bis(2,4,6-triisopropylphenyl)-1,1′-binaphthyl-2,2′-diyl hydrogenphosphate, rt = room temperature, PhMe = toluene, $PhCF_3$ = trifluorotoluene, THF = tetrahydrofurane, MeCN = acetonitrile, MeOH = methanol.

Table S3: Catalyst and additive optimization, reactions were carried out on a 0.05 mmol scale, yield based on ^{1}H NMR experiments using CH_2Br_2 as an internal standard.

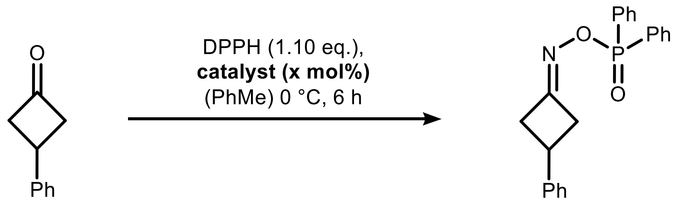

Entry	catalyst	catalyst loading	additive	yield	er
1	-	-	-	13%	-
2	-	-	4 Å MS	25%	-
3	(*S*)-TRIP	10 mol%	4 Å MS	74%	15:85
4	(*S*)-TRIP	10 mol%	diphenylphosphinic acid	69%	16:84
5	(*S*)-TRIP	10 mol%	-	78%	15:85
6	(*S*)-3,3'-(9-anthracenyl)-BINOL-P	10 mol%	-	96%	30:70
7	(*R*)-H8-TRIP	10 mol%	-	67%	84:16
8	(*S*)-3,3'-C_6F_5-BINOL-P	10 mol%	-	37%	35:65
9	(*R*)-TCYP	10 mol%	-	72%	91:9
10	(*R*)-TCYP	5 mol%	-	65%	92:8
11	(*S*)-3,3'-$SiPh_3$-BINOL-P	10 mol%	-	25%	27:73
12	Mg(O*t*Bu)$_2$	10 mol%	-	8%	-
13	Mg_2((*S*)-TRIP)	10 mol%	-	48%	18:82

DPPH = *O*-Diphenylphosphinylhydroxylamine, PhMe = toluene, (*S*)-TRIP = (*S*)-3,3′-Bis(2,4,6-triisopropylphenyl)-1,1′-binaphthyl-2,2′-diyl hydrogenphosphate, (S)-3,3'-(9-anthracenyl)-BINOL-P = (S)-3,3′-Bis(9-anthracenyl)-1,1′-binaphthyl-2,2′-diyl hydrogenphosphate, (*R*)-H8-TRIP = (11b*R*)-4-Hydroxy-2,6-bis(2,4,6-triisopropylphenyl)-8,9,10,11,12,13,14,15-octahydrodinaphtho[2,1-d:1',2'-f][1,3,2]dioxaphosphepine 4-oxide, (S)-3,3'-C_6F_5-BINOL-P = (11b*S*)-4-Hydroxy-2,6-bis[2,3,4,5,6-pentafluorophenyl]-4-oxide-dinaphtho[2,1-d:1,2-f][1,3,2]dioxaphosphepin, (*R*)-TCYP = (*R*)-4-Hydroxy-2,6-bis(2,4,6-tricyclohexylphenyl)dinaphtho[2,1-d:1',2'-f][1,3,2]dioxaphosphepine 4-oxide, (*S*)-3,3'-$SiPh_3$-BINOL-P = (S)-3,3′-Bis(triphenylsilyl)-1,1′-binaphthyl-2,2′-diyl hydrogenphosphate, O*t*Bu = *tert*-butoxide.

Table S4: Temperature optimization, reactions were carried out on a 0.05 mmol scale, yield based on ^{1}H NMR experiments using CH_2Br_2 as an internal standard.

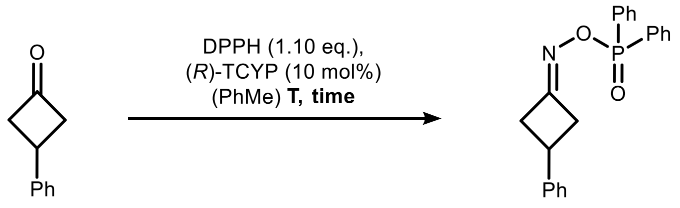

Entry	temperature	time	yield	er
1	0 °C	6 h	72 %	91:9
2	–20 °C	24 h	70%	95:5
3	–33 °C	24 h	35%	97:3

DPPH = *O*-Diphenylphosphinylhydroxylamine, (*R*)-TCYP = (*R*)-4-Hydroxy-2,6-bis(2,4,6-tricyclohexylphenyl)dinaphtho[2,1-d:1',2'-f][1,3,2]dioxaphosphepine 4-oxide, PhMe = toluene.

General procedure I (GP-I) for the optimization reaction of racemic oximes:

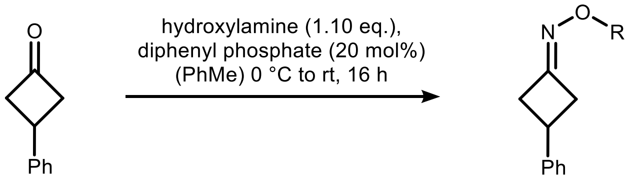

In a round bottom flask, diphenyl phosphate (20 mol%) and 3-phenylcyclobutanone (0.05 mmol, 1.00 eq.) were dissolved in toluene (0.05 M). The mixture was cooled to 0 °C. The corresponding hydroxylamine (0.055 mmol, 1.10 eq.) was added, and the reaction mixture was allowed to warm to room temperature. NEt_3 (0.05 mmol, 1.00 eq.) was added. The solvent was removed under reduced pressure and the crude product was purified *via* flash column chromatography with the conditions given in the corresponding entry.

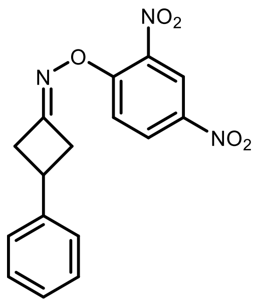

3-Phenylcyclobutan-1-one *O*-(2,4-dinitrophenyl) oxime [S13]

Following **GP-I** using 3-phenylyclobut-1-one [**1a**] (7.31 mg, 50.0 µmol, 1.00 eq.) and *O*-(2,4-dinitrophenyl)hydroxylamine (11.0 mg, 55.0 µmol, 1.10 eq.) the product (15.6 mg, 47.5 µmol, 95%) was obtained *via* automated flash column chromatography ((SiO_2, cyclohexane:EtOAc, 95:5 → 50:50, stained with $KMnO_4$) as a colorless powder.

M.P.: 93 – 101 °C. **IR (neat)**: $\tilde{\nu}$ = 1603 (s), 1524 (s), 1473 (m), 1341 (s), 1316 (m), 1285 (m), 1231 (w), 1142 (w), 1066 (w), 924 (m), 864 (m), 836 (w), 820 (m), 742 (m), 698 (m), 640 (w). **^{1}H NMR (400 MHz, $CDCl_3$)**: δ = 8.87 (d, J = 2.7 Hz, 1H, C$H_{arom.}$), 8.42 (dd, J = 9.4, 2.8 Hz, 1H, C$H_{arom.}$), 7.91 (d, J = 9.4 Hz, 1H, C$H_{arom.}$), 7.42 – 7.34 (m, 2H, C$H_{arom.}$), 7.33 – 7.27 (m, 3H, C$H_{arom.}$), 3.81 – 3.65 (m, 3H, C*H*, CH_2), 3.61 – 3.50 (m, 1H, CH_2), 3.38 – 3.19 (m, 2H, CH_2). **^{13}C NMR (101 MHz, $CDCl_3$)**: δ = 166.2 (C_q), 157.6 (C_q), 142.8 (C_q), 140.7 (C_q), 135.6 (C_q), 129.5 (*C*H), 129.0 (*C*H), 127.2 (*C*H), 126.5 (*C*H), 122.2 (*C*H), 117.2 (*C*H), 39.7 (CH_2), 39.2 (CH_2), 32.7 (*C*H). **HRMS (ESI):** Calculated for $C_{16}H_{13}N_3O_5Na$ $[M+Na]^+$: 350.0747, Found: 350.0746.

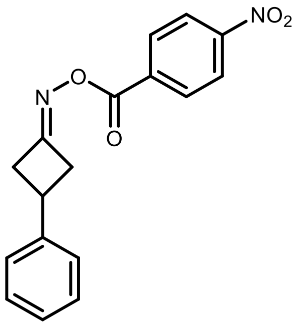

3-Phenylcyclobutan-1-one O-(4-nitrobenzoyl) oxime [S14]

Following **GP-I** using 3-phenylyclobut-1-one [**1a**] (7.31 mg, 50.0 µmol, 1.00 eq.) and *O*-(4-nitrobenzoyl)hydroxylamine (10.0 mg, 55.0 µmol, 1.00 eq.) the product (14.0 mg, 45.0 µmol, 90%) was obtained via automated flash column chromatography ((SiO_2, cyclohexane:EtOAc 95:5 → 50:50, stained with $KMnO_4$) as a colorless powder.

M.P.: 111 – 116 °C. **IR (neat)**: $\tilde{\nu}$ = 1743 (s), 1688 (w), 1606 (w), 1524 (s), 1496 (w), 1455 (w), 1398 (w), 1345 (m), 1320 (w), 1258 (s), 1235 (s), 1071 (s), 1013 (m), 872 (m), 853 (s), 777 (w), 743 (m), 715 (s), 698 (s), 670 (w), 505 (m)**^{1}H NMR (400 MHz, $CDCl_3$)**: δ = 8.35 – 8.29 (m, 2H, C$H_{arom.}$), 8.26 – 8.21 (m, 2H, C$H_{arom.}$), 7.42 – 7.34 (m, 2H, C$H_{arom.}$), 7.33 – 7.27 (m, 3H, C$H_{arom.}$), 3.81 – 3.70 (m, 1H, C*H*), 3.70 – 3.55 (m, 2H, CH_2), 3.35 – 3.21 (m, 2H, CH_2). **^{13}C NMR (101 MHz, $CDCl_3$)**: δ = 167.4 (C_q), 162.3 (C_q), 150.8 (C_q), 142.8 (C_q), 134.6 (C_q), 130.9 (*C*H), 129.0 (*C*H), 127.2 (*C*H), 126.4 (*C*H), 123.9 (*C*H), 39.7 (*C*H), 32.6 (*C*H). **HRMS (ESI):** Calculated for $C_{17}H_{14}N_2O_2Na$ $[M+Na]^+$: 333.0846, Found: 333.0847.

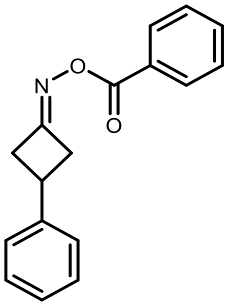

3-Phenylcyclobutan-1-one *O*-benzoyl oxime [S15]

Following **GP-I** using 3-phenylyclobut-1-one [**1a**] (7.31 mg, 50.0 µmol, 1.00 eq.) and *O*-benzoylhydroxylammonium trifluoromethanesulfonate [**S2**] (17.2 mg, 55.0 µmol, 1.00 eq.) the product (10.2 mg, 42.0 µmol, 84%) was obtained *via* flash column chromatography ((SiO_2, *n*-pentane:EtOAc 90:10, stained with $KMnO_4$) as a colorless powder.

^{1}H NMR (300 MHz, $CDCl_3$): δ = 8.12 – 8.02 (m, 2H), 7.65 – 7.54 (m, 1H), 7.47 (ddt, *J* = 8.3, 6.7, 1.1 Hz, 2H), 7.42 – 7.33 (m, 2H), 7.33 – 7.23 (m, 3H), 3.81 – 3.51 (m, 3H), 3.37 – 3.17 (m, 2H). **^{13}C NMR (101 MHz, $CDCl_3$):** δ = 166.2, 164.1, 143.2, 133.4, 129.8, 128.9, 128.7, 127.1, 126.5, 39.7, 39.6, 32.7. The spectroscopic data was in agreement to those previously reported.[19]

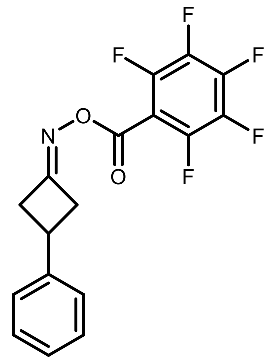

3-Phenylcyclobutan-1-one *O*-perfluorobenzoyl oxime [S16]

Following **GP-I** using 3-phenylyclobut-1-one [**1a**] (7.31 mg, 50.0 µmol, 1.00 eq.) and *O*-(perfluorobenzoyl)hydroxylammonium trifluoromethanesulfonate [**S4**] (22.6 mg, 55.0 µmol, 1.00 eq.) the product (13.9 mg, 43.0 µmol, 86%) was obtained *via* flash column chromatography (SiO_2, *n*-pentane:EtOAc 90:10, stained with $KMnO_4$) as a colorless powder.

^{1}H NMR (400 MHz, $CDCl_3$): δ = 7.42 – 7.32 (m, 2H), 7.32 – 7.23 (m, 3H), 3.71 (ddd, J = 16.2, 8.9, 7.3 Hz, 1H), 3.63 – 3.49 (m, 2H), 3.33 – 3.12 (m, 2H). **^{13}C NMR (101 MHz, $CDCl_3$):** δ = 168.2, 156.8, 145.6 (d, $^1J_{C\text{-}F}$ = 259.2 Hz), 143.6 (d, $^1J_{C\text{-}F}$ = 260.21 Hz),142.7, 137.9 (d, $^1J_{C\text{-}F}$ = 251.8 Hz), 128.9, 127.2, 126.4, 39.8, 39.4, 32.4. **^{19}F NMR** (282 MHz, $CDCl_3$): δ = -132.20 – -137.76 (m), -144.14 – -148.07 (m), -158.06 (tdd, J = 19.9, 6.0, 2.0 Hz). The spectroscopic data was in agreement to those previously reported.[20]

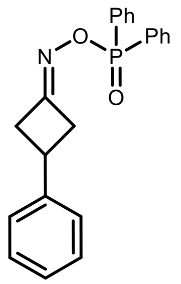

(((3-Phenylcyclobutylidene)amino)oxy)diphenylphosphine oxide [6a]

Following **GP-I** using 3-phenylyclobut-1-one [**1a**] (7.31 mg, 50.0 µmol, 1.00 eq.) and *O*-(diphenylphosphinyl)hydroxylamine [**2**] (12.8 mg, 55.0 µmol, 1.10 eq.) the product (16.0 mg, 44.3 µmol, 89%) was obtained *via* automated flash column chromatography (SiO_2, Cyclohexane:EtOAc, 100:0 → 0:100, stained with $KMnO_4$) as a colorless solid.

^{1}H NMR (400 MHz, $CDCl_3$): δ = 7.93 – 7.81 (m, 4H), 7.61 – 7.42 (m, 6H), 7.38 – 7.30 (m, 2H), 7.29 – 7.20 (m, 3H), 3.72 – 3.53 (m, 2H), 3.49 – 3.34 (m, 1H), 3.27 – 3.03 (m, 2H).**^{13}C NMR (101 MHz, $CDCl_3$)**: δ = 166.5 (d, $^3J_{C\text{-}P}$ = 12.4 Hz), 143.2, 132.5 (d, $^4J_{C\text{-}P}$ = 2.7 Hz), 132.2 (*app.* t, $^2J_{C\text{-}P}$ 10.5 Hz), 130.8 (d, $^1J_{C\text{-}P}$ = 136.1 Hz), 130.7 (d, $^1J_{C\text{-}P}$ = 135.7 Hz), 128.8 (d, $^3J_{C\text{-}P}$ = 13.3 Hz), 127.0, 126.5, 39.7, 39.5, 32.5. **^{31}P NMR (162 MHz, $CDCl_3$)**: δ = 35.2. The spectroscopic data was in agreement to those previously reported.[3]

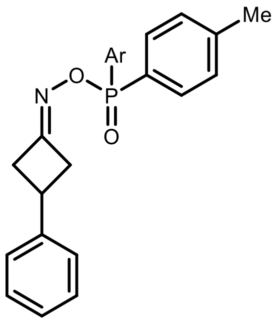

(((3-Phenylcyclobutylidene)amino)oxy)di-p-tolylphosphine oxide [S17]

Following **GP-I** using 3-phenylyclobut-1-one [**1a**] (7.31 mg, 50.0 µmol, 1.00 eq.) and *O*-(di-*p*-tolylphosphinyl)hydroxylamine [**S8**] (13.0 mg, 55.0 µmol, 1.00 eq.) the product (18.7 mg, 48.0 µmol, 96%) was obtained *via* flash column chromatography ((SiO_2, *n*-pentane:EtOAc (100:0 → 0:100)) as a colorless powder.

M.P.: 115 - 119 °C. **IR (neat)**: $\tilde{\nu}$ = 3029 (w), 1699 (m), 1603 (m), 1233 (m), 1186 (m), 1128 (s), 1110 (m), 877 (s), 808 (m), 749 (m), 700 (m), 668 (s), 530 (s), 468 (m), 443 (m), 411 (m). **^{1}H NMR (400 MHz, CDCl$_3$)**: δ = 7.78 – 7.70 (m, 4H, C$H_{arom.}$), 7.38 – 7.31 (m, 2H, C$H_{arom.}$), 7.30 – 7.20 (m, 7H, C$H_{arom.}$), 3.67 – 3.51 (m, 2H, C*H*, CH_2), 3.47 – 3.35 (m, 1H, CH_2), 3.24 – 3.02 (m, 2H, CH_2), 2.39 (s, 6H, CH_3). **^{13}C NMR (101 MHz, CDCl$_3$)**: δ = 166.1 (d, $^3J_{C-P}$ = 12.4 Hz, C_q), 143.3 (C_q), 142.9 (d, $^4J_{C-P}$ = 2.7 Hz, C_q), 132.2 (*app.* t, $^2J_{C-P}$ 10.7 Hz, CH$_{arom.}$), 129.4 (d, $^3J_{C-P}$ = 13.3 Hz, CH$_{arom.}$), 128.8 (CH$_{arom.}$), 127.8 (d, $^1J_{C-P}$ = 138.6 Hz, C_q), 127.7 (d, $^1J_{C-P}$ = 138.3 Hz, C_q), 126.9 (CH$_{arom.}$), 126.5 (CH$_{arom.}$), 39.8 (CH$_2$), 39.5 (CH$_2$), 32.5 (CH), 21.8 (CH$_3$). **^{31}P NMR (162 MHz, CDCl$_3$)**: δ = 36.29. **HRMS (ESI):** Calculated for $C_{24}H_{25}NO_2P$ [M+H]$^+$: 390.1617, Found: 390.1613.

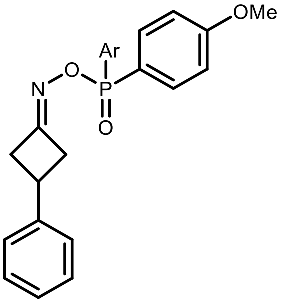

Bis(4-methoxyphenyl)(((3-phenylcyclobutylidene)amino)oxy)phosphine oxide [S18]

Following **GP-I** using 3-phenylyclobut-1-one [**1a**] (7.31 mg, 50.0 µmol, 1.00 eq.) and *O*-(bis-*p*-methoxyphenylphosphinyl)hydroxylamine [**S12**] (16.0 mg, 55.0 µmol, 1.00 eq.) the product (14.7 mg, 38.5 µmol, 77%) was obtained *via* automated flash column chromatography ((SiO_2, cyclohexane:EtOAc (100:0 → 0:100)) as a colorless powder.

M.P.: 117 - 122 °C. **IR (neat)**: $\tilde{\nu}$ = 2925 (w), 1597 (s), 1571 (w), 1504 (m), 1457 (w), 1296 (m), 1253 (s), 1227 (s), 1179 (m), 1127 (s), 1025 (m), 871 (s), 831 (m), 805 (m), 749 (m), 699 (m), 672 (m), 543 (s), 457 (m). **^{1}H NMR (400 MHz, $CDCl_3$)**: δ = 7.78 (ddd, J = 11.9, 8.7, 3.4 Hz, 4H, $CH_{arom.}$), 7.38 – 7.31 (m, 2H, $CH_{arom.}$), 7.26 – 7.21 (m, 3H, $CH_{arom.}$), 6.99 – 6.93 (m, 4H, $CH_{arom.}$), 3.83 (s, 6H, CH_3), 3.65 – 3.54 (m, 2H, C*H*, CH_2), 3.41 (ddt, J = 16.9, 8.9, 3.2 Hz, 1H, CH_2), 3.20 – 3.12 (m, 1H, CH_2), 3.12 – 3.06 (m, 1H, CH_2). **^{13}C NMR (151 MHz, $CDCl_3$)**: δ = 166.0 (d, 12.4 Hz, $^3J_{C-P}$ = 12.4 Hz, C_q), 162.8 (d, $^4J_{C-P}$ = 3.0 Hz, C_q), 143.3 (C_q), 134.1 (d, $^2J_{C-P}$ 11.3 Hz, *C*H), 134.0 (d, $^2J_{C-P}$ 11.4 Hz, *C*H), 128.8 (*C*H), 126.9 (*C*H), 126.5 (*C*H), 122.2 (d, $^1J_{C-P}$ = 144.0 Hz, C_q), 122.1 (d, $^1J_{C-P}$ = 143.5 Hz, C_q), 114.2 (d, $^3J_{C-P}$ = 14.3 Hz, *C*H), 114.2 (d, $^3J_{C-P}$ = 14.3 Hz, *C*H), 55.5 (CH_3), 39.8 (CH_2), 39.5 (CH_2), 32.4 (*C*H). **^{31}P NMR (162 MHz, $CDCl_3$)**: δ = 36.26.

4. DFT calculations for the asymmetric condensation

Methods

All computations were performed with the TURBOMOLE program.[21] The structures were optimized without any geometry constraints using the TPSS meta-GGA functional[22] and an atom-pairwise dispersion correction (D3).[23] A flexible triple zeta basis set (def2-TZVP)[24] and DFT integration grid m5 was used in all calculations. For the calculation of free energy contributions of translation, rotations and harmonic vibrations (G^{RRHO}, computed with TPSS-D3/def2-TZVP), a rotor approximation was applied for vibrational modes with wave numbers below 100 cm^{-1}.[25] Transition structures were identified by one imaginary harmonic vibrational frequency. The nature of transition structures **TS1[a-d]** and **TS2[a-d]** was tested on some representative examples by IRC pathway optimizations.

Single point energy calculations were performed with the hybrid functional PW6B95(-D3).[26] Free energies of solvation (G^{solv}) were obtained with the COSMO-RS model using toluene ($CH_3C_6H_5$) as solvent.[27]

Results

We have optimized transition structures and intermediates for four stereochemically distinct pathways: the C-N bond of hemiaminal **INT1** can be formed by attack from the two diastereotopic faces of the C=O group, with the catalyst *ent*-**7e** bound from two different sides of the N-C-O plane (*via* **TS1[a-d]**). The four intermediates **INT1[a-d]1** have to undergo endothermic proton shift to form **INT1[a-d]2** which is the precursor of water elimination through **TS2[a-d]**. We have optimized several conformers of the second transition structure obtained by rotation of the O-P single bond. We report only the lowest conformer of each pathway here.

In the following a detailed overview of the computational results is provided. The structures shown in **Scheme 2C** in the manuscript translate as follows to **Figure S1** provided in the SI:

Scheme 2C	Figure S1
TS1	**TS1c**
TS1’	**TS1a**
INT1	**INT1d1**
INT1’	**INT1b1**
TS2	**TS2d**
TS2’	**TS2b**

To keep the discussion of the DFT assessment in the paper as simple as possible, the following simplifications were made:

1. In **Scheme 2C** only the energetically most favorable pathways leading to the two different enantiomers are depicted. The complete computational picture is provided below.

2. **TS1a** and **TS1b**, as well as **TS1c** and **TS1d** can be interconverted by rotation of the chiral phosphoric acid. Due to the low barrier for this interconversion by hydrogen bond rearrangement, the more favorable transition states **TS1a** (**TS1'**) and **TS1c** (**TS1**) were chosen for representation in **Scheme 2C**. The same holds true for the intermediates **Int1a1** and **Int1b1**, as well as **Int 1c1** and **Int1d1**. Therefore, the more stable intermediates **Int1b1** (**Int1'**) and **Int1d1** (**Int1**) are shown in **Scheme 2C**.
3. The calculations were performed using the (*S*)-enantiomer of the chiral phosphoric acid (*ent*-**7e**). The optimization and scope of the study were conducted with the (*R*)-enantiomer of the chiral phosphoric acid (**7e**), leading to the opposite enantiomer. To match the absolute configuration compared to those depicted throughout the manuscript, the depiction in **Scheme 2C** has been adjusted based on equal energies for enantiomeric transition states. Therefore, the stereoconfiguration for the transition states, intermediates and products in **Scheme 2C** are interconverted compared to **Figure S1**.

Scheme S1 presents all computed intermediates together with the calculated free energies, related to the isolated reactants and the catalyst, depicted in Scheme S1. All species and the contributions to their free energies are compiled in Table S5.

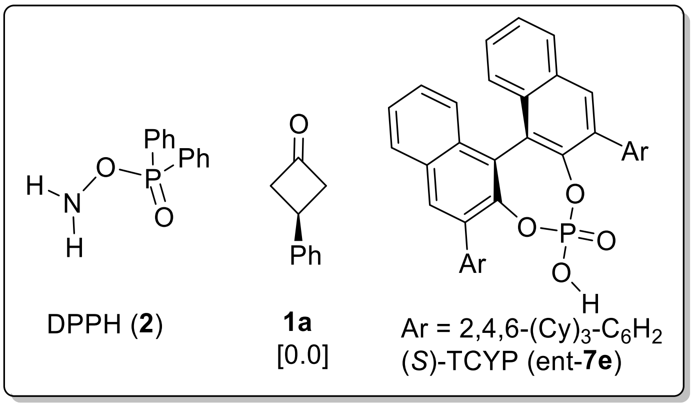

Scheme S1

Figures S1 and **S2** depict the transition structures **TS1[a-d]** and **TS2[a-d]**. As can be seen from the free energies, the hemiaminal **INT1** should be easily formed without any *syn*/*anti*-preference (**TS1b** *vs.* **TS1c,** $\Delta\Delta G^{\ddagger}$ = 0 kcal/mol). We assume that a rearrangement of hydrogen bonds would allow an interconversion of **INT1a1** with **INT1b1** and **INT1c1** with **INT1d1**. The selectivity in the water elimination step is then determined by **TS2b** *vs.* **TS2d** ($\Delta\Delta G^{\ddagger}$ = 2.4 kcal/mol) in qualitatively correct agreement with the experimentally observed product ratio.

Figure S1   DFT-calculated reaction pathways.

Path (a)

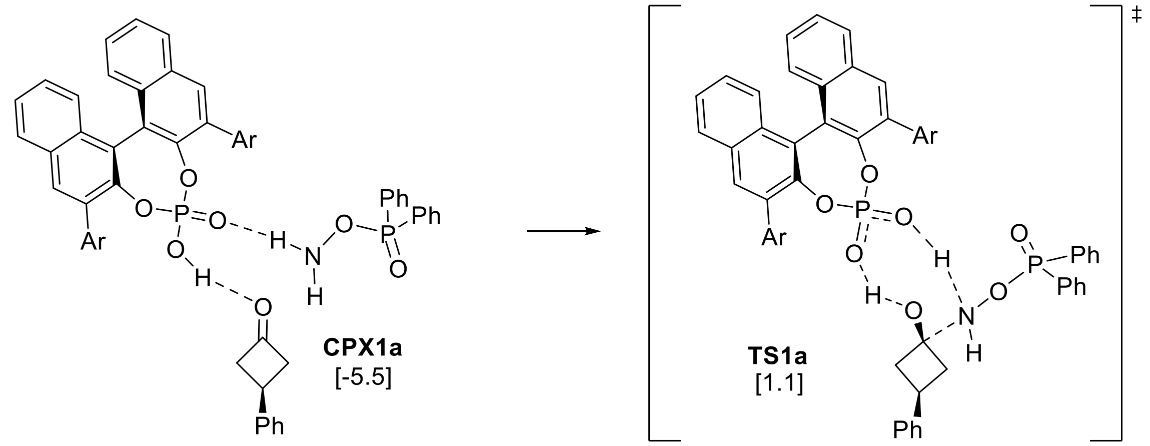

Path (b)

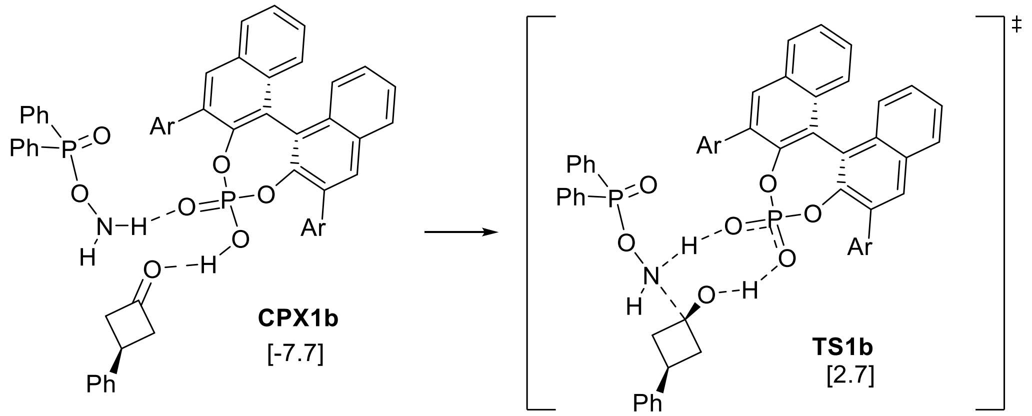

Path (c)

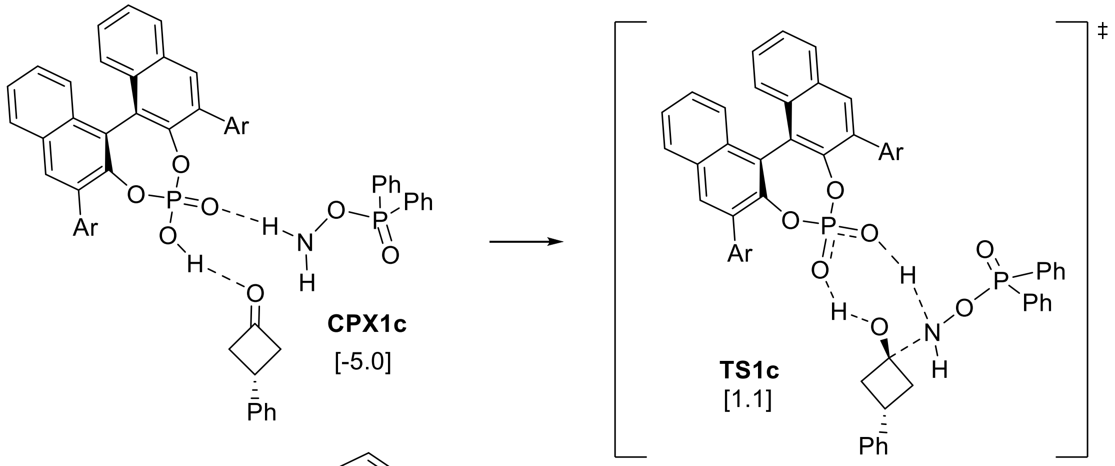

Path (d)

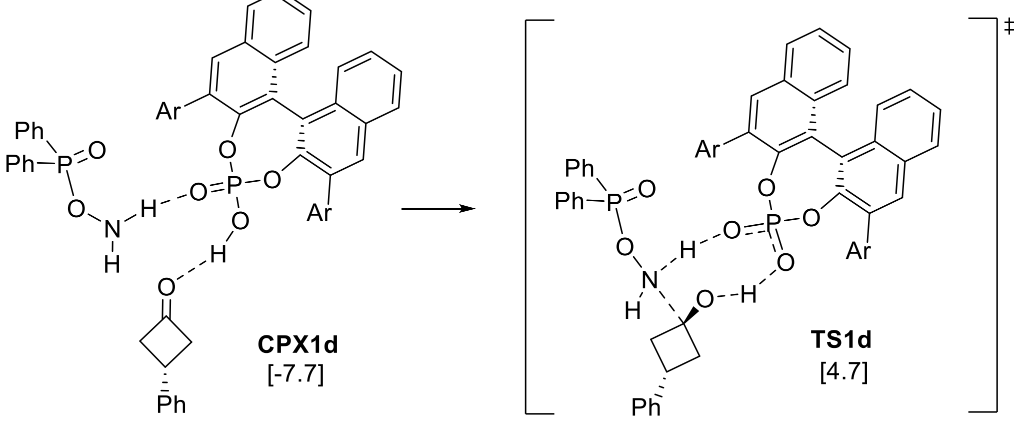

Figure S1 (continued) DFT-calculated reaction pathways.

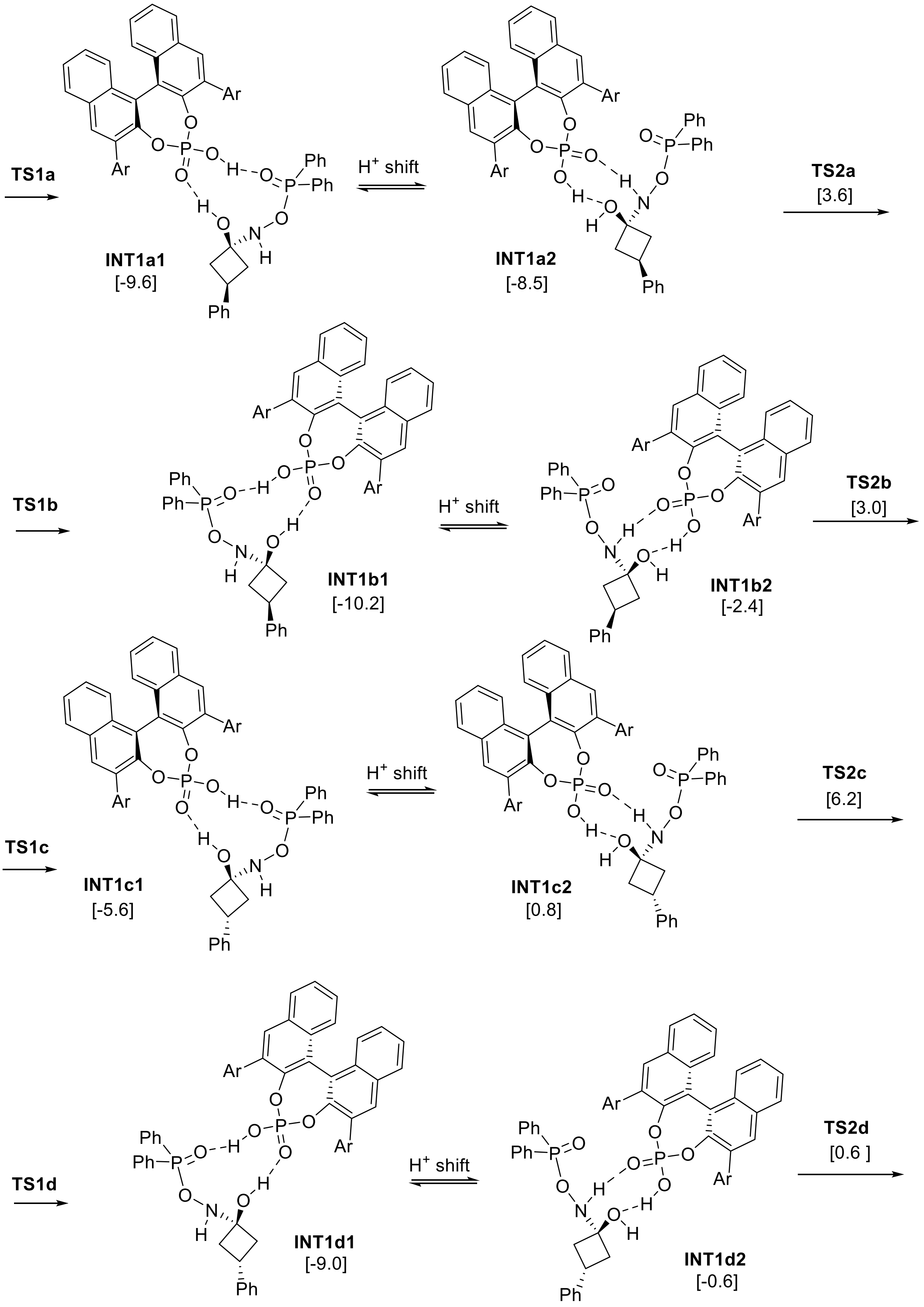

Figure S1 (continued) DFT-calculated reaction pathways.

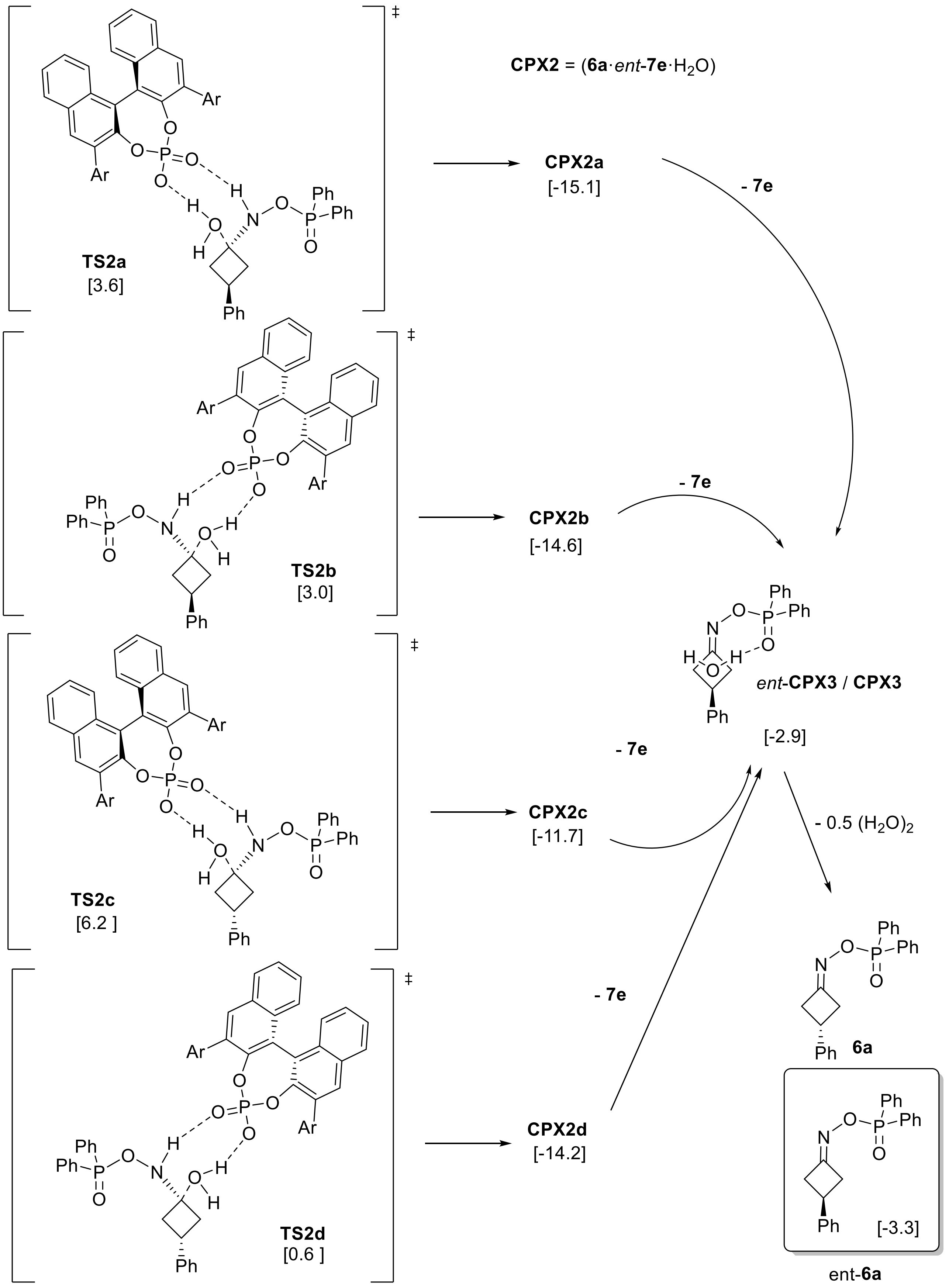

Table S5: Relative energies of molecular species in the reactions, as calculated with DFT[a] for T = 298.15 K. $\Delta G(298)_{solv} = \Delta E(PW6B95\text{-}D3//TPSS\text{-}D3) + \Delta G^{RRHO}_{298} + \Delta G^{solv}_{298}$

Species	E(TPSS-D3) [E_h]	G^{RRHO}_{298} [kcal/mol]	E(PW6B95-D3) [E_h]	G^{solv}_{298} (CH_3Ph) [kcal/mol]	**$\Delta G(298)_{solv}$ (CH_3Ph)[b] [kcal/mol]**
**1a**	-462.557580	84.309	-463.053863	-5.864	
TPPH (**2**)	-1011.511860	108.851	-1012.485558	-11.972	
CPA (**7e**)	-3284.382239	774.739	-3287.817412	-34.958	
**Ref**	**-4758.451680**	**967.899**	**-4763.356833**	**-52.794**	**0.0**
**6a**	-1397.608572	193.329	-1399.003584	-18.680	
H2O	-76.464555	1.975	-76.540530	1.507	
H2O dimer	-152.937544	11.152	-153.089184	-5.413	
**CPX1a**	-4758.524297	1004.268	-4763.425901	-50.841	-5.0
**CPX1b**	-4758.521383	1003.371	-4763.424248	-51.427	-5.5
**CPX1c**	-4758.523749	1004.169	-4763.427952	-52.172	-7.7
**CPX1d**	-4758.521803	1003.275	-4763.424454	-53.407	-7.7
**TS1a**	-4758.517037	1003.129	-4763.412486	-51.977	1.1
**TS1b**	-4758.513179	1003.637	-4763.410058	-52.394	2.7
**TS1c**	-4758.521468	1004.326	-4763.416230	-50.801	1.1
**TS1d**	-4758.509027	1002.448	-4763.406085	-51.781	4.7
**INT1a1**	-4758.535425	1007.090	-4763.439907	-49.436	-9.6
**INT1b1**	-4758.535761	1007.243	-4763.439273	-50.577	-10.2
**INT1c1**	-4758.530573	1006.924	-4763.434482	-48.663	-5.6
**INT1d1**	-4758.531433	1007.004	-4763.436395	-50.957	-9.0
**INT1a2**	-4758.530862	1006.864	-4763.432889	-52.492	-8.5
**INT1b2**	-4758.520736	1005.949	-4763.423528	-51.385	-2.4
**INT1c2**	-4758.517911	1006.765	-4763.422233	-49.793	0.8
**INT1d2**	-4758.517922	1005.767	-4763.420582	-51.300	-0.6
**TS2a**	-4758.515010	1004.492	-4763.411241	-51.660	3.6
**TS2b**	-4758.518188	1006.119	-4763.415044	-51.455	3.0
**TS2c**	-4758.518327	1005.321	-4763.411677	-49.570	6.2
**TS2d**	-4758.519051	1004.613	-4763.415183	-52.274	0.6
**CPX2a**	-4758.534504	1002.785	-4763.436968	-52.495	-15.1
**CPX2b**	-4758.532272	1003.031	-4763.437237	-52.055	-14.6
**CPX2c**	-4758.529429	1002.451	-4763.431886	-51.915	-11.7
**CPX2d**	-4758.531185	1003.670	-4763.437504	-52.188	-14.2
**CPX3**	-1474.094956	207.232	-1475.565491	-18.448	-2.9
**6a**	-1397.608572	193.329	-1399.003584	-18.680	-0.1 / -3.3[c]
H2O	-76.464555	1.975	-76.540530	1.507	
H2O dimer	-152.937544	11.152	-153.089184	-5.413	

[a] all calculations were performed with the def2-TZVP basis set
[b] relative free energies refer to isolated reactants (REF = **1a** + **2** + **7e**)
[c] with product water treated as dimer (0.5 eq. H_2O)

Figure S2 DFT-optimized (TPSS-D3/def2-TZVP) transition structures of the addition of DPPH to the carbonyl bond of 1a. Bond distances in Å. Element colors: gray (C), red (O), blue (N), white (H), yellow (P).

[Relative free energies $\Delta G(298)_{solv}$ in kcal/mol]

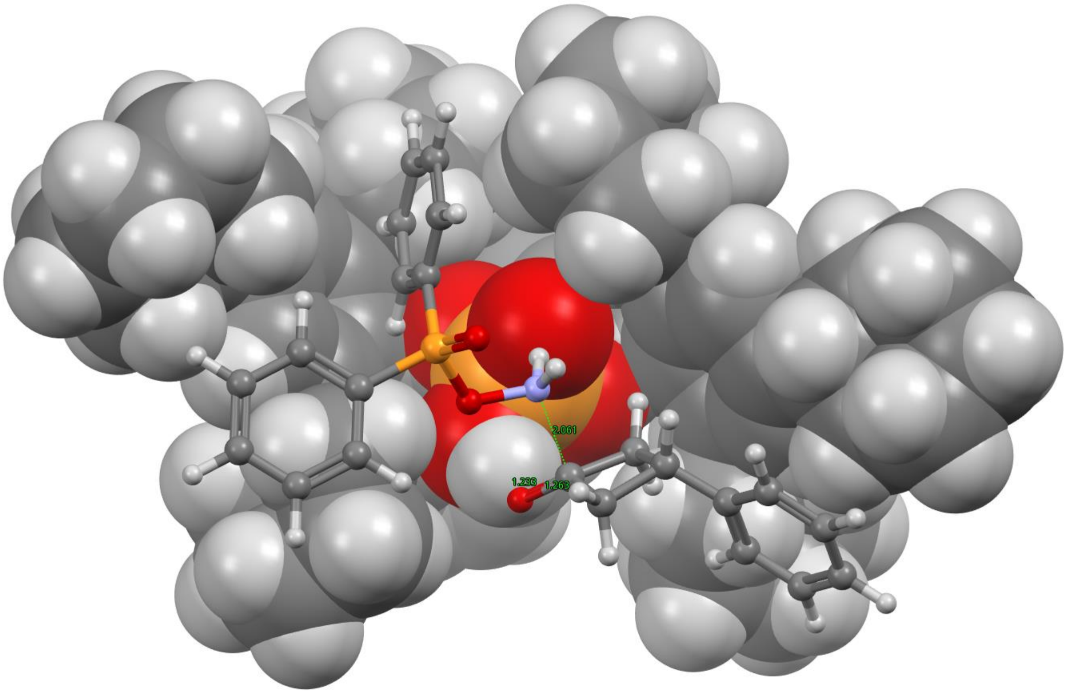

TS1a [+1.1]

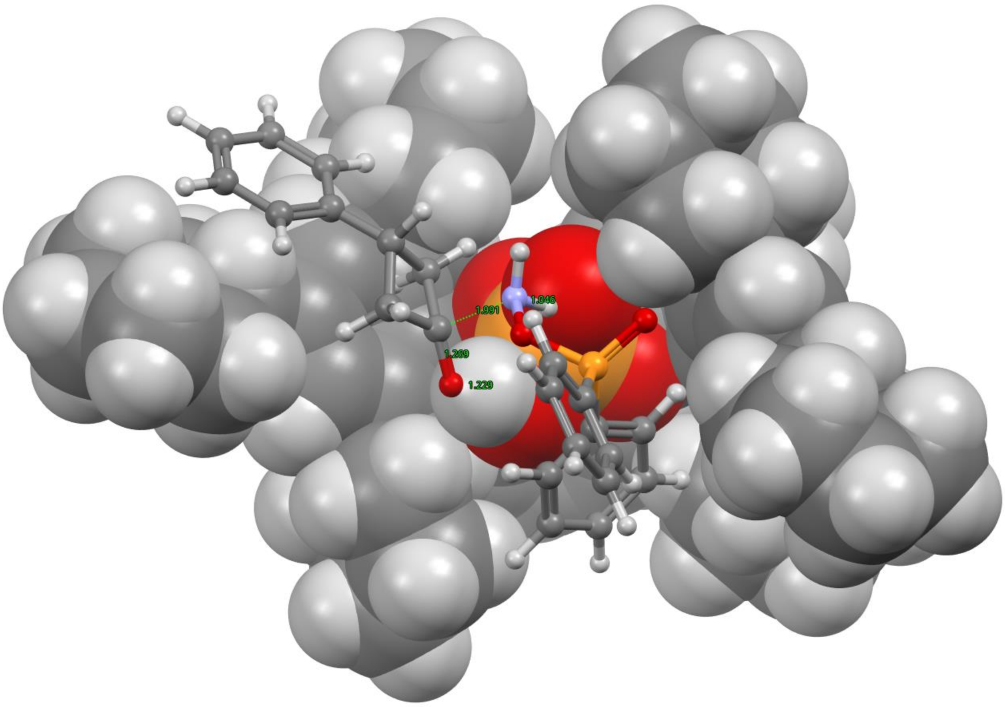

TS1b [+2.7]

Figure S2 (continued): DFT-optimized (TPSS-D3/def2-TZVP) transition structures of the addition of DPPH to the carbonyl bond of 1a. Bond distances in Å. Element colors: gray (C), red (O), blue (N), white (H) , yellow (P). [Relative free energies ΔG(298)$_{solv}$ in kcal/mol]

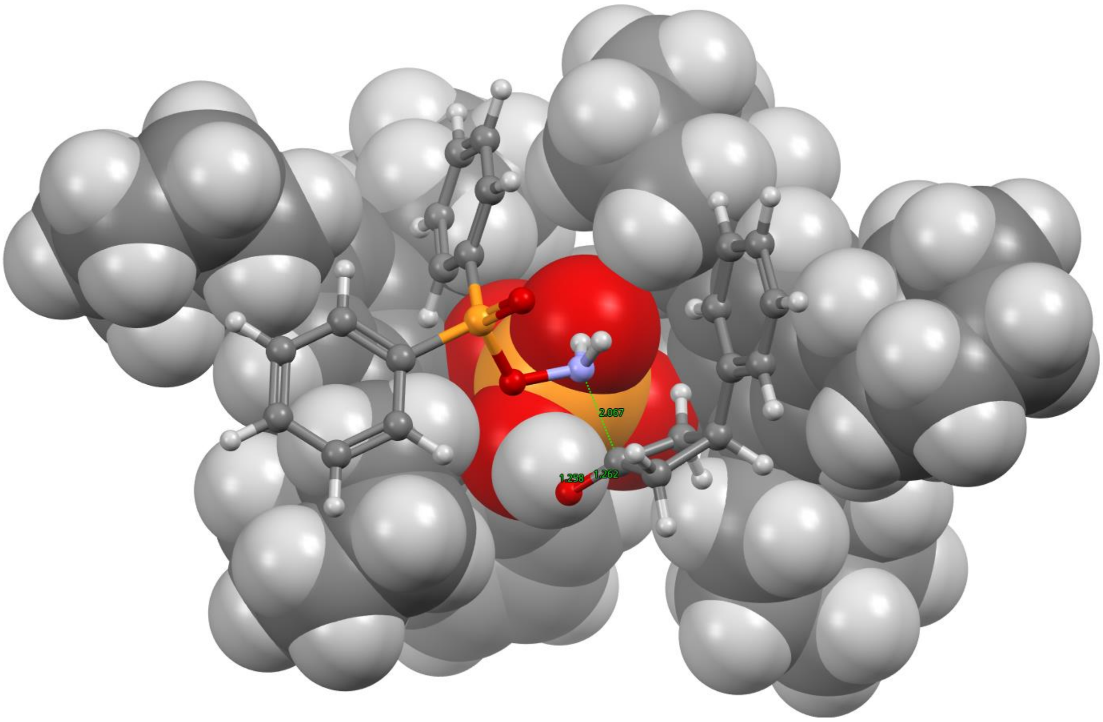

TS1c [+1.1]

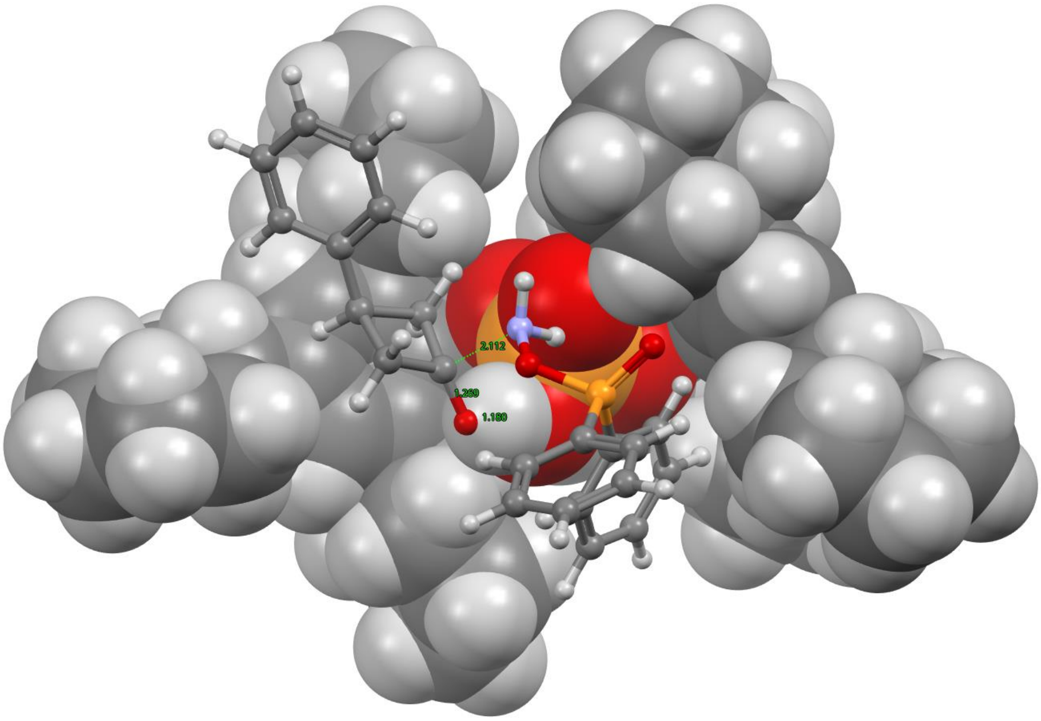

TS1d [+4.7]

Figure S3: DFT-optimized (TPSS-D3/def2-TZVP) transition structures of the elimination of water from hemiaminal INT1. Bond distances in Å. Element colors: gray (C), red (O), blue (N), white (H), yellow (P). [Relative free energies $\Delta G(298)_{solv}$ in kcal/mol]

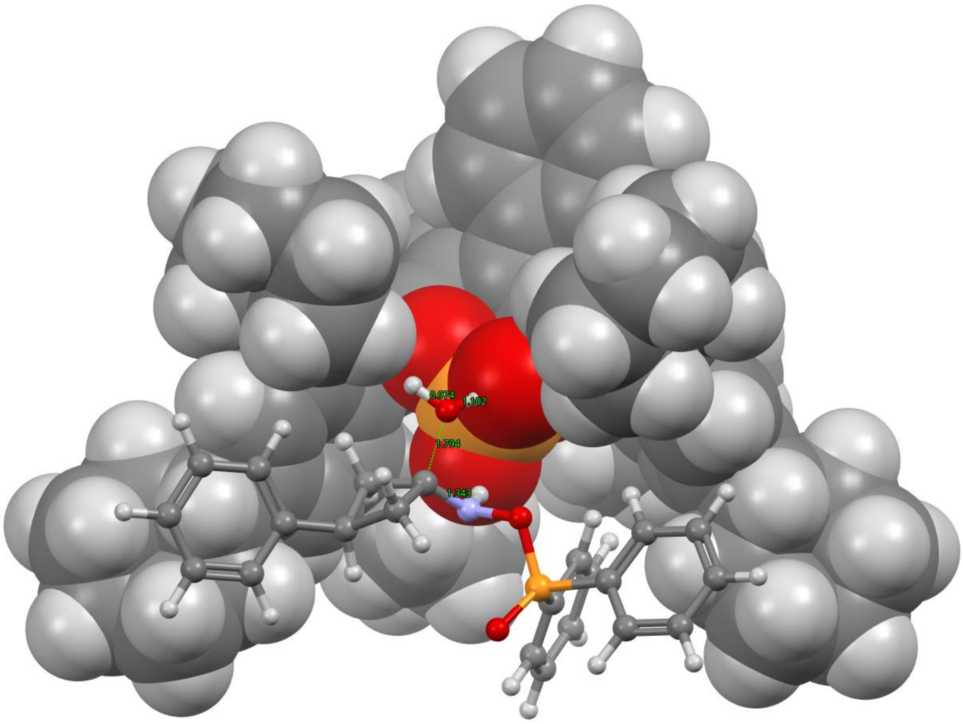

TS2a [+3.6]

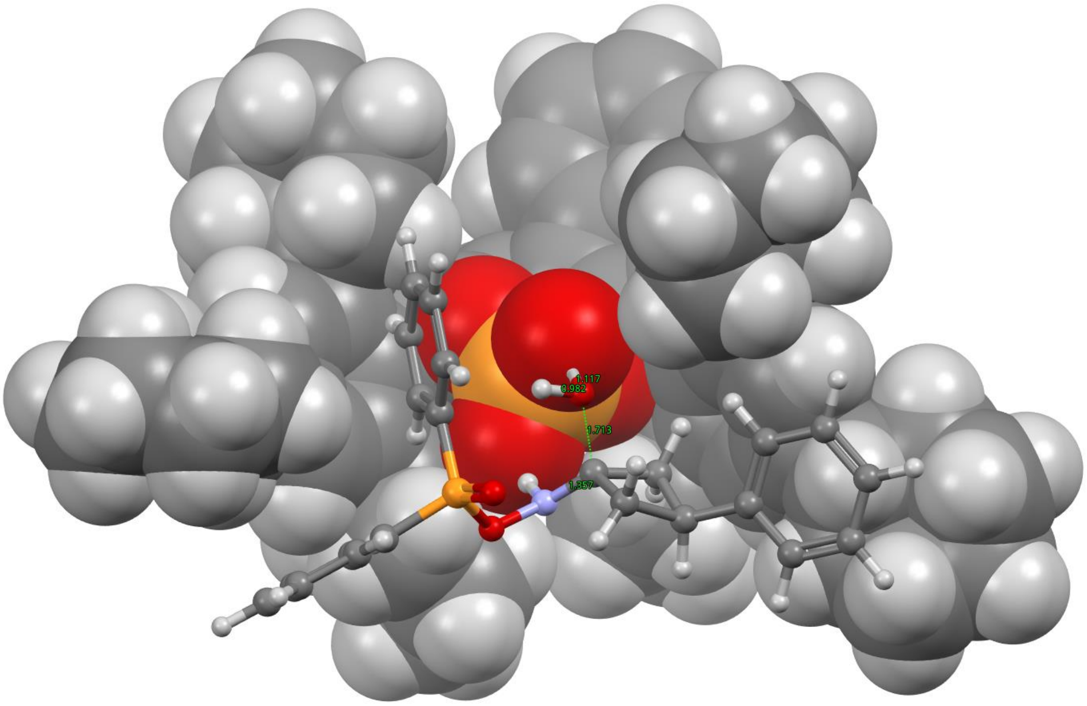

TS2b [+3.0]

Figure S3 (continued) DFT-optimized (TPSS-D3/def2-TZVP) transition structures of the elimination of water from hemiaminal INT1. Bond distances in Å. Element colors: gray (C), red (O), blue (N), white (H), yellow (P). [Relative free energies $\Delta G(298)_{solv}$ in kcal/mol]

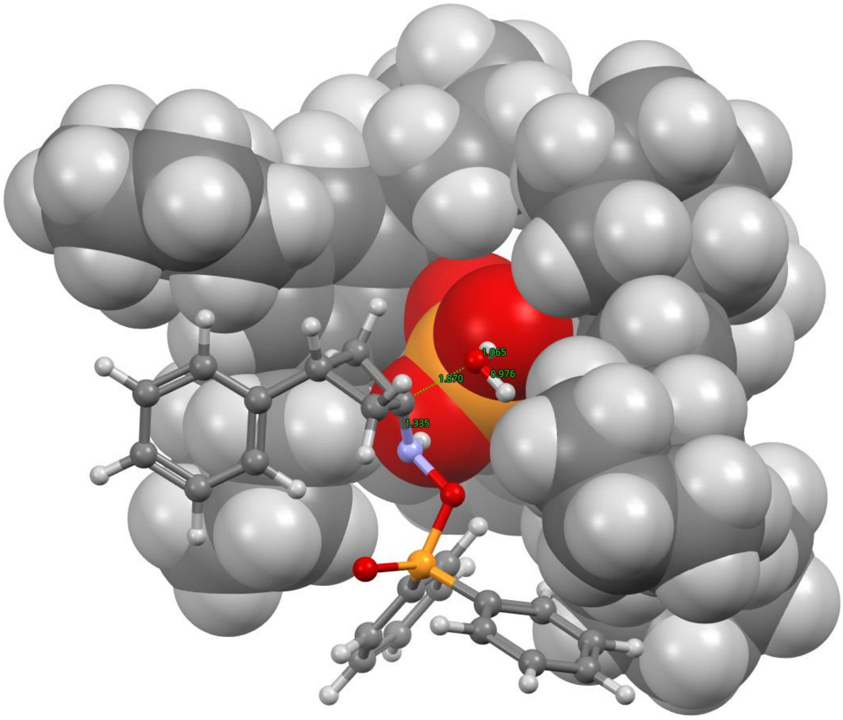

TS2c [+6.2]

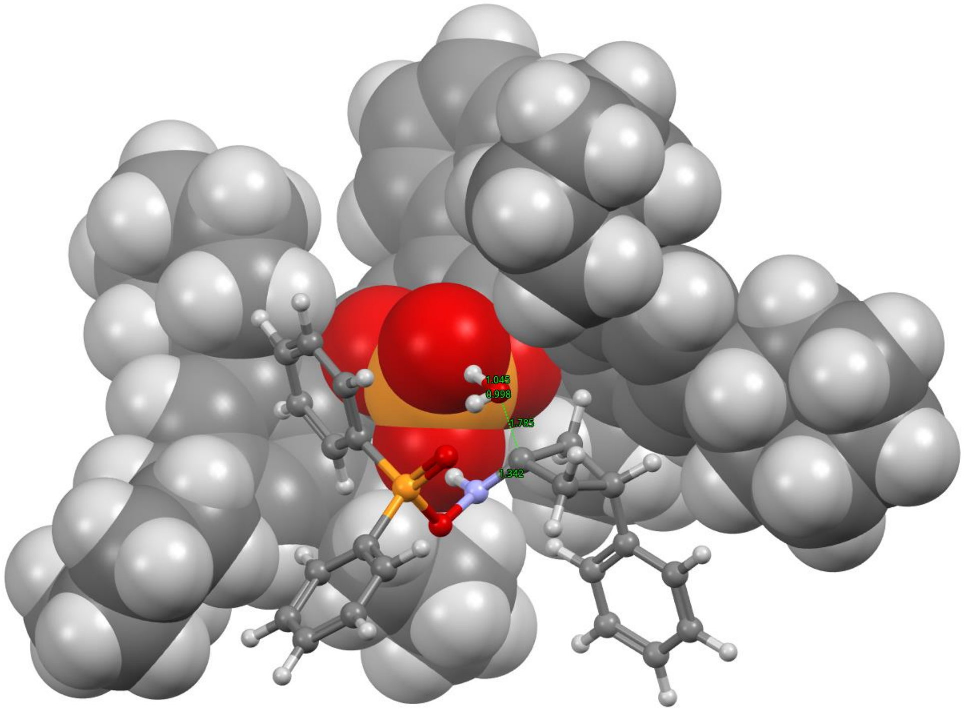

TS2d [+0.6]

5. Substrate scope cyclobutanone oxime esters

The racemic products were prepared according to the following procedure:

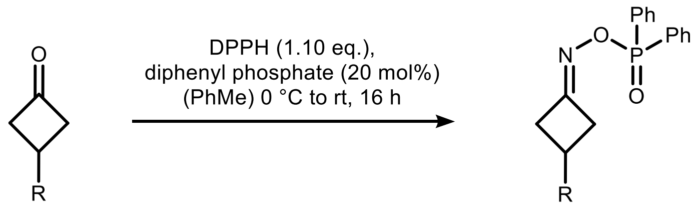

In a round bottom flask, diphenyl phosphate (20 mol%) and cyclobutanone (200 µmol, 1.00 eq.) were dissolved in toluene (0.05 M). The mixture was cooled to 0 °C. *O*-Diphenylphosphinylhydroxylamine (220 µmol, 1.10 eq.) was added and the reaction mixture was allowed to warm to room temperature over night. NEt_3 (200 µmol, 1.00 eq.) was added. The solvent was removed under reduced pressure and the crude product was purified *via* automated flash column chromatography with the conditions given in the corresponding entry.

General procedure J (GP-J) for the asymmetric condensation with (*R*)-TCYP:

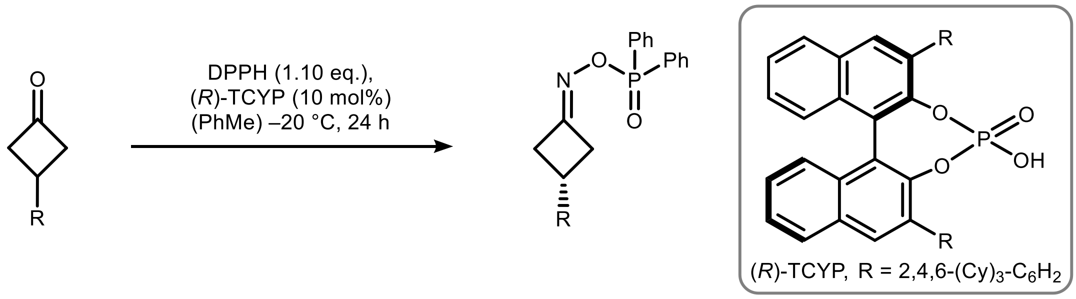

In a round bottom flask, (*R*)-TCYP (10 mol%) and cyclobutanone (200 µmol, 1.00 eq.) were dissolved in toluene (0.05 M). The mixture was cooled to –20 °C. *O*-Diphenylphosphinylhydroxylamine (220 µmol, 1.10 eq.) was added and the reaction mixture was stirred at –20 °C for 24 h. NEt_3 (200 µmol, 1.00 eq.) was added. The solvent was removed under reduced pressure and the crude product was purified *via* automated flash column chromatography with the conditions given in the corresponding entry.

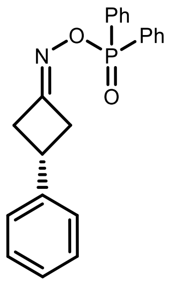

(*R*)-(((3-Phenylcyclobutylidene)amino)oxy)diphenylphosphine oxide [6a]

Following **GP-J** using 3-phenylyclobut-1-one [**1a**] (29.2 mg, 200 µmol, 1.00 eq.), (*R*)-TCYP [**7e**] (19.8 mg, 20.0 µmol, 10 mol%) and *O*-(diphenylphosphinyl)hydroxylamine [**2**] (51.3 mg, 220 µmol, 1.10 eq.) the product (64.0 mg, 177 µmol, 89%) was obtained

via automated flash column chromatography (SiO_2, Cyclohexane:EtOAc, 100:0 → 0:100, stained with $KMnO_4$) as colorless solid.

^{1}H NMR (400 MHz, $CDCl_3$): δ = 7.93 – 7.81 (m, 4H), 7.61 – 7.42 (m, 6H), 7.38 – 7.30 (m, 2H), 7.29 – 7.20 (m, 3H), 3.72 – 3.53 (m, 2H), 3.49 – 3.34 (m, 1H), 3.27 – 3.03 (m, 2H). **^{13}C NMR (101 MHz, $CDCl_3$)**: δ = 166.5 (d, $^3J_{C-P}$ = 12.4 Hz), 143.2, 132.5 (d, $^4J_{C-P}$ = 2.7 Hz), 132.2 (*app.* t, $^2J_{C-P}$ 10.5 Hz), 130.8 (d, $^1J_{C-P}$ = 136.1 Hz), 130.7 (d, $^1J_{C-P}$ = 135.7 Hz), 128.8 (d, $^3J_{C-P}$ = 13.3 Hz), 127.0, 126.5, 39.7, 39.5, 32.5. **^{31}P NMR (162 MHz, $CDCl_3$)**: δ = 35.23. The spectroscopic data was in agreement to those previously reported.[3] **Optical Rotation:** $[\alpha]_D^{25}$ = –65.1 (c = 0.5, $CHCl_3$) for an enantiomerically enriched sample of 95:05 er. The enantiomeric purity was established by HPLC analysis using a chiral column (Cellulose-1, 22 °C, 1 mL/min, 85:15 *n*-hexane:isopropanol, 210 nm, t = 15.855 min and 18.175 min).

1.0 mmol scale:

Following **GP-J** using 3-phenylyclobut-1-one [**1a**] (146 mg, 1.00 mmol, 1.00 eq.), (*R*)-TCYP [**7e**] (101 mg, 100 µmol, 10 mol%) and *O*-(diphenylphosphinyl)hydroxylamine [**2**] (257 mg, 1.10 mmol, 1.10 eq.) the product (330 mg, 913 µmol, 91%) was obtained *via* automated flash column chromatography (SiO_2, Cyclohexane:EtOAc, 100:0 → 0:100, stained with $KMnO_4$) as colorless solid. **Optical Rotation:** $[\alpha]_D^{25}$ = –61.0 (c = 1.0, $CHCl_3$) for an enantiomerically enriched sample of 94:06 er. The enantiomeric purity was established by HPLC analysis using a chiral column (Cellulose-1, 40 °C, 1 mL/min, 85:15 *n*hexane:isopropanol, 214 nm, t = 14.009 min and 15.642 min).

Reisolation of the catalyst [**7e**]*:*

Following a procedure from Klussmann *et al.*[28], the catalyst isolated by flash column chromatography was dissolved in CH_2Cl_2 (20 mL). The organic phase was washed with 1.0 M HCl (3 x 10 mL), dried over Mg_2SO_4 and concentrated under reduced pressure. (R)-TCYP [*ent*-**7e**] (93 mg, 93.6 µmol, 91%) was obtained after recrystallization from acetonitrile as a colorless solid.

^{1}H NMR (400 MHz, $CDCl_3$): δ = 7.84 (d, *J* = 8.2 Hz, 2H), 7.70 (s, 2H), 7.44 (ddd, *J* = 8.1, 6.4, 1.5 Hz, 2H), 7.31 – 7.17 (m, 4H), 6.93 – 6.86 (m, 4H), 2.51 – 2.35 (m, 2H), 2.24 – 2.01 (m, 4H), 1.97 – 0.40 (m, 60H). **^{13}C NMR (101 MHz, $CDCl_3$)**: δ = 147.0, 146.6, 146.5, 146.4, 146.3, 132.3, 132.2, 131.9, 131.8, 131.0, 128.2, 126.8, 126.2, 125.6, 122.4, 121.8, 121.6, 44.9, 42.3, 41.9, 37.1, 35.2, 34.8, 34.3, 33.3, 32.7, 27.5, 27.3, 27.3, 27.10, 26.8, 26.5, 26.4, 25.9. **^{31}P NMR (162 MHz, $CDCl_3$)**: δ = 1.27. The spectroscopic data was in agreement to those previously reported.[29]

Reuse of the catalyst:

Following **GP-J** using 3-phenylyclobut-1-one [**1a**] (29.2 mg, 200 µmol, 1.00 eq.), (*R*)-TCYP [**7e**] (19.8 mg, 20.0 µmol, 10 mol%) and *O*-(diphenylphosphinyl)hydroxylamine [**2**] (51.3 mg, 220 µmol, 1.10 eq.) the product (63.8 mg, 175 µmol, 88%) was obtained by flash column chromatography (SiO_2, Cyclohexane:EtOAc, 100:0 → 0:100, stained with $KMnO_4$) as colorless solid. **Optical Rotation:** $[\alpha]_D^{25}$ = –59.7 (c = 1.0, $CHCl_3$) for an enantiomerically enriched sample of 94:06 er. The enantiomeric purity was

established by HPLC analysis using a chiral column (Cellulose-1, 40 °C, 1 mL/min, 85:15 *n*hexane:isopropanol, 214 nm, t = 14.012 min and 15.615 min).

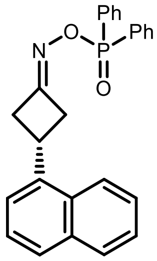

(*R*)-(((3-(Naphthalen-1-yl)cyclobutylidene)amino)oxy)diphenylphosphine oxide [6b]

Following **GP-J** using 3-(naphthalen-2-yl)cyclobutan-1-one [**1b**] (39.3 mg, 200 µmol, 1.00 eq.), (*R*)-TCYP [**7e**] (19.8 mg, 20.0 µmol, 10 mol%) and *O*-(diphenylphosphinyl)hydroxylamine [**2**] (51.3 mg, 220 µmol, 1.10 eq.) the product (68.8 mg, 167 µmol, 84%) was obtained *via* automated flash column chromatography (SiO_2, Cyclohexane:EtOAc, 100:0 → 0:100, stained with $KMnO_4$) as colorless solid.

M.P.: 121 – 125 °C. **IR (neat)**: $\tilde{\nu}$ = 3053 (w), 2975 (w), 2926 (w), 2234 (w), 1693 (w), 1633 (w), 1592 (w), 1508 (w), 1485 (w), 1439 (m), 1397 (w), 1231 (s), 1180 (w), 1129 (m), 1112 (w), 1071 (w), 1028 (w), 877 (s), 816 (m), 729 (s), 696 (s), 651 (w), 602 (w), 553 (s), 531 (m), 479 (w), 448 (w), 427 (w), 417 (w). **^{1}H NMR (400 MHz, $CDCl_3$)**: δ = 7.96 – 7.86 (m, 4H, C$H_{arom.}$), 7.86 – 7.76 (m, 3H, C$H_{arom.}$), 7.67 (s, 1H, C$H_{arom.}$), 7.59 – 7.52 (m, 2H, C$H_{arom.}$), 7.52 – 7.43 (m, J = 10.9, 4.3 Hz, 6H, C$H_{arom.}$), 7.37 (dd, J = 8.5, 1.8 Hz, 1H, C$H_{arom.}$), 3.88 – 3.75 (m, 1H, C*H*), 3.68 (*app.* ddt, J ≈ 17.4, 9.1, 3.4 Hz, 1H, C$H_{2.}$), 3.49 (*app.* ddt, J ≈ 16.0, 8.9, 3.3 Hz, 1H, CH_2), 3.35 – 3.14 (m, 2H, CH_2).. **^{13}C NMR (101 MHz, $CDCl_3$)**: δ = 166.4 (d, $^{3}J_{C-P}$ = 12.4 Hz, *C*=N), 140.5 (C_q), 133.4 (C_q), 132.5 (d, $^{4}J_{C-P}$ = 2.6 Hz, *C*H)[b], 132.2 (t, $^{2}J_{C-P}$ = 10.4 Hz, *C*H)[c], 130.8 (d, $^{1}J_{C-P}$ = 136.1 Hz, C_q), 130.7 (d, $^{1}J_{C-P}$ = 135.7 Hz, C_q), 128.8 (*C*H), 128.7 (d, $^{3}J_{C-P}$ = 13.2 Hz, *C*H), 127.8 (*C*H), 127.8 (*C*H), 126.5 (*C*H), 126.0 (*C*H), 124.9 (*C*H), 124.8 (*C*H), 39.6 (*C*H), 39.4 (CH_2), 32.64 (CH_2). **^{31}P NMR (162 MHz, $CDCl_3$)**: δ = 35.30. **HRMS (ESI):** Calculated for $C_{26}H_{22}NO_2P$ $[M+H]^+$: 412.1466, Found: 412.1462. **Optical Rotation:** $[\alpha]_D^{25}$ = –81.4 (c = 0.5, $CHCl_3$) for an enantiomerically enriched sample of 94:06 er. The enantiomeric purity was established by HPLC analysis using a chiral column (Lux® Cellulose-1, 22 °C, 1 mL/min, 85:15 *n*hexane:isopropanol, 210 nm, t = 37.277 min and 43.452 min).

---

[b] Overlapp of 2 carbon signals (d → CH and s → Cq).

[c] Appears as triplett due to overlap of 2 carbon signals (dublett)

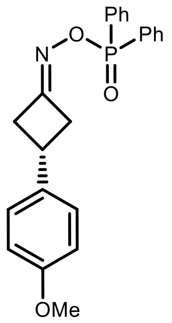

(*R*)-(((3-(4-Methoxyphenyl)cyclobutylidene)amino)oxy)diphenylphosphine oxide [6c]

Following **GP-J** using 3-(4-methoxyphenyl)cyclobutan-1-one [**1c**] (35.2 mg, 200 µmol, 1.00 eq.), (*R*)-TCYP [**7e**] (19.8 mg, 20.0 µmol, 10 mol%) and *O*-(diphenylphosphinyl)hydroxylamine [**2**] (51.3 mg, 220 µmol, 1.10 eq.) the product (65.2 mg, 167 µmol, 83%) was obtained *via* automated flash column chromatography (SiO_2, Cyclohexane:EtOAc, 100:0 → 0:100, stained with $KMnO_4$) as colorless solid.

M.P.: 89 – 90 °C. **IR (neat)**: $\tilde{\nu}$ = 2956 (w), 2922 (w), 1688 (w), 1612 (w), 1592 (w), 1514 (m), 1488 (w), 1460 (w), 1439 (m), 1396 (w), 1247 (s), 1180 (m), 1127 (s), 1096 (m), 1070 (m), 1037 (m), 878 (s), 827 (m), 727 (s), 695 (s), 625 (w), 552 (s), 534 (s), 451 (w), 441 (w), 417 (w). **^{1}H NMR (400 MHz, CDCl$_3$)**: δ = 7.91 – 7.83 (m, 4H, C$H_{arom.}$), 7.57 – 7.51 (m, 2H, C$H_{arom.}$), 7.50 – 7.43 (m, 4H, C$H_{arom.}$), 7.19 – 7.12 (m, 2H, C$H_{arom.}$), 6.90 – 6.84 (m, 2H, C$H_{arom.}$), 3.80 (s, 3H, CH_3), 3.63 – 3.51 (m, 2H, C*H*, CH_2), 3.44 – 3.34 (m, 1H, CH_2), 3.20 – 3.00 (m, 2H, 2xCH_2). **^{13}C NMR (101 MHz, CDCl$_3$)**: δ = 166.6 (d, $^3J_{C-P}$ = 12.4 Hz, *C*=N), 158.6 (C_q), 135.4 (C_q), 132.4 (d, $^4J_{C-P}$ = 2.8 Hz, *C*H), 132.2 (t, $^2J_{C-P}$ = 9.8 Hz, *C*H)[d], 130.8 (d, $^1J_{C-P}$ = 136.2 Hz, C_q), 130.7 (d, $^1J_{C-P}$ = 135.7 Hz, C_q), 128.6 (d, $^3J_{C-P}$ = 13.3 Hz, *C*H), 127.5 (*C*H), 114.2 (*C*H), 55.5 (*C*H$_3$), 40.0 (*C*H$_2$), 39.8 (*C*H$_2$), 31.8 (*C*H). **^{31}P NMR (162 MHz, CDCl$_3$)**: δ = 35.12. **HRMS (ESI):** Calculated for $C_{23}H_{22}NO_3P$ $[M+H]^+$: 392.1416, Found: 392.1411. **Optical Rotation:** $[\alpha]_D^{25}$ = –28.9 (c = 1.0, $CHCl_3$) for an enantiomerically enriched sample of 89:11 er. The enantiomeric purity was established by HPLC analysis using a chiral column (Lux® Cellulose-1, 22 °C, 1 mL/min, 85:15 *n*hexane:isopropanol, 210 nm, t = 47.464 min and 52.304 min).

[d] Appears as triplett due to overlap of 2 carbon signals (dublett)

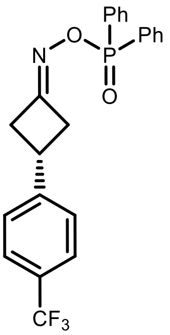

(*R*)-(((3-(4-(Trifluoromethyl)phenyl)cyclobutylidene)amino)oxy)diphenyl phosphine oxide [6d]

Following **GP-J** using 3-[4-(trifluoromethyl)phenyl]cyclobutan-1-one [**1d**] (42.8 mg, 200 µmol, 1.00 eq.), (*R*)-TCYP [**7e**] (19.8 mg, 20.0 µmol, 10 mol%) and *O*-(diphenylphosphinyl)hydroxylamine [**2**] (51.3 mg, 220 µmol, 1.10 eq.) the product (68.7 mg, 160 µmol, 80%) was obtained *via* automated flash column chromatography (SiO_2, Cyclohexane:EtOAc, 100:0 → 0:100, stained with $KMnO_4$) as colorless solid.

M.P.: 120 – 123 °C. **IR (neat)**: $\tilde{\nu}$ = 3061 (w), 2924 (w), 1619 (w), 1592 (w), 1439 (w), 1326 (s), 1232 (m), 1165 (m), 1128 (s), 1113 (s), 1069 (m), 1017 (w), 877 (m), 834 (w), 748 (w), 729 (m), 696 (m), 606 (w), 553 (m), 536 (m), 462 (w), 435 (w). **^{1}H NMR (400 MHz, $CDCl_3$)**: δ = 7.92 – 7.82 (m, 4H, $CH_{arom.}$), 7.60 (d, J = 8.0, 2H, $CH_{arom.}$), 7.58 – 7.52 (m, 2H, $CH_{arom.}$), 7.51 – 7.44 (m, 4H, $CH_{arom.}$), 7.36 (d, J = 8.1 Hz, 2H, $CH_{arom.}$), 3.75 – 3.59 (m, 2H, C*H*, CH_2), 3.51 – 3.42 (m, 1H, CH_2), 3.25 – 3.06 (m, 1H, 2xCH_2). **^{13}C NMR (101 MHz, $CDCl_3$)**: δ = 165.6 (d, $^3J_{C\text{-}P}$ = 12.4 Hz, *C*=N), 147.2 (*C*H), 132.5 (d, $^4J_{C\text{-}P}$ = 2.8 Hz, *C*H), 132.2 (t, $^2J_{C\text{-}P}$ = 10.2 Hz, *C*H)[e], 130.6 (d, $^1J_{C\text{-}P}$ = 136.1 Hz, C_q), 130.6 (d, $^1J_{C\text{-}P}$ = 135.8 Hz, C_q), 129.3 (q, $^2J_{C\text{-}F}$ = 32.5 Hz, C_q), 128.7 (d, $^3J_{C\text{-}P}$ = 13.2 Hz, *C*H), 126.9 (*C*H), 125.8 (q, $^3J_{C\text{-}F}$ = 3.8 Hz, *C*H), 124.2 3 (q, $^1J_{C\text{-}F}$ = 272.1 Hz, C_q), 39.6 (CH_2), 39.4 (CH_2), 32.3 (*C*H). **^{31}P NMR (162 MHz, $CDCl_3$)**: δ = 35.43. **^{19}F NMR (376 MHz, $CDCl_3$)** δ = –62.40. **HRMS (ESI):** Calculated for $C_{23}H_{19}F_3NO_2P$ $[M+H]^+$: 430.1184, Found: 430.1181. **Optical Rotation:** $[\alpha]_D^{25}$ = –22.0 (c = 0.5, $CHCl_3$) for an enantiomerically enriched sample of 93:07 er. The enantiomeric purity was established by HPLC analysis using a chiral column (Lux® Cellulose-1, 22 °C, 1 mL/min, 85:15 *n*hexane:isopropanol, 210 nm, t = 20.683 min and 26.437 min).

[e] Appears as triplett due to overlap of 2 carbon signals (dublett)

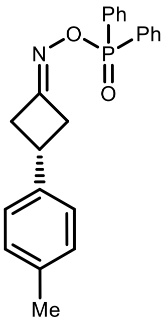

(*R*)-(((3-(*p*-Tolyl)cyclobutylidene)amino)oxy)diphenylphosphine oxide [6e]

Following **GP-J** using 3-(4-methylphenyl)cyclobutanone [**1e**] (32.0 mg, 200 µmol, 1.00 eq.), (*R*)-TCYP [**7e**] (19.8 mg, 20.0 µmol, 10 mol%) and *O*-(diphenylphosphinyl)hydroxylamine [**2**] (51.3 mg, 220 µmol, 1.10 eq.) the product (67.8 mg, 181 µmol, 90%) was obtained *via* automated flash column chromatography (SiO_2, Cyclohexane:EtOAc, 100:0 → 0:100, stained with $KMnO_4$) as colorless solid.

M.P.: 97 – 100 °C. **IR (neat)**: $\tilde{\nu}$ = 3058 (w), 3021 (w), 2921 (w), 1690 (w), 1592 (w), 1516 (w), 1485 (w), 1439 (m), 1396 (w), 1233 (s), 1179 (w), 1129 (s), 1112 (m), 1072 (w), 1028 (w), 999 (w), 963 (w), 877 (s), 811 (m), 729 (s), 696 (s), 648 (w), 627 (w), 579 (w), 554 (s), 532 (s), 479 (w), 463 (w), 449 (w), 438 (w), 417 (w). **^{1}H NMR (400 MHz, $CDCl_3$)**: δ = 7.91 – 7.82 (m, 4H, C$H_{arom.}$), 7.58 – 7.51 (m, 2H, C$H_{arom.}$), 7.51 – 7.42 (m, 4H, C$H_{arom.}$), 7.18 – 7.10 (m, 4H, C$H_{arom.}$), 3.65 – 3.52 (m, 2H, C*H*, CH_2), 3.45 – 3.34 (m, 1H, CH_2), 3.21 – 3.01 (m, 2H, 2xCH_2), 2.34 (s, 3H, CH_3). **^{13}C NMR (101 MHz, $CDCl_3$)**: δ = 166.6 (d, $^{3}J_{C\text{-}P}$ = 12.5 Hz, *C*=N), 140.3 (C_q), 136.6 (C_q), 132.4 (d, $^{4}J_{C\text{-}P}$ = 2.9 Hz, *C*H), 132.2 (d, $^{2}J_{C\text{-}P}$ = 10.6 Hz, *C*H), 132.1 (d, $^{2}J_{C\text{-}P}$ = 10.6 Hz, *C*H), 130.8 (d, $^{1}J_{C\text{-}P}$ = 136.2 Hz, C_q), 130.7 (d, $^{1}J_{C\text{-}P}$ = 135.7 Hz, C_q), 129.5 (*C*H), 128.6 (d, $^{3}J_{C\text{-}P}$ = 13.2 Hz, *C*H), 126.4 (*C*H), 39.8 (CH_2), 39.6 (CH_2), 32.1 (*C*H), 21.1 (CH_3). **^{31}P NMR (162 MHz, $CDCl_3$)**: δ = 35.18. **HRMS (ESI):** Calculated for $C_{23}H_{22}NO_2P$ $[M+H]^+$: 376.1466, Found: 376.1461. **Optical Rotation:** $[\alpha]_D^{25}$ = –43.5 (c = 0.5, $CHCl_3$) for an enantiomerically enriched sample of 91:09 er. The enantiomeric purity was established by HPLC analysis using a chiral column (Lux® Cellulose-1, 22 °C, 1 mL/min, 85:15 *n*hexane:isopropanol, 210 nm, t = 13.930 min and 15.762 min).

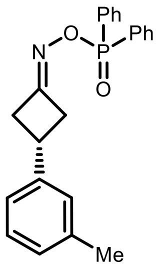

(*R*)-(((3-(m-Tolyl)cyclobutylidene)amino)oxy)diphenylphosphine oxide [6f]

Following **GP-J** using 3-(3-methylphenyl)cyclobutan-1-one [**1f**] (32.0 mg, 200 µmol, 1.00 eq.), (*R*)-TCYP [**7e**] (19.8 mg, 20.0 µmol, 10 mol%) and *O*-(diphenylphosphinyl)hydroxylamine [**2**] (51.3 mg, 220 µmol, 1.10 eq.) the product (62.0 mg, 165 µmol, 83%) was obtained *via* automated flash column chromatography (SiO_2, Cyclohexane:EtOAc, 100:0 → 0:100, stained with $KMnO_4$) as colorless solid.

M.P.: 127 – 129 °C. **IR (neat)**: $\tilde{\nu}$ = 3057 (w), 2922 (w), 1692 (w), 1607 (w), 1591 (w), 1487 (w), 1439 (w), 1398 (w), 1311 (w), 1231 (m), 1180 (w), 1128 (m), 1112 (w), 1071 (w), 1028 (w), 999 (w), 974 (w), 872 (s), 811 (m), 785 (m), 727 (s), 695 (s), 672 (w), 645 (w), 550 (s), 534 (s), 442 (w). **^{1}H NMR (400 MHz, $CDCl_3$)**: δ = 7.93 – 7.82 (m, 4H, $CH_{arom.}$), 7.58 – 7.51 (m, 2H, $CH_{arom.}$), 7.51 – 7.42 (m, 4H, $CH_{arom.}$), 7.26 – 7.20 (m, 1H, $CH_{arom.}$), 7.09 – 7.01 (m, 3H, $CH_{arom.}$), 3.66 – 3.52 (m, 2H, C*H*, CH_2), 3.46 – 3.34 (m, 1H, CH_2), 3.25 – 3.03 (m, 2H, 2xCH_2), 2.35 (s, 3H, CH_3). **^{13}C NMR (101 MHz, $CDCl_3$)**: δ = 166.6 (d, $^3J_{C\text{-}P}$ = 12.3 Hz, *C*=N), 143.2 (C_q), 138.5 (C_q), 132.4 (d, $^4J_{C\text{-}P}$ = 2.9 Hz, *C*H), 132.2 (t, $^2J_{C\text{-}P}$ = 10.2 Hz, *C*H)[f], 130.8 (d, $^1J_{C\text{-}P}$ = 136.2 Hz, C_q), 130.7 (d, $^1J_{C\text{-}P}$ = 135.7 Hz, C_q), 128.7 (*C*H), 128.6 (d, $^3J_{C\text{-}P}$ = 13.1 Hz, *C*H), 127.7 (*C*H), 127.2 (*C*H), 123.5 (*C*H), 39.7 (CH_2), 39.5 (CH_2), 32.4 (*C*H), 21.6 (CH_3). **^{31}P NMR (162 MHz, $CDCl_3$)**: δ = 35.17. **HRMS (ESI):** Calculated for $C_{23}H_{22}NO_2P$ $[M+H]^+$: 376.1466, Found: 376.1464. **Optical Rotation:** $[\alpha]_D^{25}$ = –46.9 (c = 0.5, $CHCl_3$) for an enantiomerically enriched sample of 93:07 er. The enantiomeric purity was established by HPLC analysis using a chiral column (Lux® Cellulose-1, 22 °C, 1 mL/min, 85:15 *n*hexane:isopropanol, 210 nm, t = 13.238 min and 16.004 min).

[f] Appears as triplett due to overlap of 2 carbon signals (dublett)

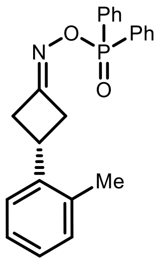

(*R*)-(((3-(*o*-Tolyl)cyclobutylidene)amino)oxy)diphenylphosphine oxide [6g]

Following **GP-J** using 3-(2-methylphenyl)cyclobutanone [**1g**] (32.0 mg, 200 µmol, 1.00 eq.), (*R*)-TCYP [**7e**] (19.8 mg, 20.0 µmol, 10 mol%) and *O*-(diphenylphosphinyl)hydroxylamine [**2**] (51.3 mg, 220 µmol, 1.10 eq.) the product (67.2 mg, 179 µmol, 90%) was obtained *via* automated flash column chromatography (SiO_2, Cyclohexane:EtOAc, 100:0 → 0:100, stained with $KMnO_4$) as colorless solid.

M.P.: 137 – 138 °C. **IR (neat)**: $\tilde{\nu}$ = 3059 (w), 2926 (w), 1692 (w), 1592 (w), 1489 (w), 1460 (w), 1439 (m), 1398 (w), 1230 (m), 1182 (w), 1128 (m), 1112 (m), 1071 (w), 1027 (w), 998 (w), 872 (s), 812 (m), 726 (s), 693 (s), 667 (m), 645 (w), 548 (s), 532 (s), 441 (w). **^{1}H NMR (400 MHz, $CDCl_3$)**: δ = 7.95 – 7.82 (m, 4H, $CH_{arom.}$), 7.59 – 7.51 (m, 2H, $CH_{arom.}$), 7.51 – 7.43 (m, 4H, $CH_{arom.}$), 7.25 – 7.19 (m, 2H, $CH_{arom.}$), 7.19 – 7.14 (m, 2H, $CH_{arom.}$), 3.75 (app. p, J ≈ 8.4 Hz, 1H, C*H*), 3.65 – 3.53 (m, 1H, CH_2), 3.44-3.33 (m, 1H, CH_2), 3.23 – 3.05 (m, 2H, CH_2), 2.26 (s, 3H, CH_3). **^{13}C NMR (101 MHz, $CDCl_3$)**: δ = 166.3 (d, $^3J_{C\text{-}P}$ = 12.3 Hz, *C*=N), 140.3 (C_q), 136.2 (C_q), 132.4 (d, $^4J_{C\text{-}P}$ = 2.9 Hz, *C*H), 132.2 (d, $^2J_{C\text{-}P}$ = 10.0 Hz, *C*H), 132.1 (d, $^2J_{C\text{-}P}$ = 10.1 Hz, *C*H), 130.8 (d, $^1J_{C\text{-}P}$ = 136.4 Hz, C_q), 130.7 (d, $^1J_{C\text{-}P}$ = 135.7 Hz, C_q), 130.6 (*C*H), 128.6 (d, $^3J_{C\text{-}P}$ = 13.1 Hz, *C*H), 127.0 (*C*H), 126.4 (*C*H), 124.8 (*C*H), 38.3 (CH_2), 38.2 (CH_2), 30.3 (*C*H), 19.8 (CH_3). **^{31}P NMR (162 MHz, $CDCl_3$)**: δ = 35.18. **HRMS (ESI):** Calculated for $C_{23}H_{22}NO_2P$ $[M+H]^+$: 376.1466, Found: 376.1461. **Optical Rotation:** $[\alpha]_D^{25}$ = –53.9 (c = 0.5, $CHCl_3$) for an enantiomerically enriched sample of 95:05 er. The enantiomeric purity was established by HPLC analysis using a chiral column (Lux® Cellulose-1, 22 °C, 1 mL/min, 85:15 *n*hexane:isopropanol, 210 nm, t = 17.260 min and 20.956 min).

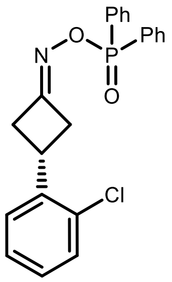

(*R*)-(((3-(2-chlorophenyl)cyclobutylidene)amino)oxy)diphenylphosphine oxide [6h]

Following **GP-J** using 3-(2-chlorophenyl)cyclobut-1-one [**1h**] (90.3 mg, 500 µmol, 1.00 eq.), (*R*)-TCYP [**7e**] (50.7 mg, 50.0 µmol, 10 mol%) and *O*-(diphenylphosphinyl)hydroxylamine [**2**] (128.3 mg, 550 µmol, 1.10 eq.) the product (181 mg, 457 µmol, 91%) was obtained *via* automated flash column chromatography (SiO_2, Cyclohexane:EtOAc, 100:0 → 0:100, stained with $KMnO_4$) as colorless solid.

M.P.: 115 – 117 °C. **IR (neat)**: $\tilde{\nu}$ = 3058 (w), 1475 (w), 1439 (m), 1231 (m), 1129 (m), 1112 (w), 877 (s), 818 (w), 751 (m), 729 (s), 695 (s), 655 (w), 553 (s), 492 (m), 455 (m), 425 (m). **^{1}H NMR (400 MHz, $CDCl_3$)**: δ = 7.92 – 7.83 (m, 4H, C$H_{arom.}$), 7.58 – 7.51 (m, 2H, C$H_{arom.}$), 7.51 – 7.43 (m, 4H, C$H_{arom.}$), 7.39 – 7.35 (m, 1H, C$H_{arom.}$), 7.32 – 7.27 (m, 2H, C$H_{arom.}$), 7.22 – 7.18 (m, 1H, C$H_{arom.}$), 3.92 (tt, *J* = 8.8, 7.8 Hz, 1H, C*H*), 3.65 (*app.* ddt, *J* ≈ 17.5, 9.1, 3.4 Hz, 1H, CH_2), 3.45 (*app.* ddt, *J* ≈ 16.9, 9.0, 3.4 Hz, 1H, CH_2), 3.16 (dddd, *J* = 22.0, 16.9, 7.8, 3.0 Hz, 2H, CH_2). **^{13}C NMR (101 MHz, $CDCl_3$)**: δ = 166.1 (d, $^3J_{C-P}$ = 12.5 Hz, C_q), 139.7 (C_q), 134.1 (C_q), 132.5 (d, $^4J_{C-P}$ = 2.9 Hz, *C*H), 132.2 (d, $^2J_{C-P}$ = 10.1 Hz, *C*H), 132.2 (d, $^2J_{C-P}$ = 10.1 Hz, *C*H), 130.7 (d, $^1J_{C-P}$ = 136.1 Hz, C_q), 130.7 (d, $^1J_{C-P}$ = 135.9 Hz, C_q), 129.9 (*C*H), 128.7 (d, $^3J_{C-P}$ = 13.2 Hz, *C*H), 128.3 (*C*H), 127.2 (*C*H), 126.8 (*C*H), 38.1 (CH_2), 38.0 (CH_2), 30.7 (*C*H). **^{31}P NMR (162 MHz, $CDCl_3$)**: δ = 35.25. **HRMS (ESI):** Calculated for $C_{22}H_{18}ClNO_2P$ $[M+H]^+$: 430.0517, Found: 430.0525. **Optical Rotation:** $[\alpha]_D^{25}$ = –67.8 (c = 0.5, $CHCl_3$) for an enantiomerically enriched sample of 93:7 *er*. The enantiomeric purity was established by HPLC analysis using a chiral column (Cellulose-1, 22 °C, 1 mL/min, 85:15 *n*-hexane:isopropanol, 214 nm, t = 14.493 min and 16.558 min).

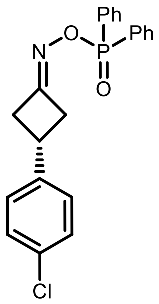

(*R*)-(((3-(4-Chlorophenyl)cyclobutylidene)amino)oxy)diphenylphosphine oxide [6i]

Following **GP-J** using 3-(4-chlorophenyl)cyclobutan-1-one [**1i**] (36.1 mg, 200 µmol, 1.00 eq.), (*R*)-TCYP [**7e**] (19.8 mg, 20.0 µmol, 10 mol%) and *O*-(diphenylphosphinyl)hydroxylamine [**2**] (51.3 mg, 220 µmol, 1.10 eq.) the product (60.5 mg, 153 µmol, 76%) was obtained *via* automated flash column chromatography (SiO_2, Cyclohexane:EtOAc, 100:0 → 0:100, stained with $KMnO_4$) as colorless solid.

M.P.: 139 – 145 °C. **IR (neat)**: $\tilde{\nu}$ = 3057 (w), 2235 (w), 1693 (w), 1592 (w), 1493 (m), 1439 (m), 1399 (w), 1232 (s), 1180 (w), 1129 (s), 1112 (m), 1092 (m), 1014 (w), 876 (s), 817 (m), 763 (m), 729 (s), 695 (s), 584 (w), 553 (s), 537 (s). **^{1}H NMR (400 MHz, $CDCl_3$)**: δ = 7.87 (m, 4H, C$H_{arom.}$), 7.58 – 7.51 (m, 2H, C$H_{arom.}$), 7.51 – 7.43 (m, 4H, C$H_{arom.}$), 7.33 – 7.28 (m, 2H, C$H_{arom.}$), 7.20 – 7.13 (m, 2H, C$H_{arom.}$), 3.66 – 3.54 (m, 2H, C*H*, CH_2), 3.47 – 3.35 (m, 1H, CH_2), 3.21 – 3.01 (m, 2H, CH_2). **^{13}C NMR (101 MHz, $CDCl_3$)**: δ = 166.0 (d, $^3J_{C-P}$ = 12.4 Hz, *C*=N), 141.7 (C_q), 132.7 (C_q), 132.5 (d, $^4J_{C-P}$ = 2.9 Hz, *C*H), 132.2 (app. t, $^2J_{C-P}$ = 9.8 Hz, *C*H), 130.7 (d, $^1J_{C-P}$ = 136.2 Hz, C_q), 130.6 (d, $^1J_{C-P}$ = 135.7 Hz, C_q), 129.0 (*C*H), 128.7 (d, $^3J_{C-P}$ = 13.2 Hz, *C*H), 127.9 (*C*H), 39.7 (CH_2), 39.5 (CH_2), 32.0 (*C*H). **^{31}P NMR (162 MHz, $CDCl_3$)**: δ = 35.4. **HRMS (ESI):** Calculated for $C_{22}H_{19}ClNO_2P$ $[M+H]^+$: 309.0920, Found: 396.0916. **Optical Rotation:** $[\alpha]_D^{25}$ = –57.3 (c = 0.5, $CHCl_3$) for an enantiomerically enriched sample of 93:07 er. The enantiomeric purity was established by HPLC analysis using a chiral column (Lux® Cellulose-1, 22 °C, 1 mL/min, 93:07 *n*-hexane:isopropanol, 210 nm, t = 20.341 min and 23.495 min).

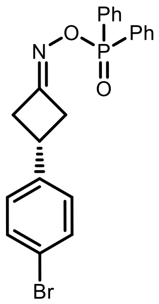

(*R*)-(((3-(4-Bromophenyl)cyclobutylidene)amino)oxy)diphenylphosphine oxide [6j]

Following **GP-J** using 3-(4-bromophenyl)cyclobutan-1-one [**1j**] (45.0 mg, 200 µmol, 1.00 eq.), (*R*)-TCYP [**7e**] (19.8 mg, 20.0 µmol, 10 mol%) and *O*-(diphenylphosphinyl)hydroxylamine [**2**] (51.3 mg, 220 µmol, 1.10 eq.) the product (74.3 mg, 169 µmol, 84%) was obtained *via* automated flash column chromatography (SiO_2, Cyclohexane:EtOAc, 100:0 → 0:100, stained with $KMnO_4$) as colorless solid.

M.P.: 126 – 134 °C. **IR (neat)**: $\tilde{\nu}$ = 3059 (w), 2927 (w), 2230 (w), 2029 (w), 2003 (w), 1692 (w), 1592 (w), 1488 (m), 1439 (m), 1398 (w), 1230 (s), 1180 (w), 1129 (s), 1112 (m), 1072 (w), 1027 (w), 1010 (w), 965 (w), 890 (s), 874 (s), 814 (m), 753 (m), 728 (s), 695 (s), 645 (w), 553 (s), 536 (s), 524 (m), 452 (w), 419 (w). **^{1}H NMR (400 MHz, $CDCl_3$)**: δ = 7.90 – 7.82 (m, 4H, C$H_{arom.}$), 7.59 – 7.51 (m, 2H, C$H_{arom.}$), 7.50 – 7.43 (m, 6H, C$H_{arom.}$), 7.14 – 7.08 (m, 2H, C$H_{arom.}$), 3.66 – 3.53 (m, 2H, C*H*, CH_2), 3.48 – 3.36 (m, 1H, CH_2), 3.20 – 3.00 (m, 2H, CH_2). **^{13}C NMR (101 MHz, $CDCl_3$)**: δ = 165.9 (d, $^3J_{C-P}$ = 12.5 Hz, *C*=N), 142.2 (C_q), 132.5 (d, $^4J_{C-P}$ = 2.9 Hz, *C*H), 132.2 (app. t, $^2J_{C-P}$ = 9.8 Hz, *C*H), 131.9 (*C*H), 130.7 (d, $^1J_{C-P}$ = 136.1 Hz, C_q), 130.6 (d, $^1J_{C-P}$ = 135.7 Hz, C_q), 128.7 (d, $^3J_{C-P}$ = 13.2 Hz, *C*H), 128.3 (*C*H), 120.8 (C_q), 39.6 (CH_2), 39.5 (CH_2), 32.0 (*C*H). **^{31}P NMR (162 MHz, $CDCl_3$)**: δ = 35.42. **HRMS (ESI):** Calculated for $C_{22}H_{19}BrNO_2P$ $[M+H]^+$: 440.0415, Found: 440.0412. **Optical Rotation:** $[\alpha]_D^{25}$ = –60.8 (c = 0.5, $CHCl_3$) for an enantiomerically enriched sample of 93:07 er. The enantiomeric purity was established by HPLC analysis using a chiral column (Lux® Cellulose-1, 22 °C, 1 mL/min, 85:15 *n*hexane:isopropanol, 210 nm, t = 22.481 min and 26.136 min).

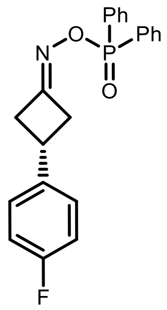

(*R*)-(((3-(4-Fluorophenyl)cyclobutylidene)amino)oxy)diphenylphosphine oxide [6k]

Following **GP-J** using 3-(4-fluorophenyl)cyclobutan-1-one [**1k**] (32.8 mg, 200 µmol, 1.00 eq.), (*R*)-TCYP [**7e**] (19.8 mg, 20.0 µmol, 10 mol%) and *O*-(diphenylphosphinyl)hydroxylamine [**2**] (51.3 mg, 220 µmol, 1.10 eq.) the product (71.0 mg, 187 µmol, 94%) was obtained *via* automated flash column chromatography (SiO_2, Cyclohexane:EtOAc, 100:0 → 0:100, stained with $KMnO_4$) as colorless solid.

M.P.: 84 – 89 °C. **IR (neat)**: $\tilde{\nu}$ = 2955 (w), 2924 (m), 2854 (w), 1604 (w), 1510 (s), 1439 (m), 1229 (s), 1180 (w), 1160 (w), 1129 (s), 1112 (m), 876 (s), 831 (m), 813 (m), 729 (s), 696 (s), 625 (w), 553 (s), 532 (s). **^{1}H NMR (600 MHz, $CDCl_3$)**: δ = 7.90 – 7.83 (m, 4H, C$H_{arom.}$), 7.57 – 7.52 (m, 2H, C$H_{arom.}$), 7.50 – 7.44 (m, 4H, C$H_{arom.}$), 7.23 – 7.17 (m, 2H, C$H_{arom.}$), 7.06 – 6.99 (m, 2H, C$H_{arom.}$), 3.65 – 3.56 (m, 2H, C*H*, CH_2), 3.46 – 3.38 (m, 1H, CH_2), 3.14 (qd, J = 10.3, 3.2 Hz, 1H, CH_2), 3.09 – 3.03 (m, 1H, CH_2). **^{13}C NMR (151 MHz, $CDCl_3$)**: δ = 166.1 (d, $^3J_{C\text{-}P}$ = 12.4 Hz, C_q), 161.8 (d, $^1J_{C\text{-}F}$ = 245.2 Hz, C_q), 138.9 (d, $^4J_{C\text{-}F}$ = 3.4 Hz, C_q), 132.5 (d, $^4J_{C\text{-}P}$ = 2.9 Hz, *C*H), 132.2 (d, $^2J_{C\text{-}P}$ = 9.8 Hz, *C*H), 132.1 (d, $^2J_{C\text{-}P}$ = 10.1 Hz, *C*H), 130.7 (d, $^1J_{C\text{-}P}$ = 136.0 Hz, C_q), 130.6 (d, $^1J_{C\text{-}P}$ = 135.6 Hz, C_q), 128.7 (d, $^3J_{C\text{-}P}$ = 13.2 Hz, *C*H), 128.0 (d, $^3J_{C\text{-}F}$ = 7.9 Hz, *C*H), 115.7 (d, $^2J_{C\text{-}F}$ = 21.4 Hz, *C*H), 39.90, 39.71, 31.85. **^{31}P NMR (162 MHz, $CDCl_3$)**: δ = 35.29. **^{19}F NMR** (376 MHz, $CDCl_3$) δ = -115.79 (tt, J = 8.8, 4.9 Hz). **HRMS (ESI):** Calculated for $C_{22}H_{19}FNO_2P$ $[M+H]^+$: 380.1210, Found: 380.1203. **Optical Rotation:** $[\alpha]_D^{25}$ = –63.3 (c = 0.5, $CHCl_3$) for an enantiomerically enriched sample of 90:10 er. The enantiomeric purity was established by HPLC analysis using a chiral column (Lux® Cellulose-1, 22 °C, 1 mL/min, 85:15 *n*-hexane:isopropanol, 210 nm, t = 16.751 min and 18.715 min).

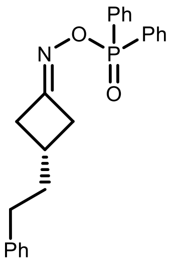

(*R*)-(((3-Phenethylcyclobutylidene)amino)oxy)diphenylphosphine oxide [6l]

Following **GP-J** using 3-phenethylcyclobutan-1-one [**1l**] (34.9 mg, 200 µmol, 1.00 eq.), (*R*)-TCYP [**7e**] (19.8 mg, 20.0 µmol, 10 mol%) and *O*-(diphenylphosphinyl)hydroxylamine [**2**] (51.3 mg, 220 µmol, 1.10 eq.) the product (56.0 mg, 144 µmol, 72%) was obtained *via* automated flash column chromatography (SiO_2, Cyclohexane:EtOAc, 100:0 → 0:100, stained with $KMnO_4$) as colorless solid.

M.P.: 105 – 110 °C. **IR (neat)**: $\tilde{\nu}$ = 2923 (m), 2854 (w), 1496 (w), 1455 (w), 1439 (m), 1395 (w), 1237 (s), 1178 (w), 1129 (s), 1112 (m), 1072 (m), 1029 (w), 880 (s), 811 (m), 753 (m), 729 (s), 698 (s), 553 (s), 532 (s), 454 (w), 418 (w). **^{1}H NMR (400 MHz, $CDCl_3$)**: δ = 7.90 – 7.81 (m, 4H, C$H_{arom.}$), 7.57 – 7.50 (m, 2H, C$H_{arom.}$), 7.50 – 7.41 (m, 4H, C$H_{arom.}$), 7.31 – 7.26 (m, 2H, C$H_{arom.}$), 7.23 – 7.17 (m, 1H, C$H_{arom.}$), 7.16 – 7.12 (m, 2H, C$H_{arom.}$), 3.22 (*app.* ddt, J ≈ 17.6, 8.7, 3.1 Hz, 1H, CH_2), 3.05 (*app.* ddt, J ≈ 16.8, 8.6, 3.0 Hz, 1H, CH_2), 2.72 – 2.51 (m, 4H, 2xCH_2), 2.43 – 2.28 (m, 1H, C*H*), 1.84 (q, J = 7.6 Hz, 2H, CH_2). **^{13}C NMR (101 MHz, $CDCl_3$)**: δ = 167.4 (d, $^3J_{C\text{-}P}$ = 12.4 Hz, *C*=N), 141.4 (C_q), 132.3 (d, $^4J_{C\text{-}P}$ = 2.8 Hz, *C*H), 132.1 (d, $^2J_{C\text{-}P}$ = 10.2 Hz, *C*H), 132.0 (d, $^2J_{C\text{-}P}$ = 10.0 Hz, *C*H), 130.8 (d, $^1J_{C\text{-}P}$ = 136.8 Hz, C_q), 130.7 (d, $^1J_{C\text{-}P}$ = 135.8 Hz, C_q), 128.5 (d, $^3J_{C\text{-}P}$ = 13.2 Hz, *C*H), 128.5 (*C*H), 128.4 (*C*H), 126.0 (*C*H), 37.7 (CH_2), 37.4 (CH_2), 37.3 (CH_2), 33.7 (CH_2), 27.7 (*C*H). **^{31}P NMR (162 MHz, $CDCl_3$)**: δ = 34.93. **HRMS (ESI):** Calculated for $C_{24}H_{24}NO_2P$ $[M+H]^+$: 390.1623, Found: 390.1605. **Optical Rotation:** $[\alpha]_D^{25}$ = –10.5 (c = 1.0, $CHCl_3$) for an enantiomerically enriched sample of 89:11 er. The enantiomeric purity was established by HPLC analysis using a chiral column (Lux® Cellulose-1, 22 °C, 1 mL/min, 85:15 *n*hexane:isopropanol, 210 nm, t = 27.395 min and 35.050 min).

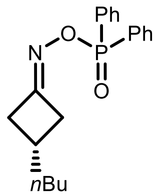

(*R*)-(((3-Butylcyclobutylidene)amino)oxy)diphenylphosphine oxide [6m]

Following **GP-J** using 3-butylcyclobut-1-one [**1m**] (25.2 mg, 200 µmol, 1.00 eq.), (*R*)-TCYP [**7e**] (19.8 mg, 20.0 µmol, 10 mol%) and *O*-(diphenylphosphinyl)hydroxylamine [**2**] (51.3 mg, 220 µmol, 1.10 eq.) the product (49.0 mg, 144 µmol, 72%) was obtained *via* automated flash column chromatography (SiO_2, Cyclohexane:EtOAc, 100:0 → 0:100, stained with $KMnO_4$) as colorless solid.

M.P.: 55 – 62 °C. **IR (neat)**: $\tilde{\nu}$ = 2957 (w), 2926 (m), 2855 (w), 1694 (w), 1439 (m), 1237 (m), 1129 (s), 1113 (w), 880 (s), 809 (w), 753 (w), 729 (s), 696 (s), 553 (s), 534 (s), 444 (w). **^{1}H NMR (400 MHz, $CDCl_3$)**: δ = 7.85 (ddt, J = 12.2, 8.4, 1.6 Hz, 4H, C$H_{arom.}$), 7.60 – 7.37 (m, 6H, C$H_{arom.}$), 3.21 (ddt, J = 17.5, 8.7, 3.1 Hz, 1H, CH_2), 3.04 (ddt, J = 16.8, 8.6, 3.1 Hz, 1H, CH_2), 2.69 – 2.47 (m, 2H, CH_2), 2.41 – 2.24 (m, 1H, C*H*), 1.50 (q, J = 7.5 Hz, 2H, CH_2), 1.39 – 1.15 (m, 4H, CH_2), 0.89 (t, J = 7.0 Hz, 3H, CH_3). **^{13}C NMR (101 MHz, $CDCl_3$)**: δ = 168.0 (d, $^3J_{C\text{-}P}$ = 12.5 Hz, C_q), 132.3 (d, $^4J_{C\text{-}P}$ = 2.9 Hz, *C*H), 132.2 (d, $^2J_{C\text{-}P}$ = 9.9 Hz, *C*H), 132.1 (d, $^2J_{C\text{-}P}$ = 10.0 Hz, *C*H), 130.9 (d, $^1J_{C\text{-}P}$ = 136.1 Hz, C_q), 130.9 (d, $^1J_{C\text{-}P}$ = 135.8 Hz, C_q), 128.6 (d, $^3J_{C\text{-}P}$ = 13.2 Hz, *C*H), 37.6 (*C*H_2), 37.5 (*C*H_2), 35.9 (*C*H), 29.6 (*C*H_2), 28.3 (*C*H_2), 22.6 (*C*H_2), 14.2 (*C*H_3). **^{31}P NMR (162 MHz, $CDCl_3$)**: δ = 34.9. **HRMS (ESI):** Calculated for $C_{20}H_{24}NO_2P$ $[M+H]^+$: 342.1617, Found: 342.1622. **Optical Rotation:** $[\alpha]_D^{25}$ = +43.9 (c = 0.5, $CHCl_3$) for an enantiomerically enriched sample of 10:90 er. The enantiomeric purity was established by HPLC analysis using a chiral column (Reprosil Chiral-AMS, 22 °C, 1 mL/min, 90:10 *n*-hexane:isopropanol, 210 nm, t = 15.594 min and 19.123 min).

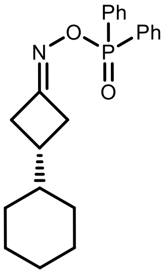

(*R*)-(((3-cyclohexylcyclobutylidene)amino)oxy)diphenylphosphine oxide [6n]

Following **GP-J** using 3-cyclohexylcyclobutanone [**1n**] (30.4 mg, 200 µmol, 1.00 eq.), (*R*)-TCYP [**7e**] (19.8 mg, 20.0 µmol, 10 mol%) and *O*-(diphenylphosphinyl)hydroxylamine [**2**] (51.3 mg, 220 µmol, 1.10 eq.) the product (40.8 mg, 111 µmol, 56%) was obtained *via* automated flash column chromatography (SiO_2, Cyclohexane:EtOAc, 100:0 → 0:100, stained with $KMnO_4$) as colorless solid.

M.P.: 116 – 119 °C. **IR (neat)**: $\tilde{\nu}$ = 2922 (s), 2850 (m), 1439 (m), 1395 (w), 1236 (s), 1179 (w), 1129 (s), 1112 (m), 1072 (w), 880 (s), 810 (w), 753 (w), 729 (s), 712 (m), 696 (s), 553 (s), 537 (m). **^{1}H NMR (400 MHz, CDCl$_3$)**: δ = 7.89 – 7.80 (m, 4H, C$H_{arom.}$), 7.56 – 7.49 (m, 2H, C$H_{arom.}$), 7.49 – 7.41 (m, 4H, C$H_{arom.}$), 3.16 (ddt, J = 17.5, 8.7, 3.2 Hz, 1H, CH_2), 2.97 (ddt, J = 16.8, 8.6, 3.2 Hz, 1H, CH_2), 2.75 – 2.54 (m, 2H, CH_2), 2.09 – 1.97 (m, 1H, C*H*), 1.78 – 1.62 (m, 4H, 2xCH_2), 1.27 – 1.07 (m, 5H, C*H*, 2xCH_2), 0.91 – 0.76 (m, 2H, CH_2). **^{13}C NMR (101 MHz, CDCl$_3$)**: δ = 167.8 (d, $^3J_{C\text{-}P}$ = 12.5 Hz, *C*=N), 132.3 (d, $^4J_{C\text{-}P}$ = 2.8 Hz, *C*H), 132.2 (d, $^2J_{C\text{-}P}$ = 10.0 Hz, *C*H), 132.1 (d, $^2J_{C\text{-}P}$ = 9.9 Hz, *C*H), 131.0 (d, $^1J_{C\text{-}P}$ = 136.3 Hz, C_q), 130.9 (d, $^1J_{C\text{-}P}$ = 135.7 Hz, C_q), 128.6 (d, $^3J_{C\text{-}P}$ = 13.2 Hz, *C*H), 43.5 (*C*H), 36.0 (CH_2), 35.9 (CH_2), 34.2 (*C*H), 30.2 (CH_2), 30.2 (CH_2), 26.4 (CH_2), 26.0 (CH_2). **^{31}P NMR (162 MHz, CDCl$_3$)**: δ = 34.79. **HRMS (ESI):** Calculated for $C_{22}H_{26}NO_2P$ $[M+H]^+$: 368.1179, Found: 368.1761. **Optical Rotation:** $[\alpha]_D^{25}$ = +11.1 (c = 0.5, $CHCl_3$) for an enantiomerically enriched sample of 82:18 er. The enantiomeric purity was established by HPLC analysis using a chiral column (Lux® Cellulose-1, 22 °C, 1 mL/min, 85:15 *n*hexane:isopropanol, 210 nm, t = 10.090 min and 11.546 min).

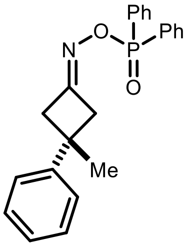

(((3-Methyl-3-phenylcyclobutylidene)amino)oxy)diphenylphosphine oxide [6o]

Following **GP-J** using 3-methyl-3-phenylcyclobutan-1-one [**1o**] (32.0 mg, 200 µmol, 1.00 eq.), (*R*)-TCYP [**7e**] (19.8 mg, 20.0 µmol, 10 mol%) and *O*-(diphenylphosphinyl)hydroxylamine [**2**] (51.3 mg, 220 µmol, 1.10 eq.) the product (43.0 mg, 115 µmol, 57%) was obtained *via* automated flash column chromatography (SiO_2, Cyclohexane:EtOAc, 100:0 → 0:100, stained with $KMnO_4$) as colorless solid.

M.P.: 109 – 115 °C. **IR (neat)**: $\tilde{\nu}$ = 3057 (w), 2956 (w), 2922 (w), 1695 (w), 1592 (w), 1496 (w), 1439 (m), 1398 (w), 1238 (s), 1183 (w), 1128 (s), 1112 (m), 1073 (w), 1028 (w), 874 (s), 812 (m), 745 (m), 728 (s), 697 (s), 663 (w), 554 (s), 544 (s). **^{1}H NMR (400 MHz, $CDCl_3$)**: δ = 7.92 – 7.79 (m, 4H, C$H_{arom.}$), 7.58 – 7.50 (m, 2H, C$H_{arom.}$), 7.50 – 7.42 (m, 4H, C$H_{arom.}$), 7.38 – 7.31 (m, 2H, C$H_{arom.}$), 7.25 – 7.19 (m, 3H, C$H_{arom.}$), 3.44 – 3.29 (m, 2H, CH_2), 3.25 (ddd, J = 17.0, 3.8, 2.8 Hz, 1H, CH_2), 3.04 (ddd, J = 16.3, 3.8, 2.8 Hz, 1H, CH_2), 1.53 (s, 3H, CH_3). **^{13}C NMR (101 MHz, $CDCl_3$)**: δ = 165.7 (d, $^3J_{C\text{-}P}$ = 12.5 Hz, C_q), 148.2 (C_q), 132.4 (t, $^4J_{C\text{-}P}$ = 2.6 Hz, *C*H)[g], 132.1 (t, $^2J_{C\text{-}P}$ = 10.3, *C*H)[a], 130.8 (d, $^1J_{C\text{-}P}$ = 136.3, C_q), 130.7 (d, $^1J_{C\text{-}P}$ = 136.3, C_q), 128.7 (*C*H), 128.6 (d, $^3J_{C\text{-}P}$ = 13.4 Hz, *C*H), 126.4 (*C*H), 125.2 (*C*H), 44.8 (*C*H_2), 44.8 (*C*H_2), 37.9 (C_q), 30.9 (*C*H_3). **^{31}P NMR (162 MHz, $CDCl_3$)**: δ = 35.09. **HRMS (ESI):** Calculated for $C_{23}H_{22}NO_2P$ $[M+H]^+$: 376.1461, Found: 376.1455. **Optical Rotation:** $[\alpha]_D^{25}$ = +11.5 (c = 1.0, $CHCl_3$) for an enantiomerically enriched sample of 35:65 er. The enantiomeric purity was established by HPLC analysis using a chiral column (Lux® Cellulose-1, 22 °C, 1 mL/min, 85:15 *n*hexane:isopropanol, 210 nm, t = 11.836 min and 13.036 min).

---

[g] Appears as triplet due to overlap of two signals (doublet)

6. Test Experiments Application

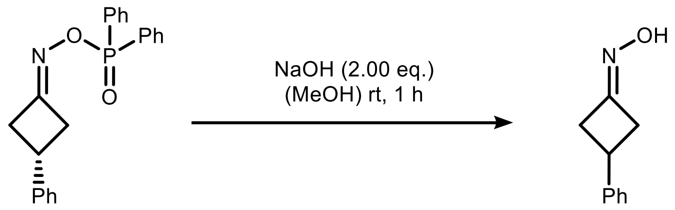

6a 95:5 er; *rac*-**8a** 83%, 50:50 er

In a round bottom flask, oxime ester [**6a**] (36.1 mg, 100 µmol, 1.00 eq.) was dissolved in a solution of NaOH (8.00 mg, 200 µmol, 2.00 eq.) in MeOH (2.0 mL). The reaction mixture was stirred at room temperature for 16 h. Water was added and the mixture was diluted with CH_2Cl_2. The phases were separated and the aqueous phase was extracted (5 x 10 mL) with CH_2Cl_2. The combined organic phases were dried over $MgSO_4$, filtered and concentrated under reduced pressure. The product **8a** (13.4 mg, 83.1 µmol, 83%) was obtained *via* automated column chromatography (SiO_2, cyclohexane:EtOAc, 50:50, stained with $KMnO_4$).

^{1}H NMR (400 MHz, $CDCl_3$): δ = 7.46 – 7.16 (m, 5H), 3.62 (ddd, J = 16.2, 9.4, 7.6 Hz, 1H), 3.51 – 3.42 (m, 1H), 3.36 (ddt, J = 16.5, 9.0, 3.1 Hz, 1H), 3.05 (dddd, J = 16.4, 7.3, 3.1, 1.5 Hz, 2H).**^{13}C NMR (101 MHz, $CDCl_3$)** δ = 156.9, 144.1, 128.8, 126.8, 126.6, 39.5, 38.3, 32.9. The spectroscopic data was in agreement to those previously reported.[3,30]

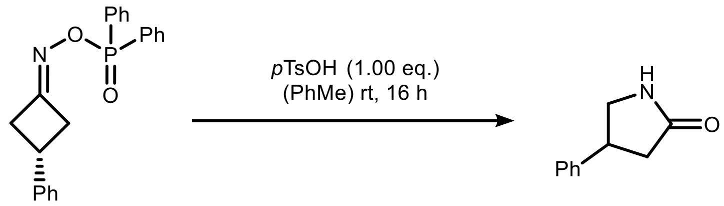

6a 95:5 er; *rac*-**3a** 82%, 50:50 er

In a round bottom flask, oxime ester [**6a**] (36.1 mg, 100 µmol, 1.00 eq.) was dissolved in dry toluene (2.0 mL). *p*-Toluenesulfonic acid (17.2 mg, 100 µmol, 1.00 eq.) was added and the reaction mixture was stirred at room temperature for 16 h. Water was added and the mixture was diluted with CH_2Cl_2. The phases were separated and the aqueous phase was extracted (5 x 10 mL) with CH_2Cl_2. The combined organic phases were dried over $MgSO_4$, filtered and concentrated under reduced pressure. The product (13.2 mg, 81.8 µmol, 82%) was obtained *via* column chromatography (SiO_2, EtOAc:MeOH, 95:5, stained with $KMnO_4$).

^{1}H NMR (400 MHz, $CDCl_3$): δ = 7.39 – 7.29 (m, 2H), 7.29 – 7.22 (m, 3H), 6.70 (br s, 1H), 3.82 – 3.74 (m, 1H), 3.74 – 3.62 (m, 1H), 3.41 (dd, J = 9.3, 7.2 Hz, 1H), 2.73 (dd, J = 16.9, 8.8 Hz, 1H), 2.51 (dd, J = 16.9, 8.8 Hz, 1H). **^{13}C NMR (101 MHz, $CDCl_3$)** δ = 177.9, 142.2, 129.0, 127.2, 126.9, 49.7, 40.4, 38.1. The spectroscopic data was in agreement to those previously reported.[31]

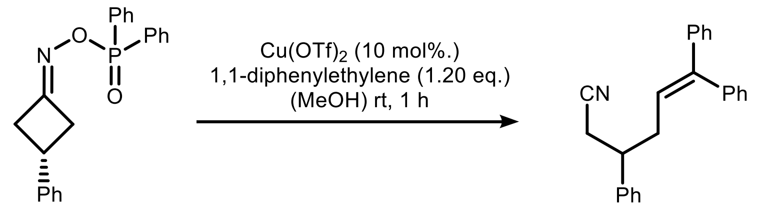

6a
95:5 er

rac-**9a**
79%, 50:50 er

Following a procedure by Zhao *et al.*[32], an oven dried Schlenk tube was charged with copper(II) trifluoromethanesulfonate (3.62 mg, 10.0 µmol, 10 mol%) and oxime ester [**6a**] (36.1 mg, 100 µmol, 1.00 equiv) under N_2 atmosphere. Dry 1,4-dioxane (500 µL), $PhCF_3$ (500 µL) and 1-phenylethenylbenzene (21.6 mg, 21.2 µL, 120 µmol, 1.20 eq.) was added. The resulting reaction mixture was stirred at 90 °C for 16 h. The solvent was removed under reduced pressure and the crude product was purified *via* automated flash column chromatography (95:5 pent:EtOAc) to obtain the title compound (25.6 mg, 79.2 µmol, 79%) as yellow oil.

^{1}H NMR (400 MHz, $CDCl_3$): δ = 7.41 – 7.30 (m, 5H), 7.30 – 7.17 (m, 4H), 7.17 – 7.02 (m, 6H), 5.91 (t, *J* = 7.3 Hz, 1H), 3.10 (ddd, *J* = 14.6, 8.1, 6.5 Hz, 1H), 2.69 – 2.49 (m, 4H). **^{13}C NMR (101 MHz, $CDCl_3$)** δ = 144.4 , 142.2, 141.2, 139.7, 129.8, 129.0, 128.5, 128.3, 127.7, 127.4, 127.4, 127.3, 125.5, 118.6, 42.9, 35.4, 24.5. The spectroscopic data was in agreement to those previously reported.[32]

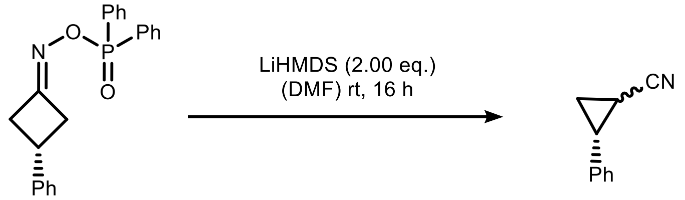

6a
95:5 er

11a
45%, 62:38 dr (trans:cis)
74:26 er (53% es)

Following a modified procedure by Shuai *et al.*[33], in a round bottom flask, oxime ester [**6a**] (36.1 mg, 100 µmol, 1.00 eq.) was dissolved in dry DMF (2.0 mL). LiHMDS in THF (1.0 M, 0.20 mL, 200 µmol, 2.00 eq.) was added and the reaction mixture was stirred at room temperature for 16 h. A sat. aqu. NH_4Cl solution was added and the mixture was diluted with EtOAc. The phases were separated and the organic phase was dried over $MgSO_4$, filtered and concentrated under reduced pressure. The diastereomeric ratio (62:38 dr, trans:cis) was determined *via* crude 1H NMR analysis using CH_2Br_2 as internal standard. The crude mixture was purified *via* column chromatography (SiO_2, pentane:EtOAc, 100:0 → 90:10, stained with CAM) to obtain [*trans*-**11a**] (4.0 mg, 27.9 µmol, 28%), and [*cis*-**11a**] (2.5 mg, 17.5 µmol, 17%).

Major diastereomer (*trans*-**11a**): **^{1}H NMR (400 MHz, $CDCl_3$)** δ = 7.35 – 7.29 (m, 2H), 7.29 – 7.23 (m, 1H), 7.14 – 7.08 (m, 2H), 2.63 (ddd, J = 9.2, 6.7, 4.7 Hz, 1H), 1.62 (*app.* dt, J ≈ 9.1, 5.2 Hz, 1H), 1.55 (ddd, J = 8.6, 5.4, 4.8 Hz, 1H), 1.45 (ddd, J = 8.6, 6.7, 5.0 Hz, 1H). **^{13}C NMR (101 MHz, $CDCl_3$)** δ = 137.7, 128.9, 127.5, 126.4, 121.2, 25.0, 15.3, 6.7. Spectroscopic data was in agreement to those previously reported.[34] Enantiomerically enriched sample of 74:26 er. The enantiomeric purity was established by HPLC analysis using a chiral column (Lux® Cellulose-1, 40 °C, 1 mL/min, 95:05

*n*hexane:isopropanol, 210 nm, t = 12.559 min and 13.604 min). The enantiospecificity (es) of the reaction therefore is 53%.[h]

Minor diastereomer (*cis*-**11a**): **^{1}H NMR (400 MHz, $CDCl_3$)** *δ* = 7.40 – 7.34 (m, 2H), 7.33 – 7.26 (m, 3H), 2.54 (td, J = 8.4, 7.1 Hz, 1H), 1.84 (ddd, J = 8.9, 8.3, 5.7 Hz, 1H), 1.60 – 1.48 (m, 2H). **^{13}C NMR (101 MHz, $CDCl_3$)** *δ* = 135.2, 128.6, 128.1, 127.7, 119.5, 23.2, 12.9, 6.4. Spectroscopic data was in agreement to those previously reported.[35] Enantiomerically enriched sample of 74:26 er. The enantiomeric purity was established by HPLC analysis using a chiral column (Lux® Cellulose-1, 40 °C, 1 mL/min, 95:05 *n*hexane:isopropanol, 210 nm, t = 12.559 min and 13.604 min).

---

[h] Enantiospecificity (es) is determined by the following formula: es = enantiomeric excess (ee) of product/ ee of starting material) * 100%, whereas ee = ((percentage of major enantiomer – percentage of minor enantiomer)/100) * 100%. The enantiospecificity (es) indicates the percentage that proceed via the enantiospecific reaction pathway.

7. Optimization of the ring contraction reaction

Table S6: Solvent and base optimization, reactions were carried out on a 0.1 mmol scale, yield based on ^{1}H NMR experiments using CH_2Br_2 as an internal standard.

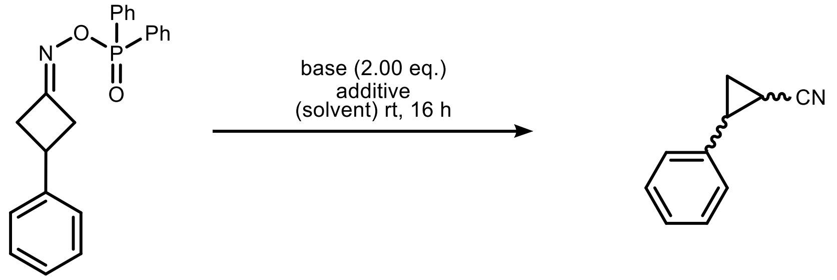

Entry	solvent	base	additives	yield	dr (trans:cis)
1	DMF	LiHMDS	-	51%	66:34
2	PhMe	LiHMDS	-	62%	71:29
3	MeCN	LiHMDS	-	0%	-
4	THF	LiHMDS	-	72%	67:34
5	cyclohexane	LiHMDS	-	<10%	n.d.
6	benzene	LiHMDS	-	32%	75:25
7	DMF	NaHMDS	-	41%	63:37
8	DMF	KHMDS	-	39%	63:37
9	DMF	*n*-BuLi	-	0%	-

rt = room temperature, DMF = *N*,*N*-dimethylformamide, HMDS = bis(trimethylsilyl)amide, $NiBr_2$ diglyme = nickel(II) bromide 2-methoxyethyl ether, complex, dtbbpy = 4,4′-di-tert-butyl-2,2′-dipyridyl, PhMe = toluene, MeCN = acetonitrile, THF = tetrahydrofurane, *n*-BuLi = *n*-butyllithium.

Table S7: Base and temperature optimisation, reactions were carried out on a 0.1 mmol scale, yield based on ^{19}F NMR experiments using $PhCF_3$ as an internal standard.

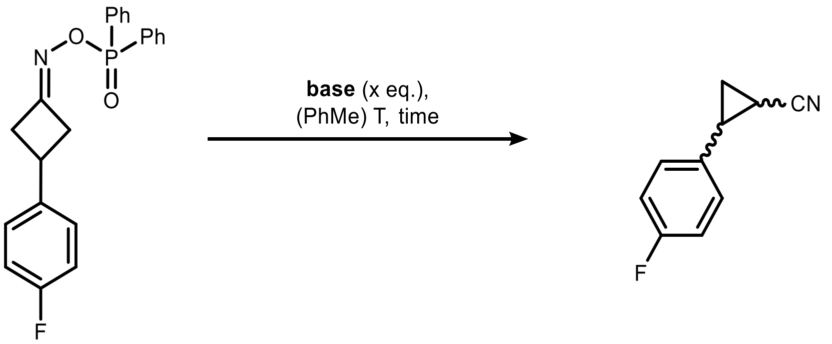

Entry	base	base (x eq.)	time	temperature	yield	dr (trans:cis)
1	LiHMDS	2.00	1 h	rt	53%	75:25
2	LiHMDS	1.00	1 h	rt	45%	5:95
3	NaHMDS	2.00	1 h	rt	22%	72:28
4	NaHMDS, 18-crown-6	2.00	1 h	rt	37%	23:77
5	KHMDS	2.00	1 h	rt	36%	78:22
6	LDA	2.00	1 h	rt	34%	34:66
7	LiTMP	2.00	1 h	rt	0%	-
8	KOtBu	2.00	1 h	rt	10%	8:92
9	KOtPent	2.00	1 h	rt	44%	59:41
10	LiHMDS	2.00	1 h	0 °C	58%	77:23
11	LiHMDS	2.00	1 h	–20 °C	60%	76:24
12	LiHMDS	2.00	1 h	–78 °C	62%	9:91
13	LiHMDS	2.00	0.5 h	–78 °C	38%	11:89

PhMe = toluene, HMDS = bis(trimethylsilyl)amide, LDA = lithium diisopropylamide, LiTMP = lithium tetramethylpiperide, KO*t*Bu = potassium *tert*-butoxide, KO*t*Pent = potassium *tert*-pentyloxide, Ph = Phenyl, Mes = mesityl, rt = room temperature, DMF = *N*,*N*-dimethylformamide, THF = tetrahydrofurane, TFE = 2,2,2-trifluoroethanol, HFIP = 1,1,1,3,3,3-hexafluoroisopropanol

Table S8: Reaction time and equivalents of base optimisation, reactions were carried out on a 0.1 mmol scale, yield based on ^{19}F NMR experiments using $PhCF_3$ as an internal standard.

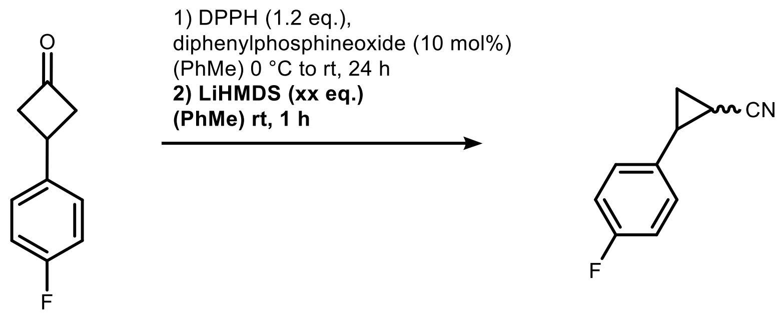

Entry	time	base (x eq.)	yield	dr (trans:cis)
1	16 h	2.00	53%	75:25
2	3 h	2.00	51%	76:24
3	1 h	2.00	52%	76:24
4	30 min	2.00	54%	76:24
5	5 min	2.00	53%	76:24
6	16 h	0.50	39%	10:90
7	16 h	1.00	38%	>5:95
8	16 h	1.50	37%	14:86
9	16 h	3.00	39%	78:22

DPPH = *O*-Diphenylphosphinylhydroxylamine, PhMe = toluene, LiHMDS = lithium bis(trimethylsilyl)amide.

Table S9: Solvent optimisation, reactions were carried out on a 0.1 mmol scale, yield based on ^{1}H NMR experiments using CH_2Br_2 as an internal standard.

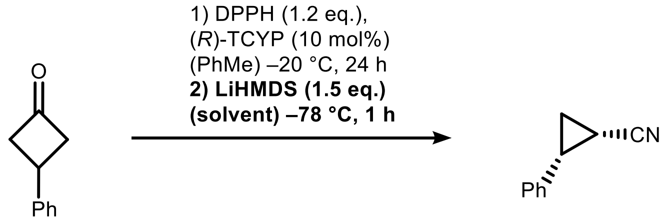

Entry	base	solvent reaction	yield	er (cis)
1	LiHMDS in PhMe	PhMe	56%	60:40
2	LiHMDS in THF	PhMe	56%	90:10
3	LiHMDS in THF	THF	12%	85:15
4	LiHMDS in THF	PhMe:THF (1:1)	45%	87:13

DPPH = *O*-Diphenylphosphinylhydroxylamine, (*R*)-TCYP = (*R*)-4-Hydroxy-2,6-bis(2,4,6-tricyclohexylphenyl)dinaphtho[2,1-d:1',2'-f][1,3,2]dioxaphosphepine 4-oxide, PhMe = toluene, LiHMDS = lithium bis(trimethylsilyl)amide.

8. Mechanistic Investigation for the Ring Contraction Reaction

In the following, different pathways plausible for the ring contraction rearrangement will be discussed. Optimization revealed the solvent mixture of toluene and THF deemed to be crucial to obtain high stereospecificity during the transformation. LiHMDS is known to undergo solvent dependent aggregation. Whereas oligomeric structures are predominant in noncoordinating solvents like toluene, degradation occurs in ethereal solution generating mono or dimeric LiHMDS.[36] Based on these observations, we propose the occurrence of low aggregated LiHMDS is essential enabling deprotonation directed by the diphenyl phosphinyl group (for diastereoselectivity see DFT). This regioselectivity directly corresponds to the enantiospecificity of the process. From anion **10a** three different mechanistic pathways are conceivable. Pathway A is based on a Neber-type rearrangement, including the formation of nitrene intermediate **S20**. The product *cis*-**11a** is formed *via* a 1,2-shift of zwitterionic intermediate **S21**. A concerted rearrangement is described by the cleavage of the weak N,O-bond and simultaneous cleavage of the C,C-bond by attack of the anion (pathway B). The third mechanistic proposal includes a [3,3]-sigmatropic rearrangement cleaving the weak N,O-bond and forming *α*-oxyphosphinylated intermediate **S23.** Similar processes have been described by Tomkinson and coworkers.[37] Subsequently, a Favorskii-type rearrangement may explain the formation of the cyclopropane nitrile based on the good leaving group ability of the phosphinyl group.

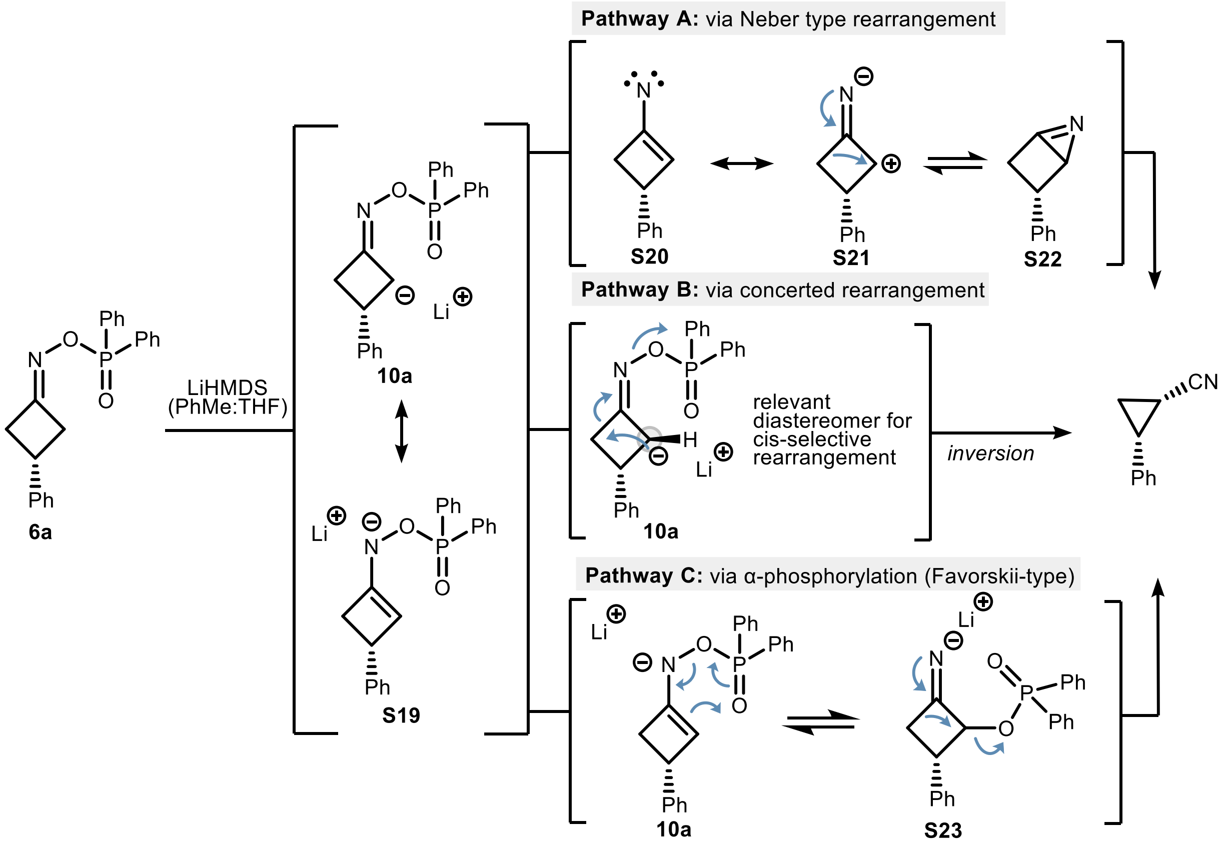

Scheme S2. Mechanistic proposals for the ring contraction reaction of cyclobutanone oxime ester towards cyclopropane nitriles.

Control Experiments

Different control experiments were conducted to identify the most likely mechanistic proposal. Treatment of cyclohexanone-derived oxime ester *rac*-**13** under identical conditions afforded pyrazine *rac*-**15**. Formation of this product can be explained by self-condensation of α-amino ketone *rac*-**14** generated *via* Neber reaction followed by aromatization through oxidation. The formation of *α*-amino ketone being involved in the process was confirmed by generation of the corresponding hydrochloride *HCl·rac*-**14** and analysis *via* MS. It should be noted that reactions of substrates with different ring size do not necessarily have to follow the same mechanistic pathway. However, a related initial deprotonation in accordance with Neber reaction of *rac*-**13** can be assumed for **6a**. Ring contraction rearrangement was also observed for oxime ether **S13** (vide infra). This observation suggests pathway C to be less likely and underlines the effect of the leaving group on the ring contraction. To shine further light on the distinction between pathway A and B, DFT calculations were performed (vide infra). Our study reveals a plausible transition state energy for a concerted rearrangement pathway without further intermediates such as, e.g., nitrene **S20**., lending proof to pathway B. In addition, the DFT calculations reveal **TS3** THF as the only reasonable TS occurring from the cis-diastereomer of **10a** (Li and Phenyl are cis). This also supports pathway B, which explains the experimentally observed high diastereoselectivity via inversion, which is only possible in a concerted pathway.

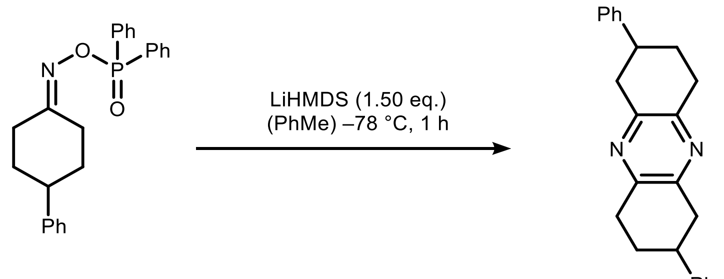

rac-**13** *rac*-**15** 38%

In a Schlenk flask, diphenyl(((4-phenylcyclohexylidene)amino)oxy)phosphine oxide [*rac*-**14**] (38.9 mg, 0.10 mmol, 1.00 eq.) was dissolved in dry toluene (1.0 mL) under N_2-atmosphere. 4 Å Molecular sieves was added and the mixture was cooled to –78 °C. LiHMDS in THF (1.0 M, 0.15 mmol, 1.5 eq.) was added and the solution was stirred for 1 h. Water was added, and the aqueous phase was extracted with CH_2Cl_2 (3 x 10 mL). The combined organic phases were dried over $MgSO_4$, filtered and concentrated under reduced pressure. The crude mixture was purified *via* column chromatography, to obtain 2,7-diphenyl-1,2,3,4,6,7,8,9-octahydrophenazine [*rac*-**15**] (13.0 mg, 0.38 mmol, 38%) as a slightly yellow solid.

M.P.: 232 – 238 °C. **IR (neat)**: $\tilde{\nu}$ = 2925 (m), 2853 (w), 1725 (w), 1494 (m), 1454 (m), 1434 (m), 1397 (s), 1158 (m), 759 (m), 700 (s), 532 (w). **^{1}H NMR (400 MHz, $CDCl_3$)** δ = 7.41 – 7.33 (m, 5H, $CH_{arom.}$), 7.32 – 7.22 (m, 5H, $CH_{arom.}$), 3.34 – 2.96 (m, 10H, CH_2, C*H*), 2.34 – 2.21 (m, 2H, CH_2), 2.12 – 1.97 (m, 2H, CH_2). **^{13}C NMR (101 MHz, $CDCl_3$)** δ = 149.2 (C_q), 145.1 (C_q), 128.8 (*C*H), 126.9 (*C*H), 126.7 (*C*H), 40.1 (*C*H), 39.1 (CH_2), 31.4 (CH_2), 30.2 (CH_2). **HRMS (ESI):** Calculated for $C_{24}H_{25}N_2$ $[M+H]^+$: 342.2012, Found: 342.2009.

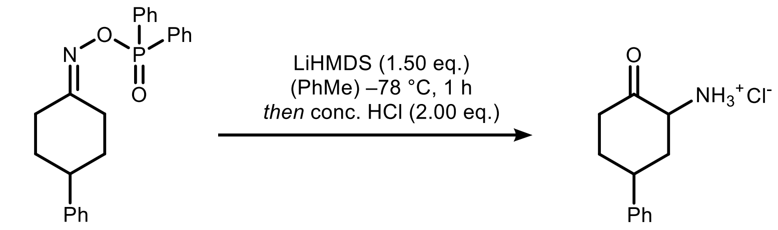

rac-**13** *HCl·rac*-**14**

In a Schlenk flask, diphenyl(((4-phenylcyclohexylidene)amino)oxy)phosphine oxide [*rac*-**13**] (38.9 mg, 100 µmol, 1.00 eq.) was dissolved in dry toluene (1.0 mL) under N_2-atmosphere. 4 Å Molecular sieves was added and the mixture was cooled to −78 °C. LiHMDS in THF (1.0 M, 150 µmol, 1.50 eq.) was added and the solution was stirred for 1 h. Conc. HCl (6.8 µL, 200 µmol, 2.00 eq.) was added and the reaction mixture was stirred for 30 minutes. Water was added and the phases were separated. The aqueous phase was concentrated under reduced pressure. The crude material was analyzed by HRMS (ESI) (Calculated for $C_{12}H_{16}NO$ $[M]^+$: 190.1226, Found: 190.1221). The yield was determined, as purification form other salts and side products was not conducted.

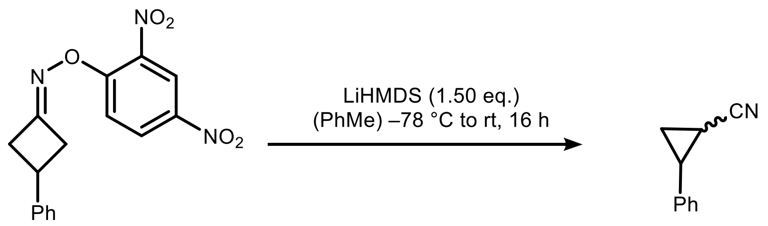

S13

11a
32%, 77:28 trans:cis

In a Schlenk flask, 3-phenylcyclobutan-1-one *O*-(2,4-dinitrophenyl) oxime [**S13**] (32.7 mg, 100 µmol, 1.00 eq.) was dissolved in dry toluene (1.0 mL) under N_2-atmosphere. 4 Å Molecular sieves was added and the mixture was cooled to −78 °C. LiHMDS in THF (1.0 M, 150 µmol, 1.50 eq.) was added and the solution was allowed to warm to room temperature over night. The mixture was filtered over a short plug of silica and the solvent was removed under reduced pressure. By ^{1}H NMR analysis of the crude reaction mixture trans and cis cyclopropane formation (32%) were observed with a diastereomeric ratio of (72:28 trans:cis).

DFT calculations for the formation of *cis*-11a:

We determined the preferred conformers of **10a** and **10a·THF** with CREST/GFN2-xTB + CENSO.[38] Both showed a *cis* configuration of Li and Ph in the cyclobutyl ring (Figure S4). Based on the minimum structures, we optimized the transition structures **TS3** and **TS3·THF**, which both show an elongated C-C bond in the cyclobutyl ring and a broken N-O bond (Figure S5). An IRC calculation of **TS3** proved that it connects **10a** with a complex of the *cis*-**11a**.

Attempts to optimize the structure of nitrene **12a** lead to the structure of the product **11a**, while a triplet nitrene (^{3}**12a**) is an (exotermic) minimum after elimination of LiO_2PPh_2. We do, however, assume that such a biradicaloid species would give rise to a broader product spectrum.

Table S10: Relative energies of molecular species in the reaction of 10a (Scheme 4), as calculated with DFT.[a] $\Delta G(T)_{solv} = \Delta E(\text{PW6B95-D3//TPSS-D3}) + \Delta G^{RRHO}_{(T)} + \Delta G^{solv}_{(T)}$

Species	E(TPSS-D3) [E_h]	G^{RRHO}_{298} [kcal/mol]	G^{RRHO}_{195} [kcal/mol]	E(PW6B95-D3) [E_h]	G^{solv}_{298} (CH_3Ph) [kcal/mol]	G^{solv}_{195} (CH_3Ph) [kcal/mol]	**$\Delta G(195)_{solv}$ (CH_3Ph)[b] [kcal/mol]**
**10a**	-1404.583558	187.171	201.180	-1405.994364	-18.959	-19.393	0.0
**THF**	-232.588006	54.454	61.394	-232.825536	-1.078	-0.018	
**10a**·THF	-1637.207518	257.002	272.952	-1638.857066	-20.472	-19.132	-12.7
**TS3**	-1404.559810	184.386	198.695	-1405.962320	-19.891	-20.933	16.1
**TS3**·THF	-1637.180220	253.181	269.697	-1638.818714	-20.776	-19.878	7.4
cis-**11a**	-441.457360	78.199	87.013	-441.938486	-7.031	-7.271	-79.8
trans-**11a**	-441.458441	78.022	86.880	-441.939690	-6.818	-6.990	-80.4
**^{3}12a**	-441.368517	76.070	84.875	-441.841671	-5.880	-5.599	-19.5
LiO_2PPh_2·THF	-1195.823086	162.595	175.469	-1197.001339	-16.058	-16.555	

[a] all calculations were performed with the def2-TZVP basis set

[b] relative free energies refer to isolated reactants (**10a** + **THF**)

Figure S4 DFT-optimized (TPSS-D3/def2-TZVP) structures of 10a and 10a·THF. Bond distances in Å. Element colors: gray (C), red (O), blue (N), white (H), yellow (P), purple (Li).

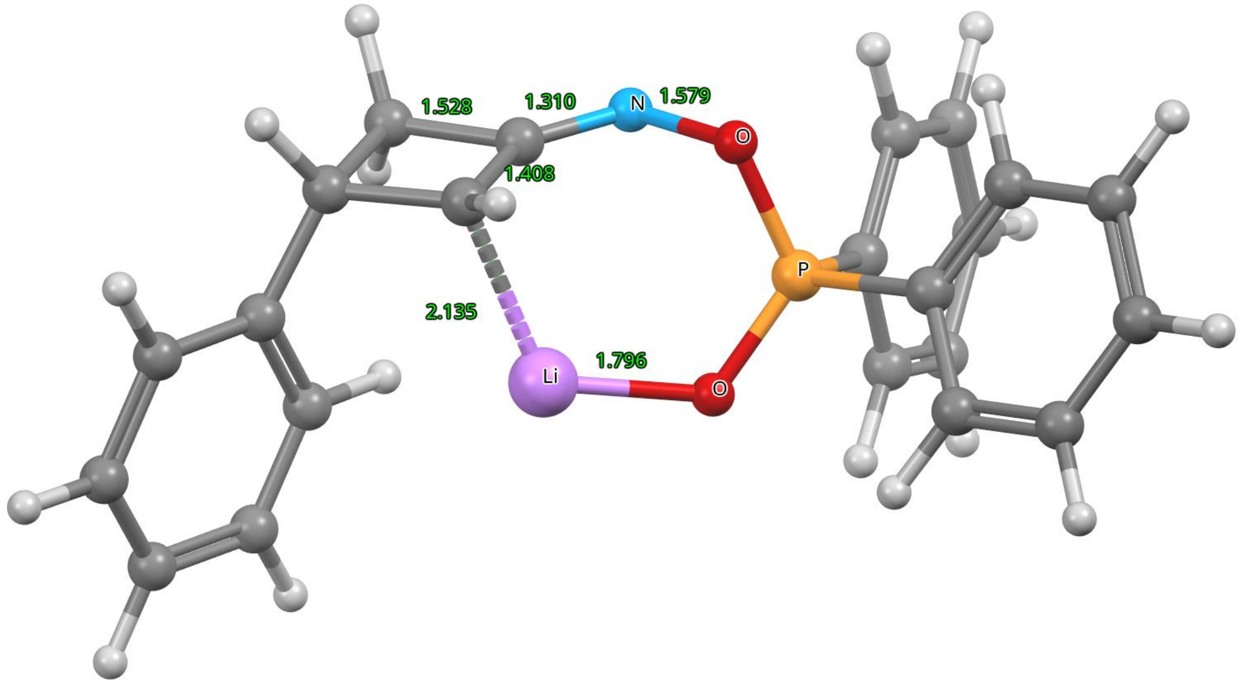

10a

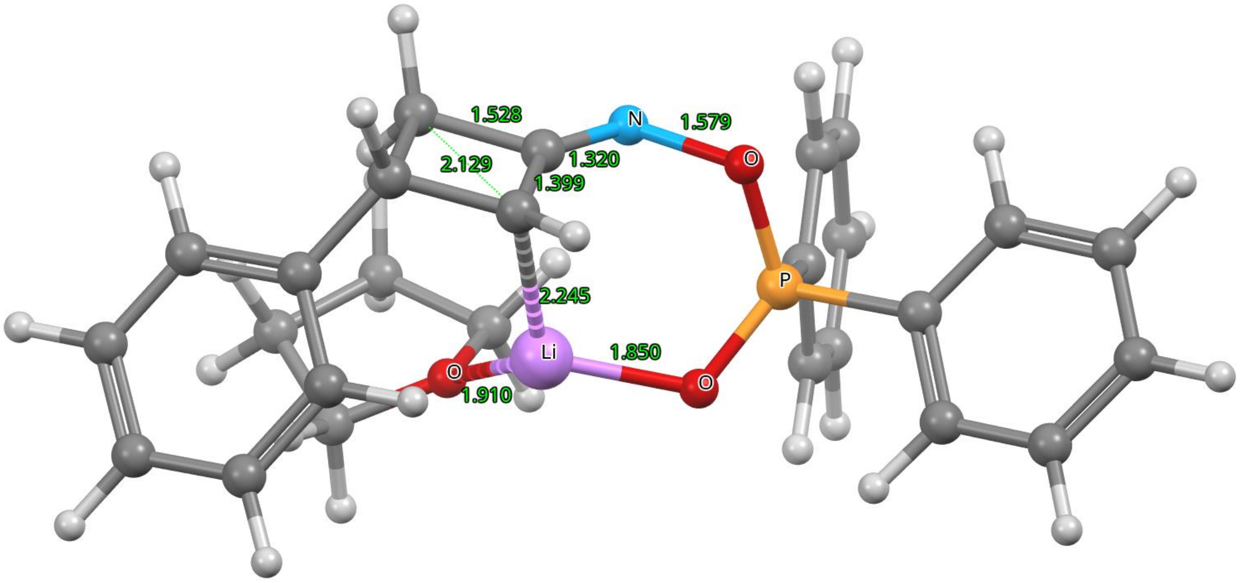

10a·THF

Figure S5 DFT-optimized (TPSS-D3/def2-TZVP) structures of TS3 and TS3·THF. Bond distances in Å. Element colors: gray (C), red (O), blue (N), white (H), yellow (P), purple (Li).

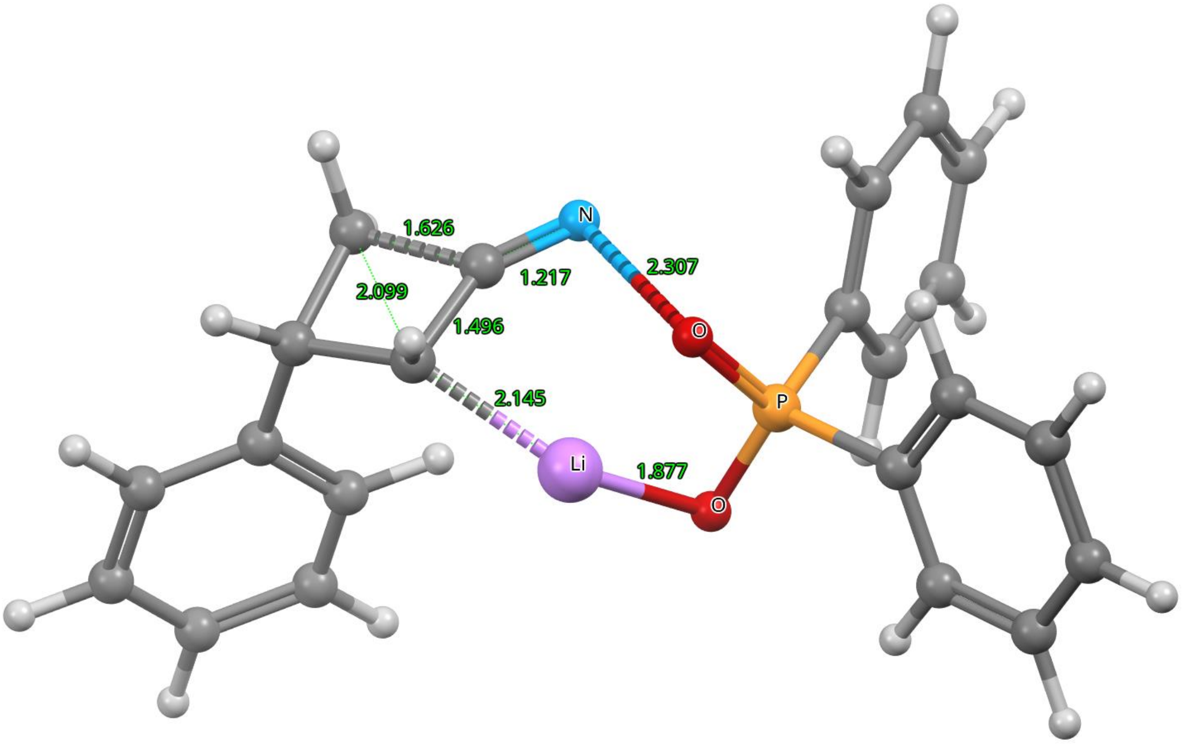

TS3

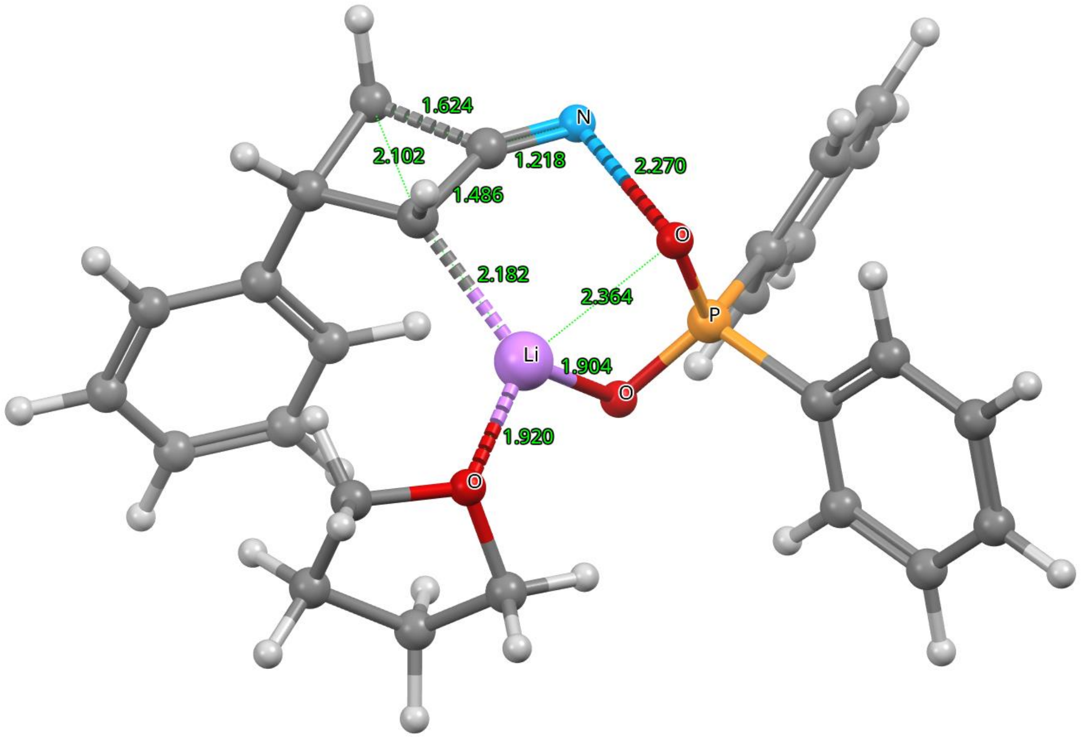

TS3·THF

9. Substrate Scope Cyclopropanes

The racemic products were prepared according to the following procedures:

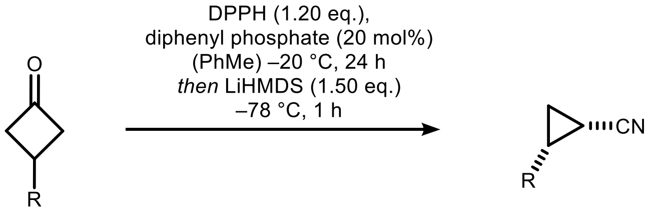

In a round bottom flask, diphenyl phosphate (20 mol%) and cyclobutanone (200 µmol, 1.00 eq.) was dissolved in toluene (0.05 M). The mixture was cooled to 0 °C. *O*-Diphenylphosphinylhydroxylamine (220 µmol, 1.10 eq.) was added and the reaction mixture was allowed to warm to room temperature over night. After cooling to –78 °C, LiHMDS in THF (1.0 M, 300 µmol, 1.50 eq.) was added and the solution was stirred for 1 h. The mixture was directly purified *via* column chromatography with the conditions given in the corresponding entry.

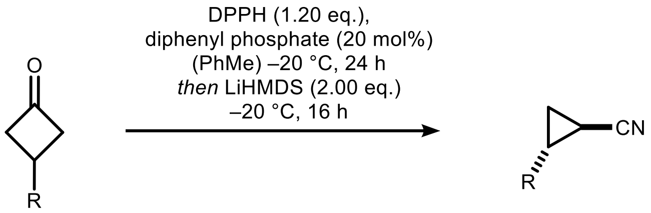

In a round bottom flask, diphenyl phosphate (20 mol%) and cyclobutanone (200 µmol, 1.00 eq.) was dissolved in toluene (0.05 M). The mixture was cooled to 0 °C. *O*-Diphenylphosphinylhydroxylamine (220 µmol, 1.10 eq.) was added and the reaction mixture was allowed to warm to room temperature overnight. After cooling to –20 °C, LiHMDS in THF (1.0 M, 400 µmol, 2.00 eq.) was added and the solution was stirred for 16 h. The mixture was directly purified *via* column chromatography with the conditions given in the corresponding entry.

General procedure K (GP-K) for the one pot synthesis of cis cyclopropanes:

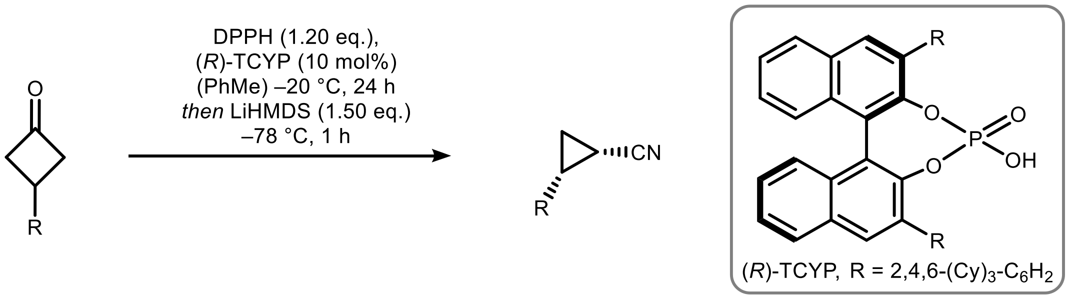

In a Schlenk flask, (*R*)-TCYP (10 mol%) and cyclobutanone (200 µmol, 1.00 eq.) was dissolved in dry toluene (0.05 M) under N_2-atmosphere. Activated 4 Å molecular sieves was added and the mixture was cooled to –20 °C. *O*-Diphenylphosphinylhydroxylamine (220 µmol, 1.10 eq.) was added and the reaction mixture was stirred at –20 °C for 24 h. After cooling to –78 °C, LiHMDS in THF (1.0 M, 300 µmol, 1.50 eq.) was added and the

solution was stirred for 1 h. The mixture was directly purified *via* column chromatography with the conditions given in the corresponding entry.

General procedure L (GP-L) for the one pot synthesis of trans cyclopropanes:

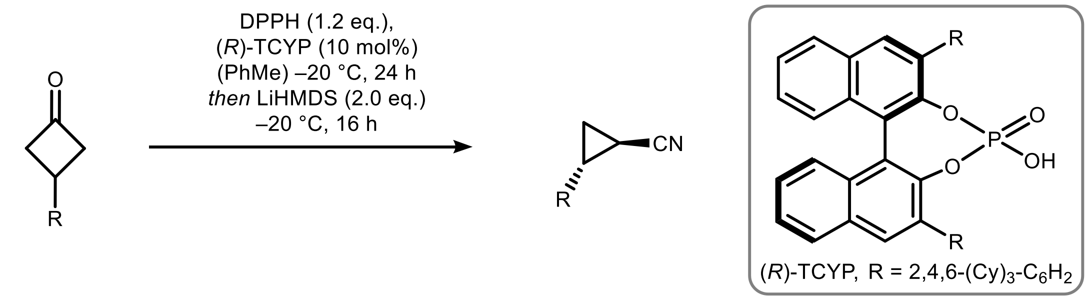

In a Schlenk flask, (*R*)-TCYP (10 mol%) and cyclobutanone (200 µmol, 1.00 eq.) was dissolved in dry toluene (0.05 M) under N_2-atmosphere. 4 Å molecular sieves was added and the mixture was cooled to –20 °C. *O*-Diphenylphosphinylhydroxylamine (220 µmol, 1.10 eq.) was added and the reaction mixture was stirred at –20 °C for 24 h. LiHMDS in THF (1.0 M, 400 µmol, 2.00 eq.) was added and the solution was stirred for 16 h. The mixture was directly purified *via* column chromatography with the conditions given in the corresponding entry.

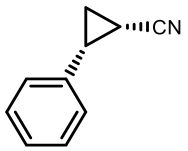

(1*S*,2*R*)-2-phenylcyclopropane-1-carbonitrile [*cis*-11a]

Following **GP-K** using 3-phenylcyclobutanone [**1a**] (29.2 mg, 200 µmol, 1.00 eq.), (*R*)-TCYP [**7e**] (19.9 mg, 20 µmol, 10 mol%) and LiHMDS (1.0 M in THF, 0.3 mL, 300 µmol, 2.00 eq.) the product (16.0 mg, 112 µmol, 56%) was obtained *via* flash column chromatography (SiO_2, pentane:EtOAc, 100:0 → 90:10, stained with CAM) as colorless solid. Crude NMR using dibromomethane as internal standard showed 62% yield and 91:9 cis:trans.

^{1}H NMR (400 MHz, $CDCl_3$) δ = 7.40 – 7.34 (m, 2H), 7.33 – 7.26 (m, 3H), 2.54 (td, J = 8.4, 7.1 Hz, 1H), 1.84 (ddd, J = 8.9, 8.3, 5.7 Hz, 1H), 1.60 – 1.48 (m, 2H). **^{13}C NMR (101 MHz, $CDCl_3$)** δ = 135.2, 128.6, 128.1, 127.7, 119.5, 23.2, 12.9, 6.4. Spectroscopic data was in agreement to those previously reported.[35] **Optical Rotation:** $[\alpha]_D^{25}$ = +15.2 (c = 0.5, $CHCl_3$) for an enantiomerically enriched sample of 90:10 er.[i] The enantiomeric purity was established by HPLC analysis using a chiral column (Lux® Cellulose-1, 40 °C, 1 mL/min, 95:05 *n*hexane:isopropanol, 214 nm, t = 12.559 min and 13.604 min).

---

[i] We noticed that the er may vary within +/- 3% for the rearrangement. 90:10 is the average over ~20 runs.

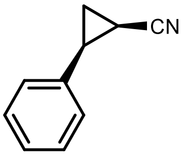

(1*R*,2*S*)-2-phenylcyclopropane-1-carbonitrile [*ent-cis*-11a]

Following **GP-K** using 3-phenylcyclobutanone [**1a**] (29.2 mg, 200 µmol, 1.00 eq.), (*S*)-TCYP [*ent*-**7e**] (19.9 mg, 20 µmol, 10 mol%) and LiHMDS (1.0 M in THF, 0.3 mL, 300 µmol, 2.00 eq.) the product (16.0 mg, 112 µmol, 56%) was obtained *via* flash column chromatography (SiO_2, pentane:EtOAc, 100:0 → 90:10, stained with CAM) as colorless solid.

^{1}H NMR (400 MHz, $CDCl_3$) δ = 7.40 – 7.34 (m, 2H), 7.33 – 7.26 (m, 3H), 2.54 (td, J = 8.4, 7.1 Hz, 1H), 1.84 (ddd, J = 8.9, 8.3, 5.7 Hz, 1H), 1.60 – 1.48 (m, 2H). **^{13}C NMR (101 MHz, $CDCl_3$)** δ = 135.2, 128.6, 128.1, 127.7, 119.5, 23.2, 12.9, 6.4. Spectroscopic data was in agreement to those previously reported.[35] **Optical Rotation:** $[\alpha]_D^{25}$ = –7.1 (c = 0.5, $CHCl_3$) for an enantiomerically enriched sample of 11:89 er. The enantiomeric purity was established by HPLC analysis using a chiral column (Lux® Cellulose-1, 40 °C, 1 mL/min, 95:05 *n*hexane:isopropanol, 214 nm, t = 12.318 min and 13.306 min).

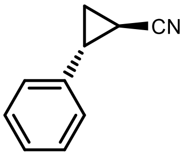

(1*R*,2*R*)-2-phenylcyclopropane-1-carbonitrile [trans-11a]

Following **GP-L** using 3-phenylcyclobutanone [**1a**] (29.2 mg, 200 µmol, 1.00 eq.), (*R*)-TCYP [**7e**] (19.9 mg, 20 µmol, 10 mol%) and LiHMDS (1.0 M in THF, 0.4 mL, 400 µmol, 2.00 eq.) the product (13.8 mg, 96 µmol, 48%) was obtained *via* flash column chromatography (SiO_2, n-hexane:EtOAc, 100:0 → 90:10, stained with CAM) as colorless solid. Crude NMR using dibromomethane as internal standard showed 63% yield and 26:74 cis:trans.

^{1}H NMR (400 MHz, $CDCl_3$) δ = 7.35 – 7.29 (m, 2H), 7.29 – 7.23 (m, 1H), 7.14 – 7.08 (m, 2H), 2.63 (ddd, J = 9.2, 6.7, 4.7 Hz, 1H), 1.62 (*app.* dt, J ≈ 9.1, 5.2 Hz, 1H), 1.55 (ddd, J = 8.6, 5.4, 4.8 Hz, 1H), 1.45 (ddd, J = 8.6, 6.7, 5.0 Hz, 1H). **^{13}C NMR (101 MHz, $CDCl_3$)** δ = 137.7, 128.9, 127.5, 126.4, 121.2, 25.0, 15.3, 6.7. Spectroscopic data was in agreement to those previously reported.[34] **Optical Rotation:** $[\alpha]_D^{25}$ = –223.5 (c = 0.5, $CHCl_3$) for an enantiomerically enriched sample of 10:90 er. The enantiomeric purity was established by HPLC analysis using a chiral column (Lux® Cellulose-1, 40 °C, 1 mL/min, 95:05 *n*hexane:isopropanol, 214 nm, t = 10.869 min and 12.470 min).

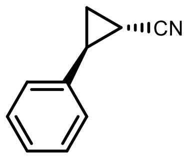

(1*S*,2*S*)-2-phenylcyclopropane-1-carbonitrile [*ent*-*trans*-11a]

Following **GP-L** using 3-phenylcyclobutanone [**1a**] (29.2 mg, 200 µmol, 1.00 eq.), (*S*)-TCYP [*ent*-**7e**] (19.9 mg, 20 µmol, 10 mol%) and LiHMDS (1.0 M in THF, 0.4 mL, 400 µmol, 2.00 eq.) the product (12.5 mg, 88 µmol, 44%) was obtained *via* flash column chromatography (SiO_2, n-hexane:EtOAc, 100:0 → 90:10, stained with CAM) as colorless solid.

^{1}H NMR (400 MHz, $CDCl_3$) δ = 7.35 – 7.29 (m, 2H), 7.29 – 7.23 (m, 1H), 7.14 – 7.08 (m, 2H), 2.63 (ddd, J = 9.2, 6.7, 4.7 Hz, 1H), 1.62 (*app.* dt, J ≈ 9.1, 5.2 Hz, 1H), 1.55 (ddd, J = 8.6, 5.4, 4.8 Hz, 1H), 1.45 (ddd, J = 8.6, 6.7, 5.0 Hz, 1H). **^{13}C NMR (101 MHz, $CDCl_3$)** δ = 137.7, 128.9, 127.5, 126.4, 121.2, 25.0, 15.3, 6.7. Spectroscopic data was in agreement to those previously reported.[34] **Optical Rotation:** $[\alpha]_D^{25}$ = +252.9 (c = 0.5, $CHCl_3$) for an enantiomerically enriched sample of 94:6 er. The enantiomeric purity was established by HPLC analysis using a chiral column (Lux® Cellulose-1, 40 °C, 1 mL/min, 95:05 *n*hexane:isopropanol, 214 nm, t = 11.187 min and 13.035 min).

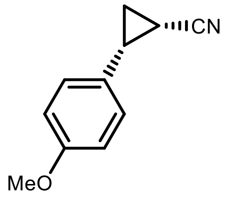

(1*S*,2*R*)-2-(4-methoxyphenyl)cyclopropane-1-carbonitrile [*cis*-11c]

Following **GP-K** using 3-(4-methoxyphenyl)cyclobutan-1-one [**1b**] (35.2 mg, 200 µmol, 1.00 eq.), (*R*)-TCYP [**7e**] (19.9 mg, 20 µmol, 10 mol%) and LiHMDS (1.0 M in THF, 0.3 mL, 300 µmol, 2.00 eq.) the product (16.2 mg, 94.1 µmol, 47%) was obtained *via* flash column chromatography (SiO_2, pentane:EtOAc, 100:0 → 90:10, stained with CAM) as colorless solid. Crude NMR using dibromomethane as internal standard showed 57% yield and 92:8 cis:trans.

^{1}H NMR (400 MHz, $CDCl_3$) δ = 7.23 – 7.17 (m, 2H), 6.93 – 6.86 (m, 2H), 3.80 (s, 3H), 2.50 (q, J = 8.1 Hz, 1H), 1.79 (td, J = 8.0, 6.6 Hz, 1H), 1.53 – 1.46 (m, 2H). **^{13}C NMR (101 MHz, $CDCl_3$)** δ = 159.2, 129.4, 127.3, 119.8, 114.2, 55.4, 22.7, 13.0, 6.27. Spectroscopic data was in agreement to those previously reported.[39] **Optical Rotation:** $[\alpha]_D^{25}$ = +12.7 (c = 0.5, $CHCl_3$) for an enantiomerically enriched sample of 88:12 er. The enantiomeric purity was established by HPLC analysis using a chiral column (Lux® Cellulose-1, 40 °C, 1 mL/min, 95:05 *n*hexane:isopropanol, 214 nm, t = 15.468 min and 16.439 min).

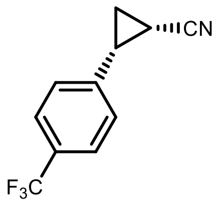

(1*S*,2*R*)-2-(4-(trifluoromethyl)phenyl)cyclopropane-1-carbonitrile [*cis*-11d]

Following **GP-K** using 3-(4-(trifluoromethyl)phenyl)cyclobutan-1-one [**1d**] (42.8 mg, 200 µmol, 1.00 eq.), (*R*)-TCYP [**7e**] (19.9 mg, 20 µmol, 10 mol%) and LiHMDS (1.0 M in THF, 0.3 mL, 300 µmol, 1.50 eq.) the product (22.1 mg, 92 µmol, 46%) was obtained *via* flash column chromatography (SiO_2, pentane:EtOAc, 100:0 → 90:10, stained with CAM) as colorless solid. Crude NMR using dibromomethane as internal standard showed 55% yield and 89:11 cis:trans.

IR (neat): $\tilde{\nu}$ = 3692 (w), 2925 (s), 2855 (m), 2363 (w), 2011 (m), 1960 (w), 1750 (m), 1326 (s), 1225 (m), 1164 (s), 1125 (s), 1069 (s), 670 (m), 650 (m), 596 (m), 582 (m), 555 (s), 527 (s), 515 (s), 504 (s), 493 (s), 482 (s), 464 (s), 446 (s), 433 (s), 413 (s). **^{1}H NMR (400 MHz, $CDCl_3$)** δ = 7.67 – 7.59 (m, 2H, C$H_{arom.}$), 7.43 – 7.36 (m, 2H, C$H_{arom.}$), 2.59 (*app.* q, $J \approx$ 8.1 Hz, 1H, C*H*), 1.93 (td, J = 8.3, 6.6 Hz, 1H, C*H*), 1.67 – 1.57 (m, 2H, CH_2). **^{13}C NMR (101 MHz, $CDCl_3$)** δ = 139.4 (C_q), 130.1 (q, $^{2}J_{C-F}$ = 32.5 Hz, C_q), 128.5 (*C*H), 125.7 (q, $^{3}J_{C-F}$ = 3.8 Hz, *C*H), 124.2 (q, $^{1}J_{C-F}$ = 272.0 Hz, C_q) 119.0 (C_q), 23.0 (*C*H), 13.3 (CH_2), 6.9 (*C*H). **^{19}F NMR (376 MHz, $CDCl_3$)** δ = –62.53. **HRMS (APCI):** Calculated for $C_{11}H_7F_3N$ $[M-H]^-$: 210.0536, Found: 210.0529. **Optical Rotation:** $[\alpha]_D^{25}$ = +43.0 (c = 0.5, $CHCl_3$) for an enantiomerically enriched sample of 13:87 er. The enantiomeric purity was established by HPLC analysis using a chiral column (Lux® Whelk-OI, 22 °C, 1 mL/min, 95:05 *n*hexane:isopropanol, 210 nm, t = 23.304 min and 30.989 min).

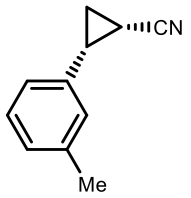

(1*S*,2*R*)-2-(*m*-tolyl)cyclopropane-1-carbonitrile [*cis*-11f]

Following **GP-K** using 3-(3-methylphenyl)cyclobutan-1-one [**1f**] (32.0 mg, 200 µmol, 1.00 eq.), (*R*)-TCYP [**7e**] (19.9 mg, 20 µmol, 10 mol%) and LiHMDS (1.0 M in THF, 0.3 mL, 300 µmol, 1.50 eq.) the product (17.2 mg, 109 µmol, 53%) was obtained *via* flash column chromatography (SiO_2, pentane:EtOAc, 100:0 → 90:10, stained with CAM) as colorless solid. Crude NMR using dibromomethane as internal standard showed 52% yield and >99:1 cis:trans.

^{1}H NMR (400 MHz, $CDCl_3$) δ = 7.29 – 7.22 (m, 1H), 7.14 – 7.05 (m, 3H), 2.51 (q, J = 8.3 Hz, 1H), 2.37 (s, 3H, CH_3), 1.82 (ddd, J = 8.9, 8.3, 5.7 Hz, 1H), 1.59 – 1.46 (m, 2H). **^{13}C NMR (101 MHz, $CDCl_3$)** δ = 138.3, 135.2, 129.0, 128.6, 128.6, 125.1, 119.6, 23.2, 21.6, 12.9, 6.4. Spectroscopic data was in agreement with those previously reported.[40] **Optical Rotation:** $[\alpha]_D^{25}$ = +13.8 (c = 0.5, $CHCl_3$) for an enantiomerically enriched sample of 90:10 er. The enantiomeric purity was established by HPLC

analysis using a chiral column (Lux® Cellulose-1, 22 °C, 1 mL/min, 95:05 *n*hexane:isopropanol, 210 nm, t = 10.936 min and 12.133 min).

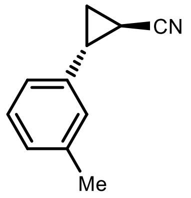

(1*R*,2*R*)-2-(m-tolyl)cyclopropane-1-carbonitrile [*trans*-11f]

Following **GP-L** using 3-(3-methylphenyl)cyclobutanone [**1f**] (32.4 mg, 200 µmol, 1.00 eq.), (*R*)-TCYP [**7e**] (19.9 mg, 20 µmol, 10 mol%) and LiHMDS (1.0 M in THF, 0.4 mL, 400 µmol, 2.00 eq.) the product (14.0 mg, 89 µmol, 45%) was obtained *via* flash column chromatography (SiO_2, n-hexane:EtOAc, 100:0 → 90:10, stained with CAM) as colorless solid. Crude NMR using dibromomethane as internal standard showed 20:80 cis:trans.

^{1}H NMR (400 MHz, $CDCl_3$) δ = 7.20 (t, J = 7.6 Hz, 1H), 7.07 (ddt, J = 7.6, 1.9, 1.0 Hz, 1H), 6.96 – 6.86 (m, 2H), 2.60 (ddd, J = 9.2, 6.7, 4.8 Hz, 1H), 2.33 (s, 3H), 1.64 – 1.50 (m, 2H), 1.44 (ddd, J = 8.5, 6.7, 4.9 Hz, 1H). **^{13}C NMR (101 MHz, $CDCl_3$)** δ = 138.7, 137.7, 128.8, 128.3, 127.3, 123.4, 121.3, 25.0, 21.5, 15.3, 6.7.[41] **Optical Rotation:** $[\alpha]_D^{25}$ = –251.6 (c = 0.33, $CHCl_3$) for an enantiomerically enriched sample of 92:8 er. The enantiomeric purity was established by HPLC analysis using a chiral column (Lux® Cellulose-1, 40 °C, 1 mL/min, 95:05 *n*hexane:isopropanol, 214 nm, t = 8.692 min and 10.456 min).

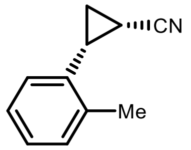

(1*S*,2*R*)-2-(*o*-tolyl)cyclopropane-1-carbonitrile [*cis*-11g]

Following **GP-K** using 3-(2-methylphenyl)cyclobutanone [**1g**] (32.0 mg, 200 µmol, 1.00 eq.), (*R*)-TCYP [**7e**] (19.9 mg, 20 µmol, 10 mol%) and LiHMDS (1 M in THF, 0.3 mL, 300 µmol, 1.50 eq.) the product (15.4 mg, 95.4 µmol, 48%) was obtained *via* flash column chromatography (SiO_2, pentane:EtOAc, 100:0 → 90:10, stained with CAM) as colorless solid. Crude NMR using dibromomethane as internal standard showed 48% yield and >99:1 cis:trans.

IR (neat): $\tilde{\nu}$ = 2953 (w), 2922 (w), 2851 (w), 2237 (m), 1492 (w), 1460 (w), 1107 (w), 1048 (w), 1034 (w), 962 (w), 839 (w), 799 (w), 768 (s), 731 (s), 637 (w), 442 (m). **^{1}H NMR (400 MHz, $CDCl_3$)** δ = 7.26 – 7.14 (m, 4H, $CH_{arom.}$), 2.48 (*app.* q, J ≈ 8.1 Hz, 1H, C*H*), 2.44 (s, 3H, CH_3), 1.90 (ddd, J = 8.8, 8.3, 5.4 Hz, 1H, C*H*), 1.61 (dt, J = 7.3, 5.4 Hz, 1H, CH_2), 1.53 (td, J = 8.6, 5.5 Hz, 1H, CH_2). **^{13}C NMR (101 MHz, $CDCl_3$)** δ = 138.6 (C_q), 133.6 (C_q), 130.4 ($CH_{arom.}$), 128.0 ($CH_{arom.}$), 127.8 ($CH_{arom.}$), 126.2 ($CH_{arom.}$), 119.6 (*C*N), 22.0 (*C*H), 19.6 (CH_3), 12.4 (CH_2), 5.4 (*C*H). **HRMS (ESI):** Calculated for $C_{11}H_{15}N_2$ $[M+NH_4]^+$: 175.1235, Found: 175.1233. **Optical Rotation:** $[\alpha]_D^{25}$ = +27.4 (c = 0.5, $CHCl_3$) for an enantiomerically enriched sample of 88:12 er. The enantiomeric

purity was established by HPLC analysis using a chiral column (Lux® Cellulose-1, 22 °C, 1 mL/min, 95:05 *n*hexane:isopropanol, 210 nm, t = 13.480 min and 14.254 min).

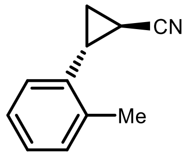

(1*R*,2*R*)-2-(o-tolyl)cyclopropane-1-carbonitrile [*trans*-11g]

Following **GP-L** using 3-(2-methylphenyl)cyclobutanone [**1g**] (30.0 mg, 180 µmol, 1.00 eq.), (*R*)-TCYP [**7e**] (18.6 mg, 19 µmol, 10 mol%) and LiHMDS (1.0 M in THF, 0.375 mL, 375 µmol, 2.00 eq.) the product (12.6 mg, 80 µmol, 43%) was obtained *via* flash column chromatography (SiO_2, n-hexane:EtOAc, 100:0 → 90:10, stained with CAM) as colorless oil. Crude NMR using dibromomethane as internal standard showed 22:78 cis:trans.

^{1}H NMR (400 MHz, $CDCl_3$) δ = 7.22 – 7.18 (m, 2H, C*H*arom.), 7.18 – 7.12 (m, 1H, C*H*arom.), 6.98 – 6.94 (m, 1H, C*H*arom.), 2.66 – 2.57 (m, 1H, C*H*), 2.46 (s, 3H, CH_3), 1.65 – 1.60 (m, 1H, CH_2), 1.49 – 1.40 (m, 2H, C*H*, CH_2). **^{13}C NMR (101 MHz, $CDCl_3$)** δ = 138.4 (*C*q), 135.8 (*C*q), 130.3 (*C*Harom.), 127.7 (*C*Harom.), 126.2 (*C*Harom.), 126.0 (*C*Harom.), 121.5 (*C*q), 23.7 (*C*H), 19.7 (*C*H_3), 14.1 (*C*H_2), 5.2 (*C*H). Spectroscopic data was in agreement to those previously reported.[41] **Optical Rotation:** $[\alpha]_D^{25}$ = –135.5 (c = 0.5, $CHCl_3$) for an enantiomerically enriched sample of 15:85 er. The enantiomeric purity was established by HPLC analysis using a chiral column (Lux® Cellulose-1, 40 °C, 1 mL/min, 95:05 *n*hexane:isopropanol, 214 nm, t = 10.225 min and 11.936 min).

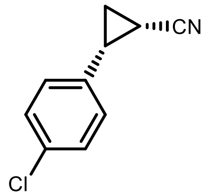

(1*S*,2*R*)-2-(4-chlorophenyl)cyclopropane-1-carbonitrile [*cis*-11i]

Following **GP-K** using 3-(4-chlorophenyl)cyclobutanone [**1i**] (36.1 mg, 200 µmol, 1.00 eq.), (*R*)-TCYP [**7e**] (19.9 mg, 20 µmol, 10 mol%) and LiHMDS (1.0 M in THF, 0.3 mL, 300 µmol, 1.50 eq.) the product (11.6 mg, 65 µmol, 33%) was obtained *via* flash column chromatography (SiO_2, n-hexane:EtOAc, 100:0 → 90:10, stained with CAM) as colorless solid. Crude NMR using dibromomethane as internal standard showed 63:37 cis:trans.

^{1}H NMR (400 MHz, $CDCl_3$) δ = 7.36 – 7.31 (m, 2H, C*H*arom.), 7.24 – 7.18 (m, 2H, C*H*arom.), 2.52 (q, J = 8.1 Hz, 1H, C*H*), 1.86 (td, J = 8.5, 6.1 Hz, 1H, C*H*), 1.60 – 1.48 (m, 2H, CH_2). **^{13}C NMR (101 MHz, $CDCl_3$)** δ = 133.9 (*C*q), 133.7 (*C*q), 129.6 (*C*H), 129.0 (*C*H), 119.3 (*C*q), 22.7 (*C*H), 13.2 (*C*H_2), 6.6 (*C*H). Spectroscopic data was in agreement to those previously reported.[39] **Optical Rotation:** $[\alpha]_D^{25}$ = +15.0 (c = 0.5, $CHCl_3$) for an enantiomerically enriched sample of 10:90 er. The enantiomeric purity was established by HPLC analysis using a chiral column (Lux® Amylose-3, 40 °C, 1 mL/min, 95:05 *n*hexane:isopropanol, 214 nm, t = 11.628 min and 12.358 min).

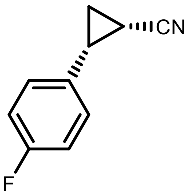

(1*S*,2*R*)-2-(4-fluorophenyl)cyclopropane-1-carbonitrile [*cis*-11k]

Following **GP-K** using 3-(4-fluorophenyl)cyclobutan-1-one [**1k**] (32.8 mg, 200 µmol, 1.00 eq.), (*R*)-TCYP [**7e**] (19.9 mg, 20 µmol, 10 mol%) and LiHMDS (1.0 M in THF, 0.3 mL, 300 µmol, 1.50 eq.) the product (17.3 mg, 106 µmol, 53%) was obtained *via* flash column chromatography (SiO_2, pentane:EtOAc, 100:0 → 90:10, stained with CAM) as colorless solid. Crude NMR using dibromomethane as internal standard showed 65% yield and 91:9 cis:trans.

IR (neat): $\tilde{\nu}$ = 2924 (w), 2854 (w), 2238 (w), 1606 (w), 1513 (s), 1451 (w), 1230 (m), 1160 (w), 1103 (w), 1015 (w), 964 (w), 836 (m), 811 (m), 765 (w), 731 (w), 614 (w), 513 (w), 483 (w). **^{1}H NMR (400 MHz, CDCl$_3$)** δ = 7.29 – 7.21 (m, 2H, C$H_{arom.}$), 7.09 – 7.01 (m, 2H, C$H_{arom.}$), 2.53 (q, J = 8.0 Hz, 1H, C*H*), 1.84 (td, J = 8.4, 6.0 Hz, 1H, C*H*), 1.58 – 1.47 (m, 2H, CH_2). **^{13}C NMR (101 MHz, CDCl$_3$)** δ = 162.4 (d, $^1J_{C\text{-}F}$ = 246.4 Hz, C_q), 131.1 (d, $^4J_{C\text{-}F}$ = 3.3 Hz, C_q), 129.9 (d, $^3J_{C\text{-}F}$ = 8.1 Hz, *C*H), 119.5 (*C*N), 115.7 (d, $^2J_{C\text{-}F}$ = 21.7 Hz, *C*H), 22.6 (*C*H), 13.2 (*C*H_2), 6.40 (*C*H). **^{19}F NMR (376 MHz, CDCl$_3$)** δ = -114.47 (tt, *J* = 8.3, 5.0 Hz). Spectroscopic data was in agreement to those previously reported.[39] **Optical Rotation:** $[\alpha]_D^{25}$ = +12.6 (c = 0.5, $CHCl_3$) for an enantiomerically enriched sample of 88:12 er. The enantiomeric purity was established by HPLC analysis using a chiral column (Lux® Cellulose-1, 22 °C, 1 mL/min, 95:05 *n*hexane:isopropanol, 210 nm, t = 17.769 min and 19.106 min).

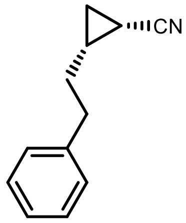

(1*S*,2*R*)-2-phenethylcyclopropane-1-carbonitrile [*cis*-11l]

Following **GP-K** using 3-phenethylcyclobutan-1-one [**1l**] (34.9 mg, 200 µmol, 1.00 eq.), (*R*)-TCYP [**7e**] (19.9 mg, 20 µmol, 10 mol%) and LiHMDS (1.0 M in THF, 0.3 mL, 300 µmol, 1.50 eq.) the product (16.1 mg, 94 µmol, 47%) was obtained *via* flash column chromatography (SiO_2, pentane:EtOAc, 100:0 → 90:10, stained with CAM) as colorless solid. Crude NMR using dibromomethane as internal standard showed 51% yield and 90:10 cis:trans.

^{1}H NMR (400 MHz, CDCl$_3$) δ = 7.33 – 7.27 (m, 2H, C$H_{arom.}$), 7.25 – 7.16 (m, 3H, C$H_{arom.}$), 2.92 – 2.72 (m, 2H, CH_2), 1.86 (dtd, J = 8.3, 7.0, 3.1 Hz, 2H, CH_2), 1.45 (ddd, J = 8.5, 8.0, 5.3 Hz, 1H, C*H*), 1.25 (ddt, J = 14.8, 7.9, 6.8 Hz, 1H, C*H*), 1.14 (td, J = 8.4, 5.0 Hz, 1H, CH_2), 0.79 (dt, J = 6.7, 5.1 Hz, 1H, CH_2). **^{13}C NMR (101 MHz, CDCl$_3$)** δ = 141.2 (C_q), 128.7 (*C*H), 128.6 (*C*H), 126.2 (*C*H), 120.7 (C_q), 35.0 (*C*H_2), 32.7 (*C*H_2), 18.2 (*C*H), 13.91 (*C*H_2), 2.7 (*C*H). Spectroscopic data was in agreement with those

previously reported.[40] **Optical Rotation:** $[\alpha]_D^{25}$ = –12.8 (c = 0.5, $CHCl_3$) for an enantiomerically enriched sample of 21:79 er. The enantiomeric purity was established by HPLC analysis using a chiral column (Lux® iAmylose-3, 40 °C, 1 mL/min, 95:05 *n*hexane:isopropanol, 214 nm, t = 7.403 min and 7.965 min).

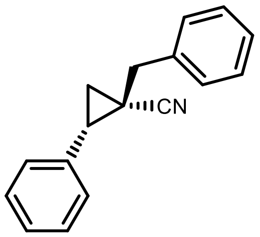

(1*R*,2S)-1-benzyl-2-phenylcyclopropane-1-carbonitrile [16]

In a Schlenk flask, (*R*)-TCYP [**7e**] (49.7 mg, 50.0 µmol, 10 mol%) and 3-phenylcyclobutanone [**1a**] (73.1 mg, 500 µmol, 1.00 eq.) was dissolved in dry toluene (4.0 mL, 0.1 M) under N_2-atmosphere. 4 Å molecular sieves was added and the mixture was cooled to –20 °C. *O*-Diphenylphosphinylhydroxylamine [**2**] (128 mg, 550 µmol, 1.10 eq.) was added and the reaction mixture was stirred at –20 °C for 24 h. LiHMDS in THF (1.0 M, 1.50 mL, 600 µmol, 3.00 eq.) was added and the solution was stirred for 1 h. Benzylbromide (85.5 mg, 0.06 mL, 500 µmol, 1.00 eq.) was added and the reaction mixture was stirred for 4 h at room temperature. Water was added and the aqueous phase was extracted with CH_2Cl_2 (3 x 20 mL). The combined organic phases were dried over $MgSO_4$, filtered and concentrated under reduced pressure. The product [**16**] (69.3 mg, 297 µmol, 59%) was obtained *via* flash column chromatography (SiO_2, cyclohexane:EtOAc, 100:0 → 90:10, stained with $KMnO_4$) as yellow oil. Crude NMR using dibromomethane as internal standard showed > 99:1 cis:trans.

IR (neat): $\tilde{\nu}$ = 3058 (w), 2234 (w), 1604 (w), 1498 (m), 1455 (w), 1077 (w), 777 (w), 734 (w), 698 (s), 474 (w), 425 (w). **^{1}H NMR (400 MHz, $CDCl_3$)** δ = 7.40 – 7.27 (m, 7H, $CH_{arom.}$), 7.20 – 7.16 (m, 2H, $CH_{arom.}$), 3.07 (d, J = 14.6 Hz, 1H, CH_2), 2.93 (d, J = 14.5 Hz, 1H, CH_2), 2.47 (dd, J = 8.8, 7.1 Hz, 1H, C*H*), 1.77 (dd, J = 7.1, 5.8 Hz, 1H, CH_2), 1.48 (dd, J = 8.8, 5.8 Hz, 1H, CH_2). **^{13}C NMR (101 MHz, $CDCl_3$)** δ = 136.9, 135.6, 129.1, 129.0, 128.7, 128.1, 127.7, 127.6, 120.9, 41.6, 30.4, 21.1, 19.3. **HRMS (ESI):** Calculated for $C_{17}H_{15}N_2$ $[M+NH_4]^+$: 251.1543, Found: 251.1543. **Optical Rotation:** $[\alpha]_D^{25}$ = –48.1 (c = 0.5, $CHCl_3$) for an enantiomerically enriched sample of 10:90 er. The enantiomeric purity was established by HPLC analysis using a chiral column (Lux® Cellulose-1, 40 °C, 1 mL/min, 95:05 *n*hexane:isopropanol, 214 nm, t = 17.027 min and 18.764 min).

The relative configuration was determined *via* ^{1}H-^{1}H NOESY experiment:

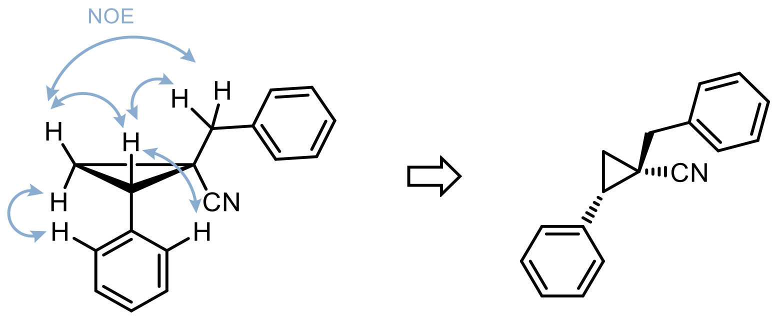

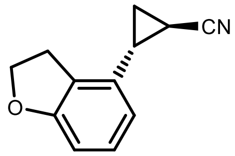

(1*R*,2*R*)-2-(2,3-dihydrobenzofuran-4-yl)cyclopropane-1-carbonitrile [*trans*-11p]

Following **GP-L** using 3-(2,3-dihydrobenzofuran-4-yl)cyclobutan-1-one [**1p**] (37.6 mg, 200 µmol, 1.00 eq.) (*R*)-TCYP [**7e**] (19.9 mg, 20 µmol, 10 mol%) and LiHMDS (1.0 M in THF, 0.4 mL, 400 µmol, 2.00 eq.) the product (20.6 mg, 112 µmol, 56%) was obtained *via* flash column chromatography (SiO_2, pentane:EtOAc, 100:0 → 90:10, stained with CAM) as colorless solid.

^{1}H NMR (400 MHz, $CDCl_3$) δ = 7.10 – 7.01 (m, 1H, $CH_{arom.}$), 6.70 (d, J = 7.9 Hz, 1H, $CH_{arom.}$), 6.38 (d, J = 7.8 Hz, 1H, $CH_{arom.}$), 4.63 (t, J = 8.7 Hz, 2H, CH_2), 3.34 – 3.25 (m, 2H, CH_2), 2.53 (ddd, J = 9.2, 6.8, 4.9 Hz, 1H, C*H*), 1.62 (dt, J = 9.1, 5.1 Hz, 1H, CH_2), 1.54 (dt, J = 8.6, 5.0 Hz, 1H, C*H*), 1.46 (ddd, J = 8.6, 6.8, 4.9 Hz, 1H, CH_2). **^{13}C NMR (101 MHz, $CDCl_3$)** δ = 160.3 (C_q), 134.3 (C_q), 128.8 (*C*H), 127.1 (C_q), 121.2 (C_q), 116.2 (*C*H), 108.7 (*C*H), 71.3 (CH_2), 28.7 (CH_2), 23.0 (*C*H), 14.6 (CH_2), 5.73 (*C*H). Spectroscopic data was in agreement with those previously reported.[42] **Optical Rotation:** $[\alpha]_D^{25}$ = –61.0 (c = 0.5, $CHCl_3$) for an enantiomerically enriched sample of 12:88 er. The enantiomeric purity was established by HPLC analysis using a chiral column (Lux® AMS, 40 °C, 1 mL/min, 95:05 *n*hexane:isopropanol, 214 nm, t = 17.147 min and 20.378 min).

10. Crystallographic data

10.1. X-ray crystal structure analysis of [6m] (MAR379)

A colorless needle like specimen of $C_{20}H_{24}NO_2P$, approximatel dimensions 0.060 x 0.110 x 0.510 mm^3, was used for the X-ray crystallographic analysis. The x-ray intensity data were measured on a STOE STADIVARI Diffractometer system. CCDC number: 2419830

Table S11: Crystal data and structure refinement for [6m] (MAR379)

Identification code	MAR379
Empirical formula	$C_{20}H_{24}NO_2P$
Moiety formula	$C_{20}H_{24}NO_2P$
Formula weight	341.37
Temperature	120(2) K
Wavelength, radiation type	0.71073Å, MoKα
Diffractometer	STOE STADIVARI
Crystal type	Monoclinic
Space group name, number	$P2_{1/c}$, (14)
Unit cell dimensions	a = 12.6992(6) Å α = 90°<br>b = 8.2618(3) Å β = 91.618(4)°<br>c = 17.7909(8)Å γ = 90°
Volume	1865.85(14) $Å^3$
Number of reflections	18382
And range used for lattice Parameters	1.60°<=Θ<=32.81°
Z	4
Density (calculated)	1.215 Mg/m^3
Absorption coefficient	0.159 mm^{-1}
Absorption correction	integration
Max. and min. transmission	0.9895 and 0.9331
F(000)	728
Crystal size, color and form	0.060 x 0.110 x 0.510 mm^3, colorless needle
Theta range for data collection	2.290 to 28.499°
Index ranges	-17<=h<=17, -11<=k=11, -23=l=23
Number of reflections:	
Collected	27382
Independent	4711 [R_{int}=0.0546]
Observed [I>2sigma(I)]	3461
Completeness of theta = 25.2°	100.0%
Refinement method	Full-matrix least-squares on F^2
Data / restraints / parameters	4711 / 70 / 265
Goodness-of-fit on F^2	1.037
Final R indices [I>2sigma(I)]	R1 = 0.0629, wR2 = 0.1739
R indices (all data)	R1 = 0.0785, wR2 = 0.1797
Largest diff. peak and hole	0.382 and -0.417 $eÅ^{-3}$

10.2. X-ray crystal structure analysis of [6h] (MAR648)

A colorless needle like specimen of $C_{22}H_{19}ClNO_2P$, approximately dimensions 0.060 x 0.080 x 0.690 mm^3, was used for the X-ray crystallographic analysis. The X-ray intensity data were measured on a STOE STADIVARI Diffractometer system. CCDC number: 2434073

Table S12. Crystal data and structure refinement for [6h] (mar648)

Identification code	MAR648
Empirical formula	$C_{22}H_{19}ClNO_2P$
Moiety formula	$C_{22}H_{19}ClNO_2P$
Formula weight	395.80
Temperature	120(2) K
Wavelength, radiation type	1.54186Å, CuK*α*
Diffractometer	STOE STADIVARI
Crystal type	Monoclinic
Space group name, number	$P2_1$, (4)
Unit cell dimensions	a = 17.7846(13) Å α = 90°
	b = 8.5371(4) Å β = 106.358(6)°
	c = 26.505(2) Å γ = 90°
Volume	3861.3(5) Å^3
Number of reflections	18838
And range used for lattice Parameters	2.59° <=Θ<= 69.30°
Z	8
Density (calculated)	1.362 Mg/m^3
Absorption coefficient	2.671 mm^{-1}
Absorption correction	integration
Max. and min. transmission	0.7735 and 0.4134
F(000)	1648
Crystal size, color and form	0.060 x 0.080 x 0.690 mm^3, colourless needle
Theta range for data collection	2.589 to 69.932°
Index ranges	-21<=h<=19, -10<=k=10, -31=l<=32
Number of reflections:	
Collected	37837
Independent	13674 [R_{int}= 0.1130]
Observed [I>2sigma(I)]	6363
Completeness of theta = 25.2°	99.1%
Refinement method	Full-matrix least-squares on F^2
Data / restraints / parameters	13674 / 49 / 973
Goodness-of-fit on F^2	1.166
Final R indices [I>2sigma(I)]	R1 = 0.1450, wR2 = 0.3428
R indices (all data)	R1 = 0.2361, wR2 = 0.3989
Absolute structure parameter	-0.01(6)
Largest diff. peak and hole	0.930 and -0.510 eÅ^{-3}
Comment	Crystal contains four distinct molecules

10.3. X-ray crystal structure analysis of [*cis*-11a] (468f2)

A colorless block like specimen of $C_{10}H_9N$, approximatel dimensions 0.060 x 0.270 x 0.530 mm^3, was used for the X-ray crystallographic analysis. The x-ray intensity data were measured on a STOE STADIVARI Diffractometer system. CCDC number: 2419831

Table S13: Crystal data and structure refinement for [cis-11a] (468f2)

Identification code	468f2
Empirical formula	$C_{10}H_9N$
Moiety formula	$C_{10}H_9N$
Formula weight	143.18
Temperature	120(2) K
Wavelength, radiation type	1.54178Å, CuKα
Diffractometer	STOE STADIVARI
Crystal type	Monoclinic
Space group name, number	$P2_1$, (4)
Unit cell dimensions	a = 5.8962(3) Å α = 90°
	b = 8.0964(4) Å β = 106.756(5)°
	c = 8.5292(5) Å γ = 90°
Volume	389.88(4) Å^3
Number of reflections	6656
And range used for lattice Parameters	5.42°<=Θ<=68.91°
Z	2
Density (calculated)	1.220 Mg/m^3
Absorption coefficient	0.553 mm^{-1}
Absorption correction	integration
Max. and min. transmission	0.9645 and 0.8243
F(000)	152
Crystal size, color and form	0.060 x 0.270 x 0.530 mm^3, colorless block
Theta range for data collection	5.416 to 68.227°
Index ranges	-6<=h<=7, -9<=k=9, -10<=l<=10
Number of reflections:	
Collected	3124
Independent	1345 [R_{int}=0.0159]
Observed [I>2sigma(I)]	1314
Completeness of theta = 25.2°	99.3%
Refinement method	Full-matrix least-squares on F^2
Data / restraints / parameters	1345 / 1 / 100
Goodness-of-fit on F^2	1.061
Final R indices [I>2sigma(I)]	R1 = 0.0269, wR2 = 0.0705
R indices (all data)	R1 = 0.0275, wR2 = 0.0710
Absolute structure parameter	0.1(3)
Largest diff. peak and hole	0.099 and -0.150 eÅ^{-3}

10.4. X-ray crystal structure analysis of [cis-11i] (jha156)

A colorless needle like specimen of $C_{10}H_8ClN$, approximate dimensions 0.070 x 0.080 x 0.210 mm^3, was used for the X-ray crystallographic analysis. The x-ray intensity data were measured on a STOE STADIVARI Diffractometer system. CCDC number: 2419832

Table S14: Crystal data and structure refinement for [cis-11i] (jha156)

Identification code	jha156
Empirical formula	$C_{10}H_8ClN$
Moiety formula	$C_{10}H_8ClN$
Formula weight	177.62
Temperature	120(2) K
Wavelength, radiation type	1.54178Å, CuKα
Diffractometer	STOE STADIVARI
Crystal type	Monoclinic
Space group name, number	$P2_12_12_1$, (19)
Unit cell dimensions	a = 5.6996(2) Å α = 90°
	b = 8.3011(3) Å β = 90°
	c = 18.4450(9) Å γ = 90°
Volume	872.69(6) Å^3
Number of reflections	6700
And range used for lattice Parameters	5.85°<=Θ<=69.04°
Z	4
Density (calculated)	1.352 Mg/m^3
Absorption coefficient	3.354 mm^{-1}
Absorption correction	integration
Max. and min. transmission	0.8089 and 0.6300
F(000)	368
Crystal size, color and form	0.070 x 0.080 x 0.210 mm^3, colorless needle
Theta range for data collection	5.845 to 68.292°
Index ranges	-6<=h<=7, -9<=k=9, -21<=l<=21
Number of reflections:	
Collected	5146
Independent	1533 [R_{int}=0.0231]
Observed [I>2sigma(I)]	1433
Completeness of theta = 25.2°	98.9%
Refinement method	Full-matrix least-squares on F^2
Data / restraints / parameters	1533 / 0 / 109
Goodness-of-fit on F^2	0.953
Final R indices [I>2sigma(I)]	R1 = 0.0230, wR2 = 0.0505
R indices (all data)	R1 = 0.0244, wR2 = 0.0506
Absolute structure parameter	-0.016(11)
Largest diff. peak and hole	0.127 and -0.147 eÅ^{-3}

11. References

[1] C. P. Rosenau, B. J. Jelier, A. D. Gossert, A. Togni, *Angew. Chem. Int. Ed.* **2018**, *57*, 9528.
[2] J. Sietmann, M. Ong, C. Mück-Lichtenfeld, C. G. Daniliuc, J. M. Wahl, *Angew. Chem. Int. Ed.* **2021**, *60*, 9719.
[3] M. Ong, M. Arnold, A. W. Walz, J. M. Wahl, *Org. Lett.* **2022**, *24*, 6171.
[4] J. Guo, X. Xu, Q. Xing, Z. Gao, J. Gou, B. Yu, *Org. Lett.* **2018**, *20*, 7410.
[5] H.-J. Xu, F.-F. Zhu, Y.-Y. Shen, X. Wan, Y.-S. Feng, *Tetrahedron* **2012**, *68*, 4145.
[6] K. S. Petersen, B. M. Stoltz, *Tetrahedron* **2011**, *67*, 4352.
[7] A. V. Malkov, F. Friscourt, M. Bell, M. E. Swarbrick, P. Kocovský, *J. Org. Chem.* **2008**, *73*, 3996.
[8] M. Tenberge, J. M. Wahl, *Synthesis* **2023**, *55*, 892.
[9] A. Drożdż, M. Foreiter, A. Chrobok, *Synlett* **2014**, *25*, 559.
[10] E. J. Corey, N. Imai, H. Y. Zhang, *J. Am. Chem. Soc.* **1991**, *113*, 728.
[11] J. Zhu, B. A. Price, S. X. Zhao, P. M. Skonezny, *Tetrahedron Lett.* **2000**, *41*, 4011.
[12] A. F. Littke, G. C. Fu, *Angew. Chem. Int. Ed.* **1999**, *38*, 2411.
[13] Y. Liu, J. Ao, S. Paladhi, C. E. Song, H. Yan, *J. Am. Chem. Soc.* **2016**, *138*, 16486.
[14] J. Liu, K. Wu, T. Shen, Y. Liang, M. Zou, Y. Zhu, X. Li, X. Li, N. Jiao, *Chem. Eur. J.* **2017**, *23*, 563.
[15] J. A. Smulik, E. Vedejs, *Org. Let.t* **2003**, *5*, 4187.
[16] Masruri, A. C. Willis, M. D. McLeod, *J. Org. Chem.* **2012**, *77*, 8480.
[17] C. A. Busacca, J. C. Lorenz, N. Grinberg, N. Haddad, M. Hrapchak, B. Latli, H. Lee, P. Sabila, A. Saha, M. Sarvestani et al., *Org. Lett* .**2005**, *7*, 4277.
[18] M. J. P. Harger, *J. Chem. Soc., Perkin Trans. 1* **1981**, 3284.
[19] T. Nishimura, Y. Nishiguchi, Y. Maeda, S. Uemura, *J. Org. Chem.* **2004**, *69*, 5342.
[20] Y.-R. Gu, X.-H. Duan, L. Yang, L.-N. Guo, *Org. Lett.* **2017**, *19*, 5908.
[21] TURBOMOLE V7.8 2023, a development of University of Karlsruhe and Forschungszentrum Karlsruhe GmbH, 1989-2007, TURBOMOLE GmbH, since 2007, available from http://www.turbomole.org.
[22] J. Tao, J. P. Perdew, V. N. Staroverov, G. E. Scuseria, *Phys. Rev. Lett.* **2003**, *91*, 146401.
[23] a) S. Grimme, S. Ehrlich, L. Goerigk, *J. Compt. Chem.* **2011**, *32*, 1456; b) S. Grimme, J. Antony, S. Ehrlich, H. Krieg, *J. Chem. Phys.* **2010**, *132*, 154104.
[24] F. Weigend, R. Ahlrichs, *Phys. Chem. Chem. Phys.* **2005**, *7*, 3297.
[25] S. Grimme, *Chem. Eur. J.* **2012**, *18*, 9955.
[26] Y. Zhao, D. G. Truhlar, *J. Chem. Phys.* **2005**, *109*, 5656.
[27] a) A. Klamt, *J. Chem. Phys.* **1995**, *99*, 2224; b) Eckert, F. & Klamt, A. COSMOtherm, Version C3.0; COSMOlogic GmbH & Co. KG, Leverkusen, Germany (2013).
[28] M. Klussmann, L. Ratjen, S. Hoffmann, V. Wakchaure, R. Goddard, B. List, *Synlett* **2010**, *2010*, 2189.
[29] V. Rauniyar, Z. J. Wang, H. E. Burks, F. D. Toste, *J. Am. Chem. Soc.* **2011**, *133*, 8486.

[30] E. Horáková, P. Drabina, L. Brůčková, Š. Štěpánková, K. Vorčáková, M. Sedlák, *Monatsh. Chem.* **2017**, *148*, 2143.
[31] J. P. Hilton-Proctor, O. Ilyichova, Z. Zheng, I. G. Jennings, R. W. Johnstone, J. Shortt, S. J. Mountford, M. J. Scanlon, P. E. Thompson, *Eur. J. Med. Chem.* **2020**, *191*, 112120.
[32] B. Zhao, Z. Shi, *Angew. Chem. Int. Ed.* **2017**, *56*, 12727.
[33] B. Shuai, P. Fang, T.-S. Mei, *Synlett* **2021**, *32*, 1637.
[34] A. L. Chandgude, R. Fasan, *Angew. Chem. Int. Ed.* **2018**, *57*, 15852.
[35] L. An, F.-F. Tong, S. Zhang, X. Zhang, *J. Am. Chem. Soc.* **2020**, *142*, 11884.
[36] a) B. Y. Kimura, T. L. Brown, *J. Organomet. Chem.* **1971**, *26*, 57; b) J. F. Remenar, B. L. Lucht, D. B. Collum, *J. Am. Chem. Soc.* **1997**, *119*, 5567; c) B. L. Lucht, D. B. Collum, *J. Am. Chem. Soc.* **1996**, *118*, 2217.
[37] a) C. S. Beshara, A. Hall, R. L. Jenkins, T. C. Jones, R. T. Parry, S. P. Thomas, N. C. O. Tomkinson, *Chem. Commun. (Cambridge, England)* **2005**, 1478; b) C. S. Beshara, A. Hall, R. L. Jenkins, K. L. Jones, T. C. Jones, N. M. Killeen, P. H. Taylor, S. P. Thomas, N. C. O. Tomkinson, *Org. Lett.* **2005**, *7*, 5729; c) O. R. S. John, N. M. Killeen, D. A. Knowles, S. C. Yau, M. C. Bagley, N. C. O. Tomkinson, *Org. Lett.* **2007**, *9*, 4009.
[38] M.-X. Wang, G.-Q. Feng, *New J. Chem.* **2002**, *26*, 1575.
[39] H. Saitoh, T. Watanabe, T. Kimura, Y. Kato, T. Satoh, *Tetrahedron* **2012**, *68*, 2481.
[40] K. J. Hock, R. Spitzner, R. M. Koenigs, *Green Chem.* **2017**, *19*, 2118.
[41] X.-A. Li, L. Yue, J. Zhu, H. Ren, H. Zhang, D. Hu, G. Han, J. Feng, Z. Nan, *Tetrahedron Lett.* **2019**, *60*, 1986.

12. NMR Spectra and HPLC Traces

[1p] ^{1}H, $CDCl_3$, 600 MHz

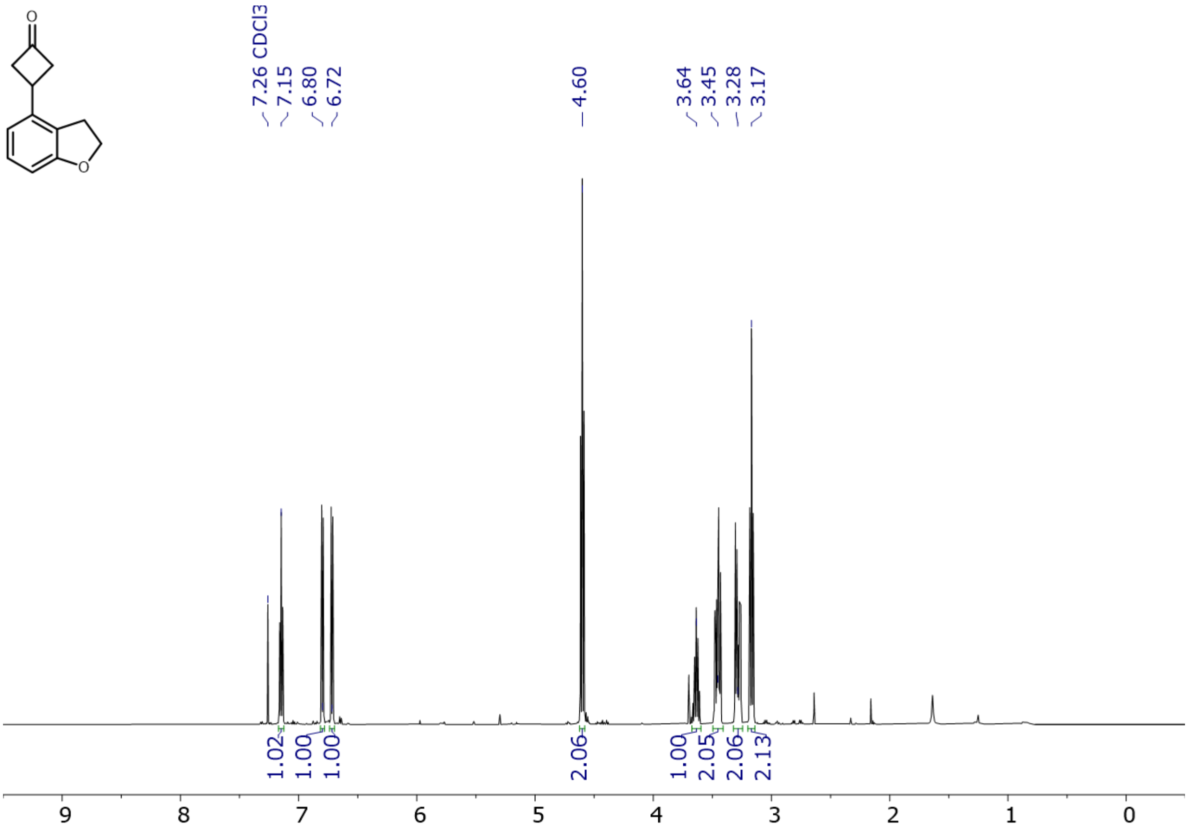

[1p] ^{13}C, $CDCl_3$, 151 MHz

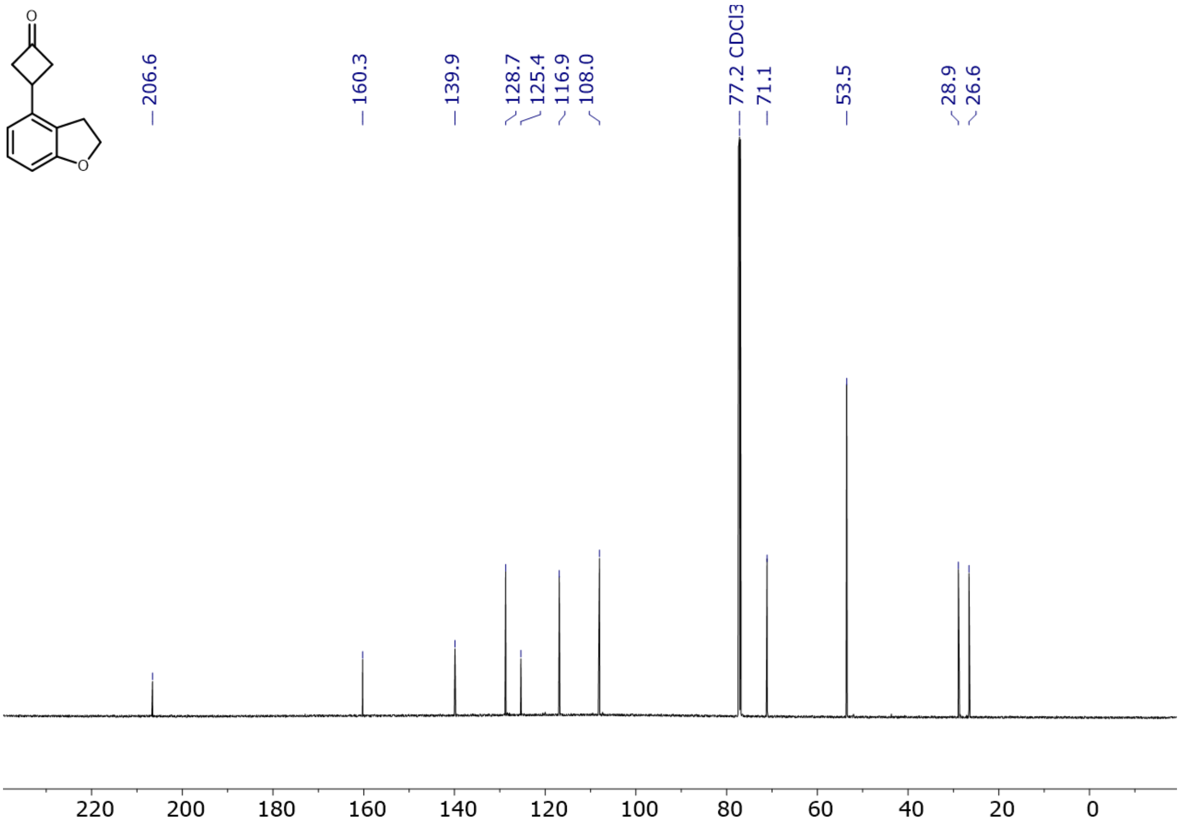

[S7] ^{1}H, $CDCl_3$, 400 MHz

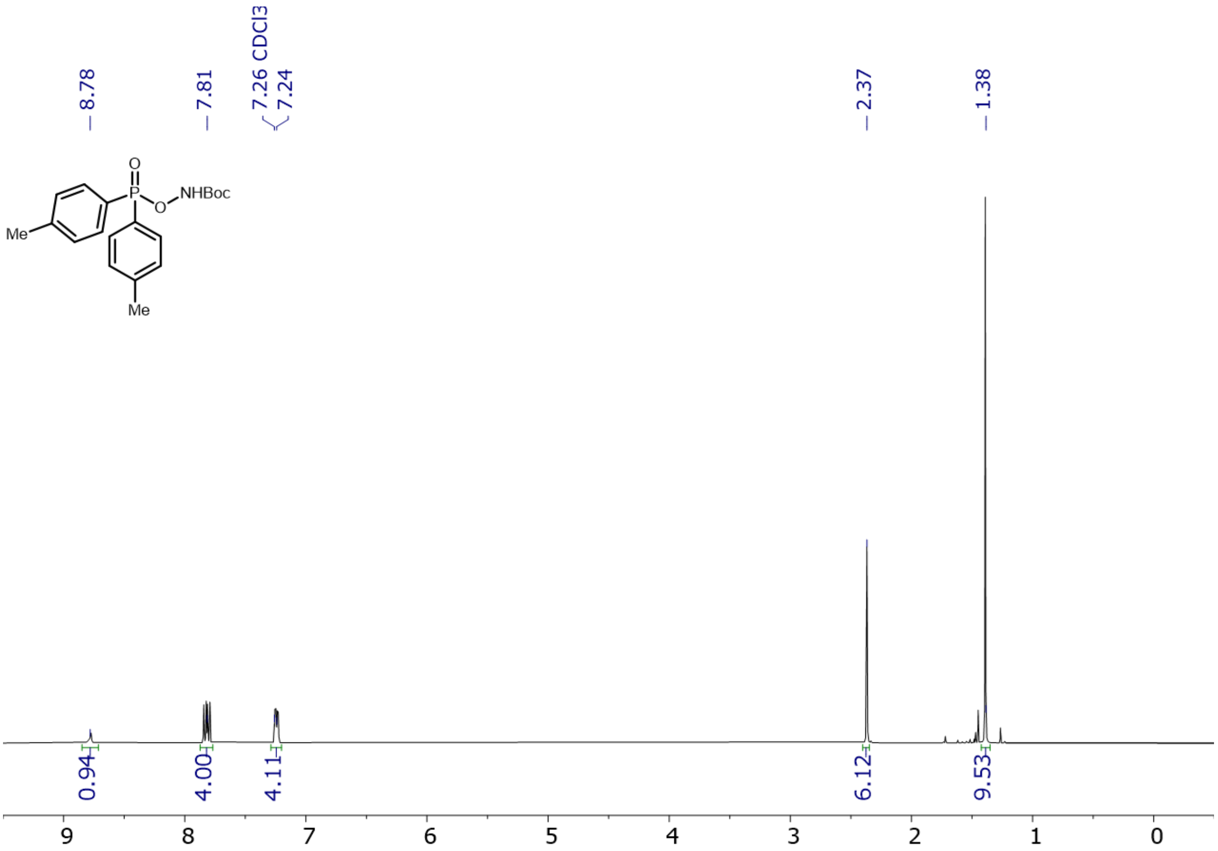

[S7] ^{13}C, $CDCl_3$, 101 MHz

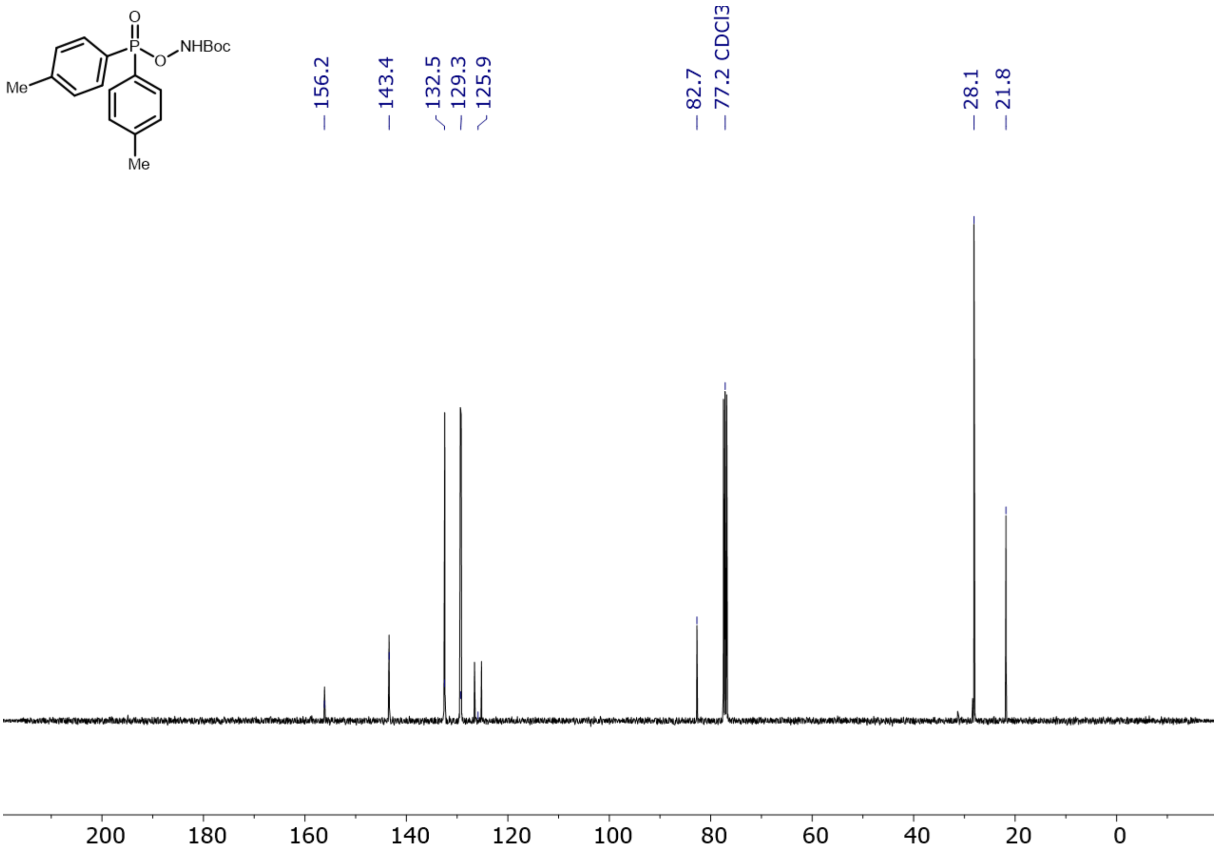

[S7] ^{31}P, $CDCl_3$, 162 MHz

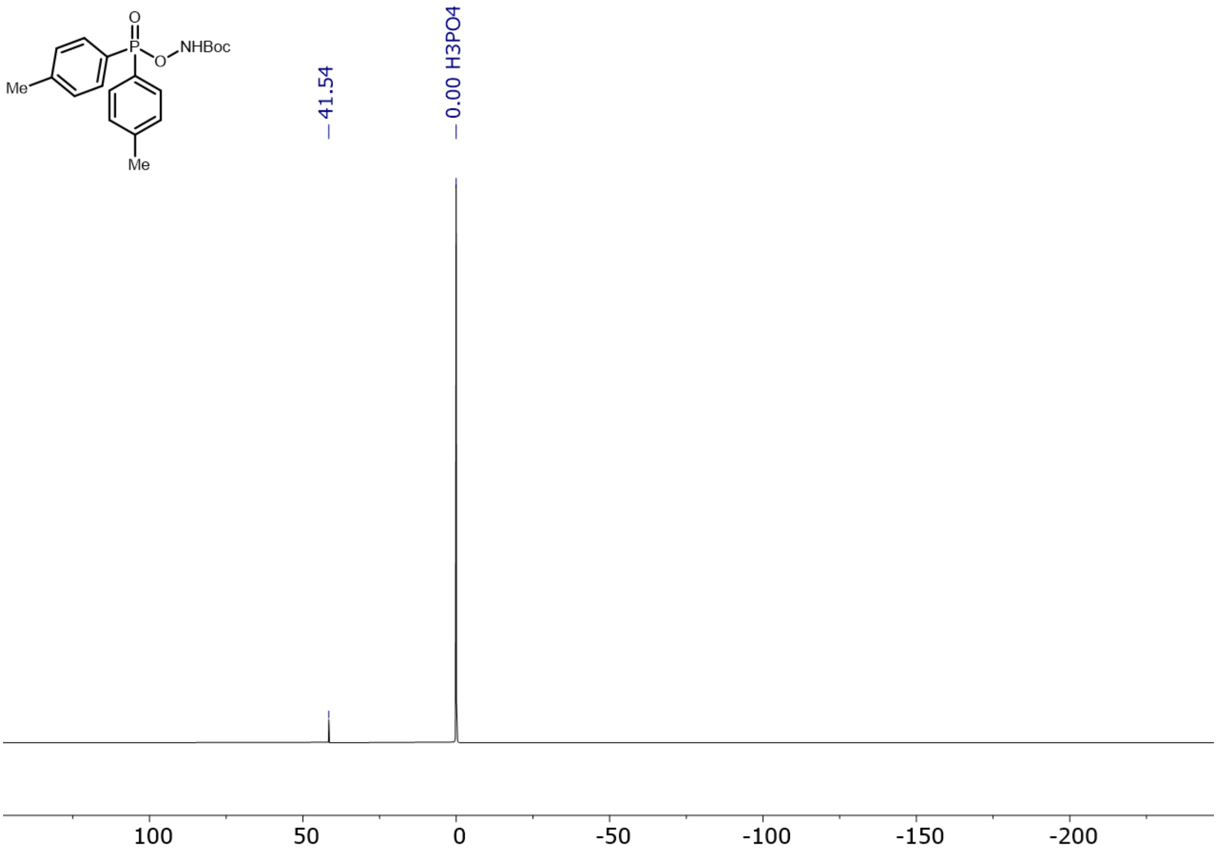

[S11] ^{1}H, $CDCl_3$, 600 MHz

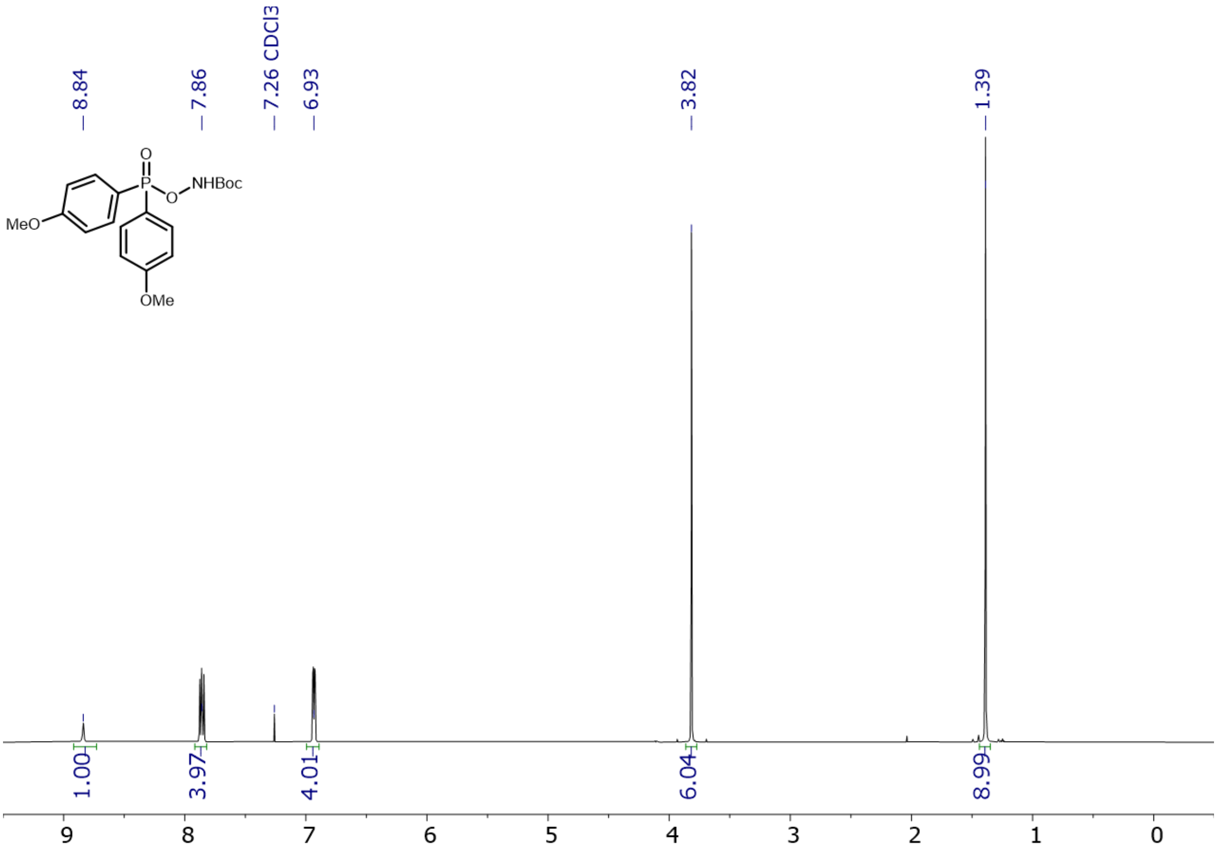

[S11] ^{13}C, $CDCl_3$, 151 MHz

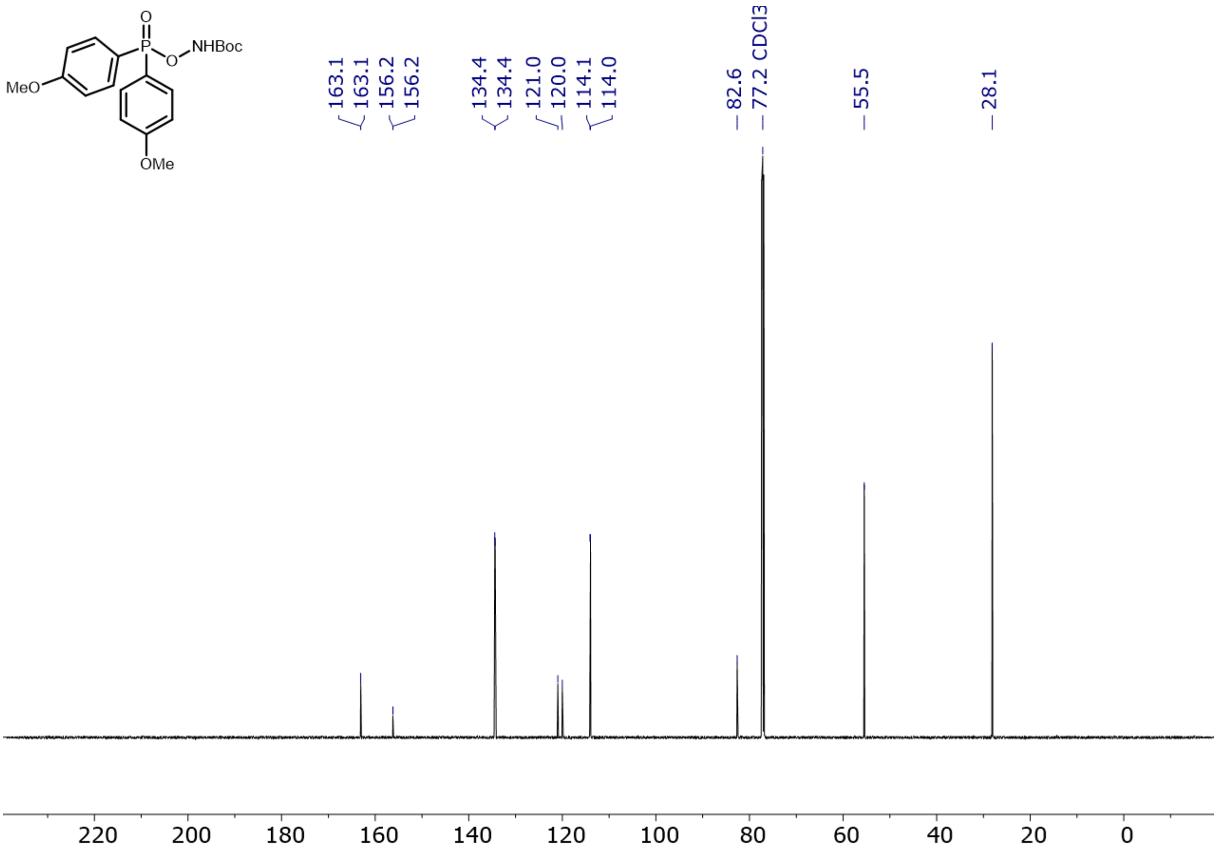

[S11] ^{31}P, $CDCl_3$, 162 MHz

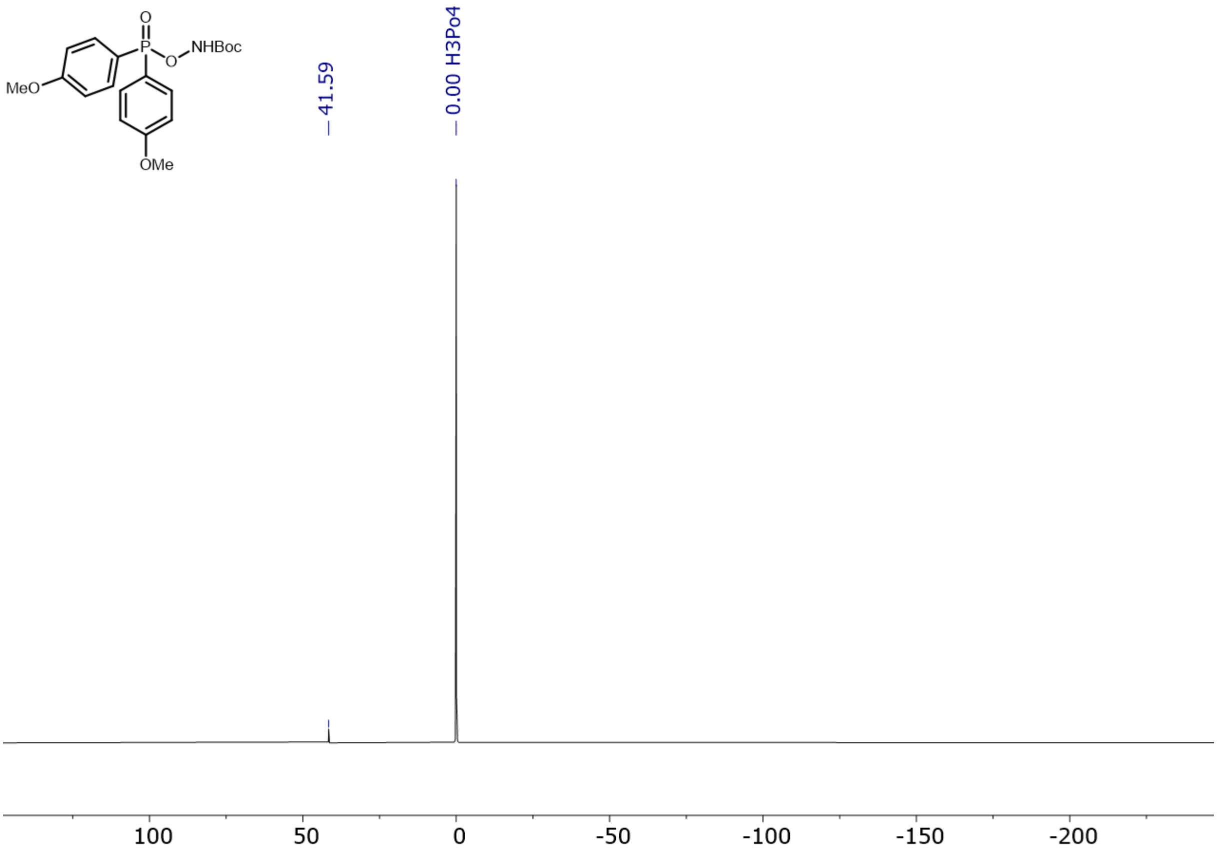

[S13] ^1H, $CDCl_3$, 400 MHz

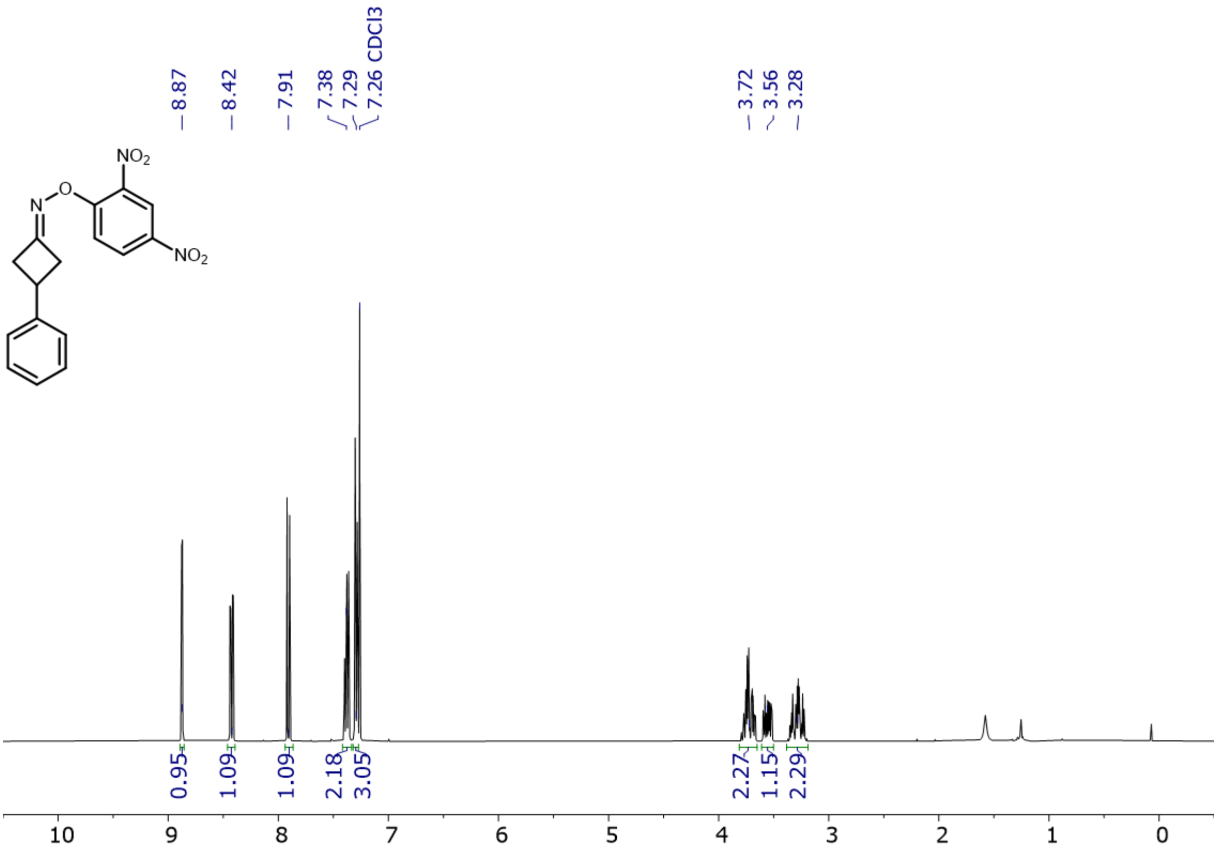

[S13] ^{13}C, $CDCl_3$, 101 MHz

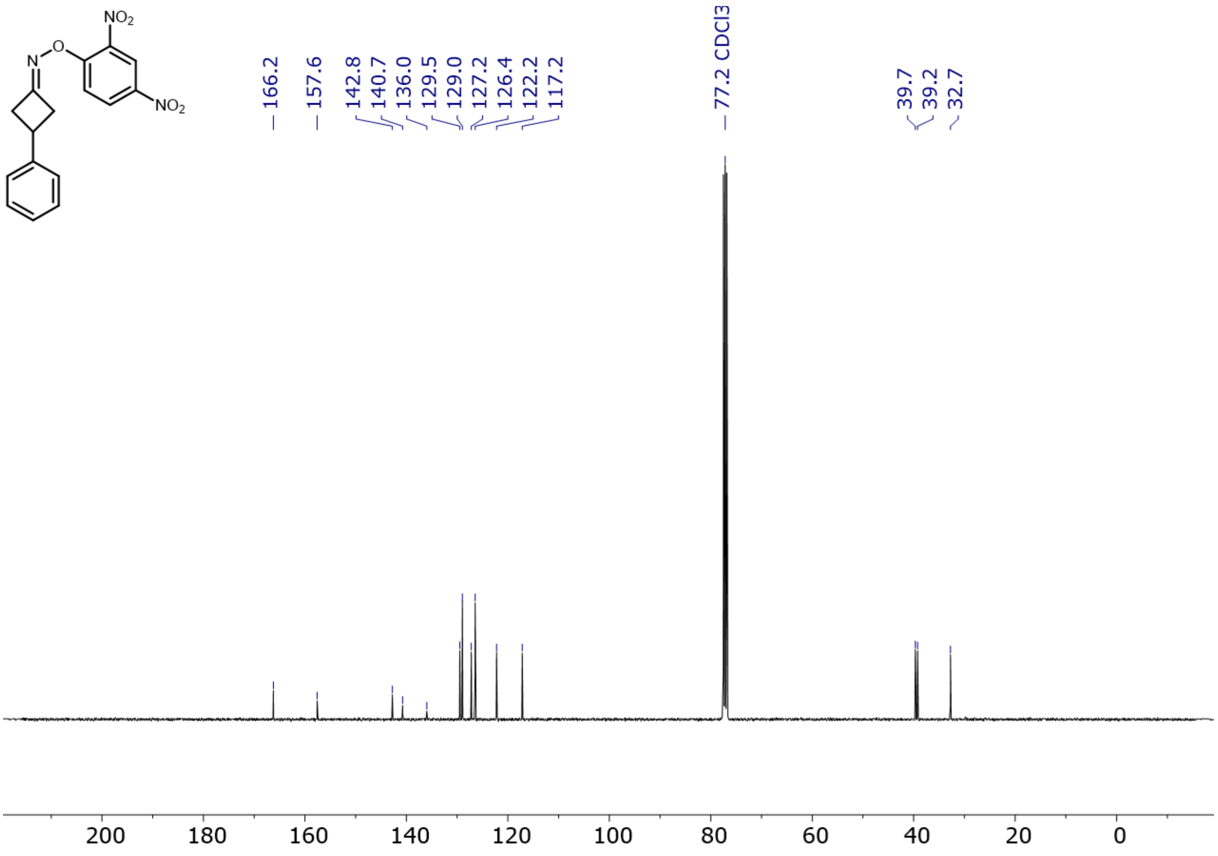

[S14] ^{1}H, $CDCl_3$, 400 MHz

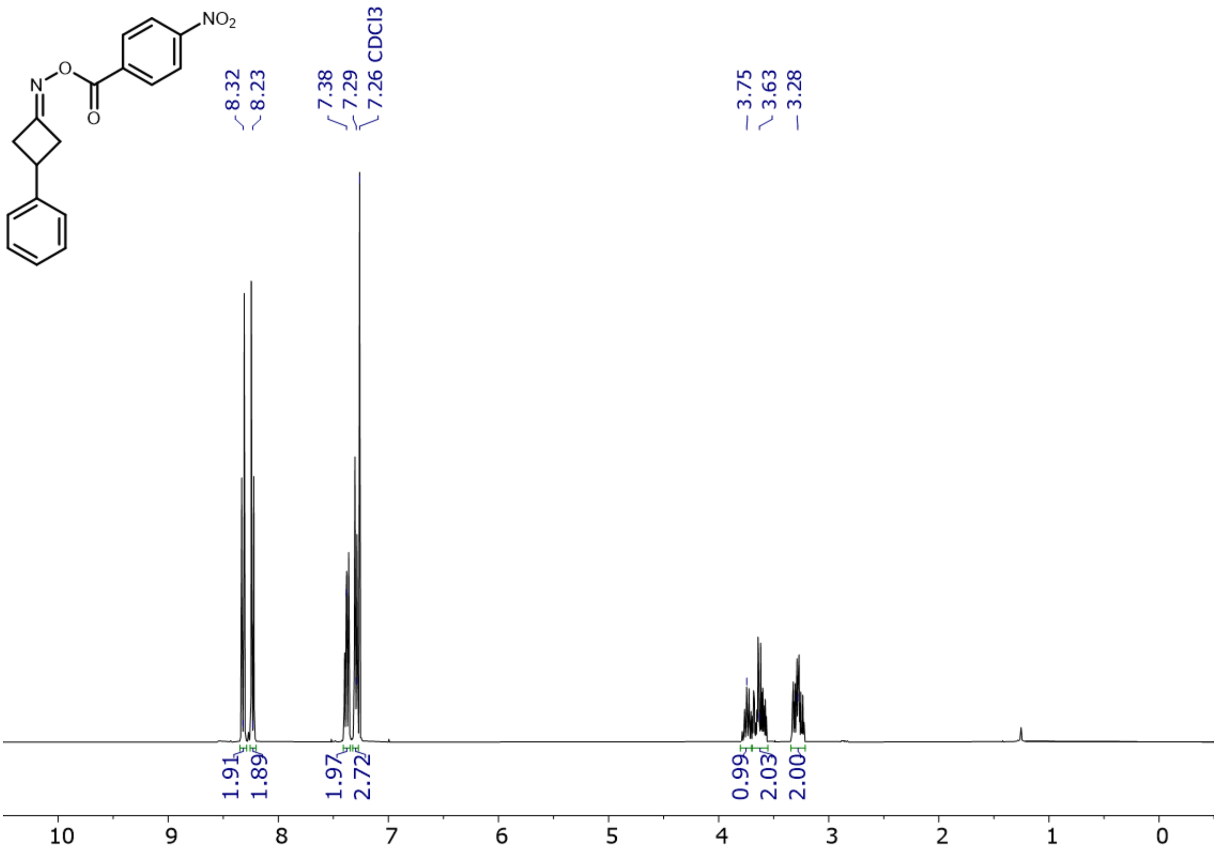

[S14] ^{13}C, $CDCl_3$, 101 MHz

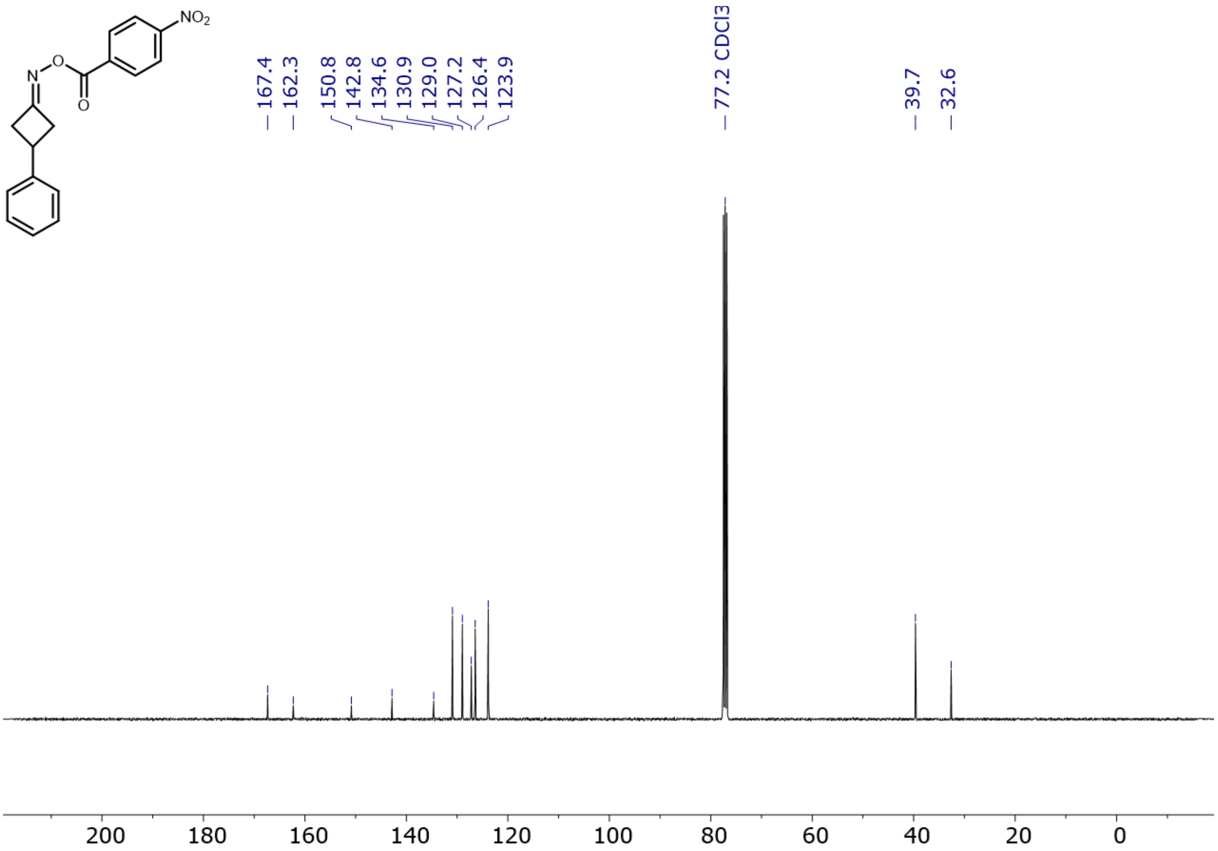

[S17] ^{1}H, $CDCl_3$, 400 MHz

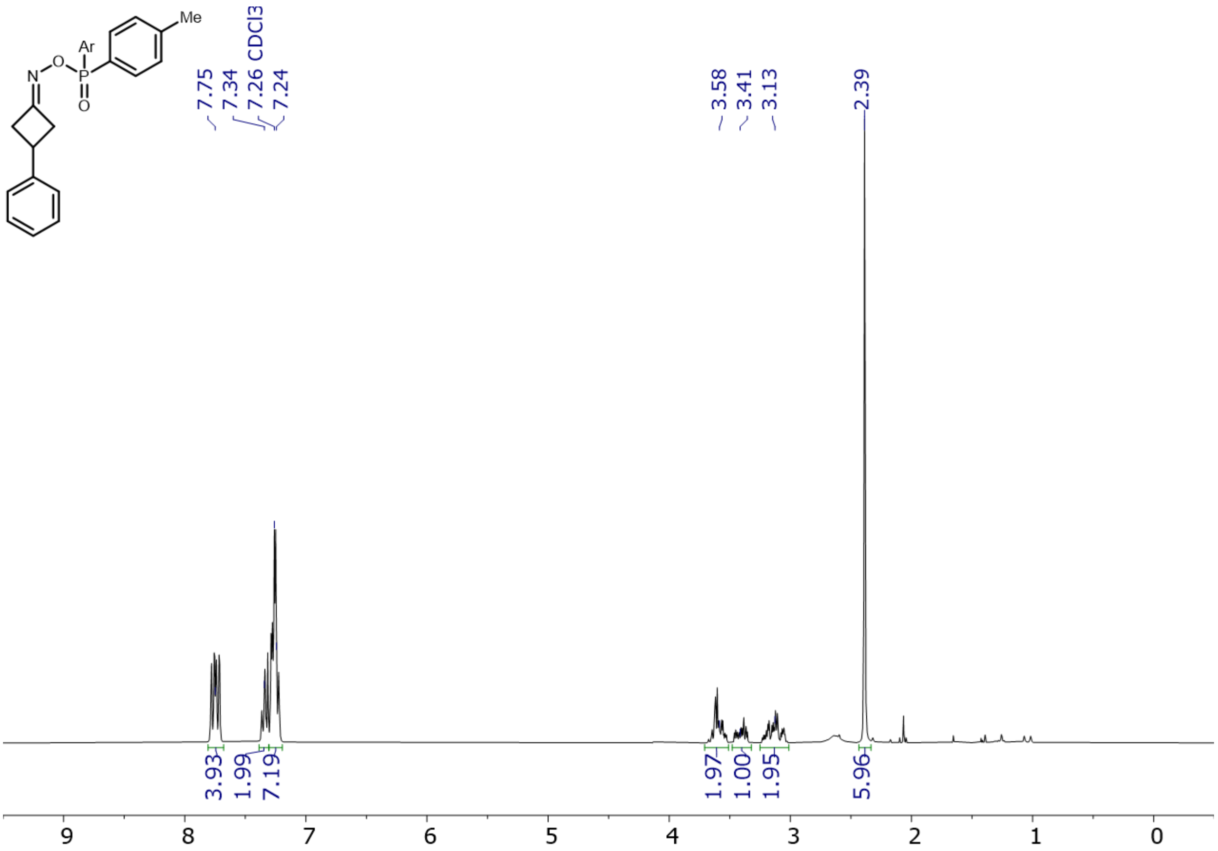

[S17] ^{13}C, $CDCl_3$, 101 MHz

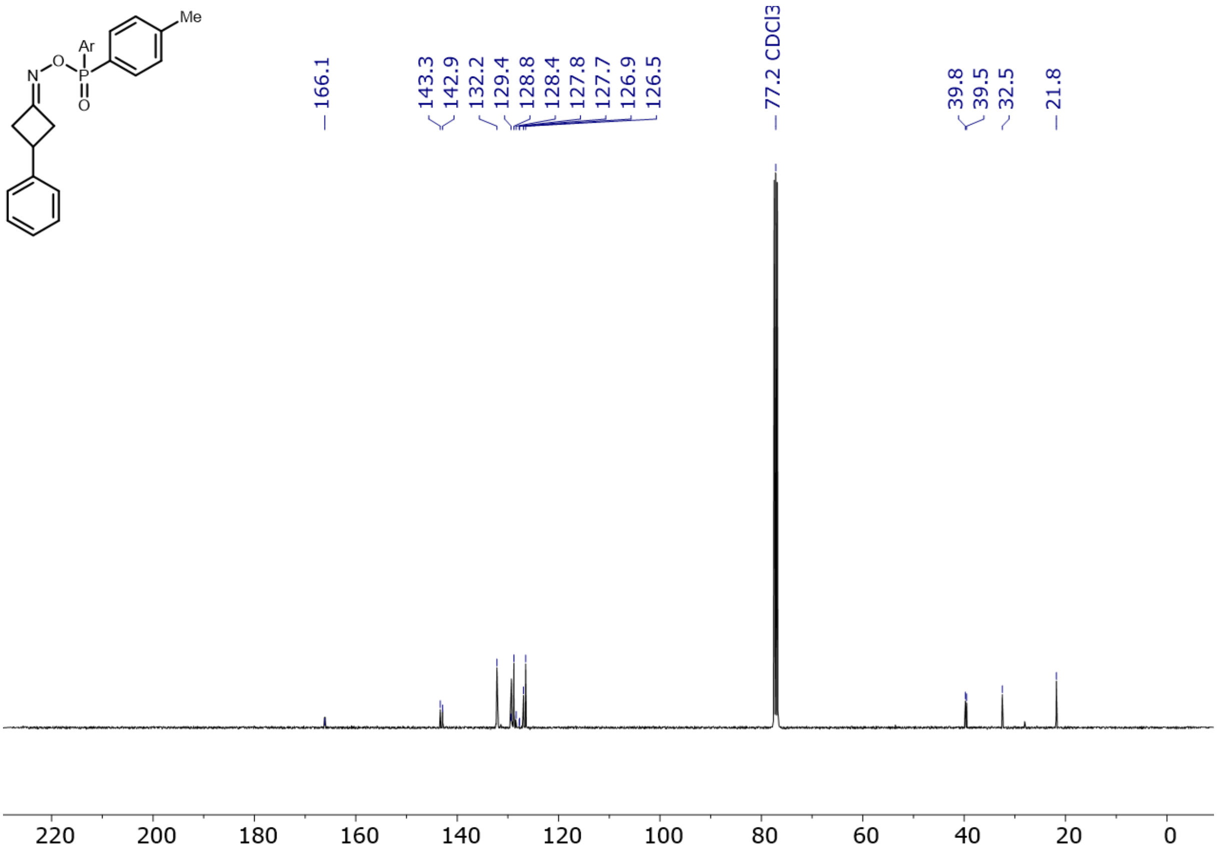

[S17] ^{31}P, $CDCl_3$, 162 MHz

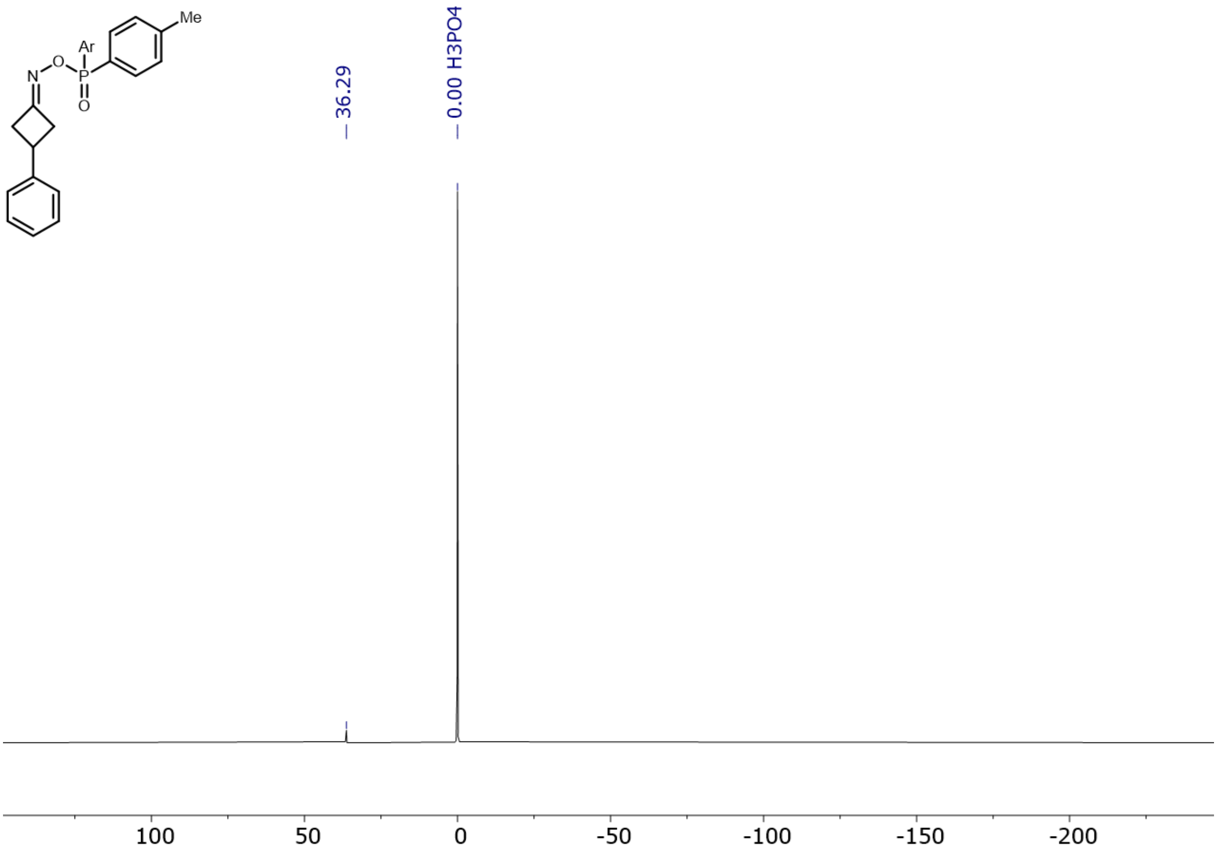

[S18] ^{1}H, $CDCl_3$, 600 MHz

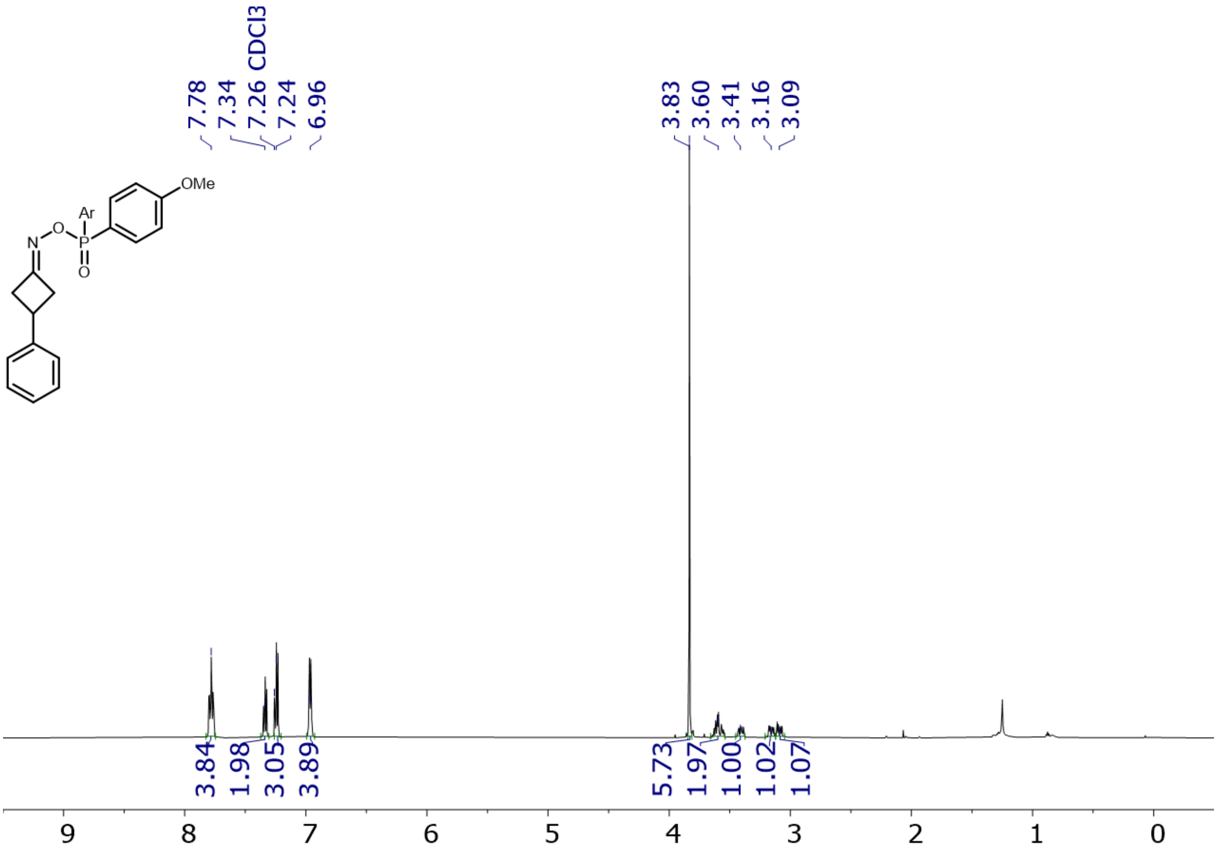

[S18] ^{13}C, $CDCl_3$, 151 MHz

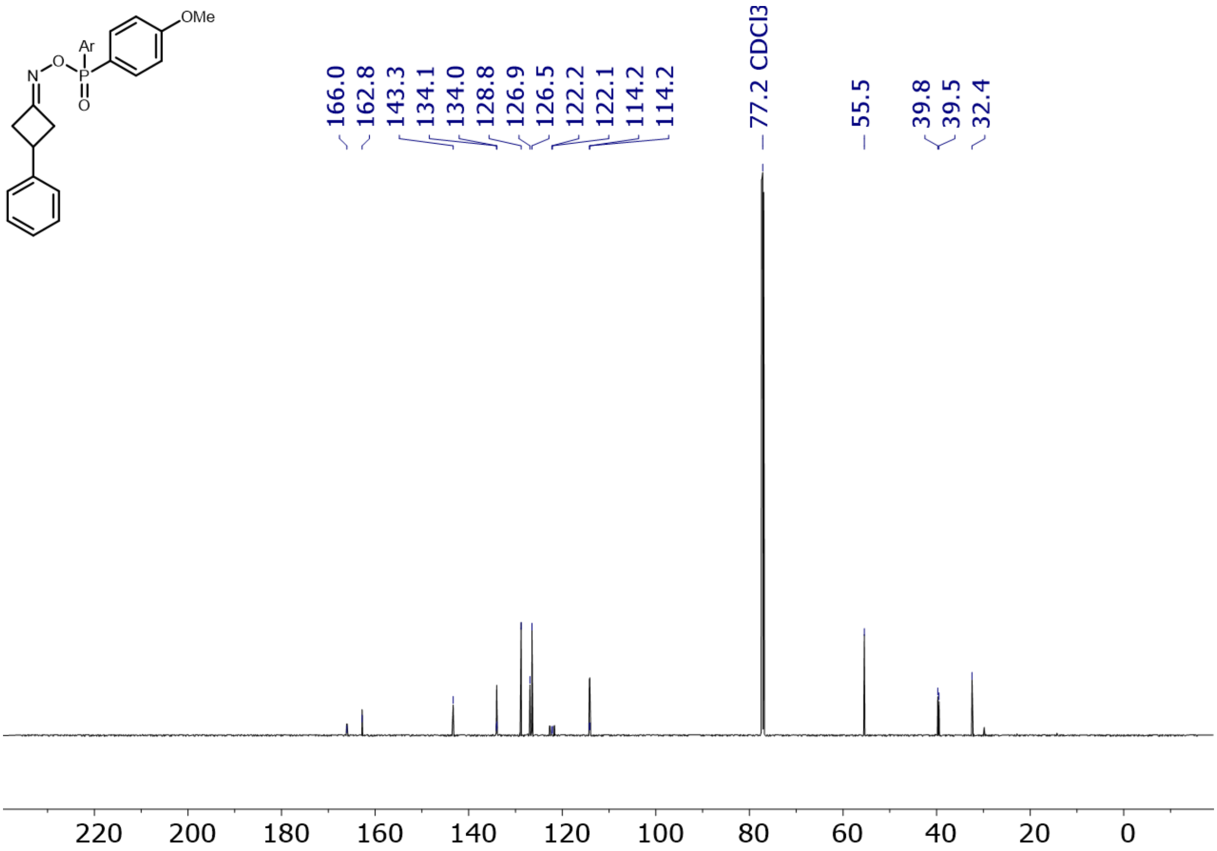

[S18] ^{31}P, $CDCl_3$, 162 MHz

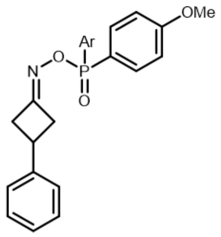

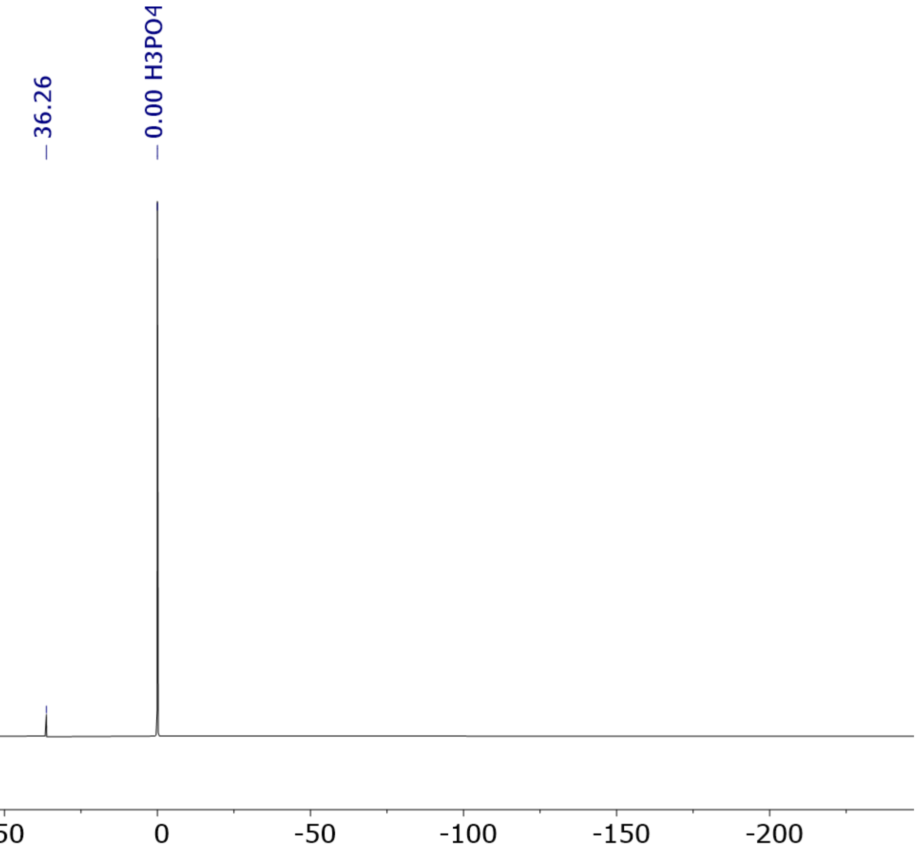

[6a] ^{1}H, $CDCl_3$, 400 MHz

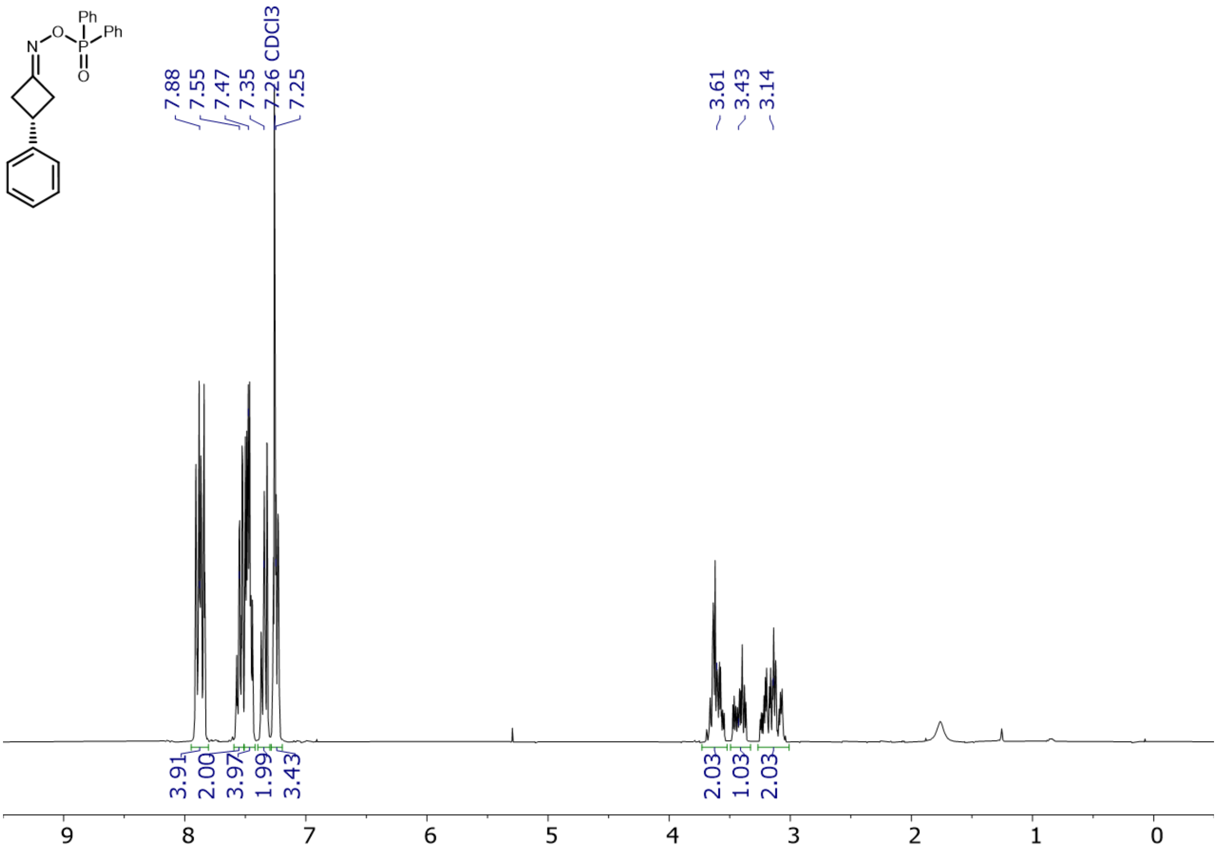

[6a] ^{13}C, $CDCl_3$, 101 MHz

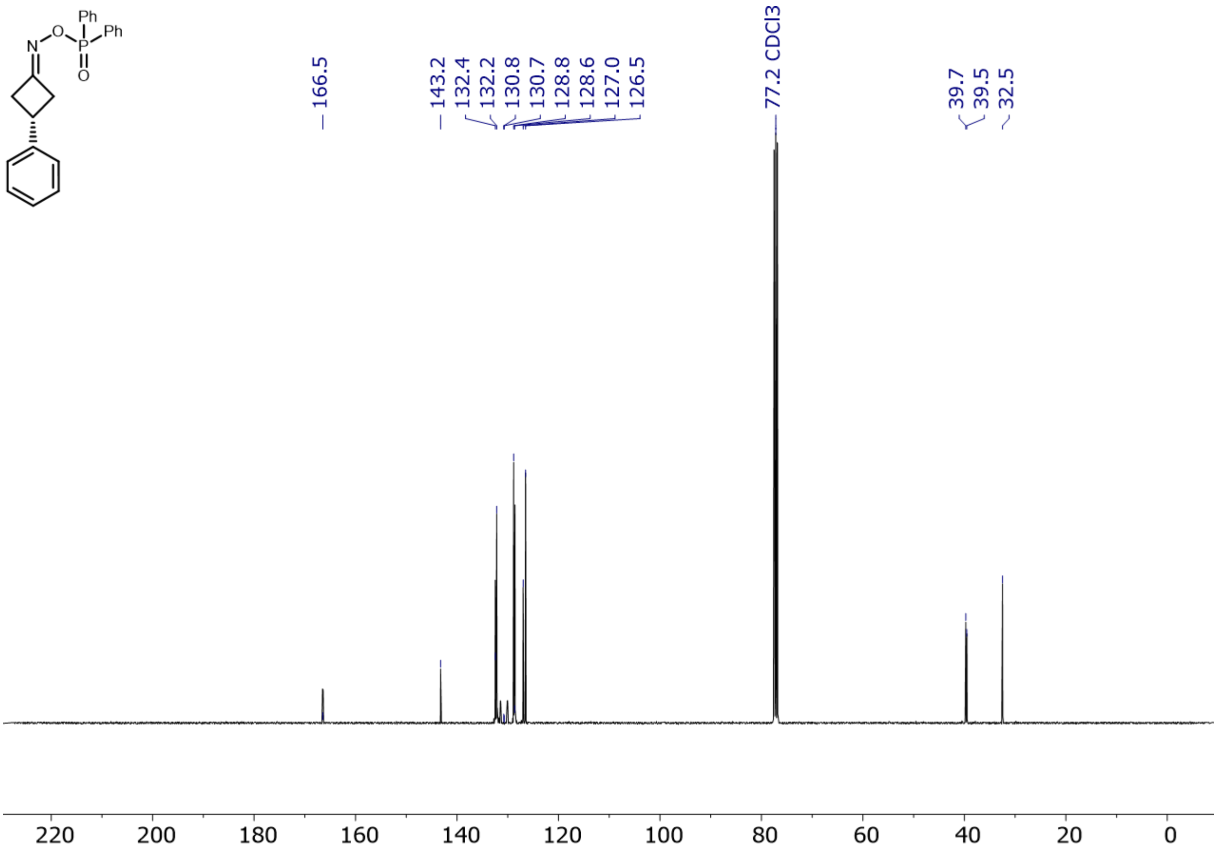

[6a] ^{31}P, $CDCl_3$, 162 MHz

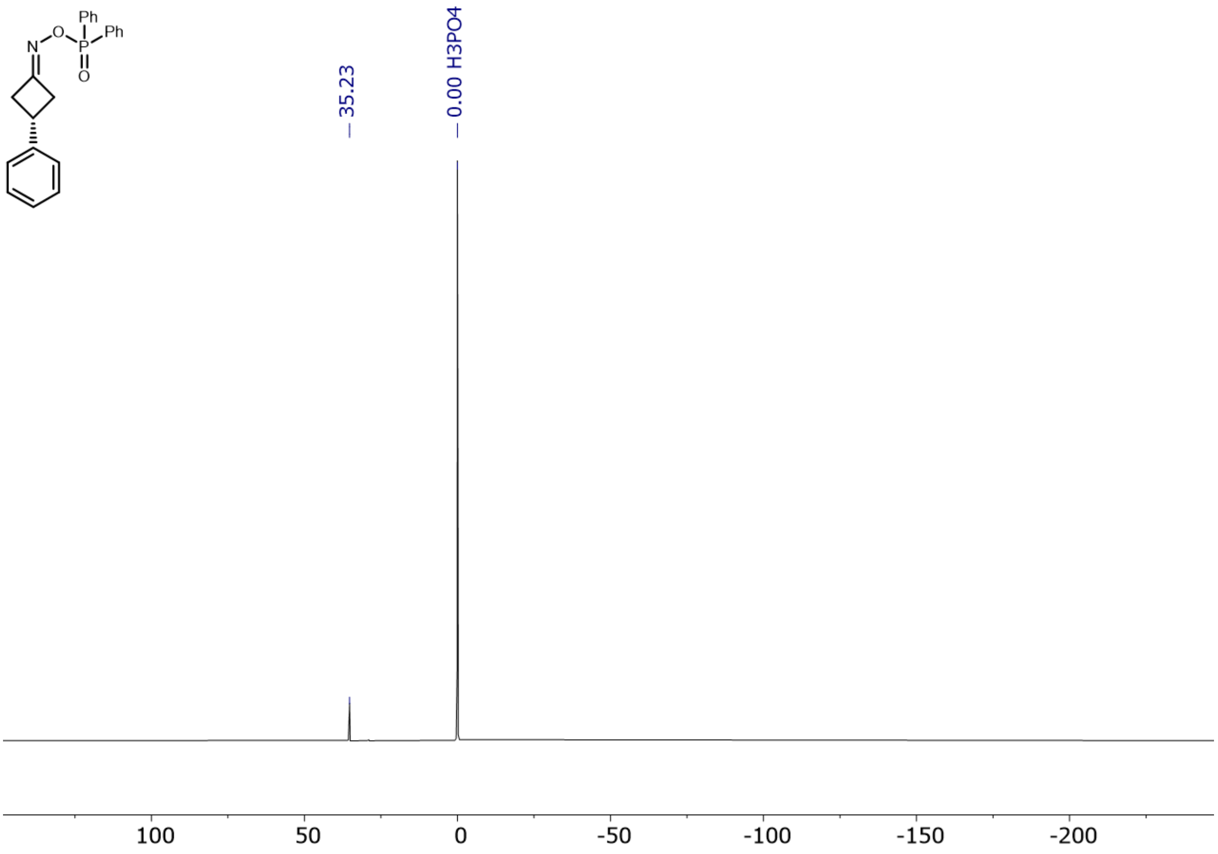

[6b] ^{1}H, $CDCl_3$, 400 MHz

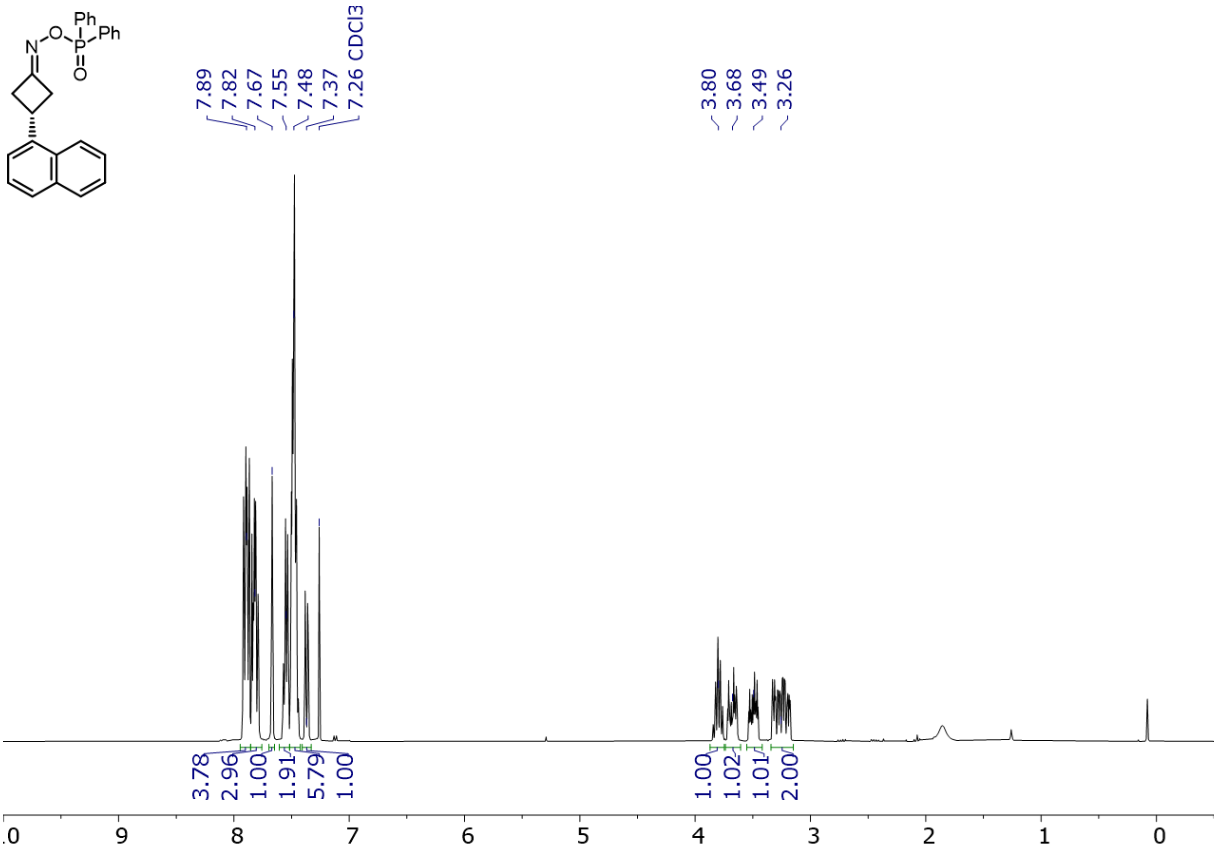

[6b] ^{13}C, $CDCl_3$, 101 MHz

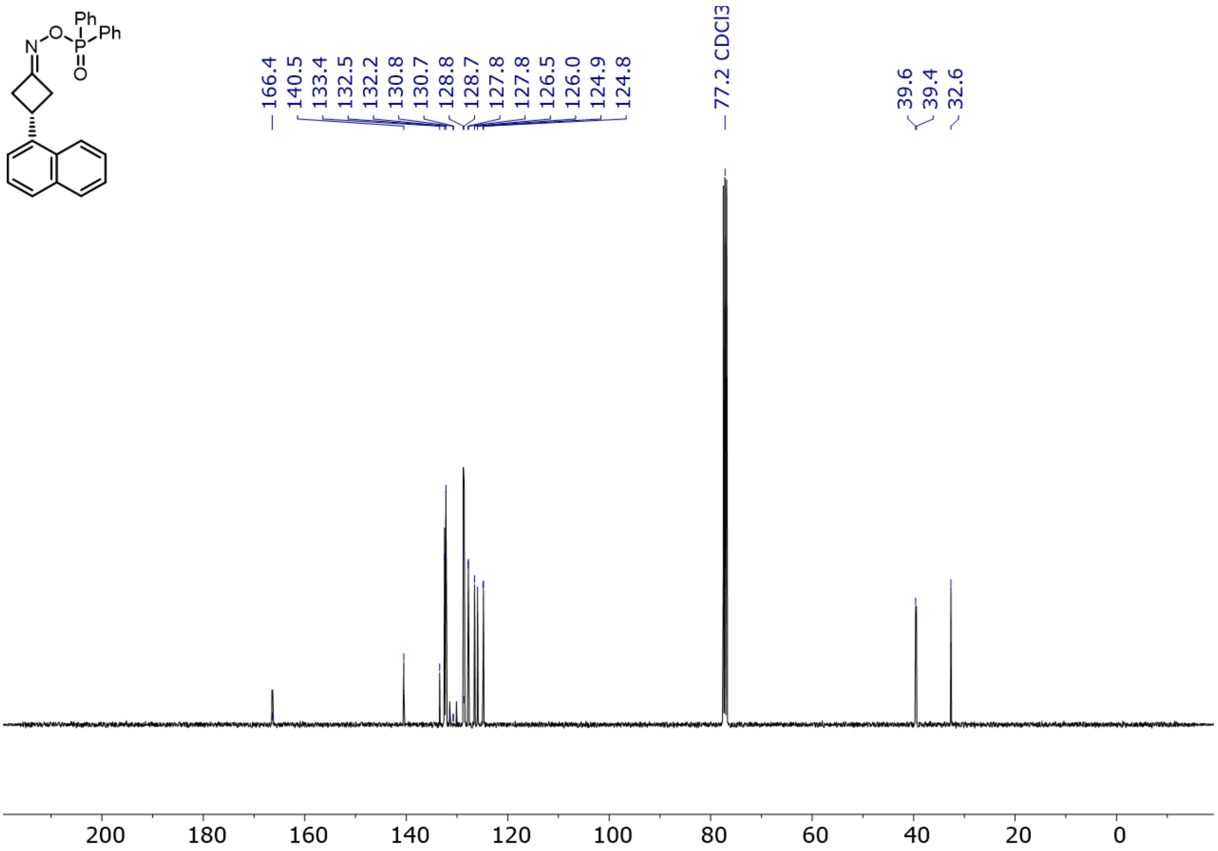

[6b] ^{31}P, $CDCl_3$, 162 MHz

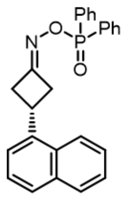

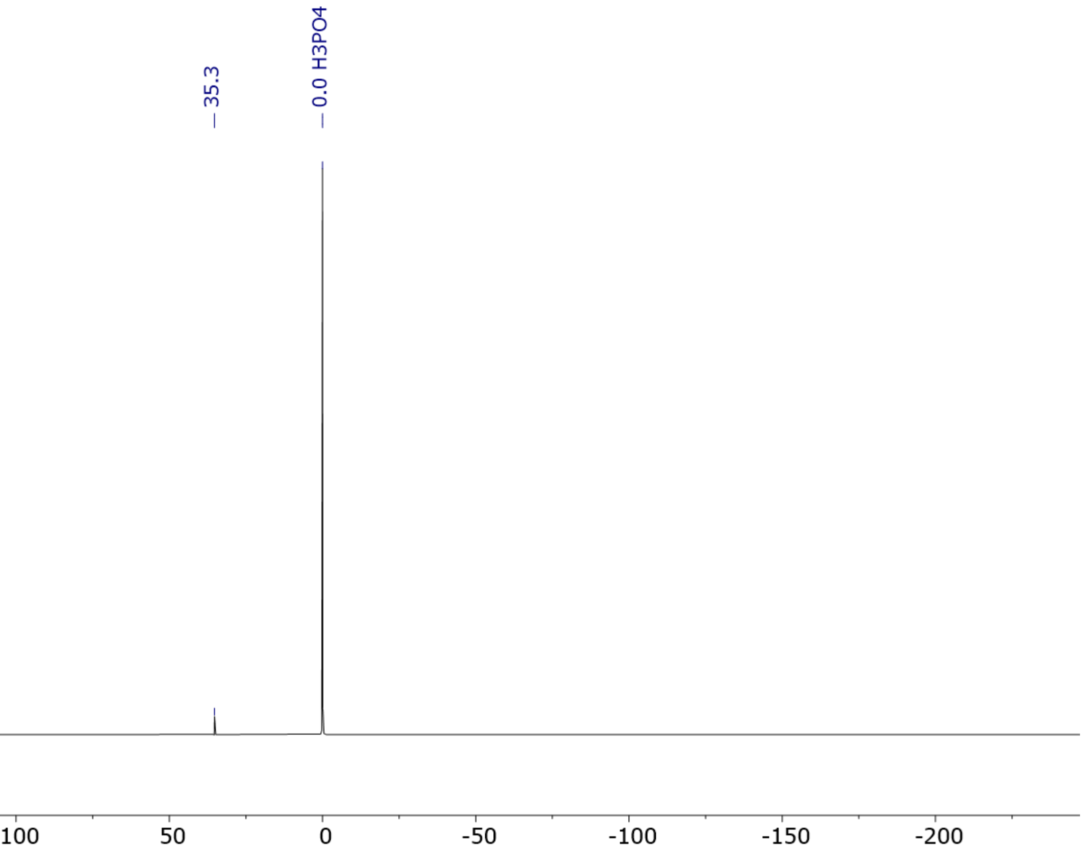

[6c] ^{1}H, $CDCl_3$, 400 MHz

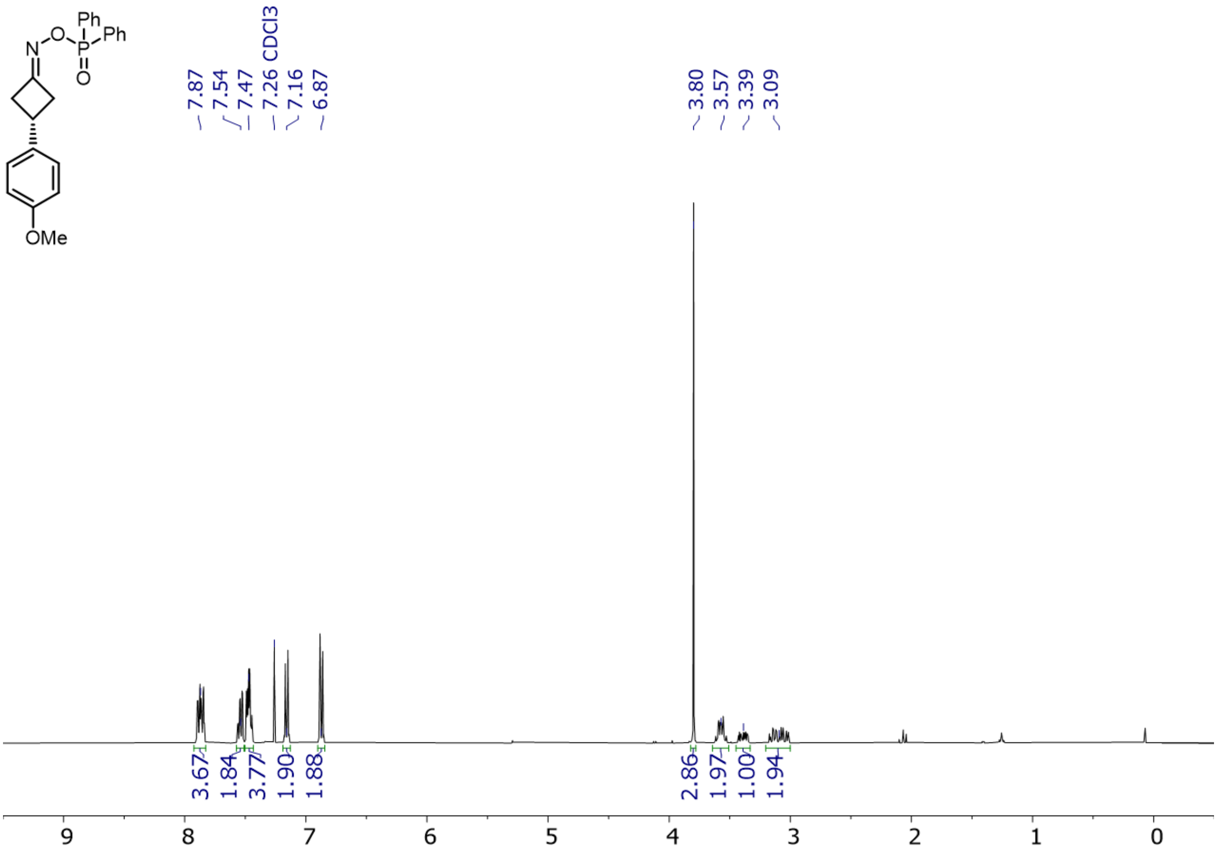

[6c] ^{13}C, $CDCl_3$, 101 MHz

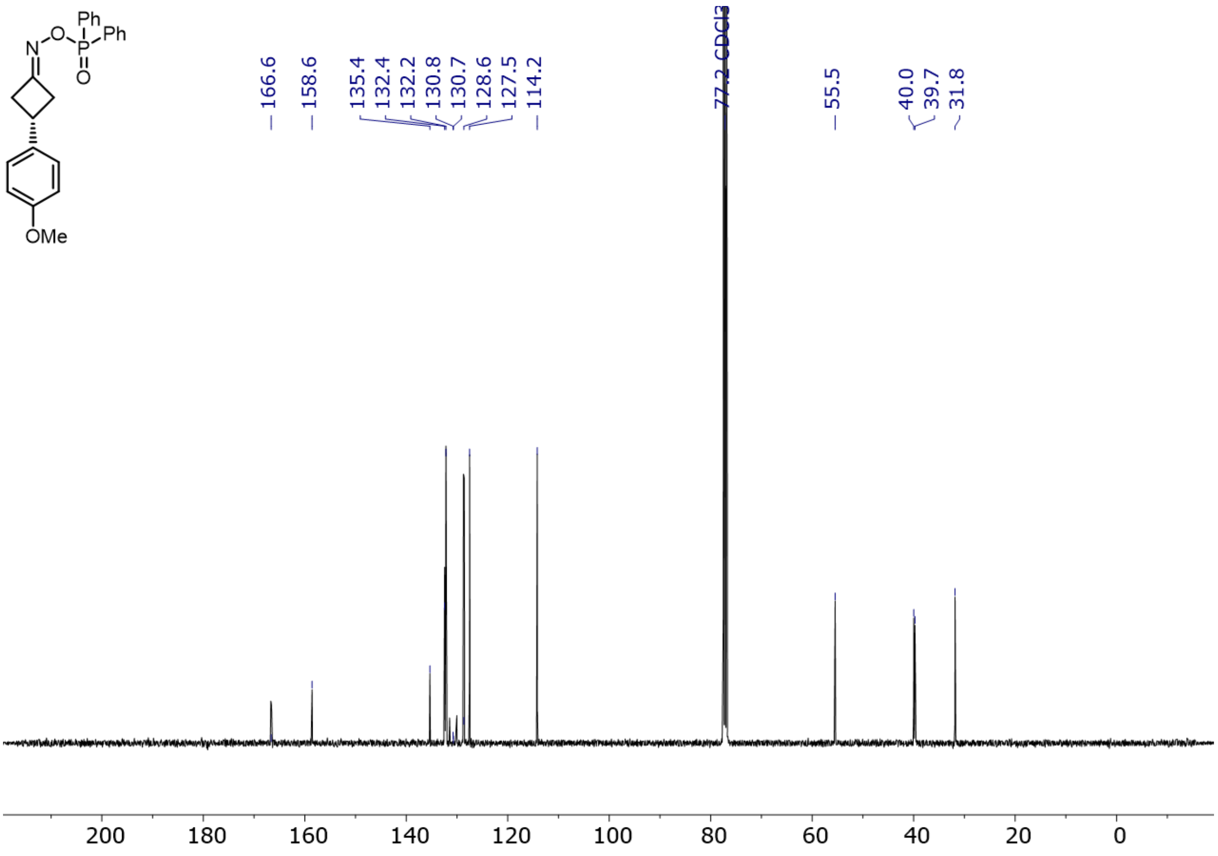

[6c] ^{31}P, $CDCl_3$, 162 MHz

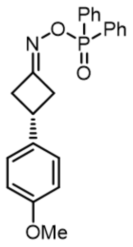

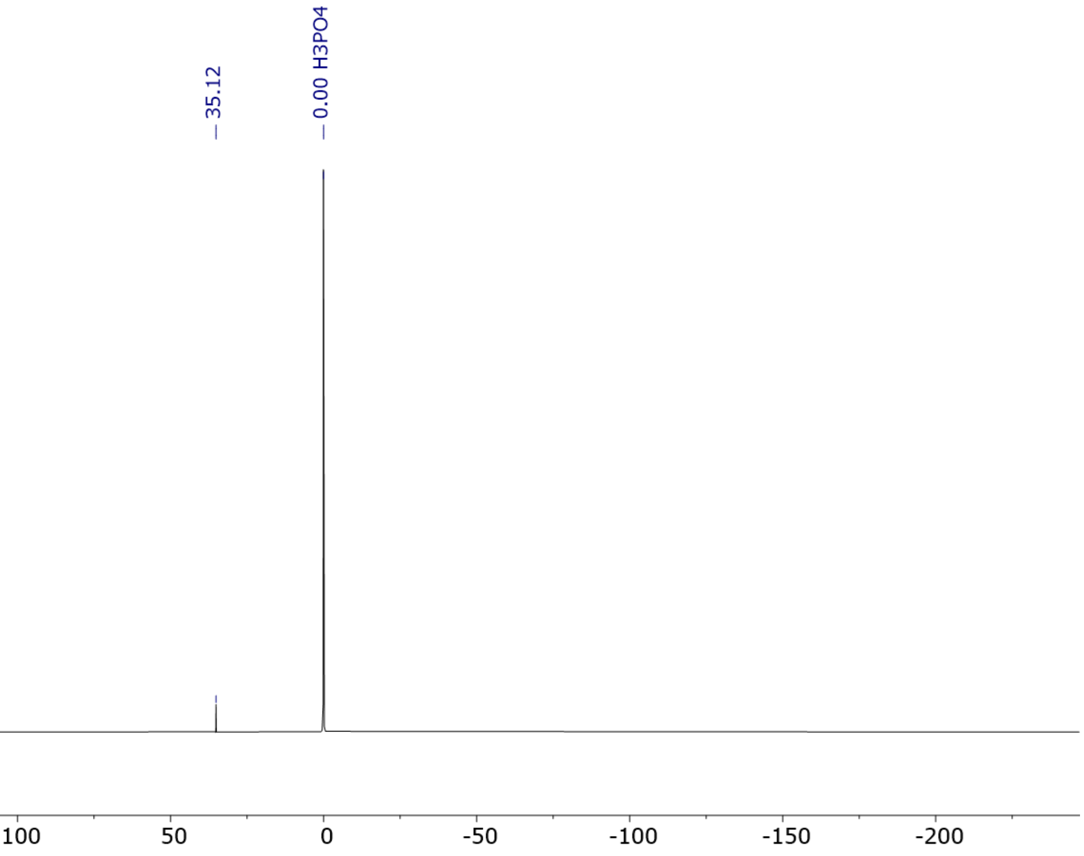

[6d] ^{1}H, $CDCl_3$, 400 MHz

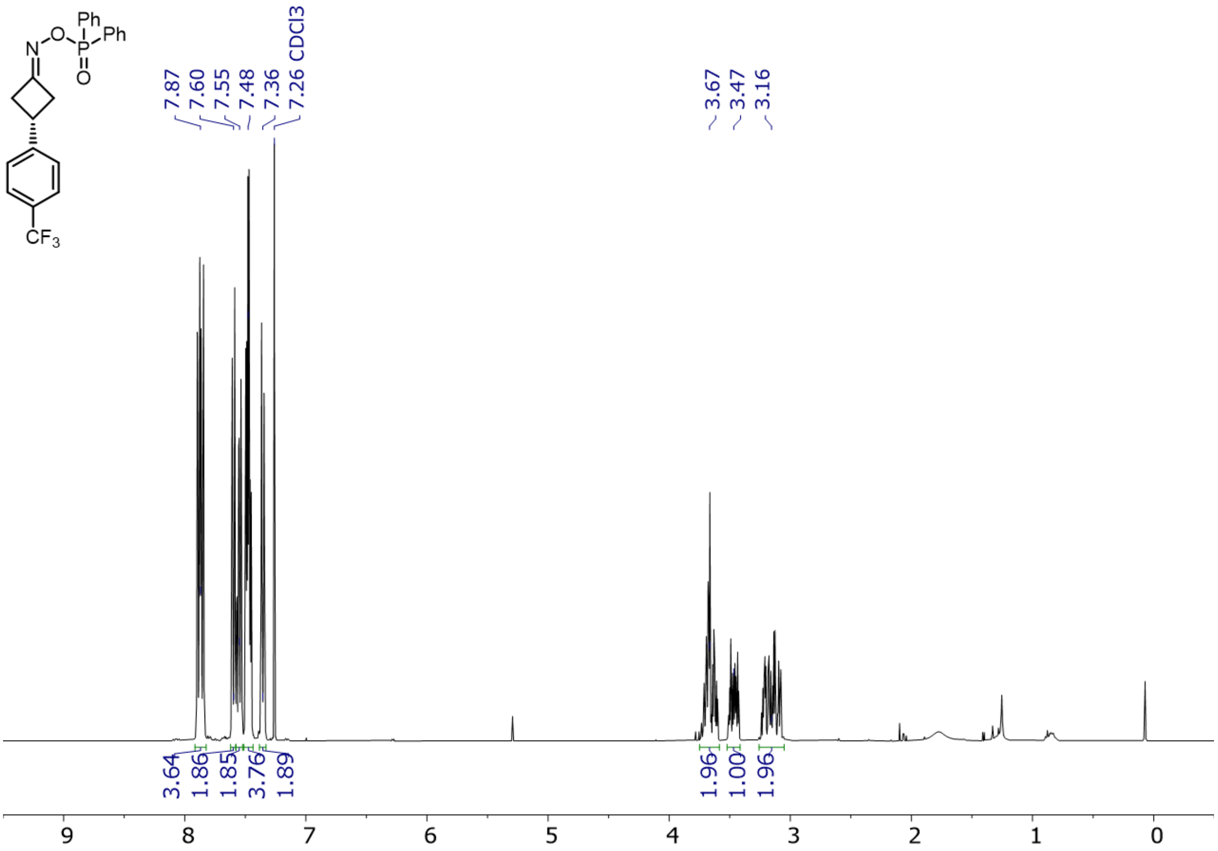

[6d] ^{13}C, $CDCl_3$, 101 MHz

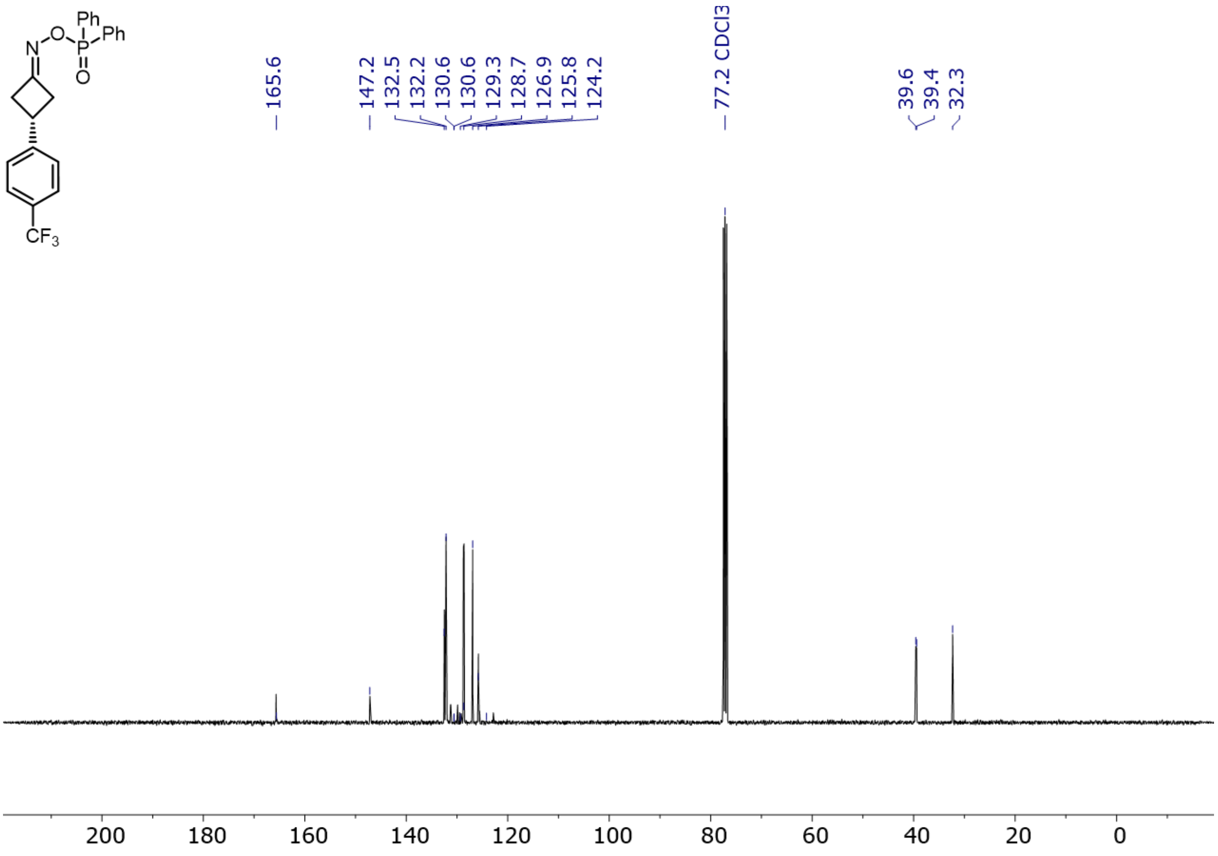

[6d] ^{31}P, $CDCl_3$, 162 MHz

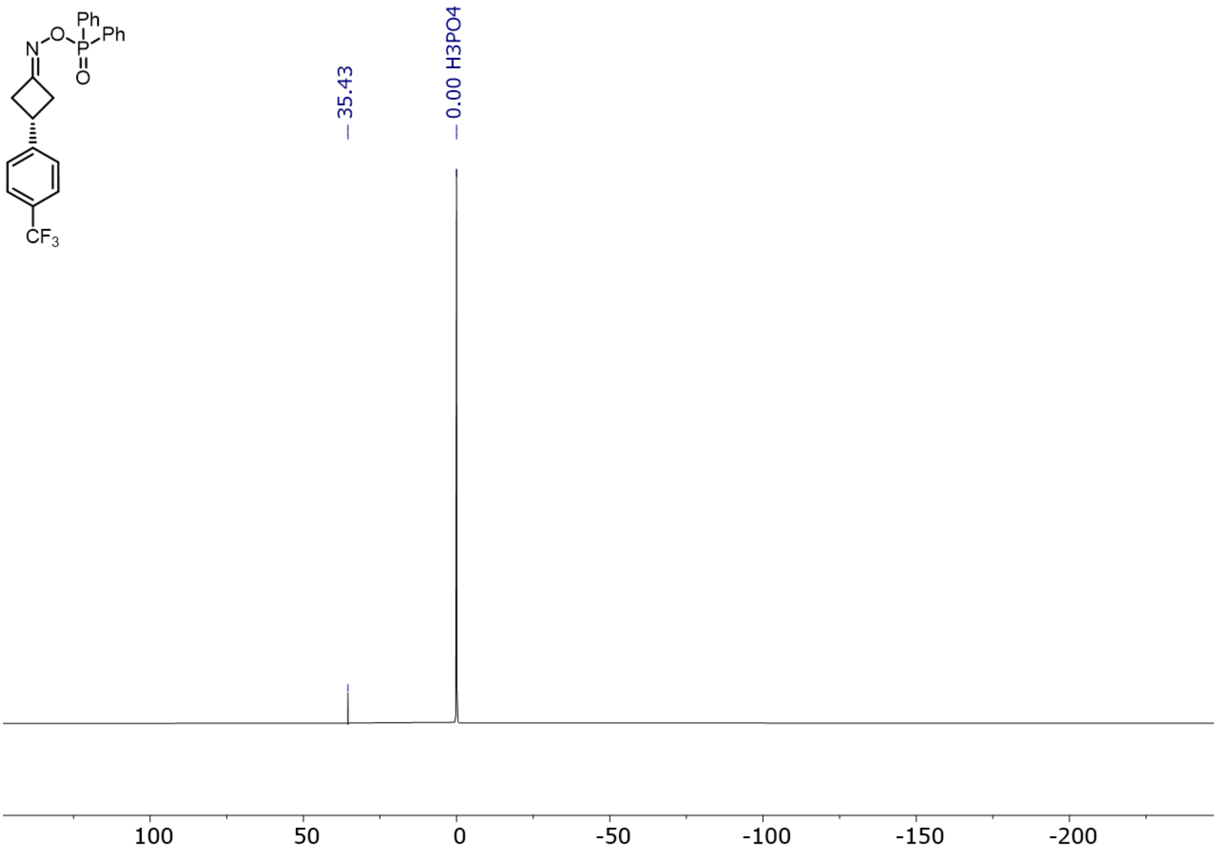

[6d] ^{19}F, $CDCl_3$, 376 MHz

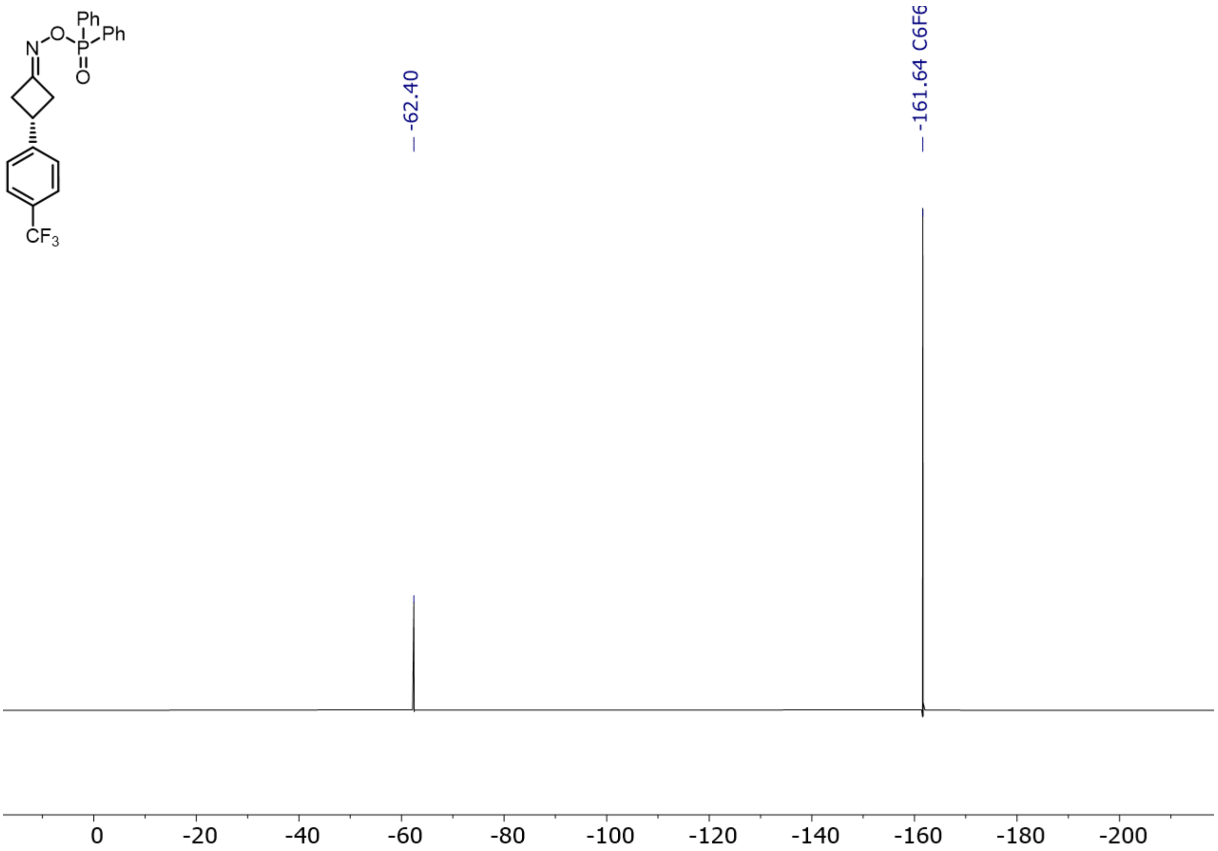

[6e] ^{1}H, $CDCl_3$, 400 MHz

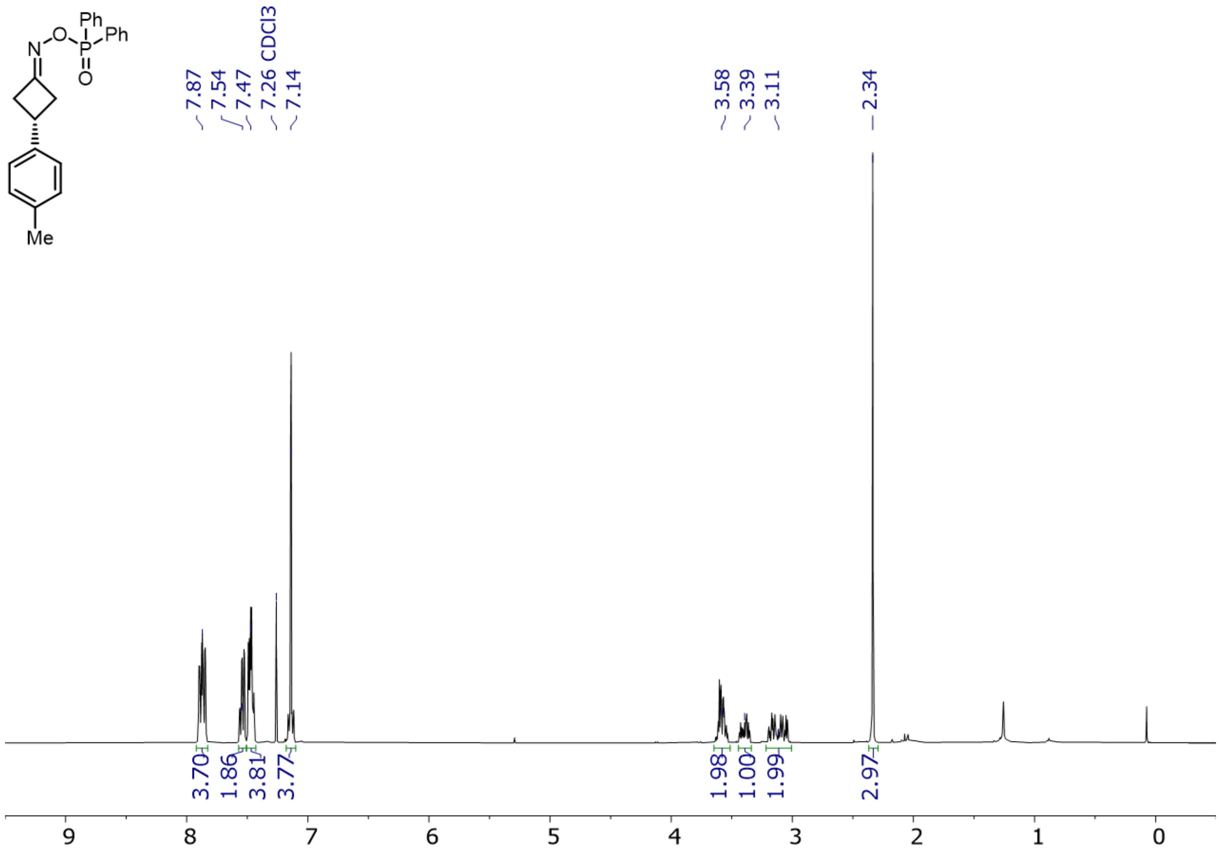

[6e] ^{13}C, $CDCl_3$, 101 MHz

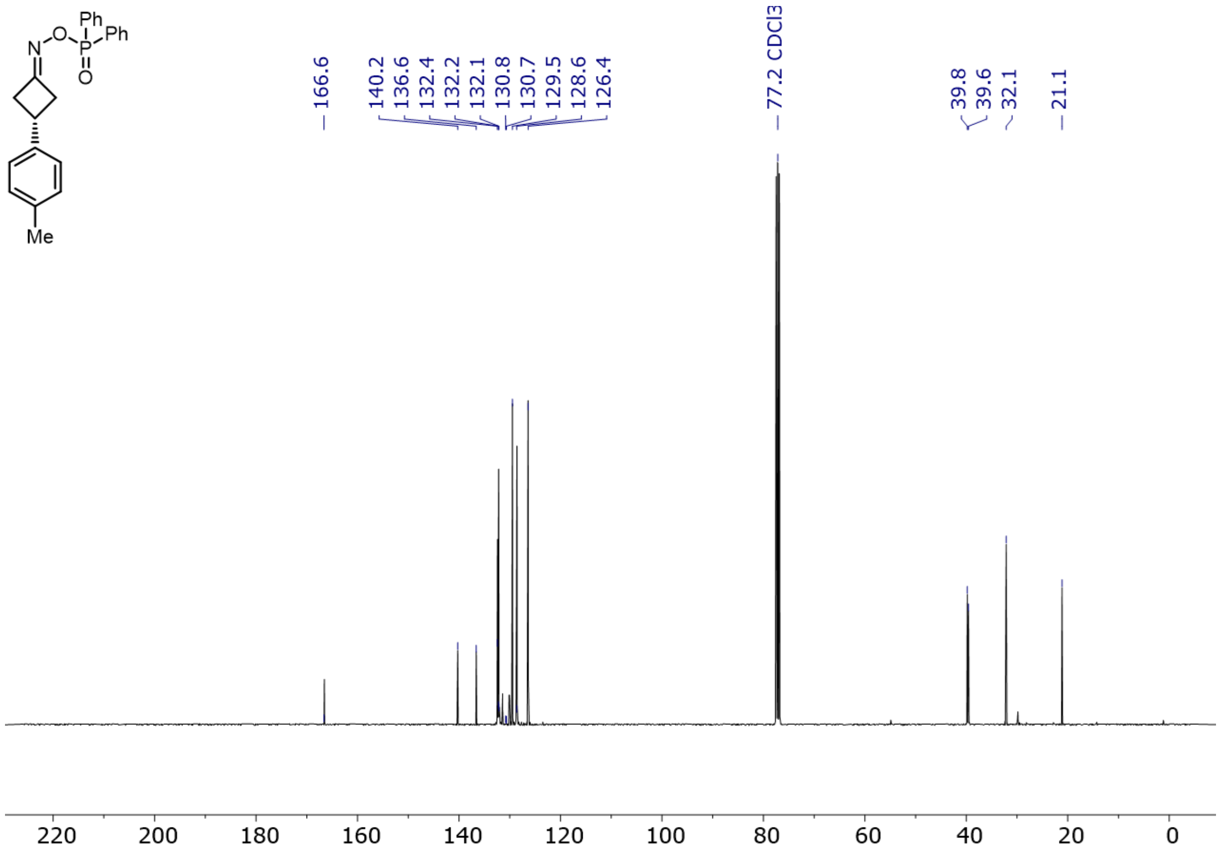

[6e] ^{31}P, $CDCl_3$, 162 MHz

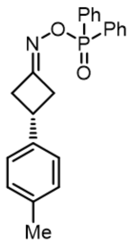

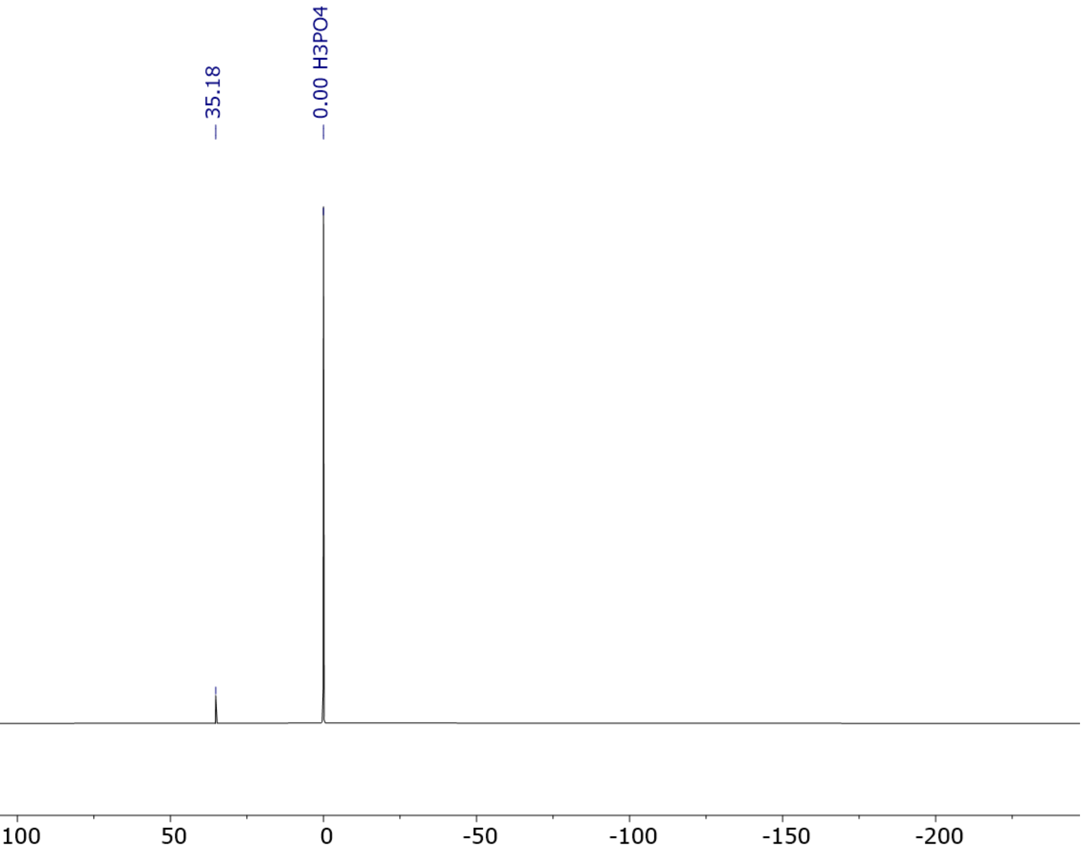

[6f] ^{1}H, $CDCl_3$, 400 MHz

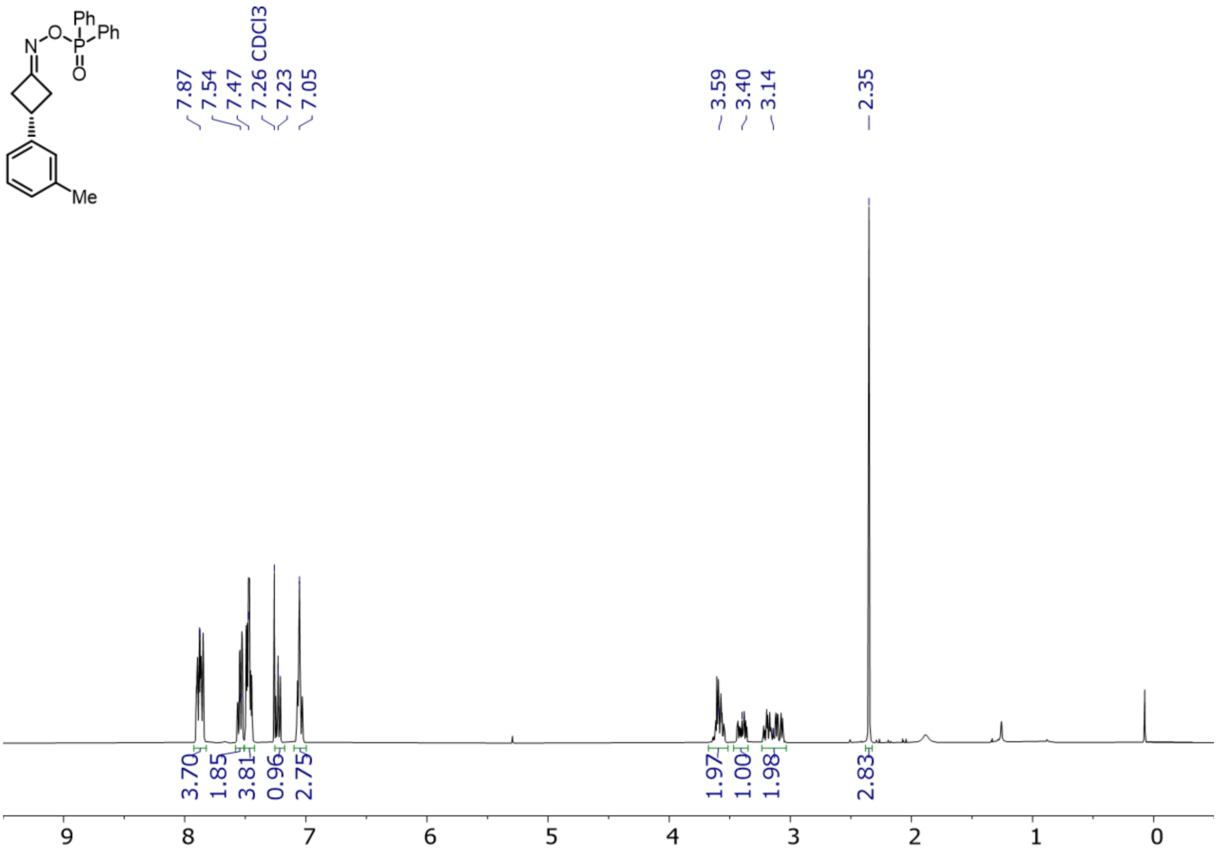

[6f] ^{13}C, $CDCl_3$, 101 MHz

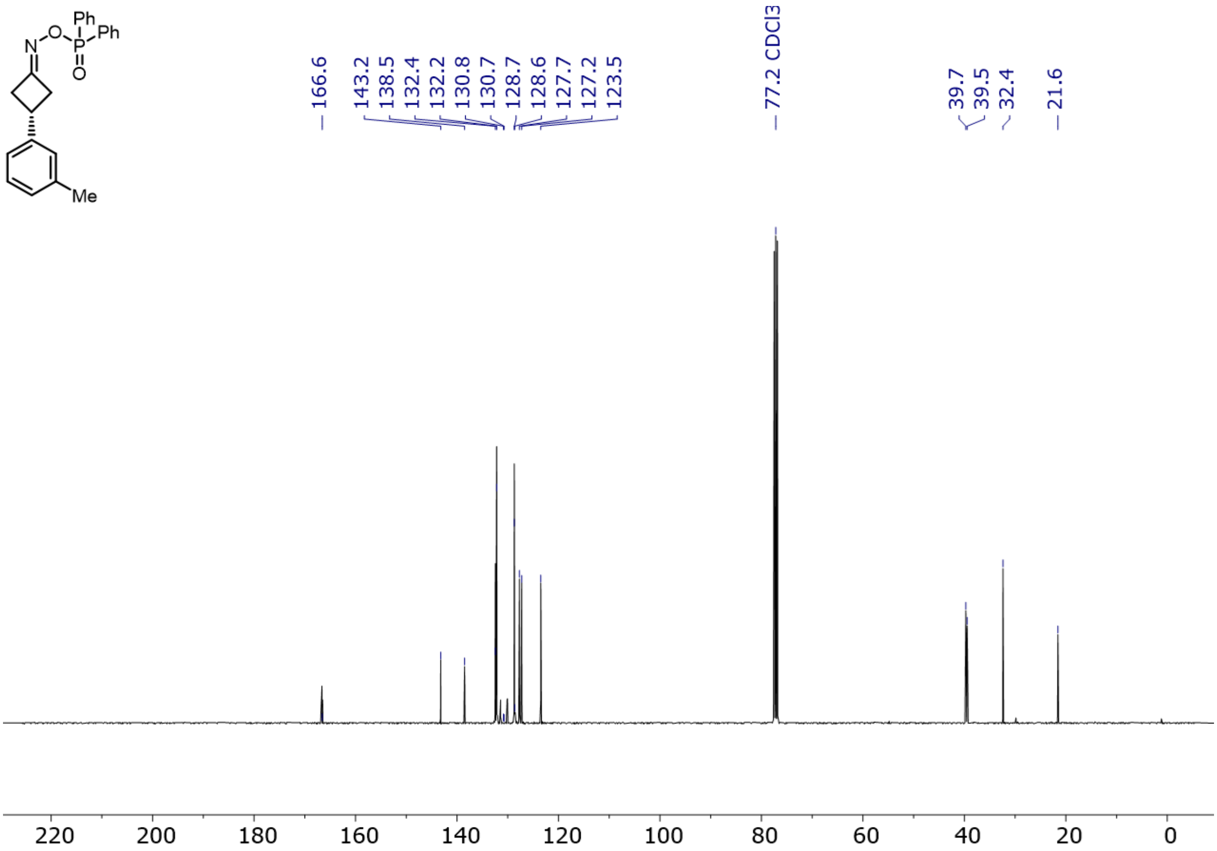

[6f] ^{31}P, $CDCl_3$, 162 MHz

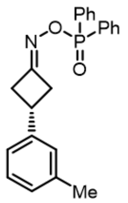

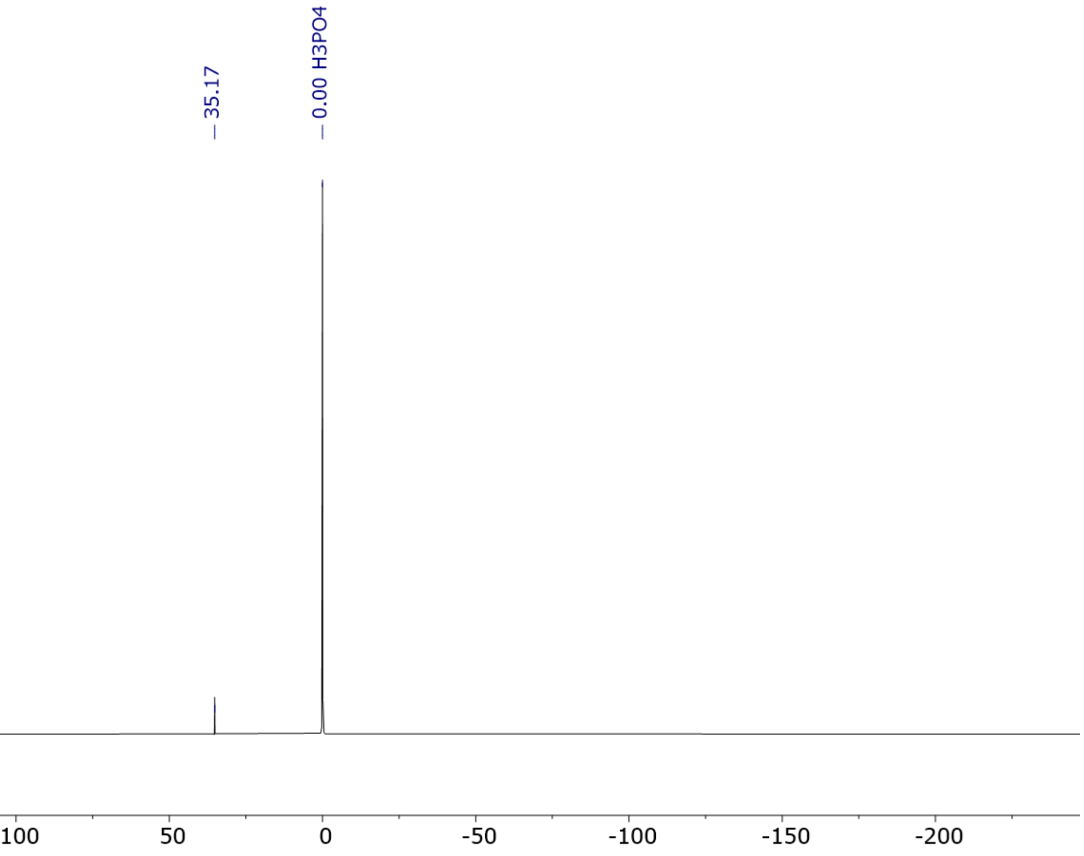

[6g] ^{1}H, $CDCl_3$, 400 MHz

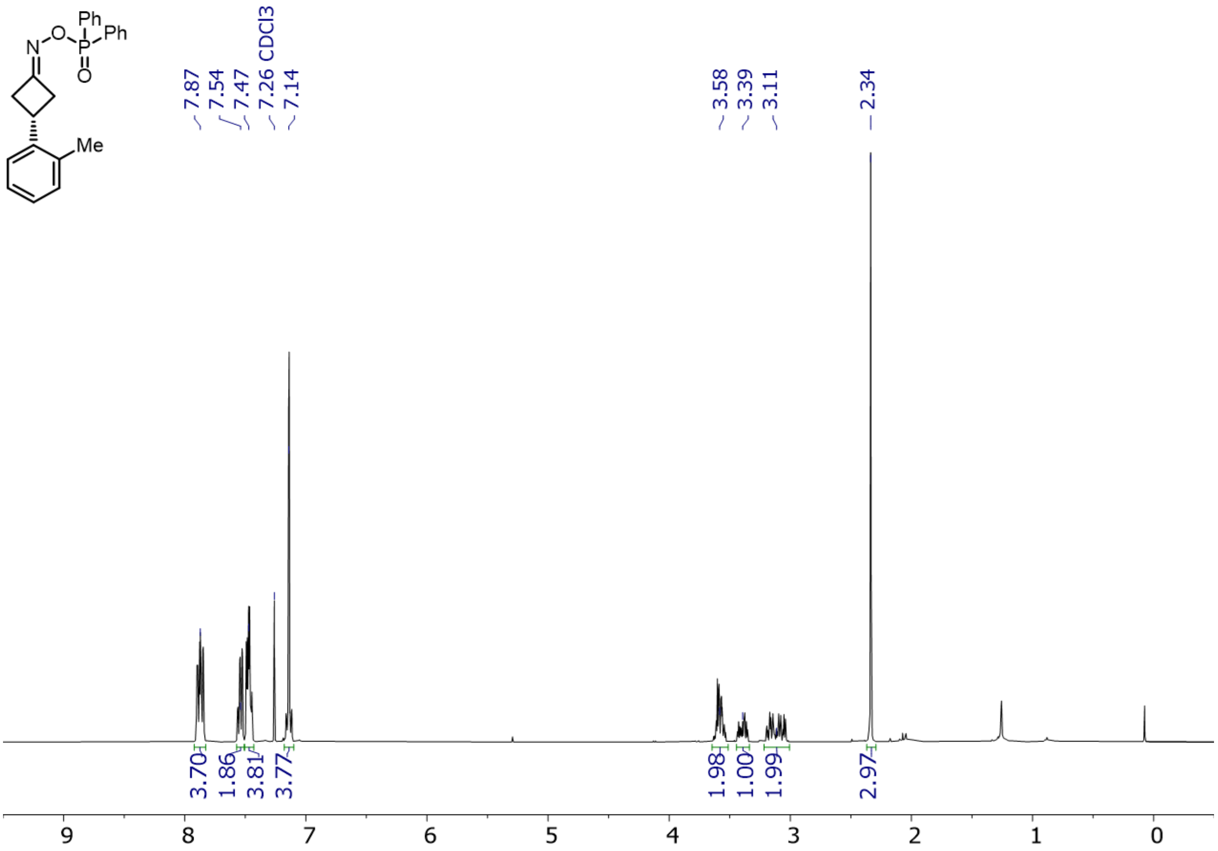

[6g] ^{13}C, $CDCl_3$, 101 MHz

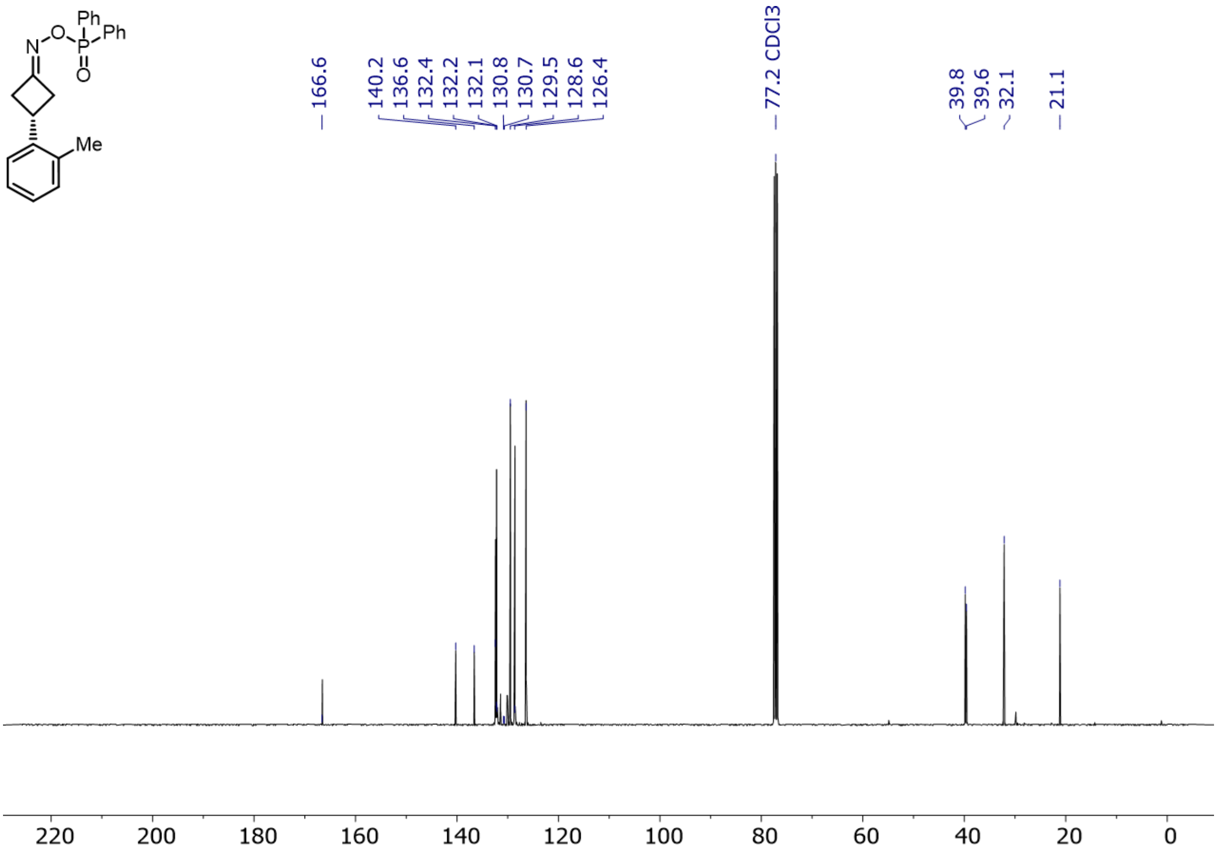

[6g] ^{31}P, $CDCl_3$, 162 MHz

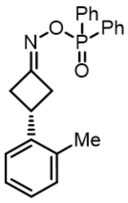

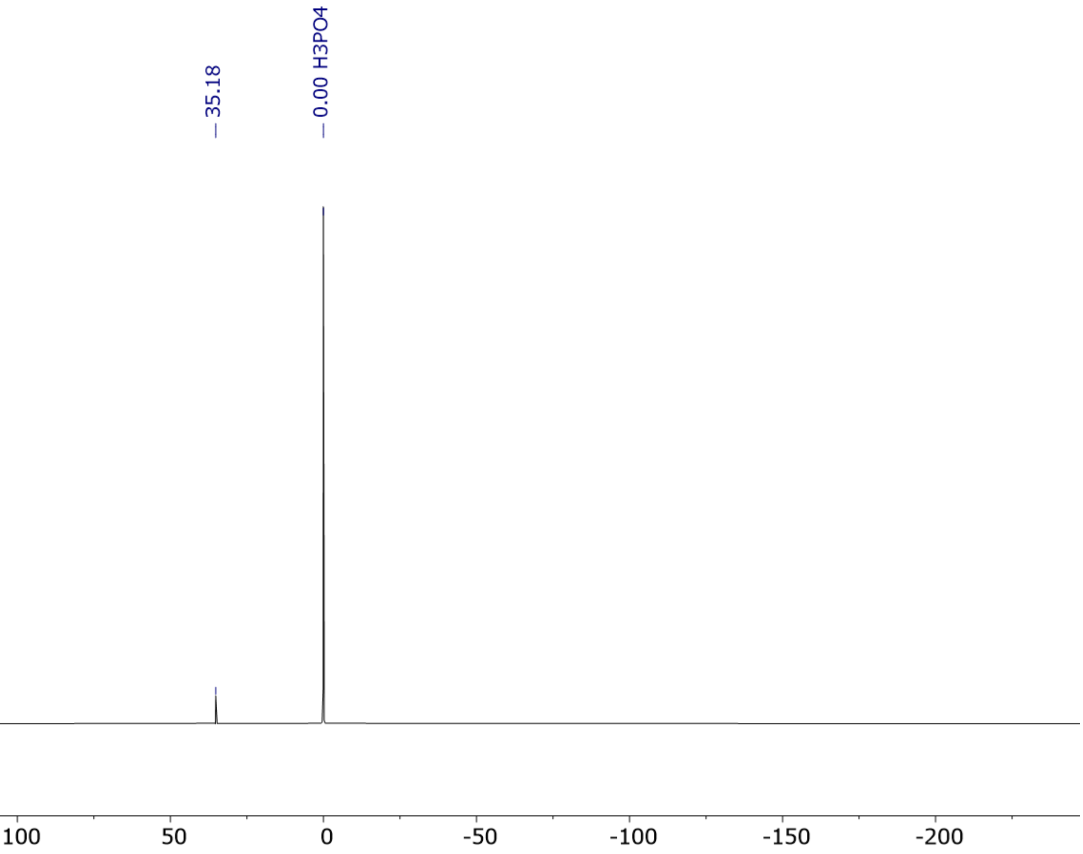

[6h] ^{1}H, $CDCl_3$, 400 MHz

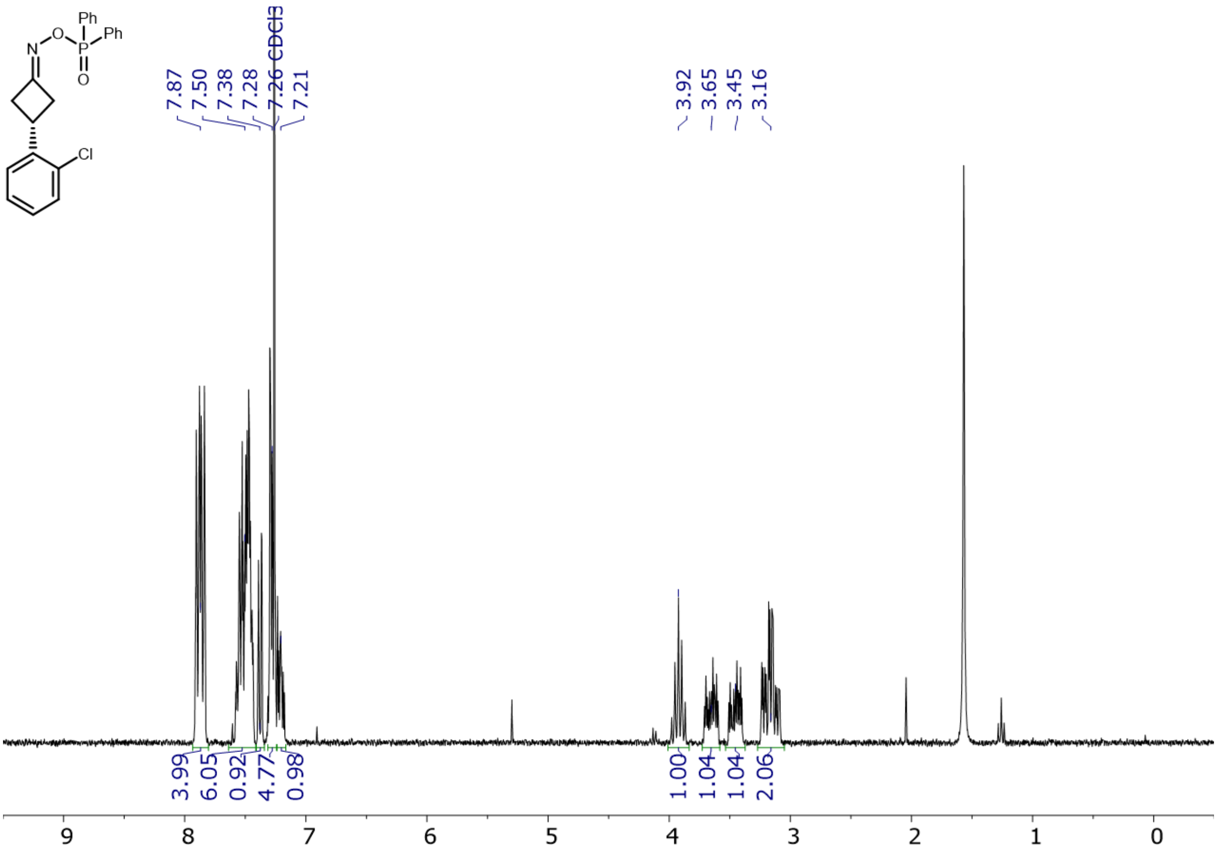

[6h] ^{13}C, $CDCl_3$, 101 MHz

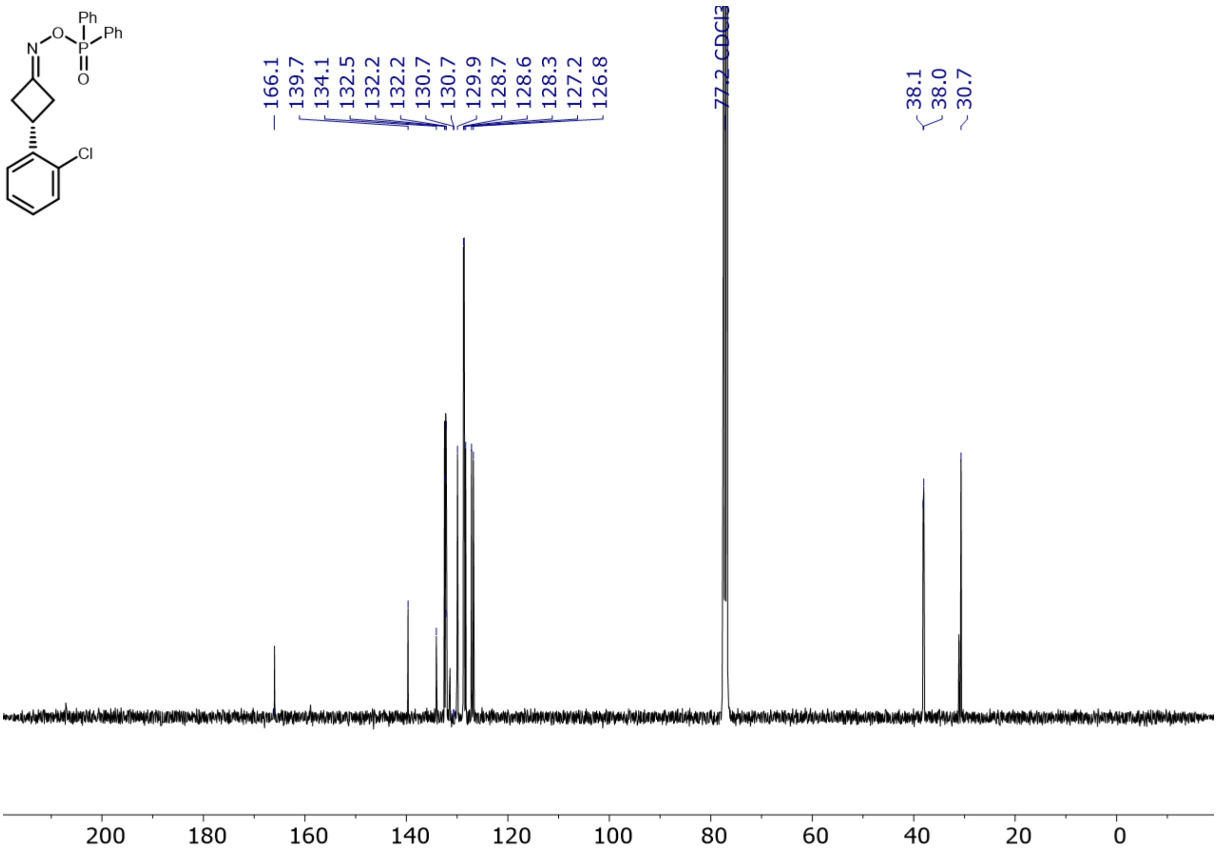

[6h] ^{31}P, $CDCl_3$, 162 MHz

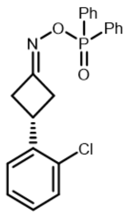

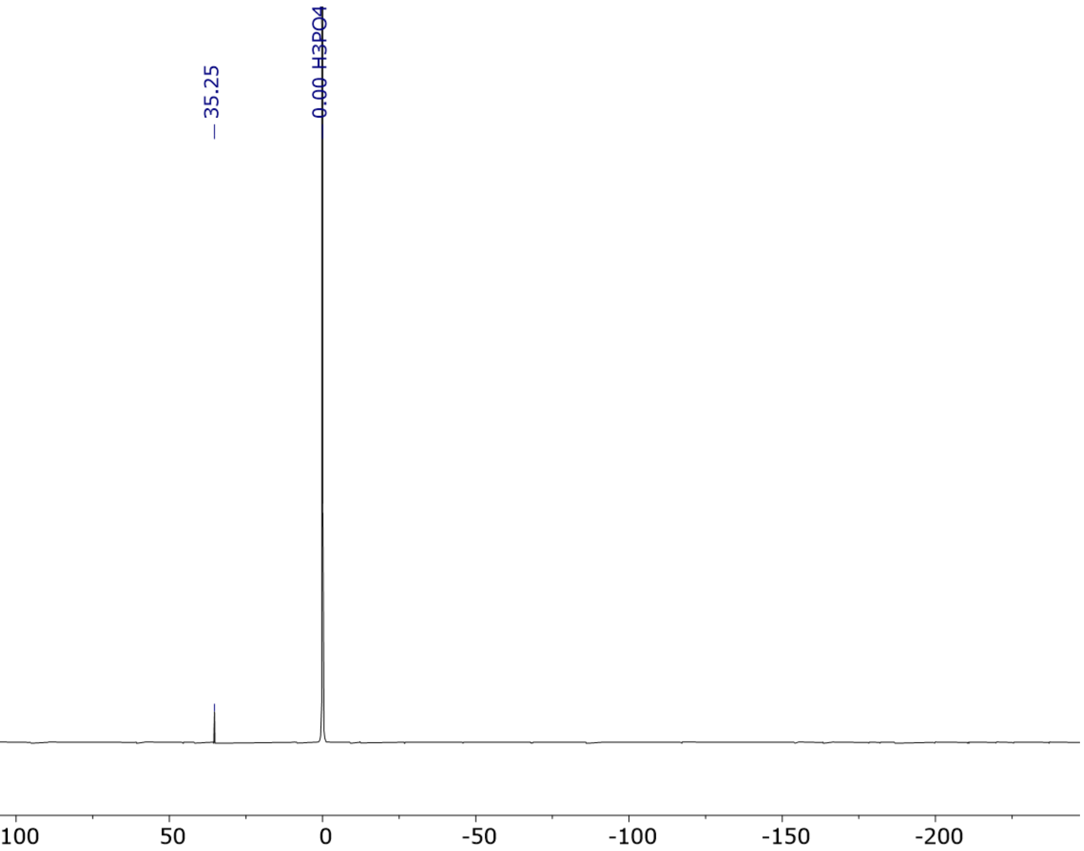

[6i] ^1H, $CDCl_3$, 400 MHz

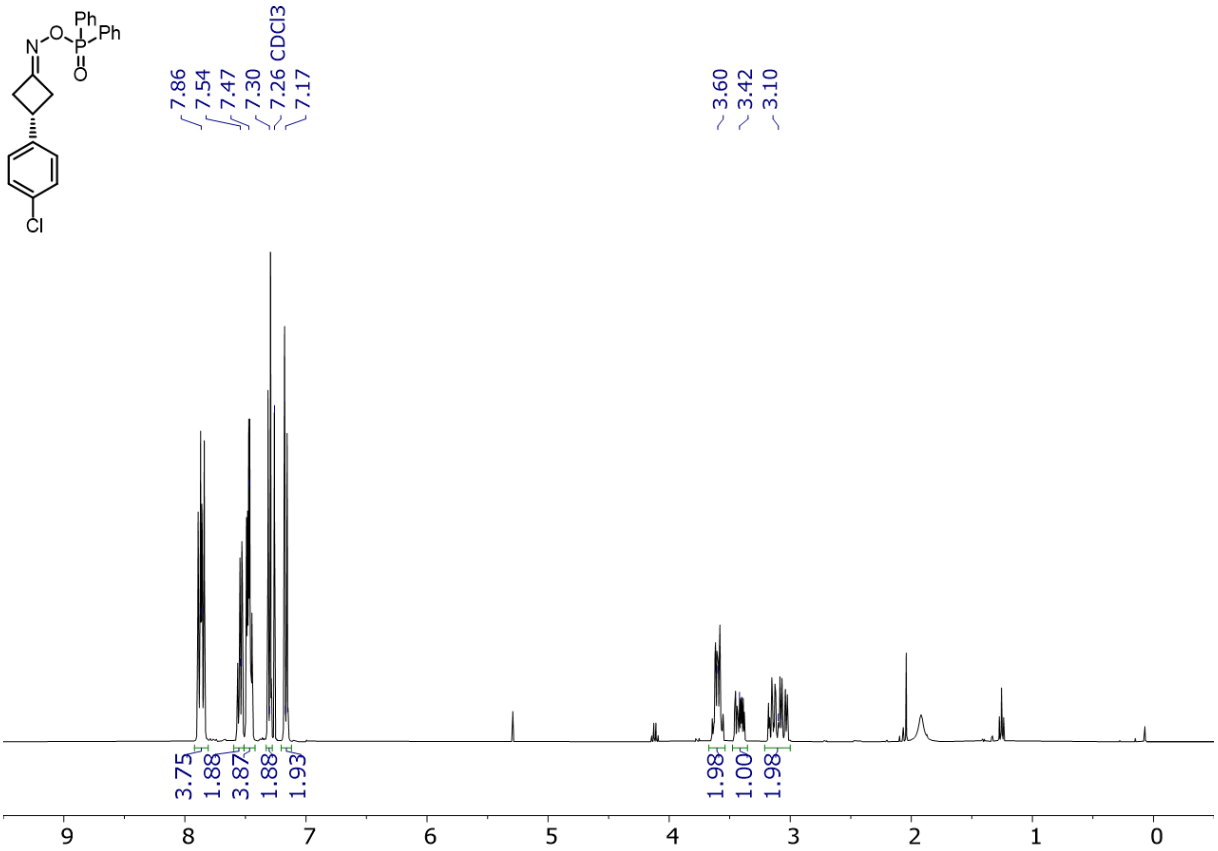

[6i] ^{13}C, $CDCl_3$, 101 MHz

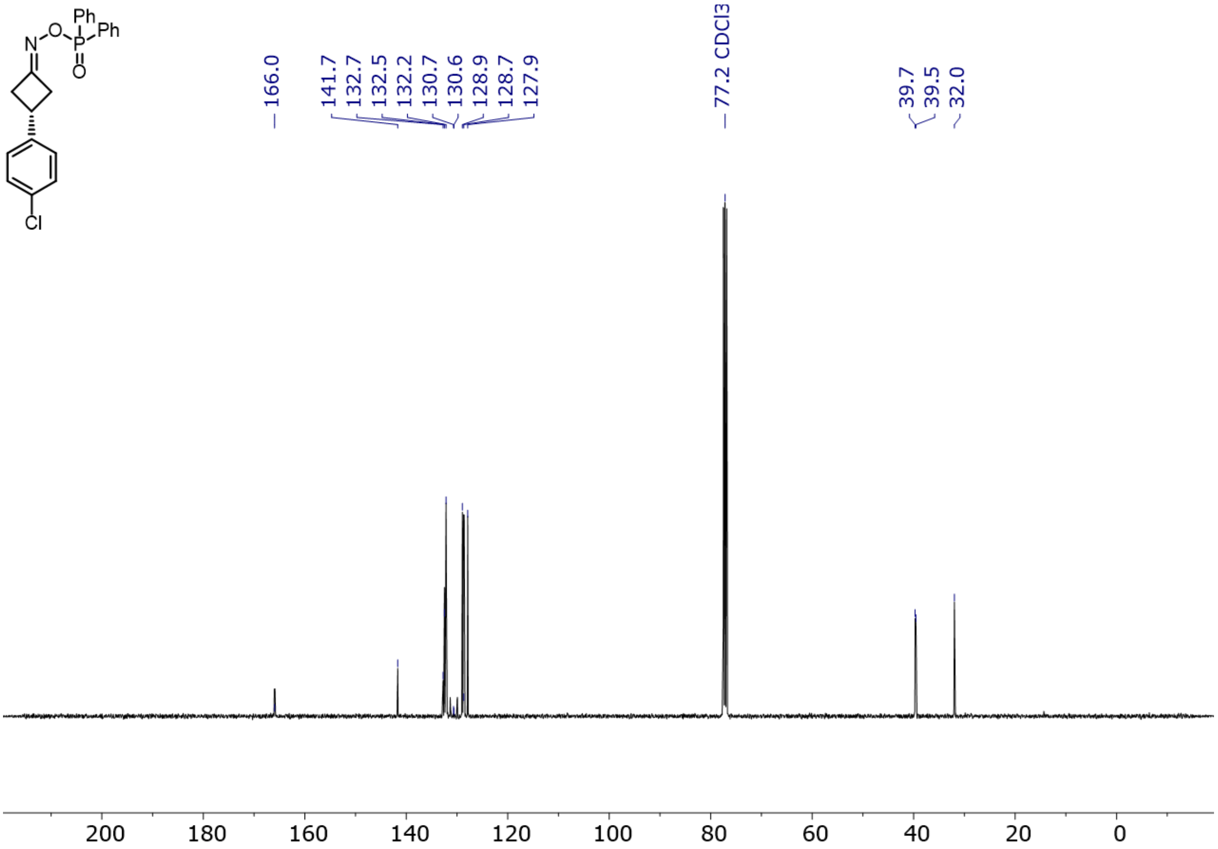

[6i] ^{31}P, $CDCl_3$, 162 MHz

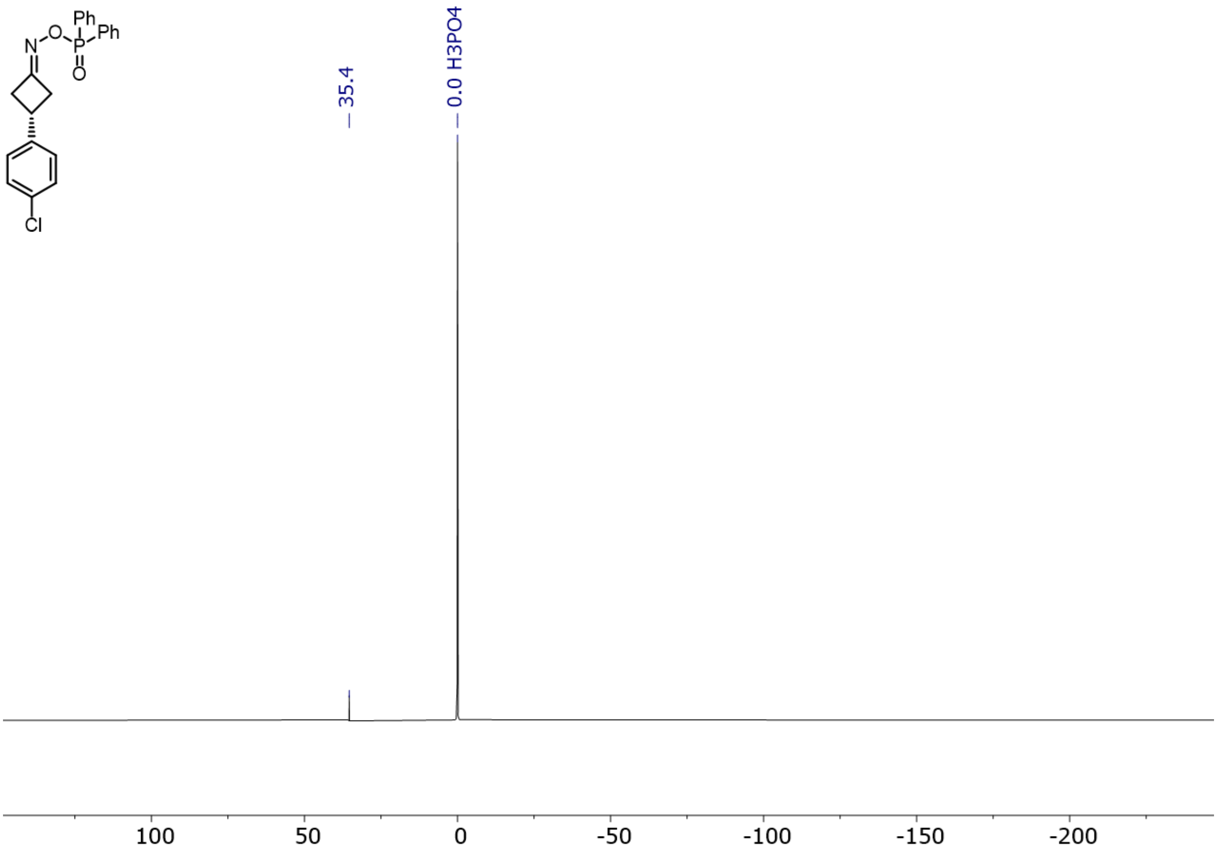

[6j] ^{1}H, $CDCl_3$, 400 MHz

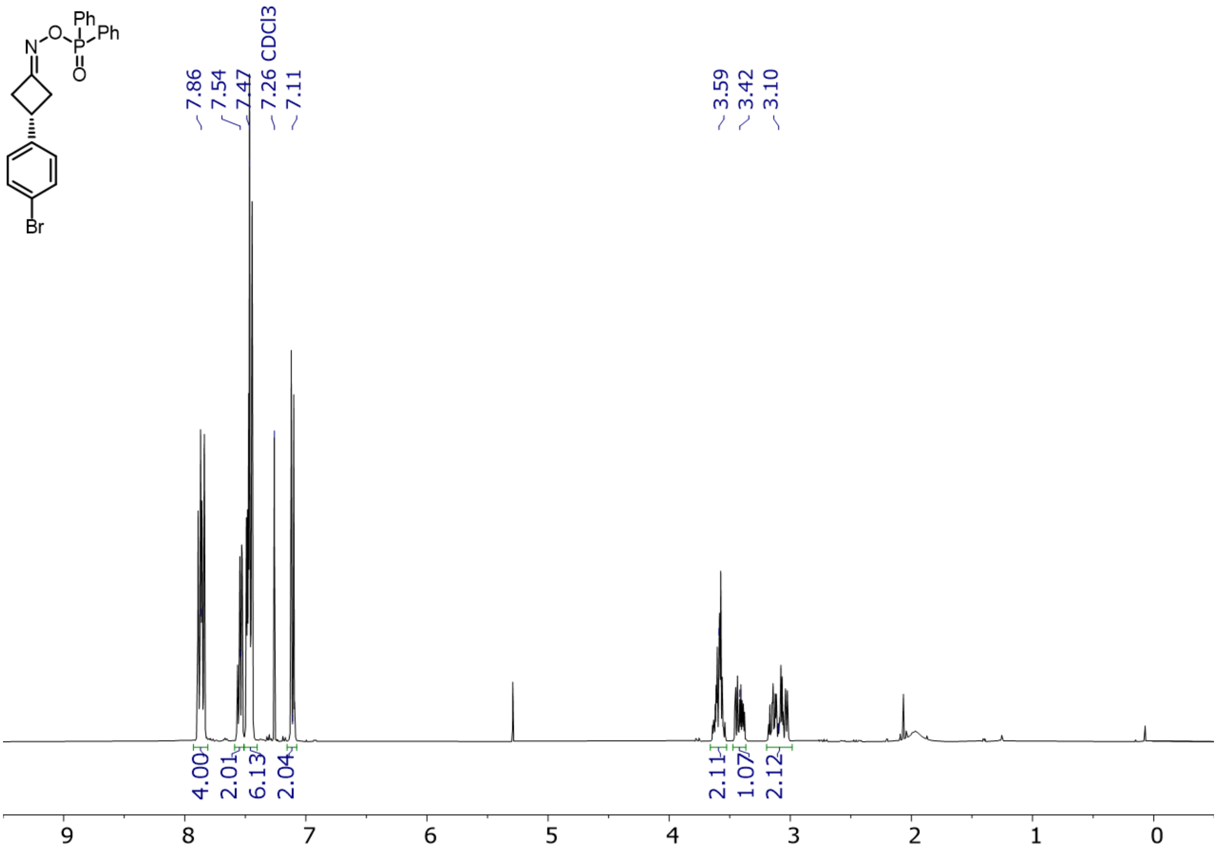

[6j] ^{13}C, $CDCl_3$, 101 MHz

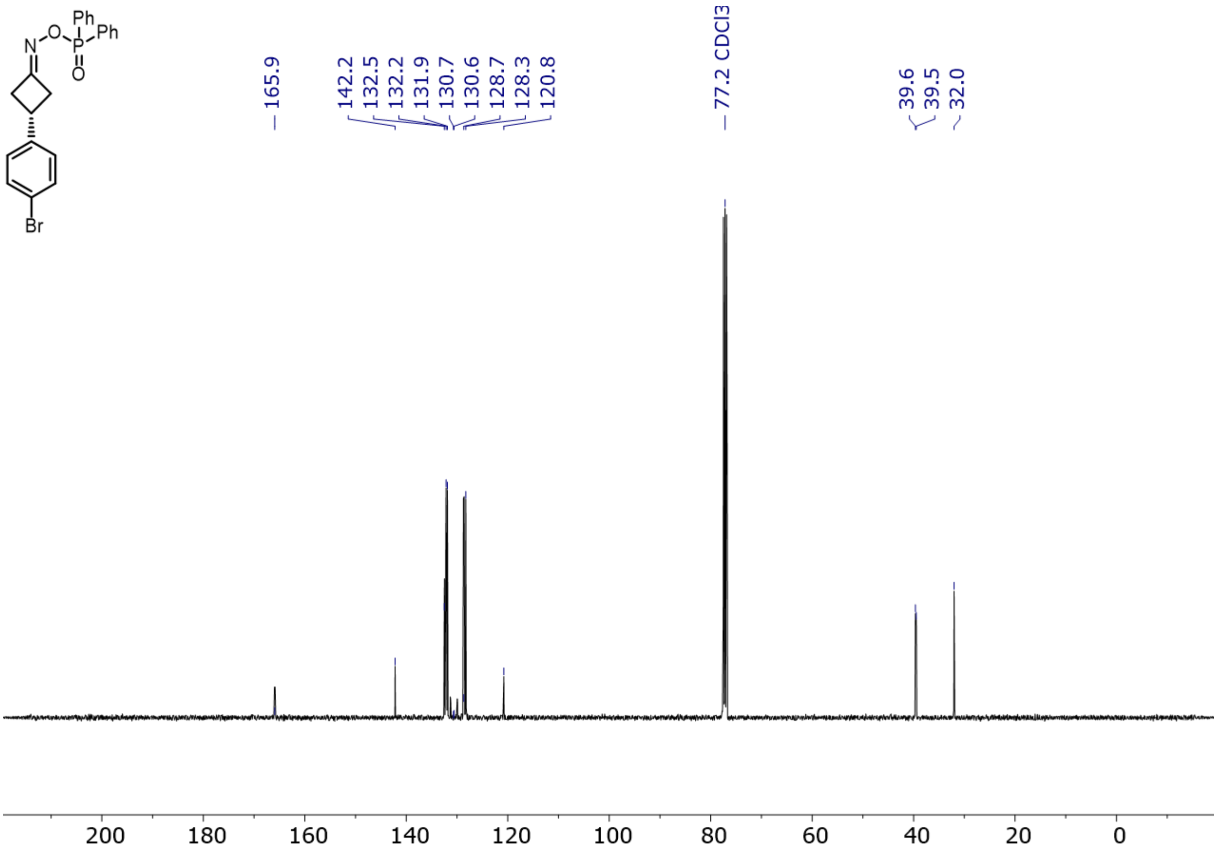

[6j] ^{31}P, $CDCl_3$, 162 MHz

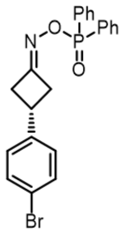

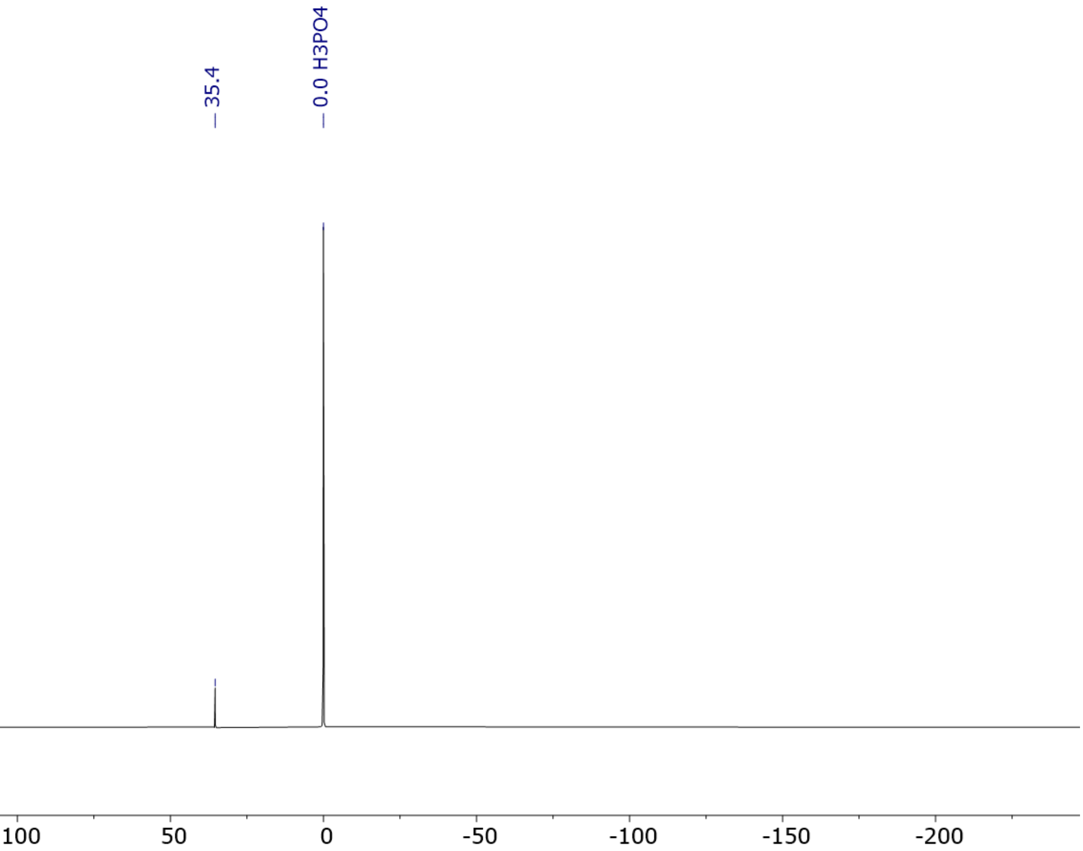

[6k] ^1H, $CDCl_3$, 400 MHz

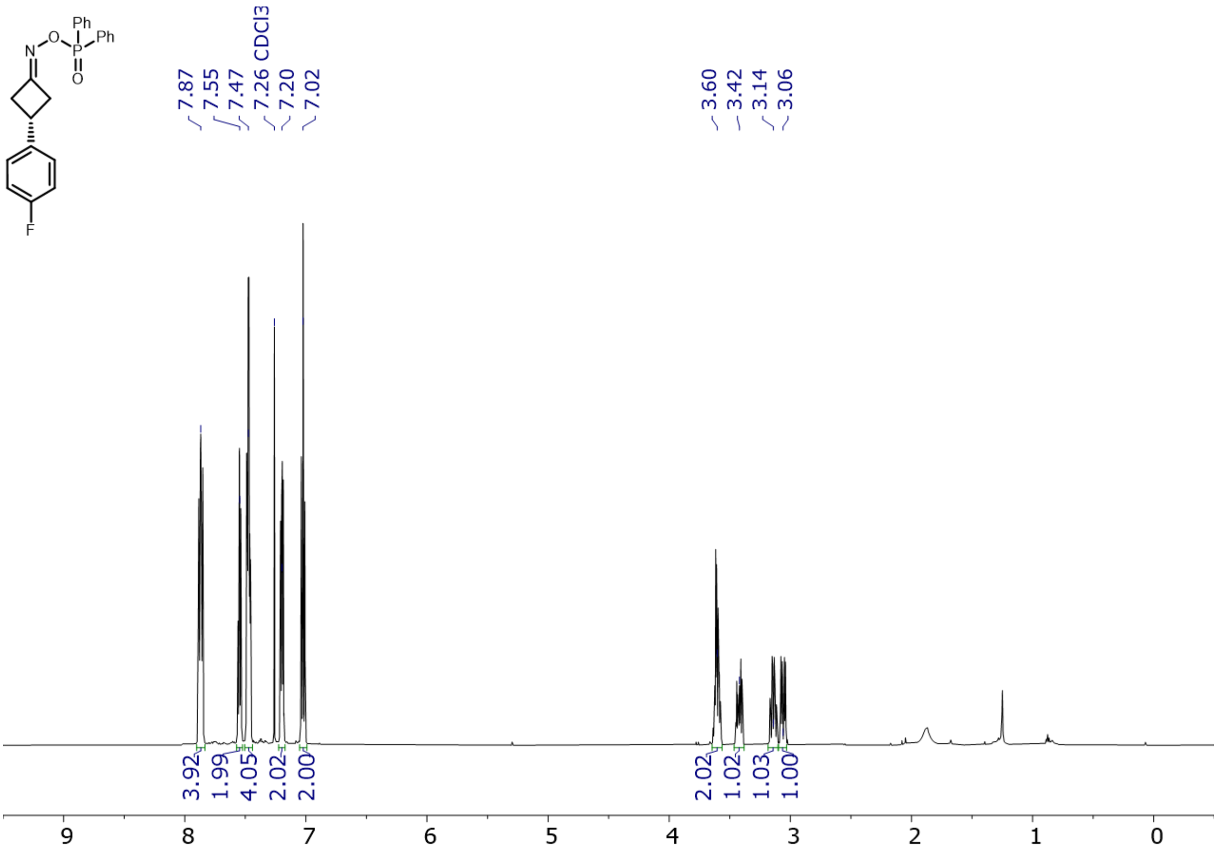

[6k] ^{13}C, $CDCl_3$, 101 MHz

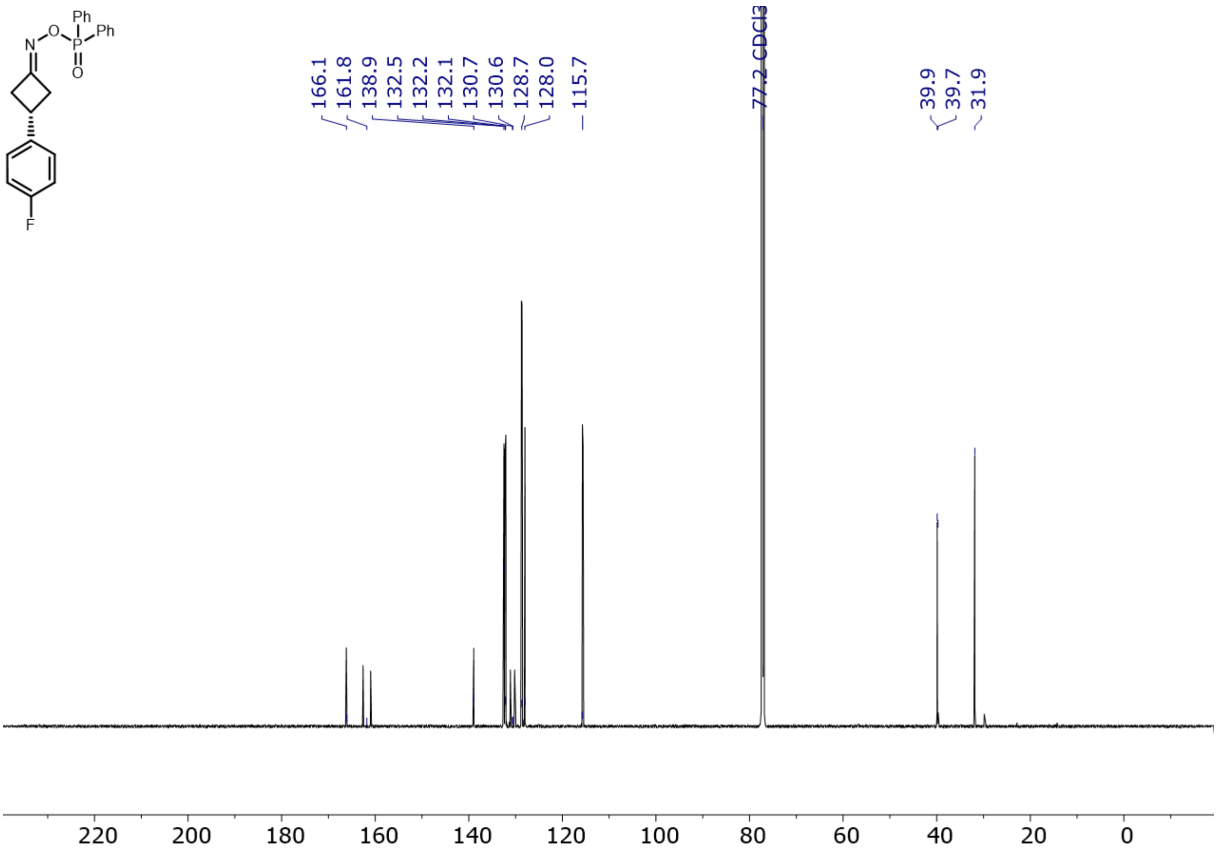

[6k] ^{31}P, $CDCl_3$, 162 MHz

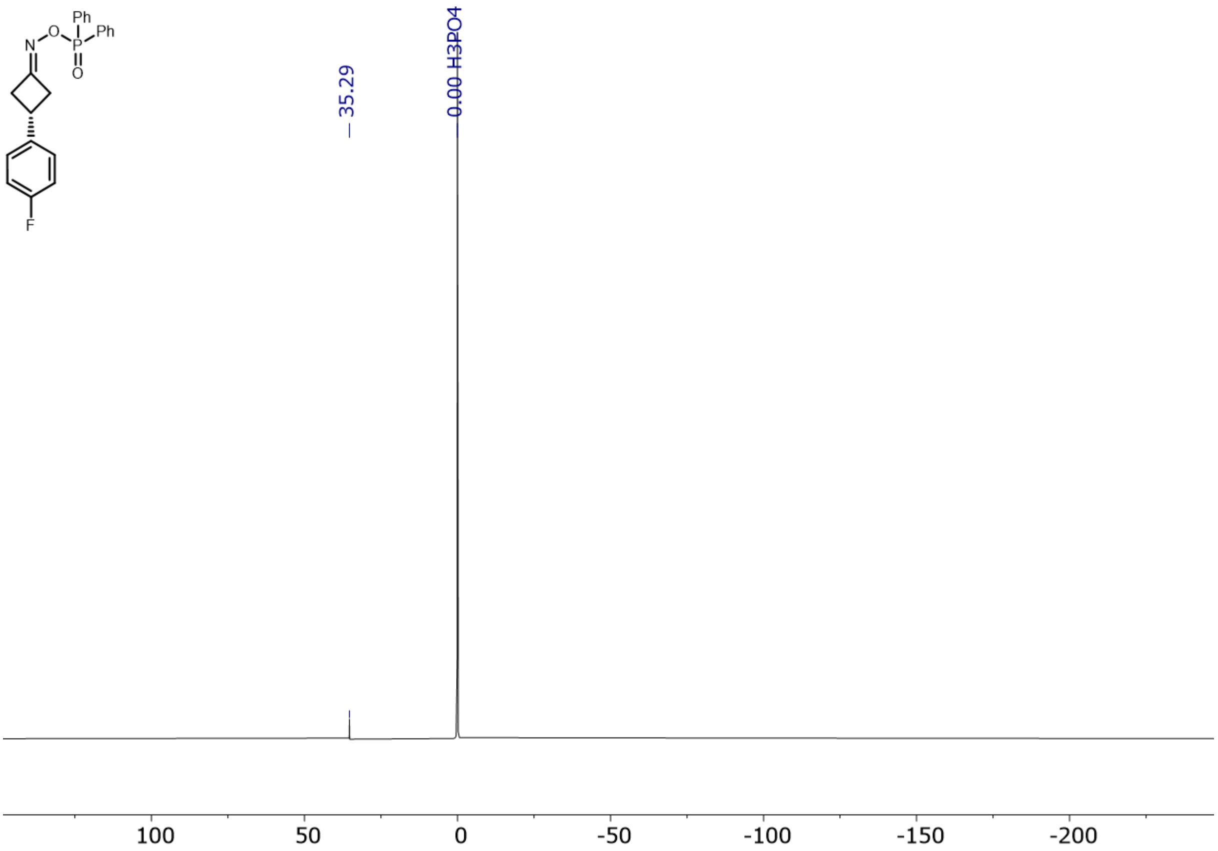

[6k] ^{19}F, $CDCl_3$, 376 MHz

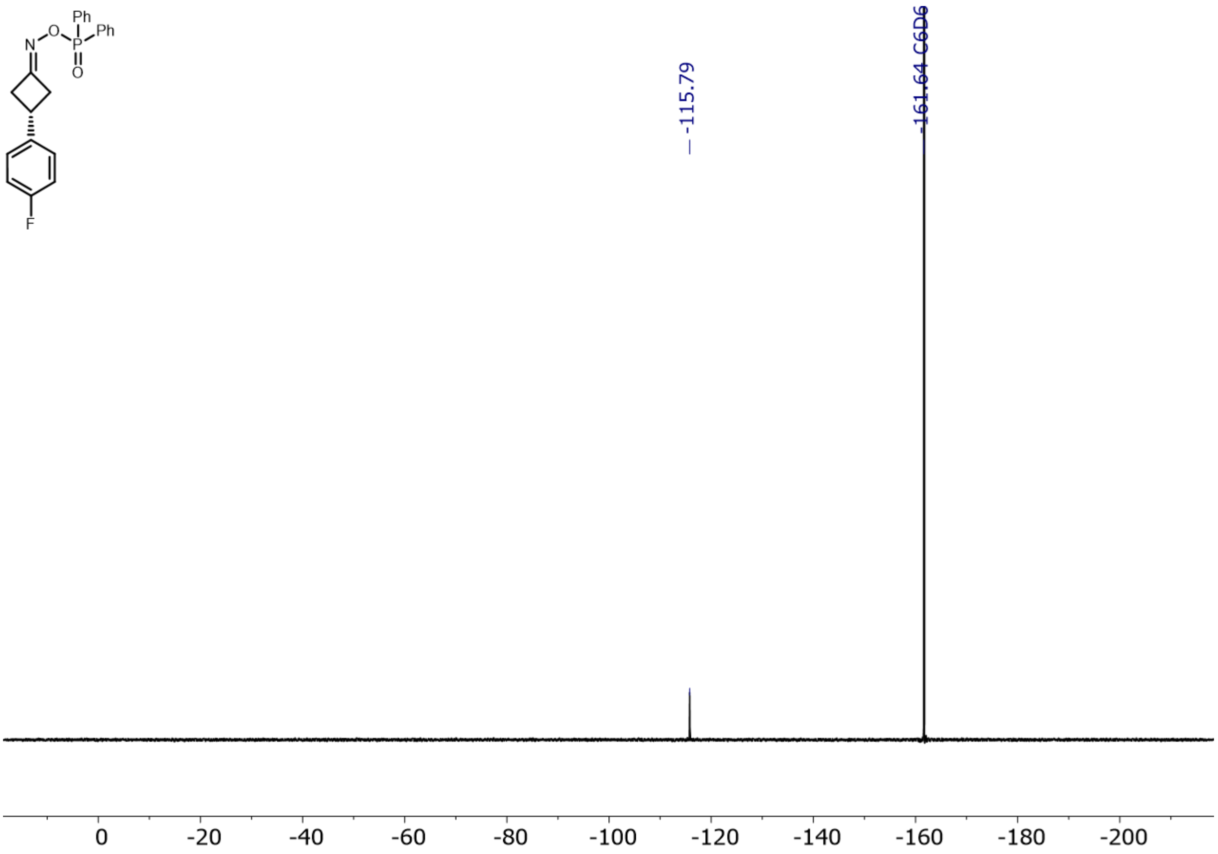

[6l] ^{1}H, $CDCl_3$, 400 MHz

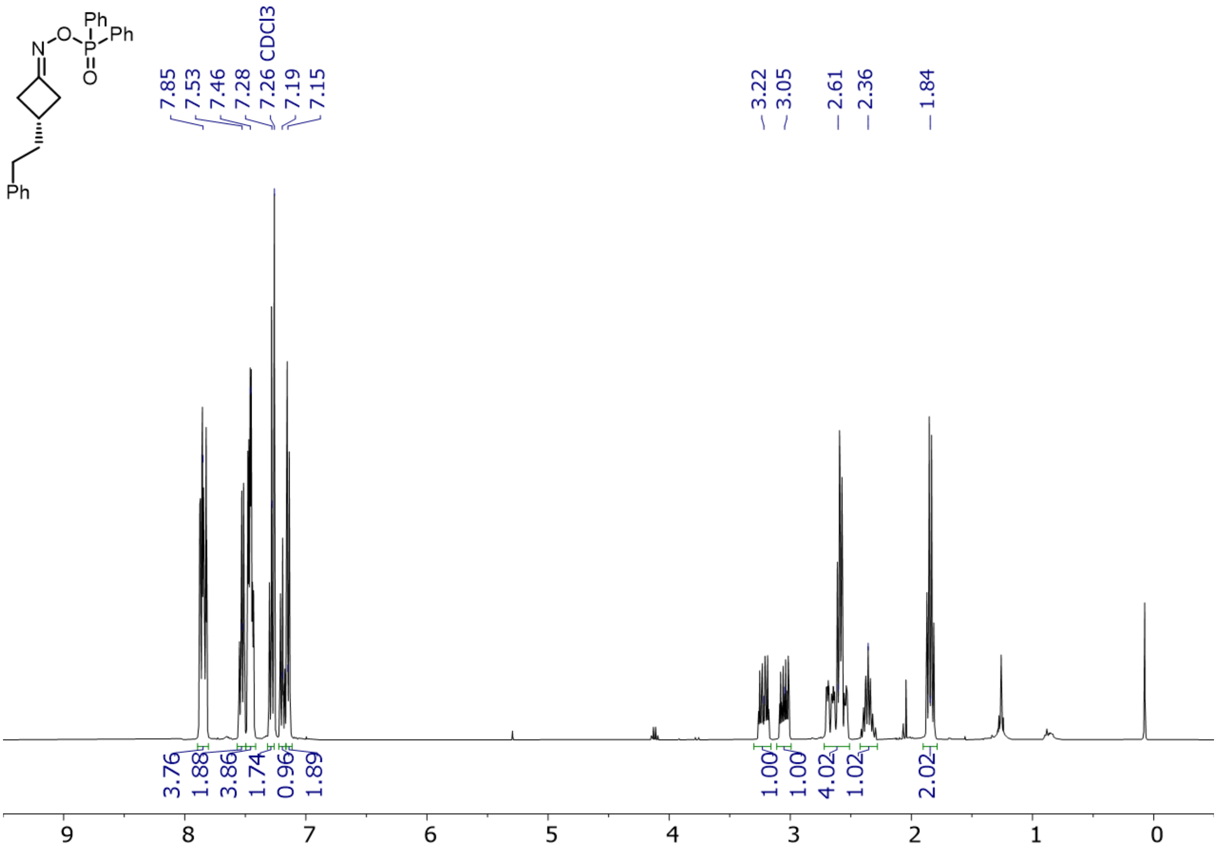

[6l] ^{13}C, $CDCl_3$, 101 MHz

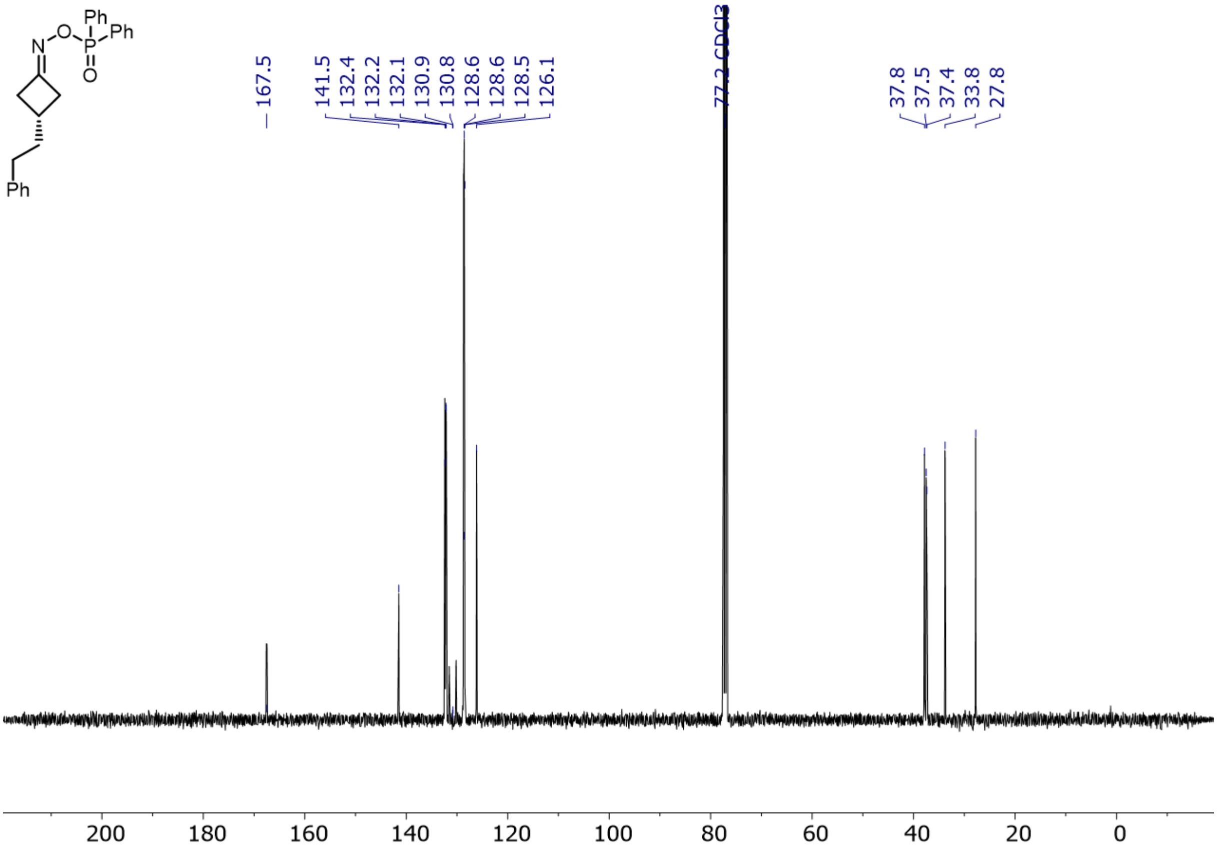

[6l] ^{31}P, $CDCl_3$, 162 MHz

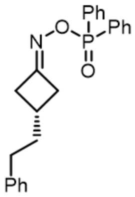

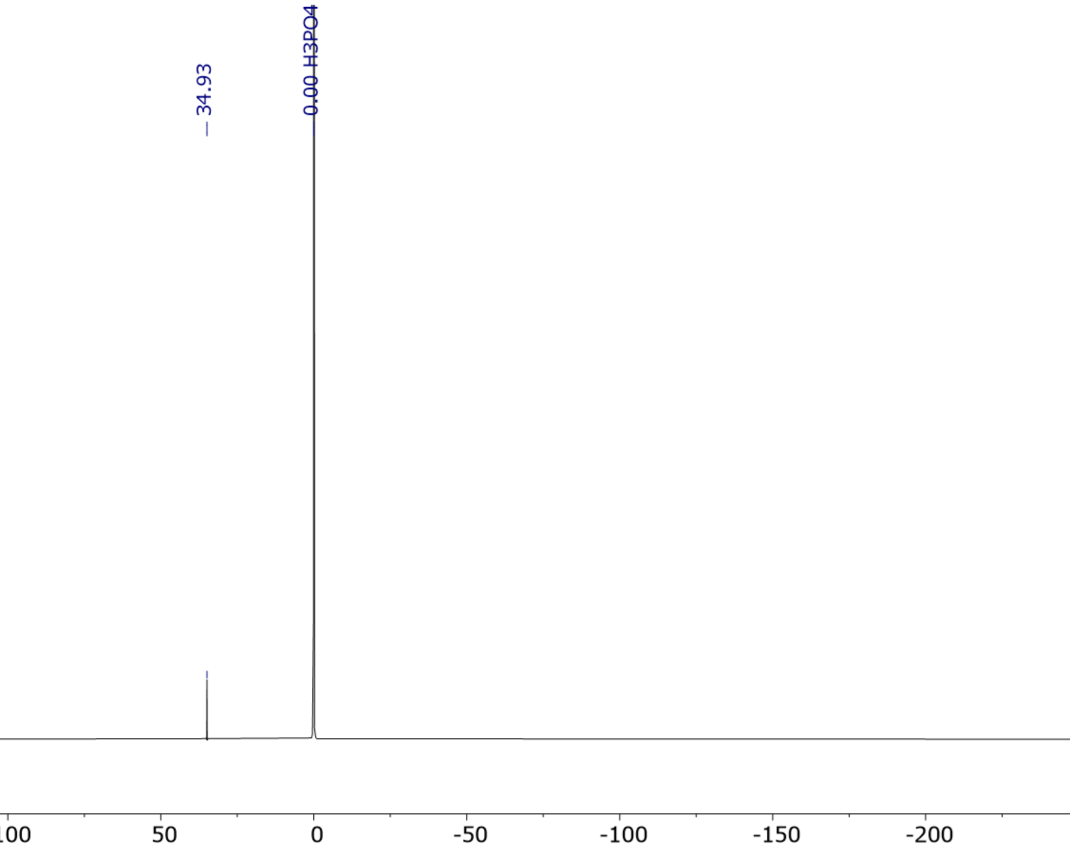

[6m] ^{1}H, $CDCl_3$, 400 MHz

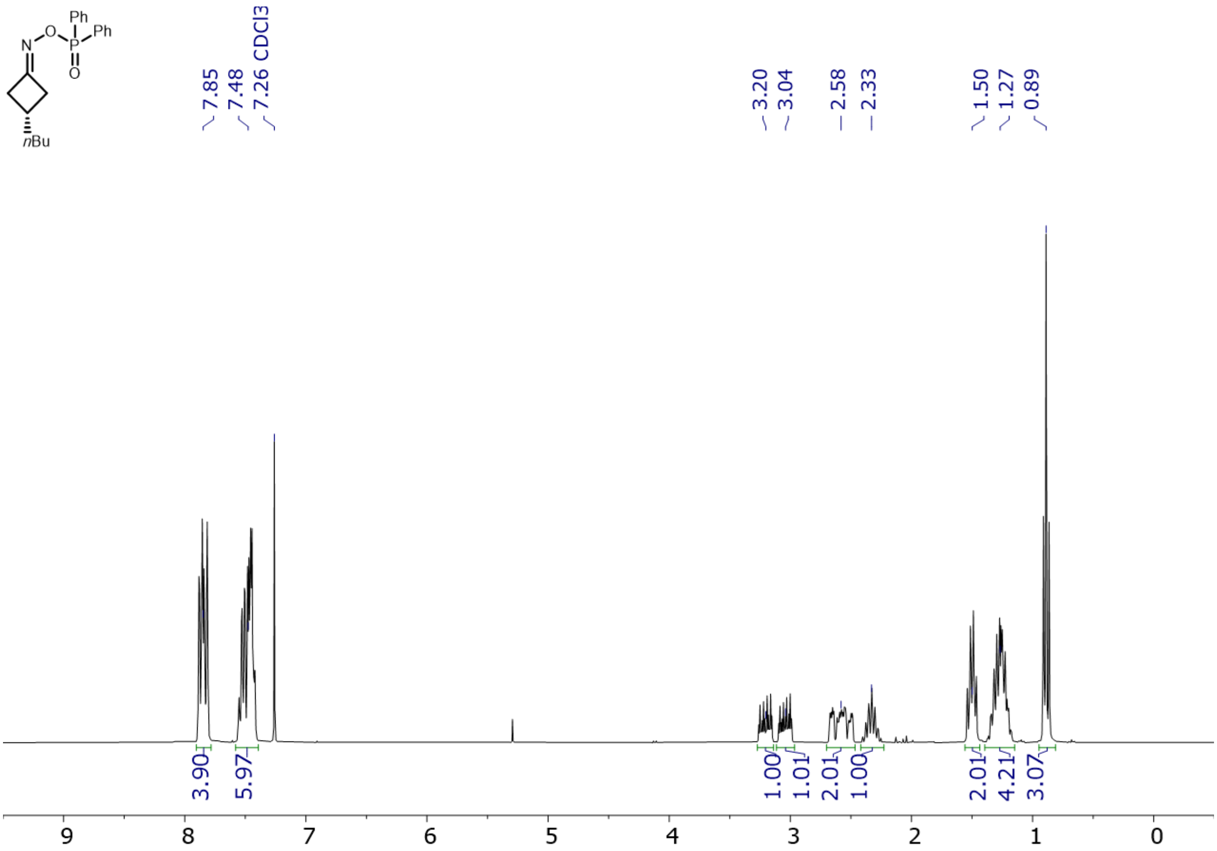

[6m] ^{13}C, $CDCl_3$, 101 MHz

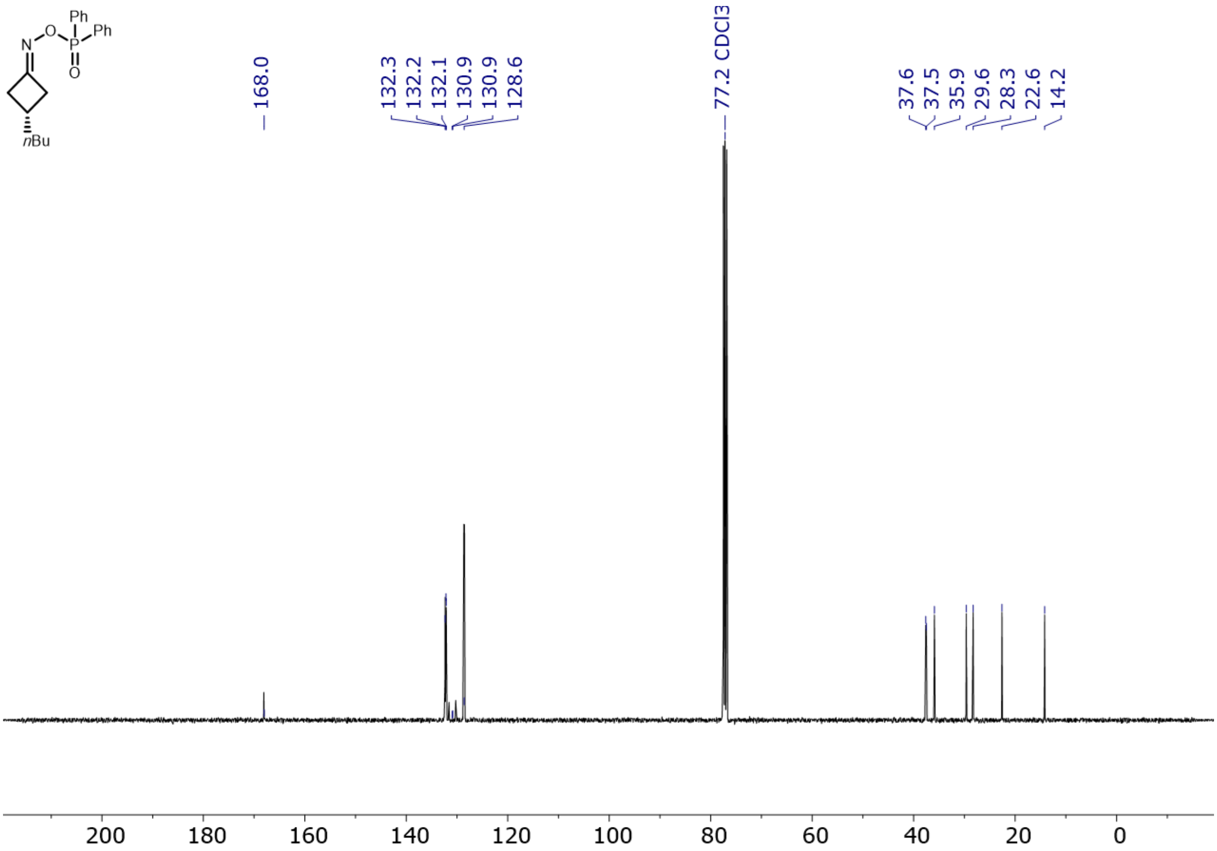

[6m] ^{31}P, $CDCl_3$, 162 MHz

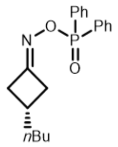

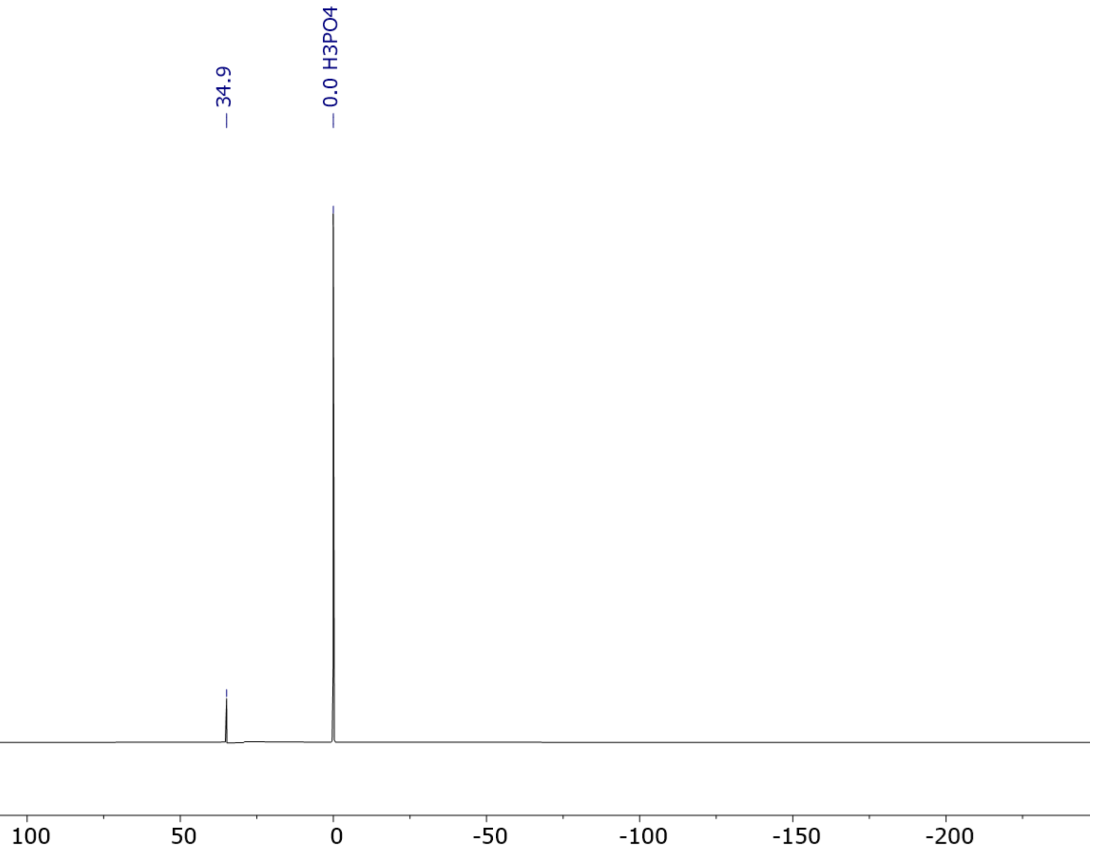

[6n] ^{1}H, $CDCl_3$, 400 MHz

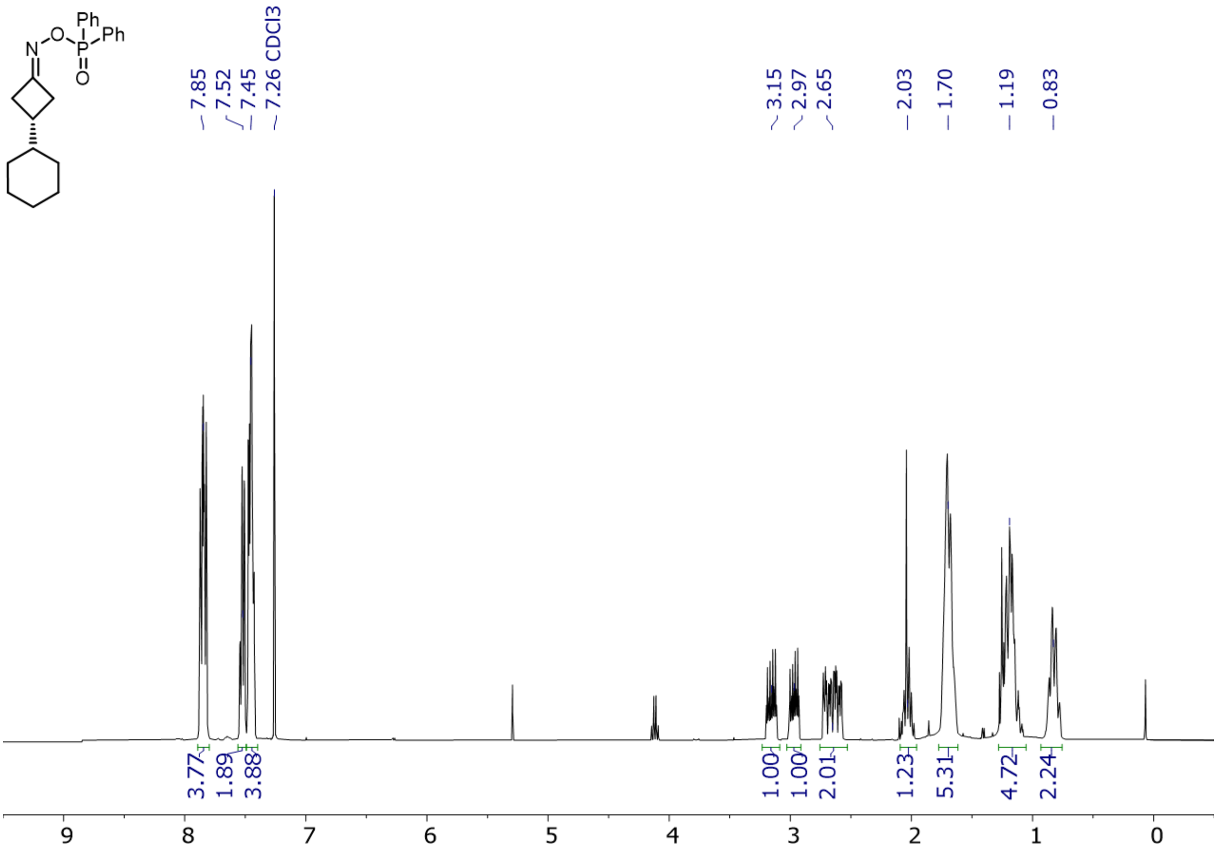

[6n] ^{13}C, $CDCl_3$, 101 MHz

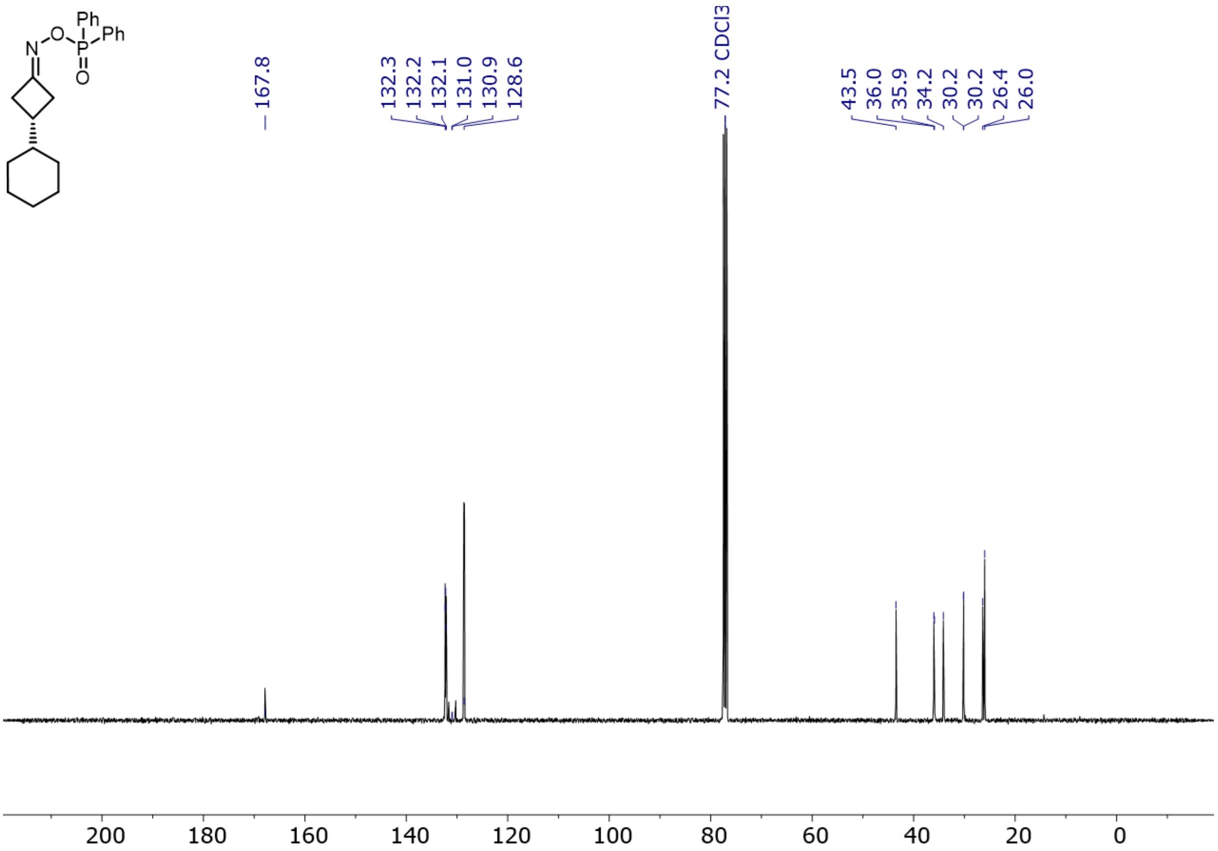

[6n] ^{31}P, $CDCl_3$, 162 MHz

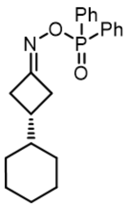

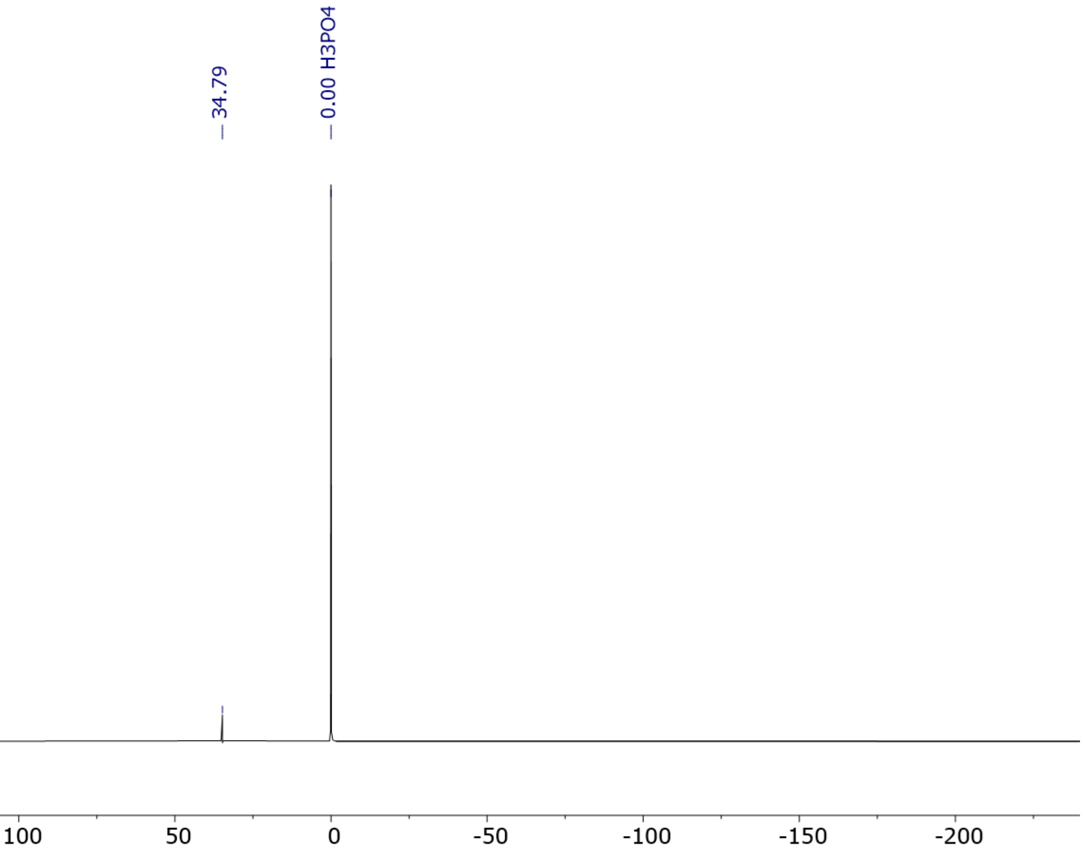

[6o] ^1H, $CDCl_3$, 400 MHz

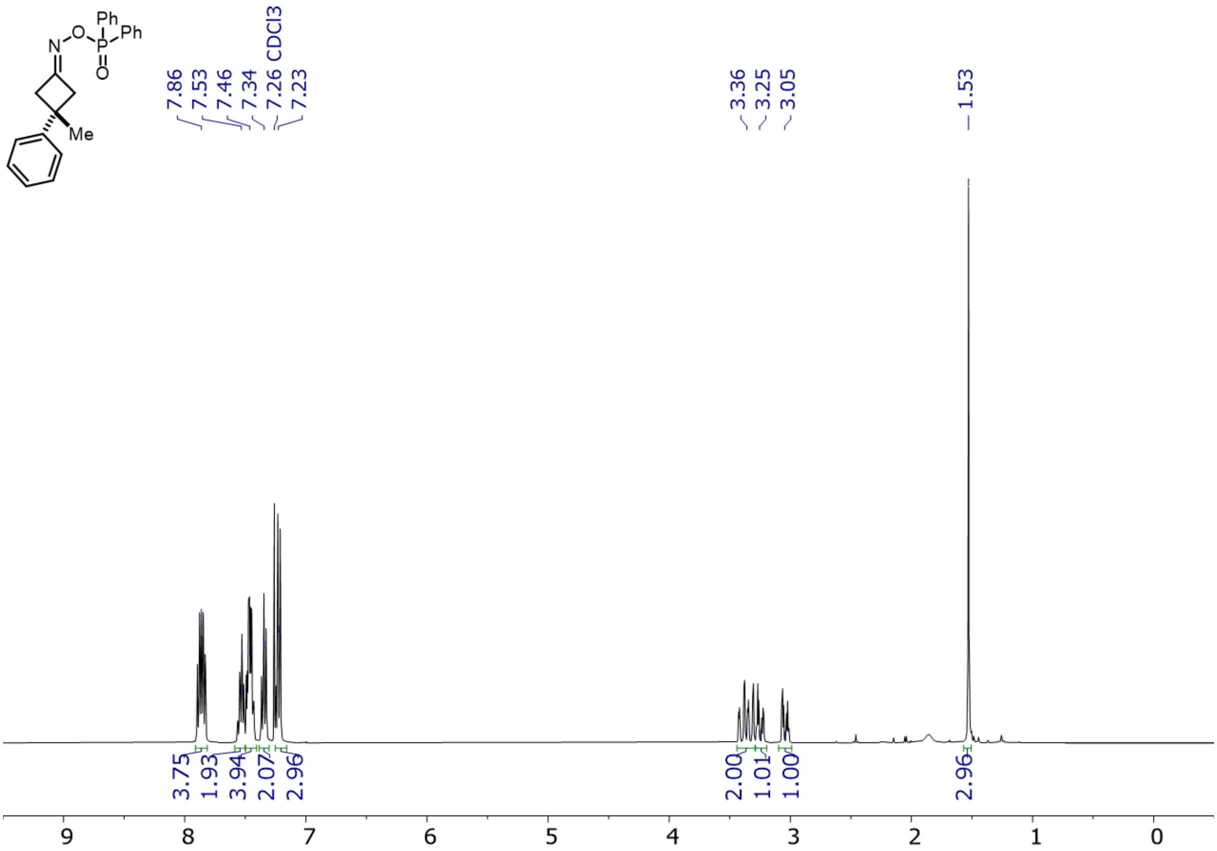

[6o] ^{13}C, $CDCl_3$, 101 MHz

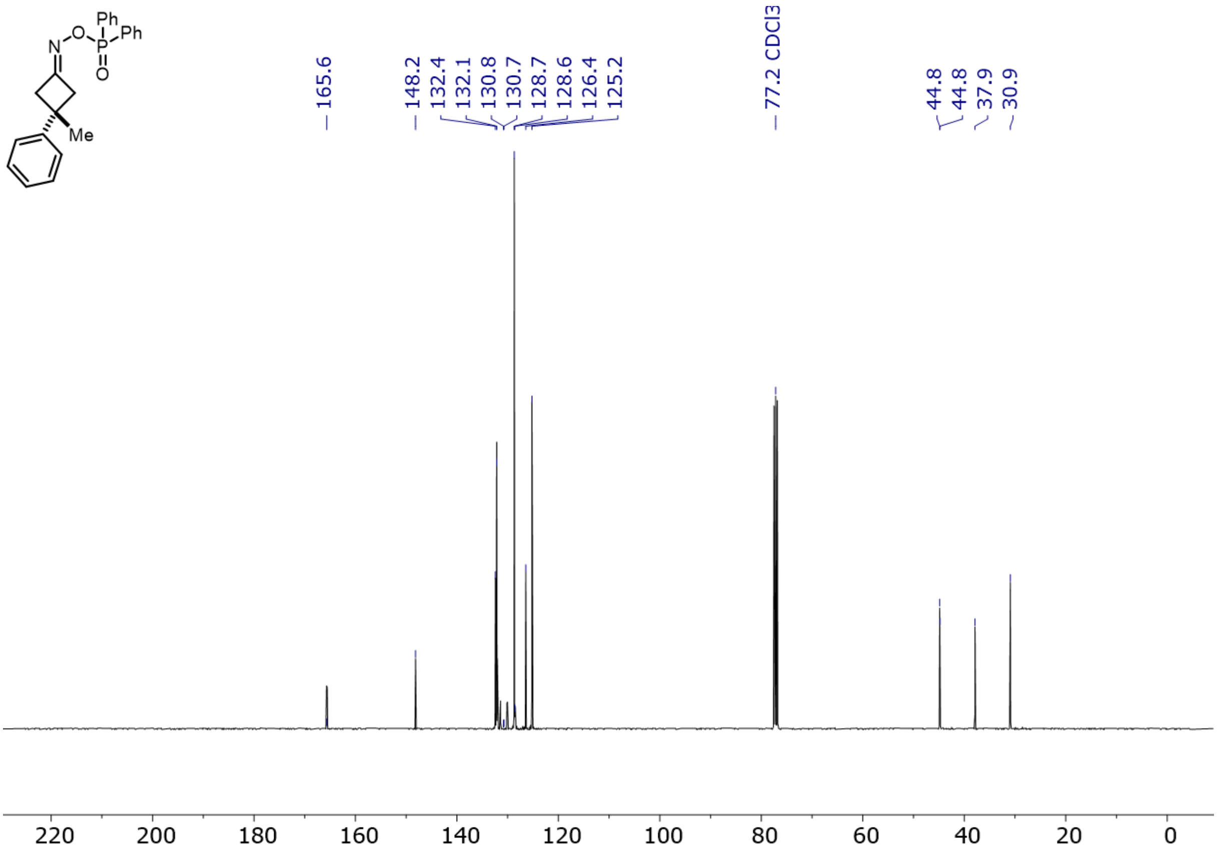

[6o] ^{31}P, $CDCl_3$, 162 MHz

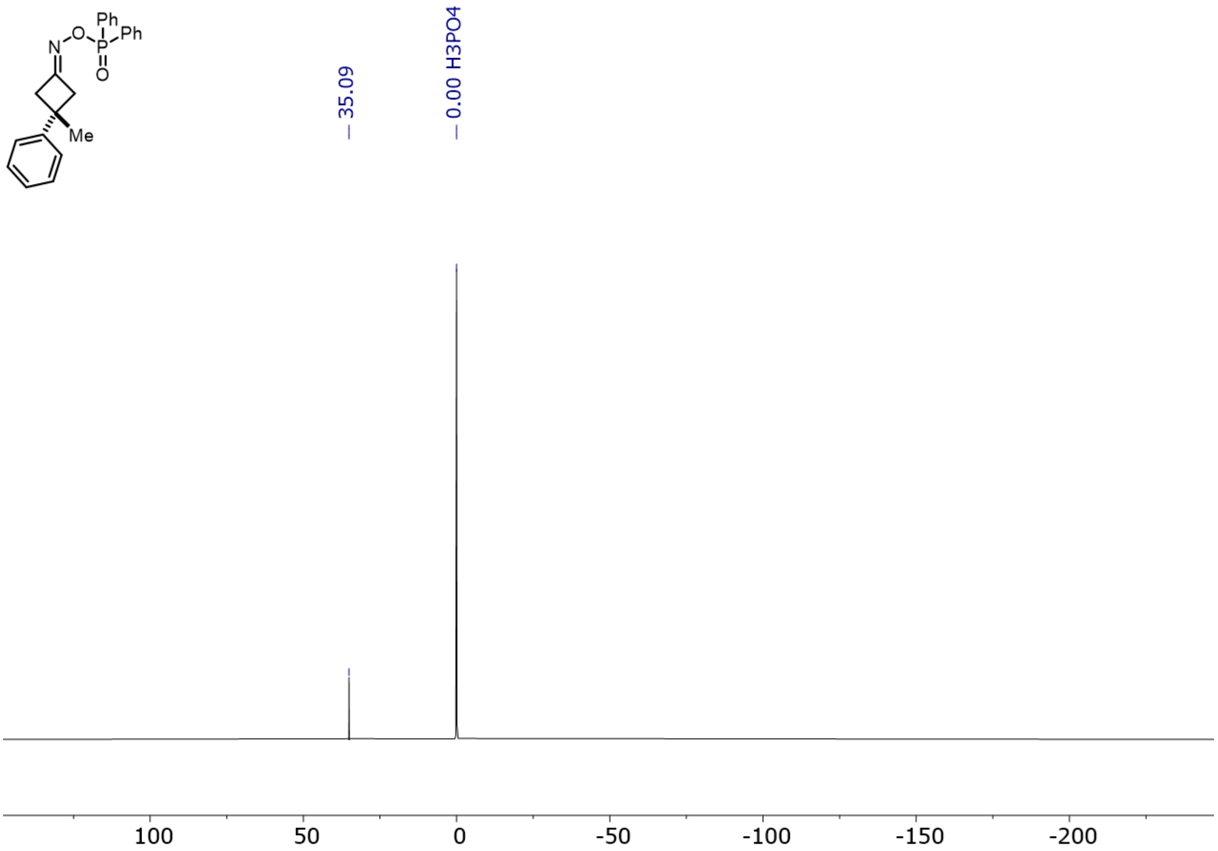

[*cis*-11a] ^{1}H, $CDCl_3$, 400 MHz

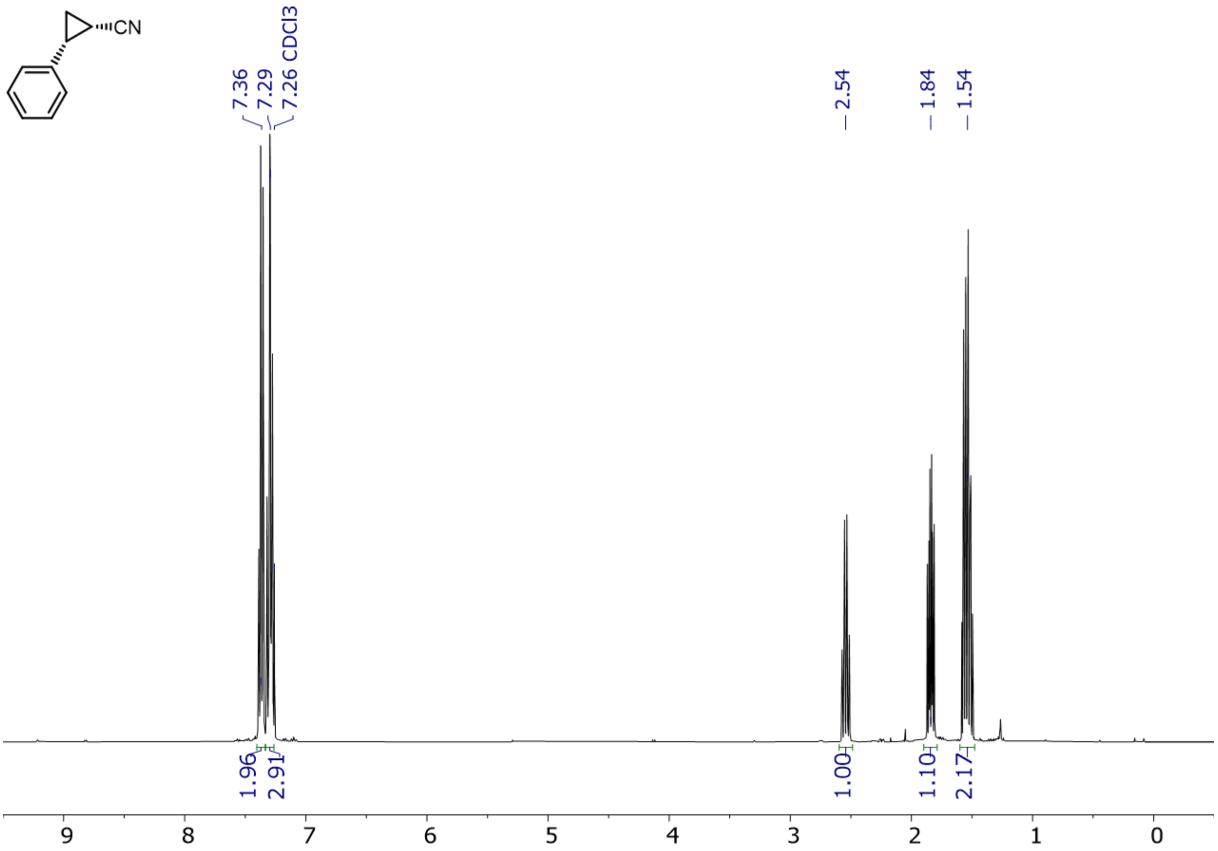

[*cis*-11a] ^{13}C, $CDCl_3$, 101 MHz

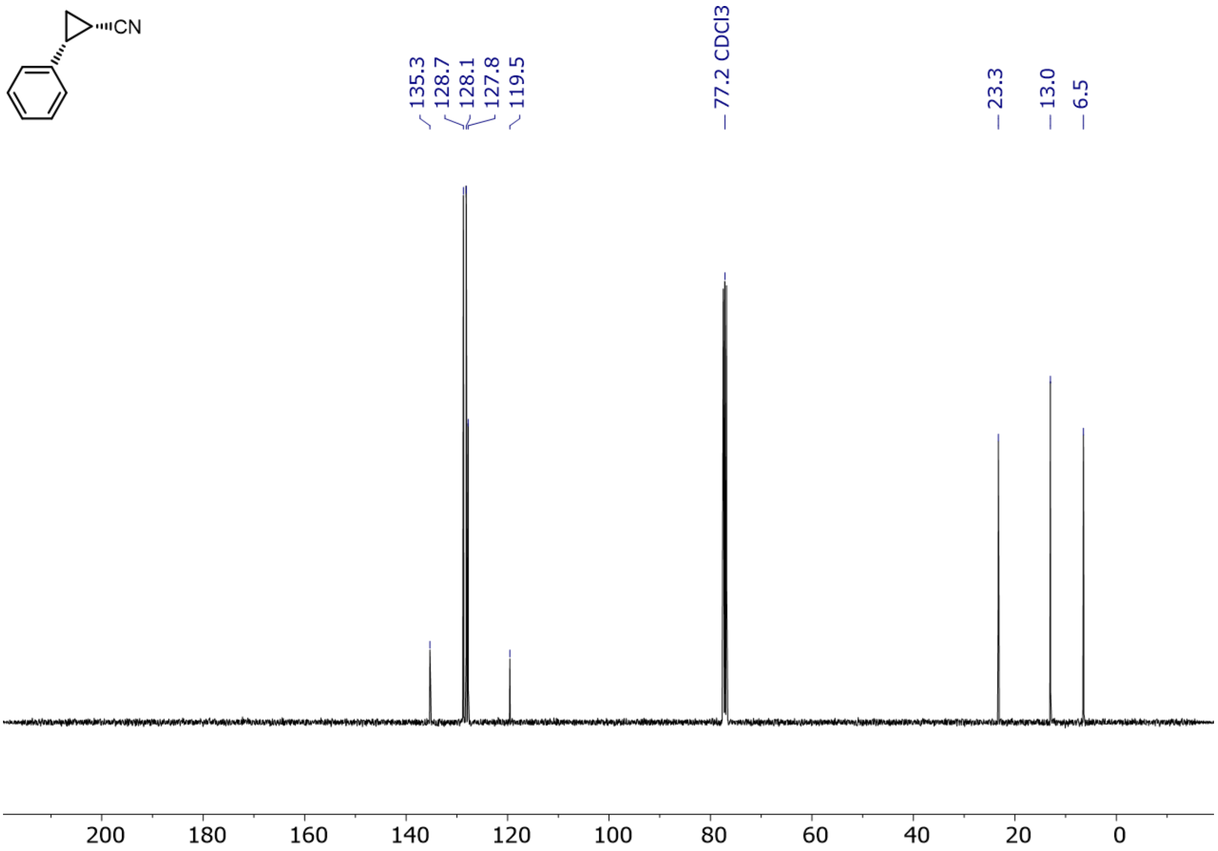

[*trans*-11a] ^{1}H, CDCl$_3$, 400 MHz

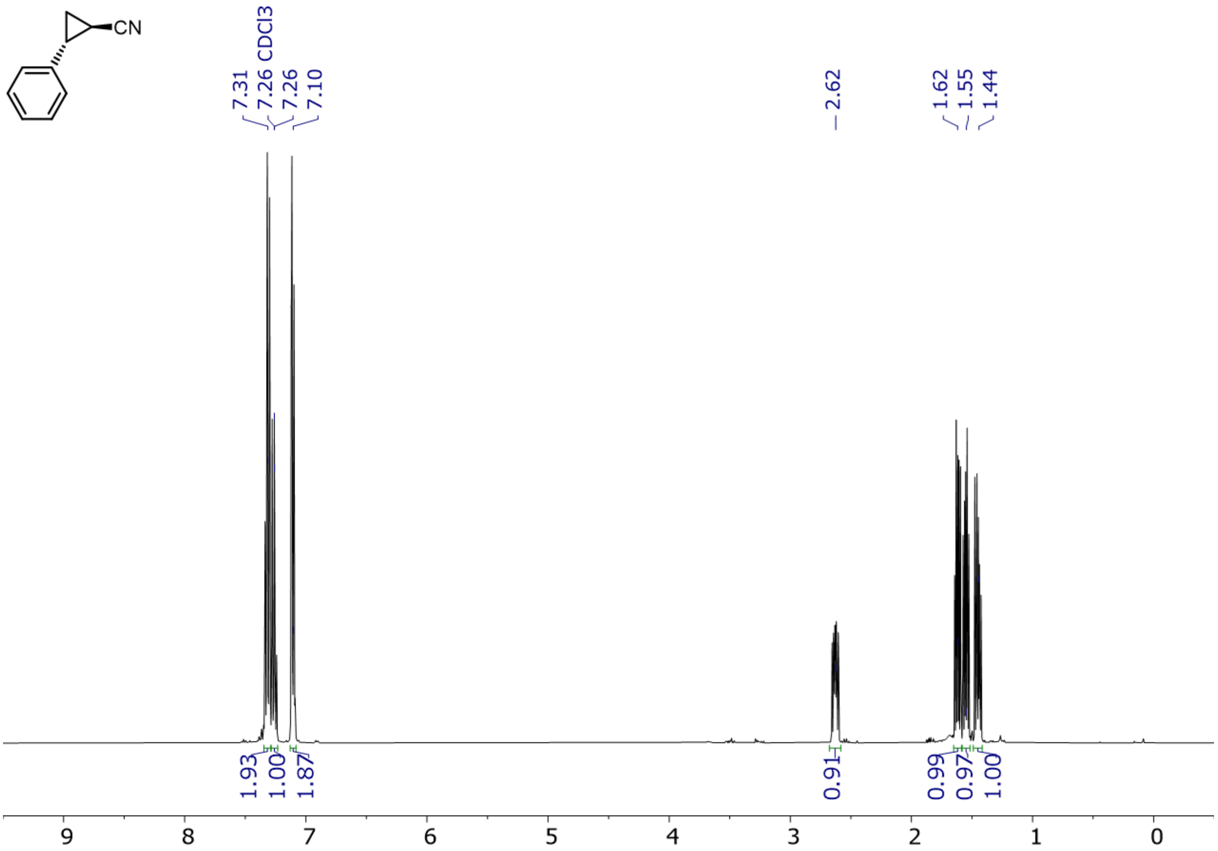

[*trans*-11a] ^{13}C, CDCl$_3$, 101 MHz

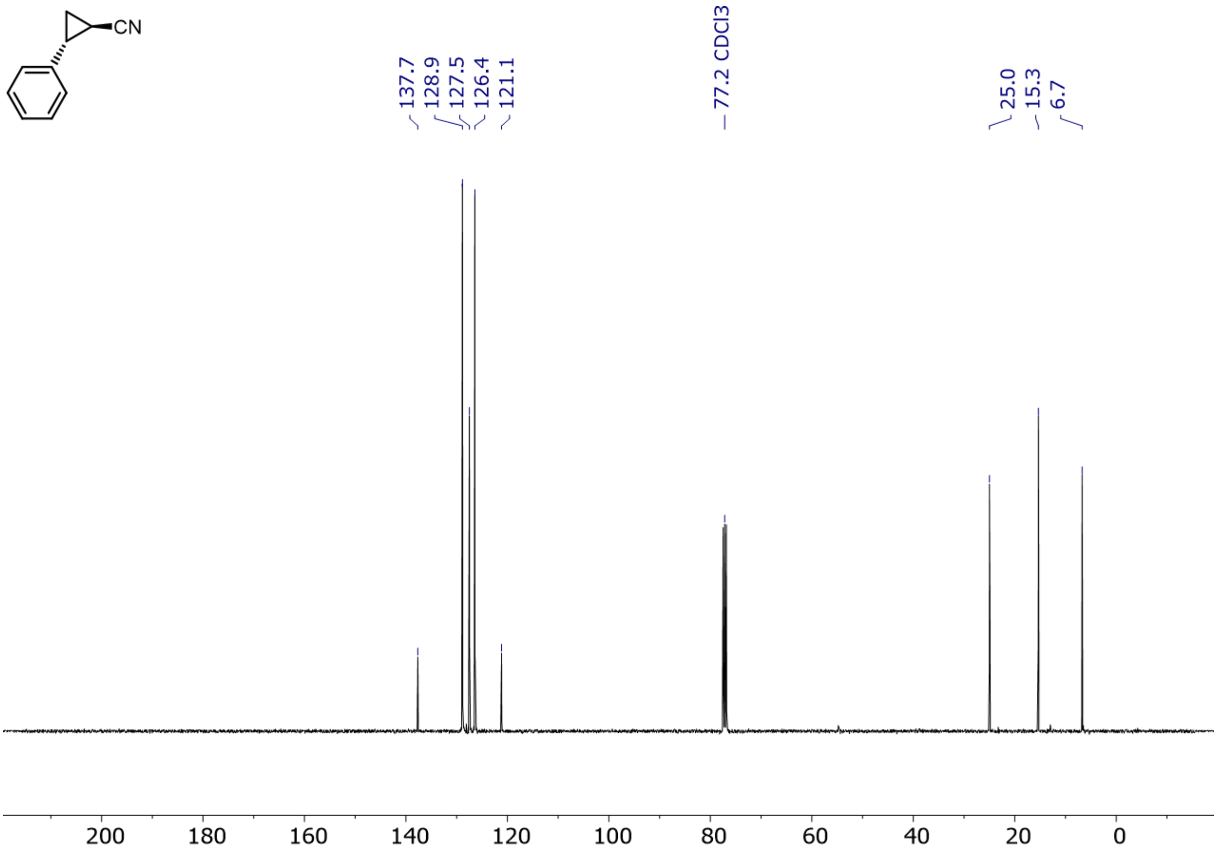

[*cis*-11c] ^{1}H, CDCl$_3$, 400 MHz

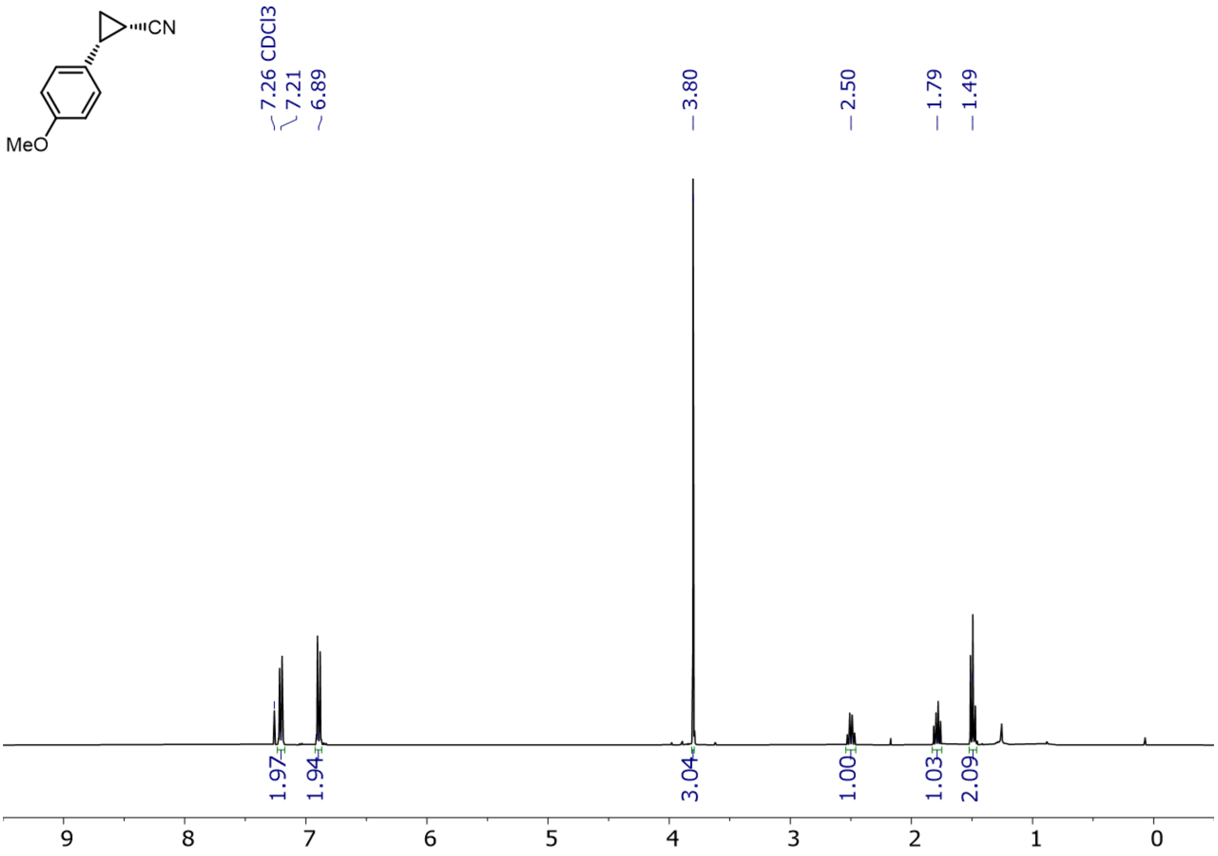

[*cis*-11c] ^{13}C, CDCl$_3$, 101 MHz

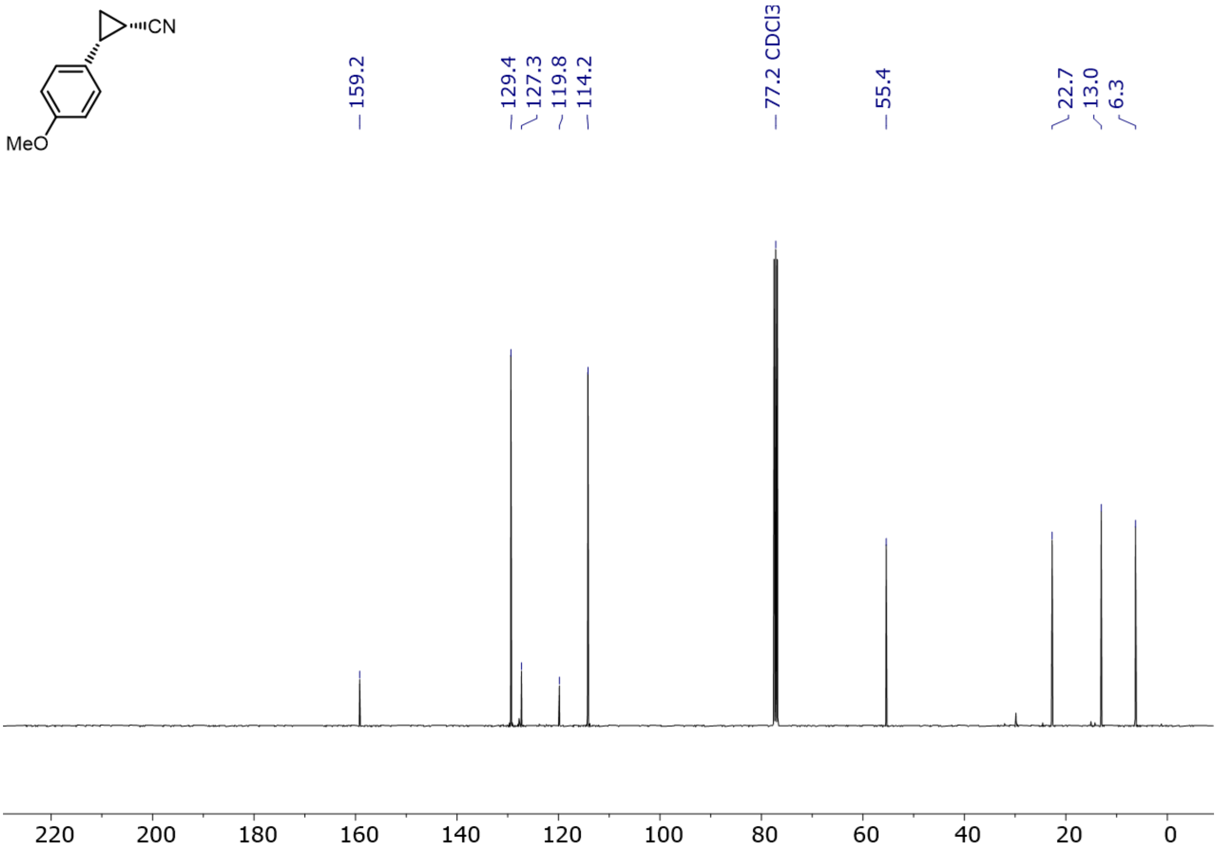

[*cis*-11d] ^{1}H, $CDCl_3$, 400 MHz

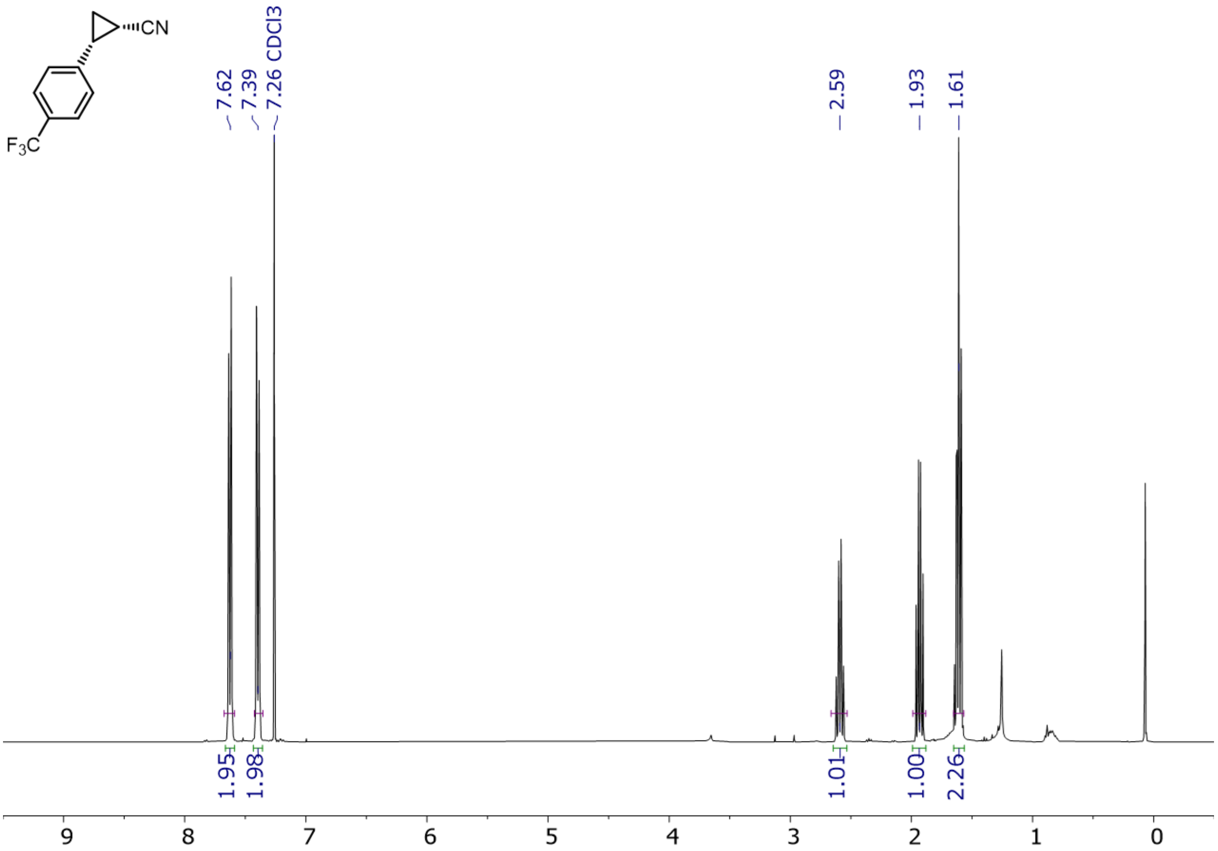

[*cis*-11d] ^{13}C, $CDCl_3$, 101 MHz

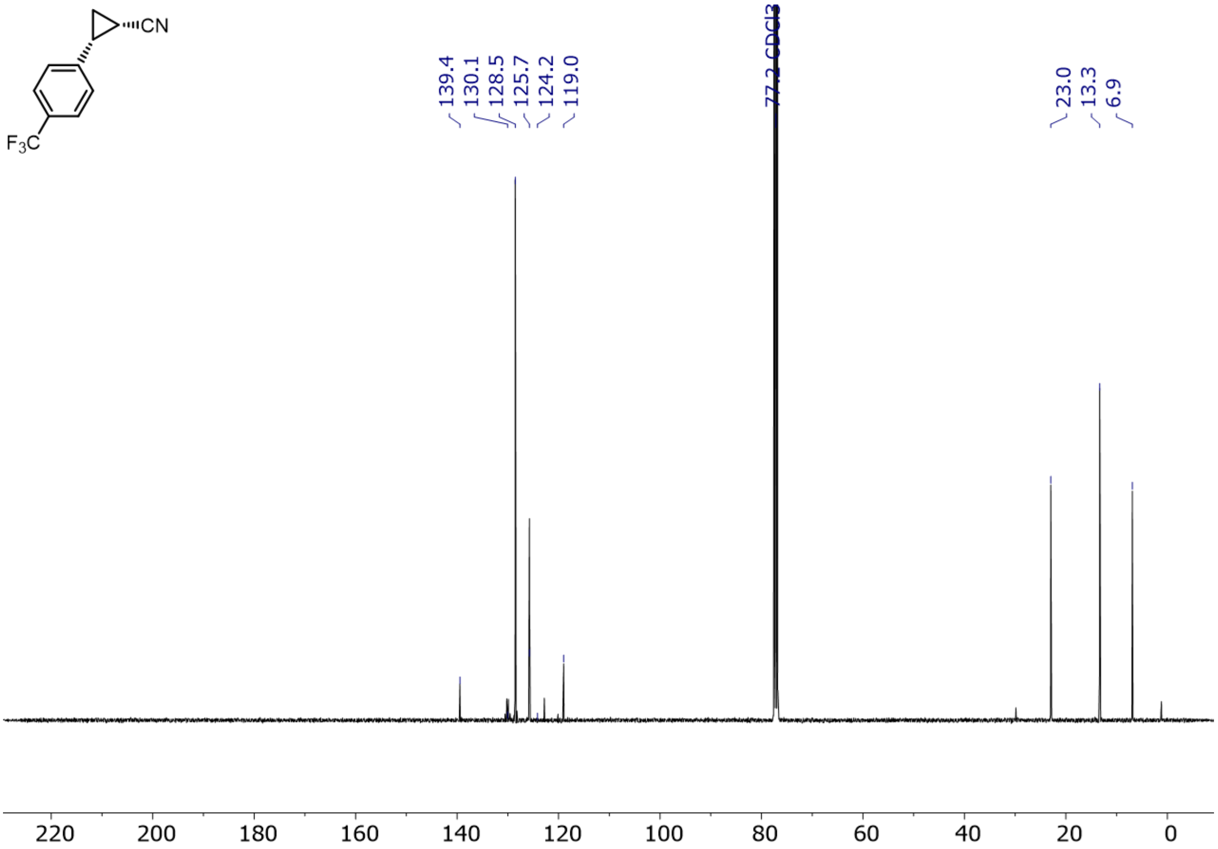

[*cis*-11e] ^{19}F, $CDCl_3$, 376 MHz

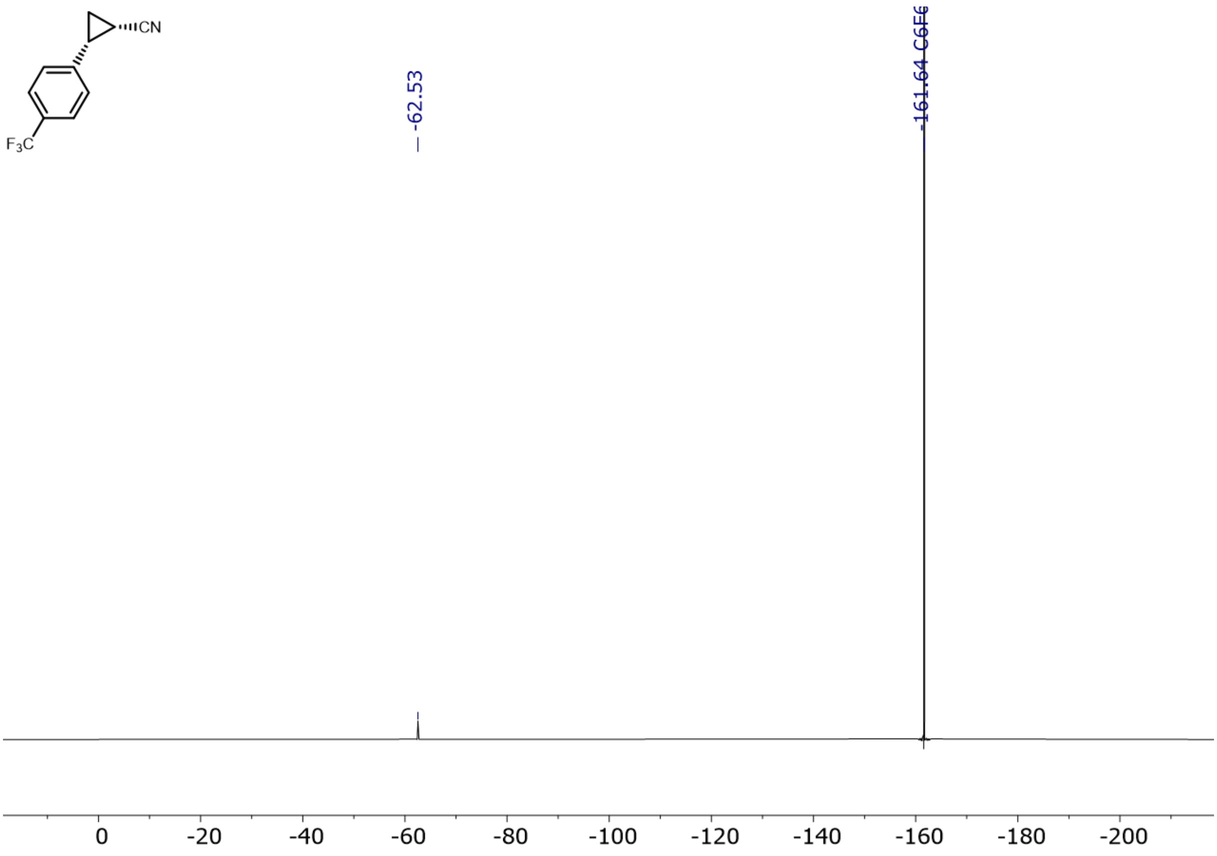

[*cis*-11f] ^{1}H, $CDCl_3$, 400 MHz

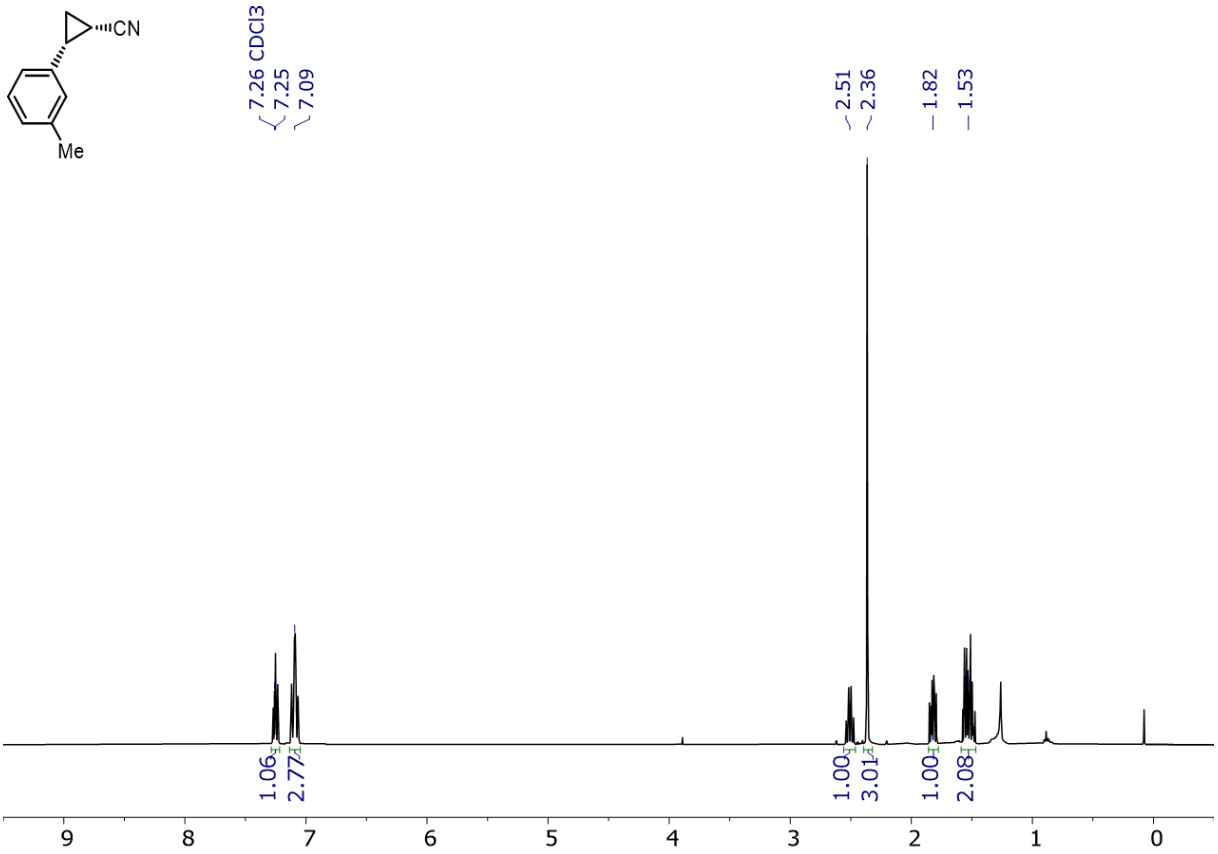

[*cis*-11f] ^{13}C, $CDCl_3$, 101 MHz

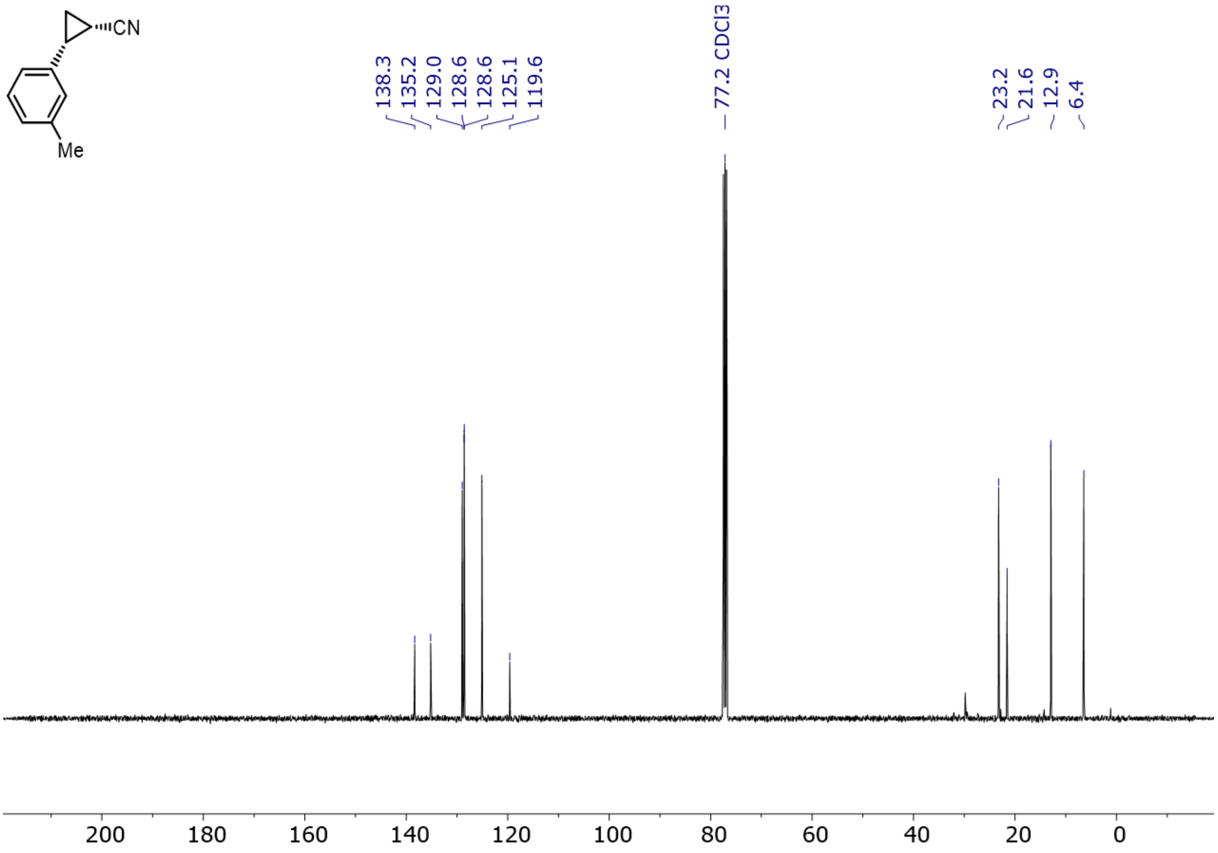

[*trans*-11f] ^{1}H, $CDCl_3$, 400 MHz

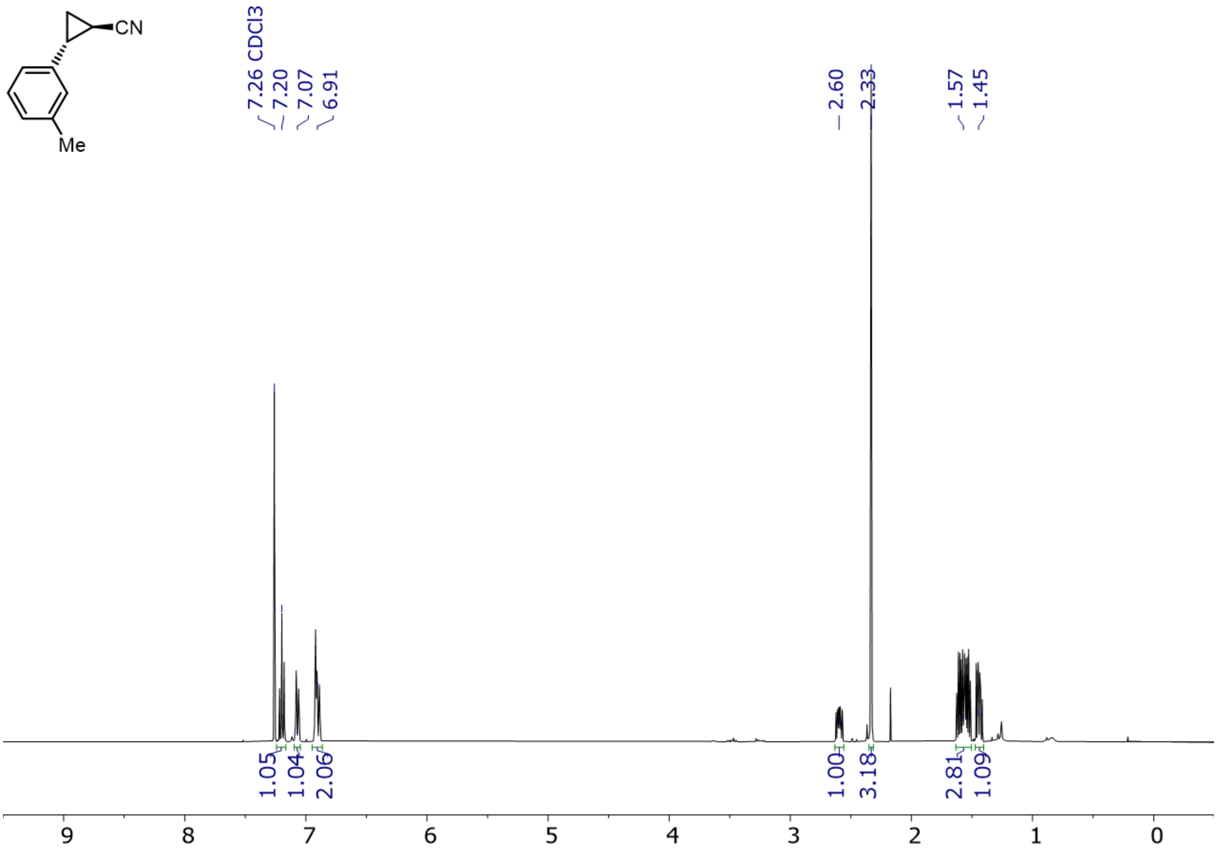

[*trans*-11f] ^{13}C, $CDCl_3$, 101 MHz

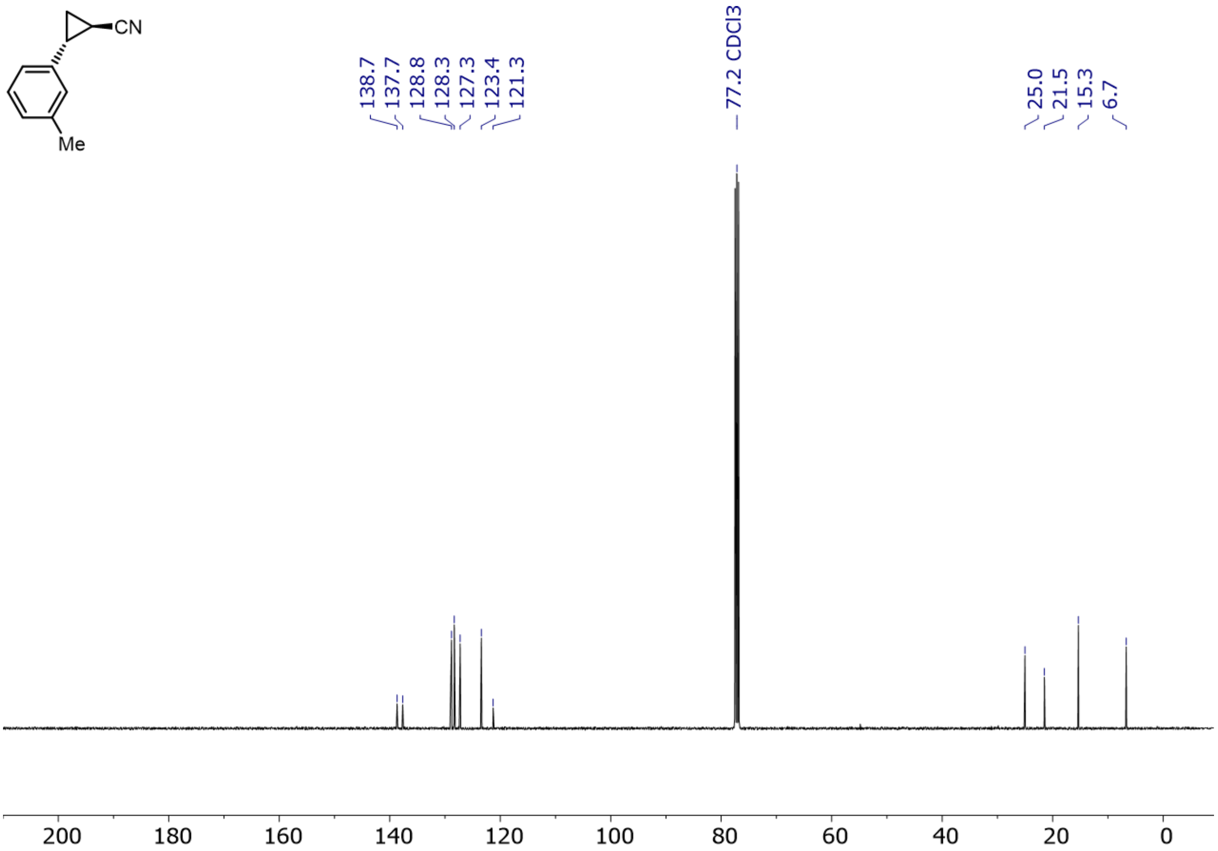

[*cis*-11g] ^{1}H, $CDCl_3$, 400 MHz

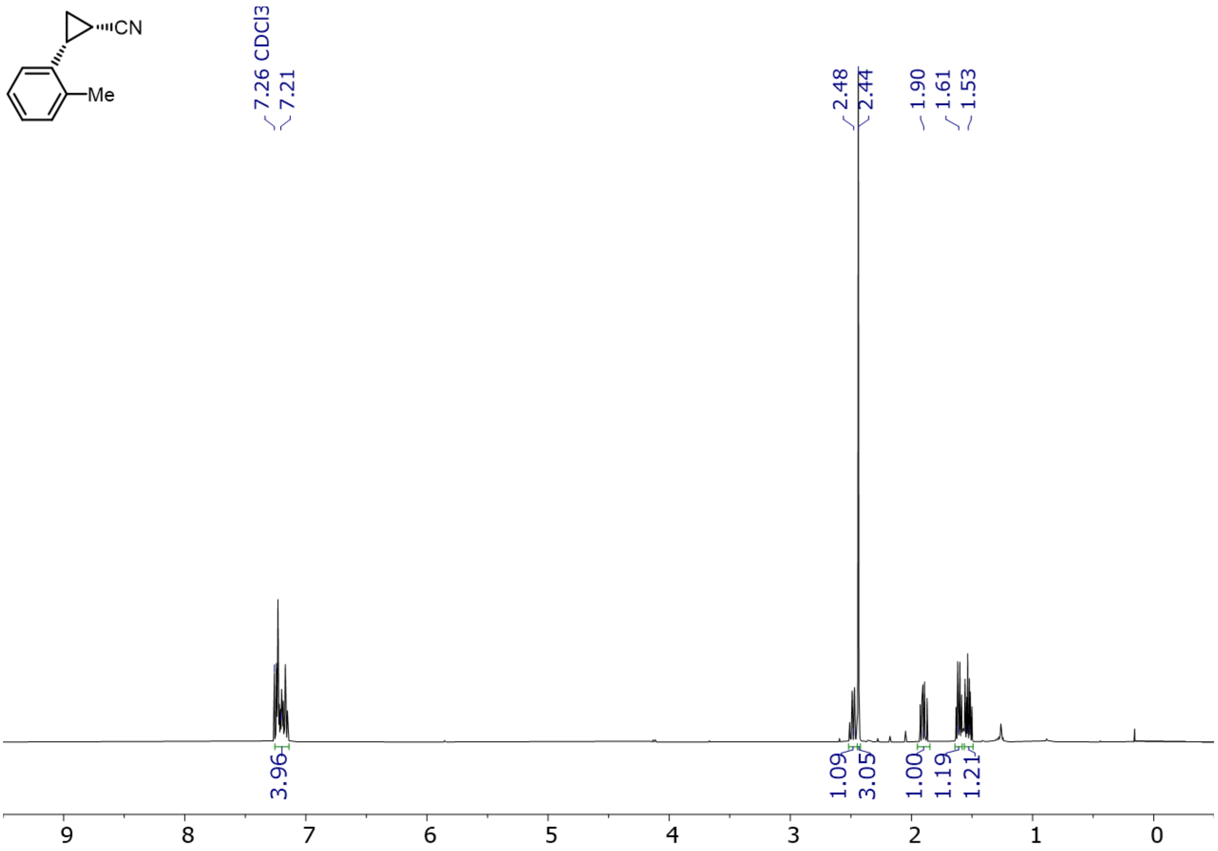

[*cis*-11g] ^{13}C, $CDCl_3$, 101 MHz

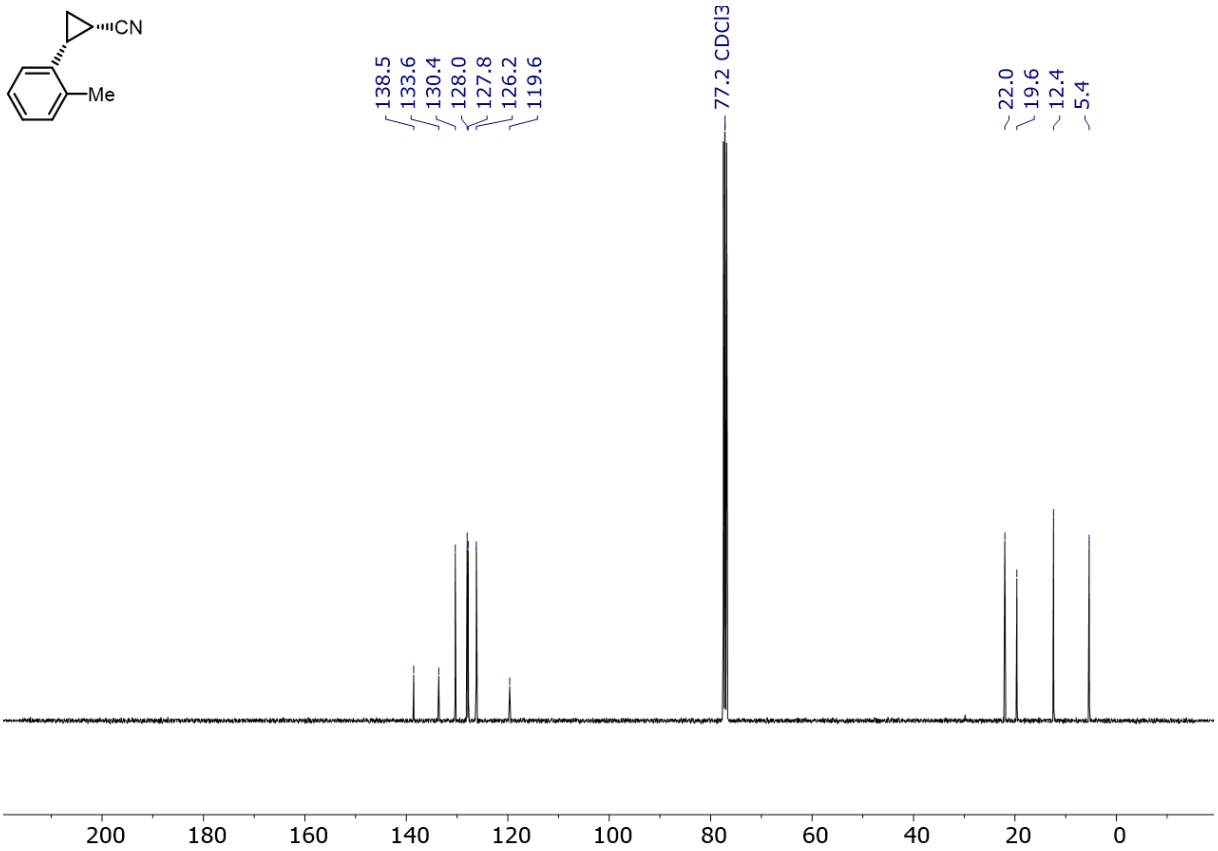

[*trans*-11g] ^{1}H, $CDCl_3$, 400 MHz

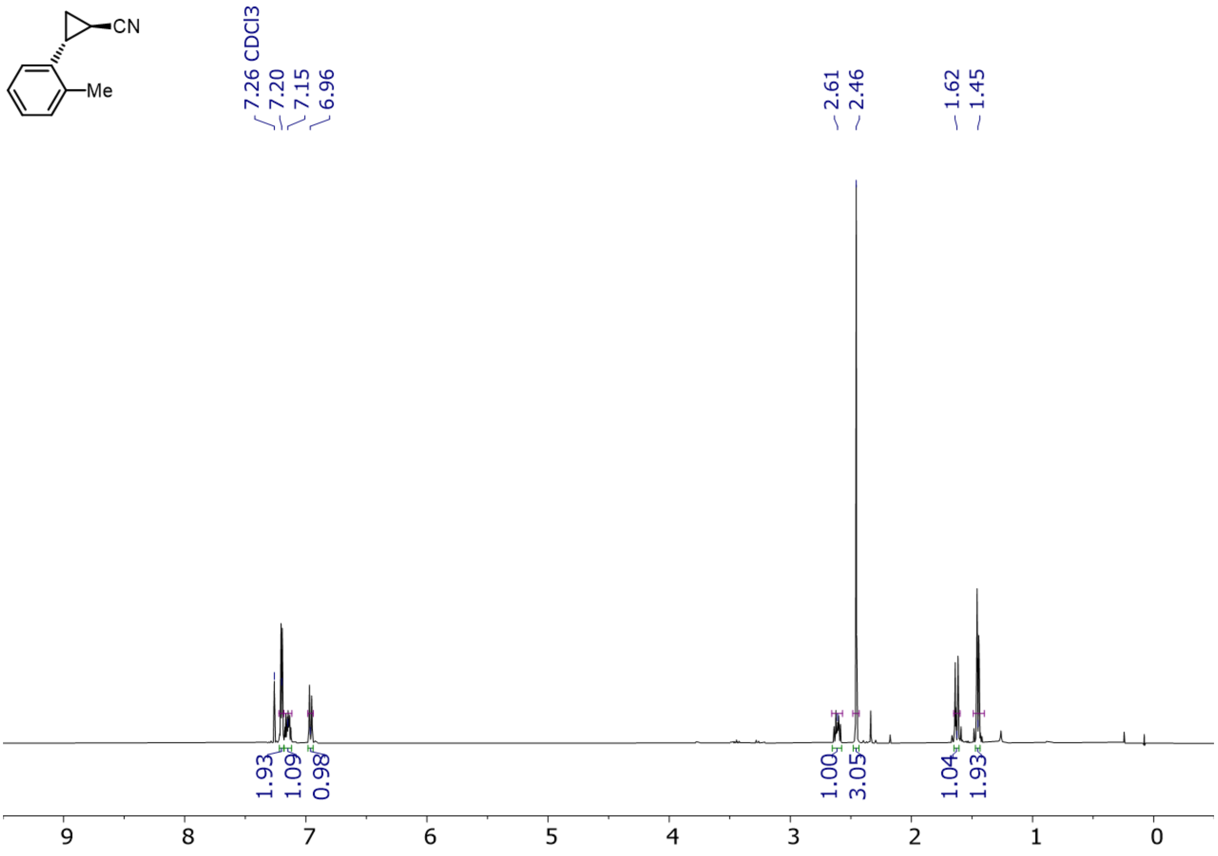

[*trans*-11g] ^{13}C, $CDCl_3$, 101 MHz

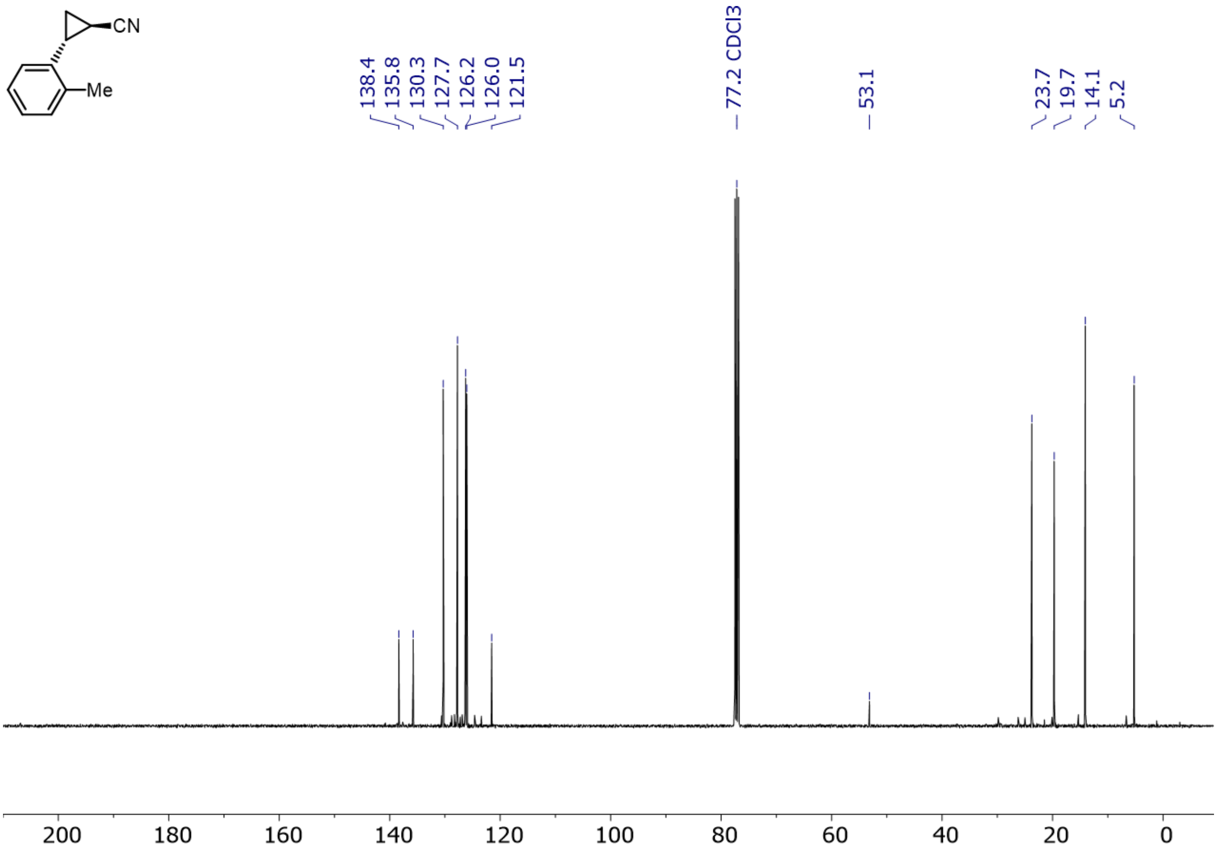

[*cis*-11i] ^{1}H, $CDCl_3$, 400 MHz

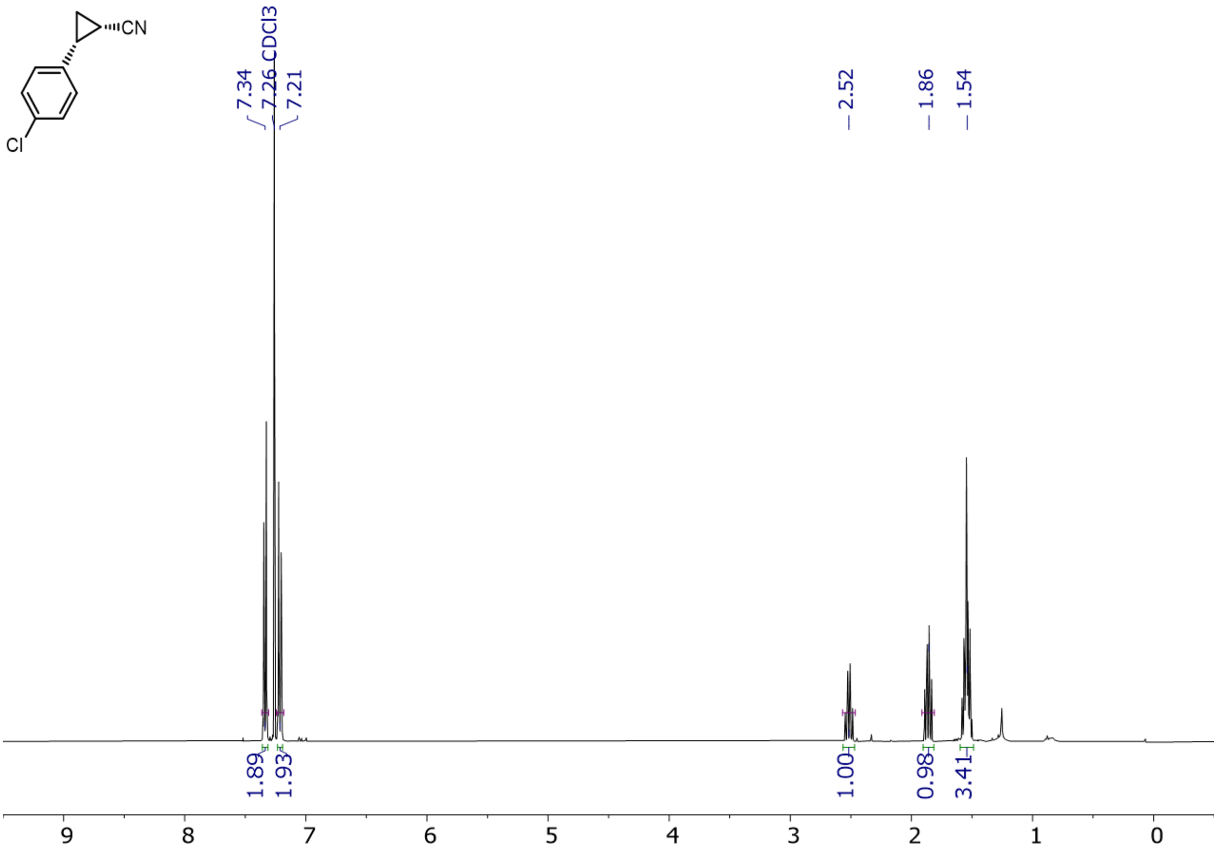

[*cis*-11i] ^{13}C, $CDCl_3$, 101 MHz

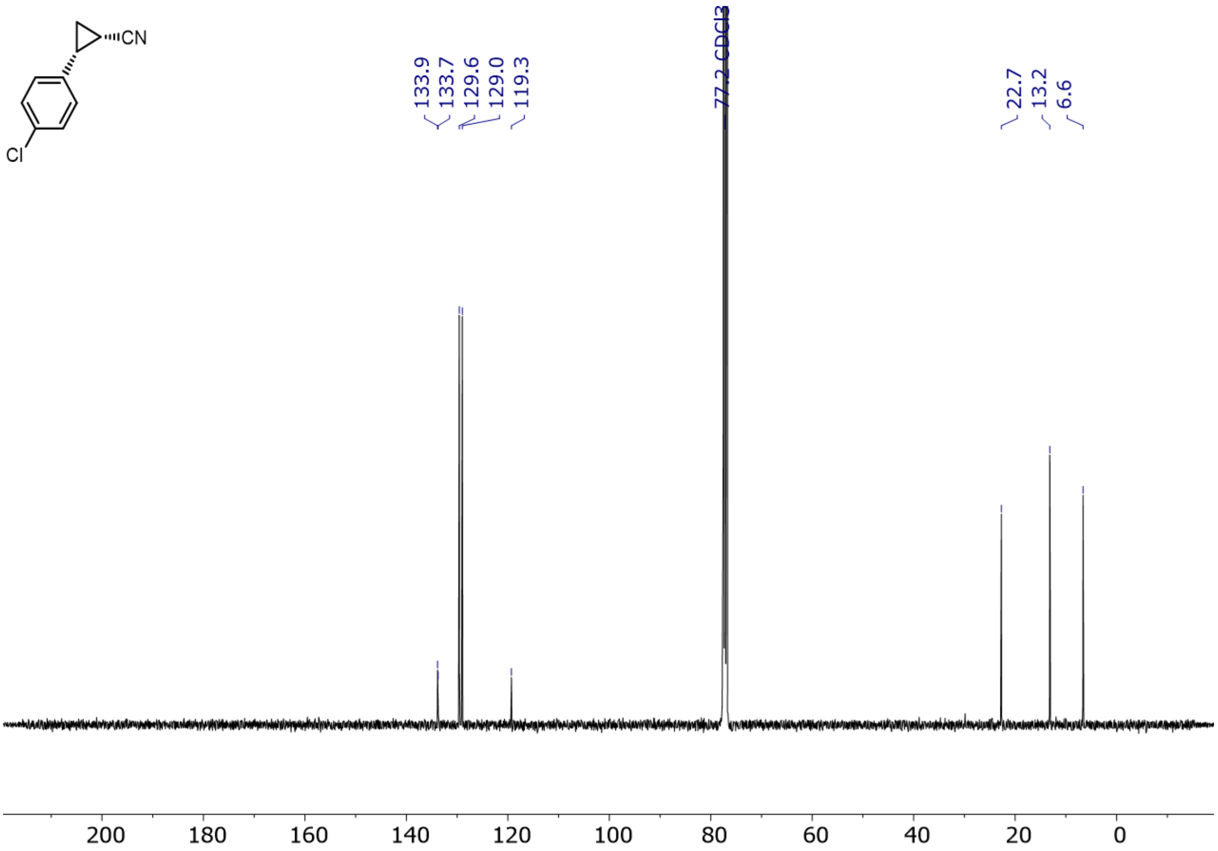

[*cis*-11k] ^1H, $CDCl_3$, 400 MHz

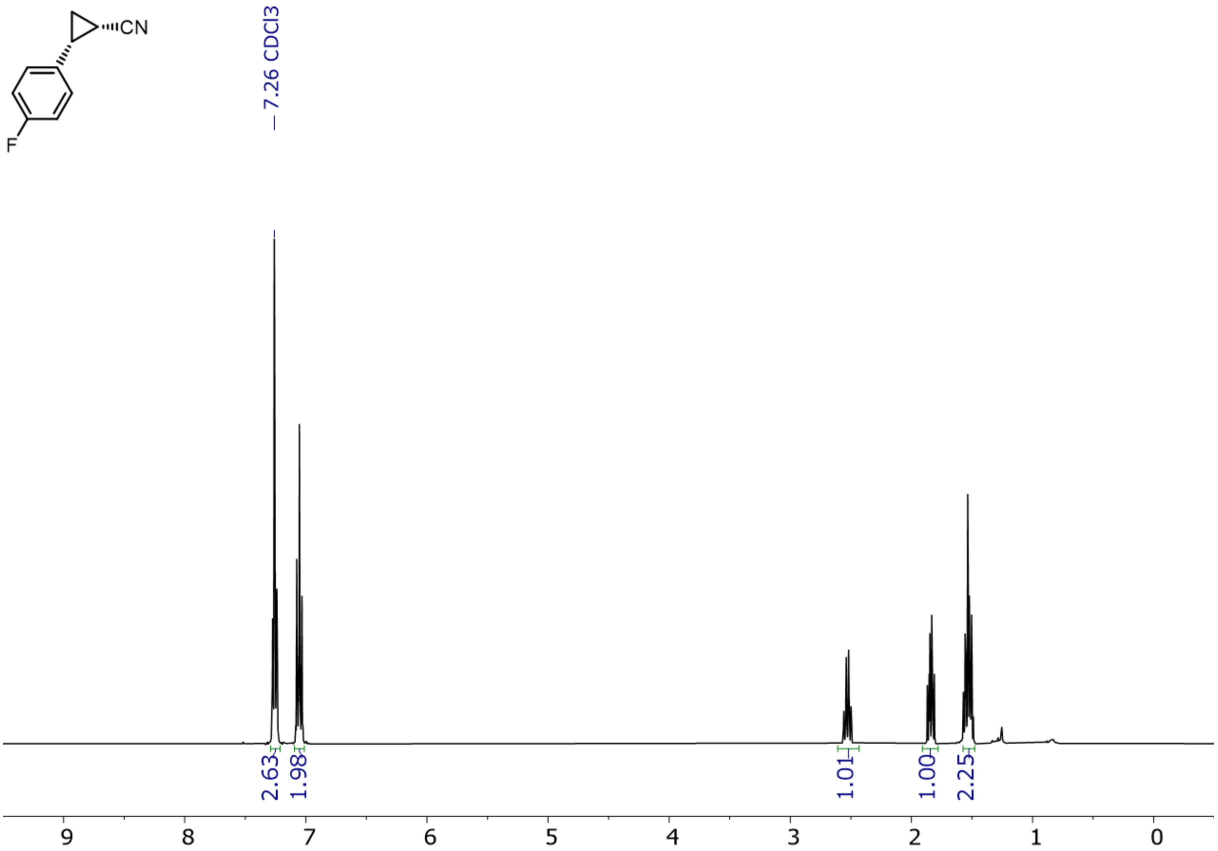

[*cis*-11k] ^{13}C, $CDCl_3$, 101 MHz

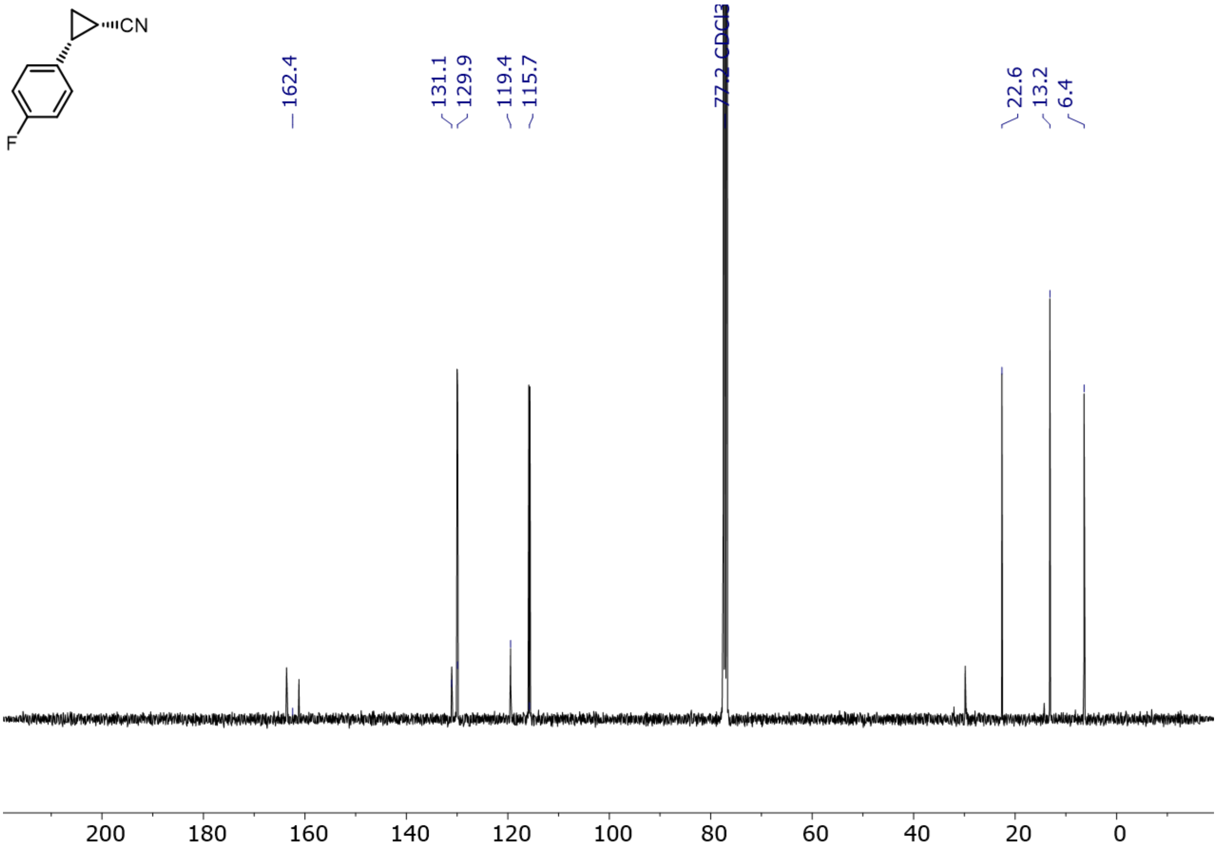

[*cis*-11k] ^{19}F, $CDCl_3$, 375 MHz

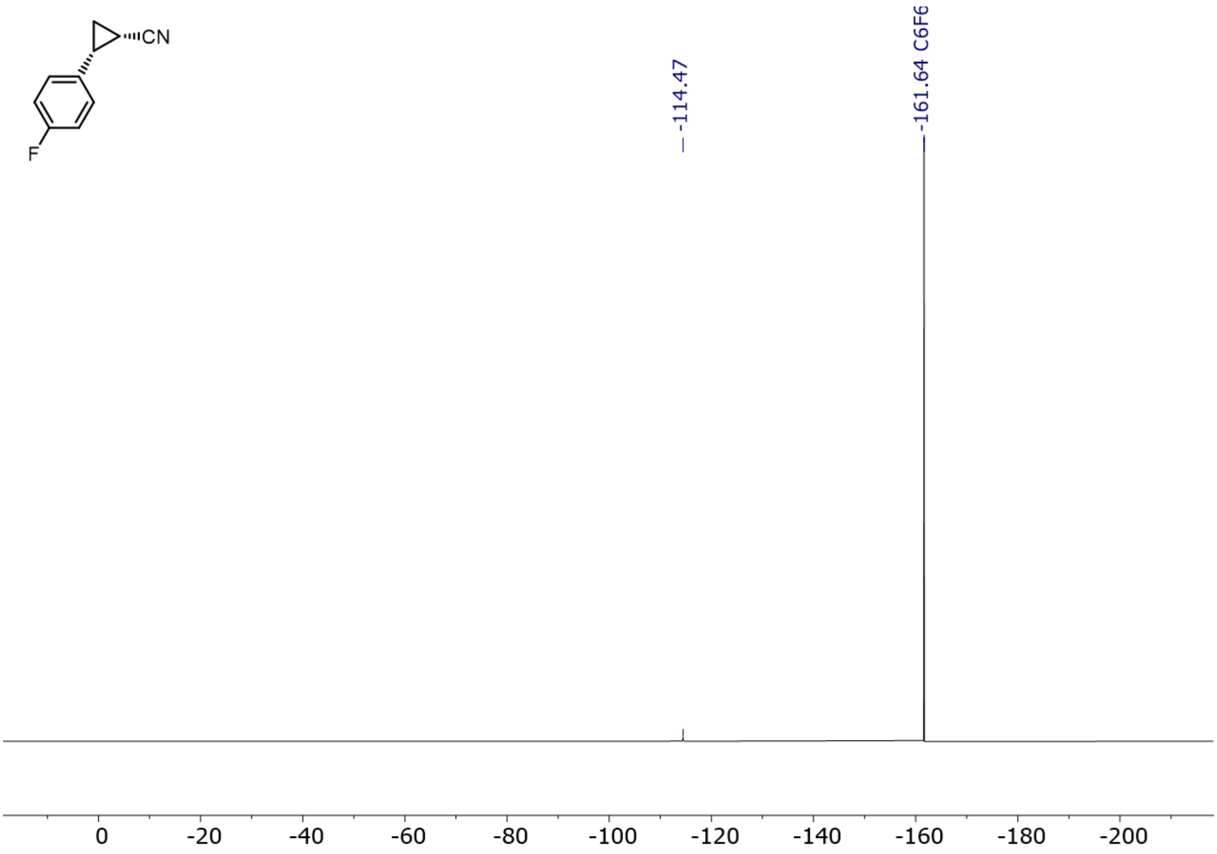

[*cis*-11l] ^{1}H, $CDCl_3$, 400 MHz

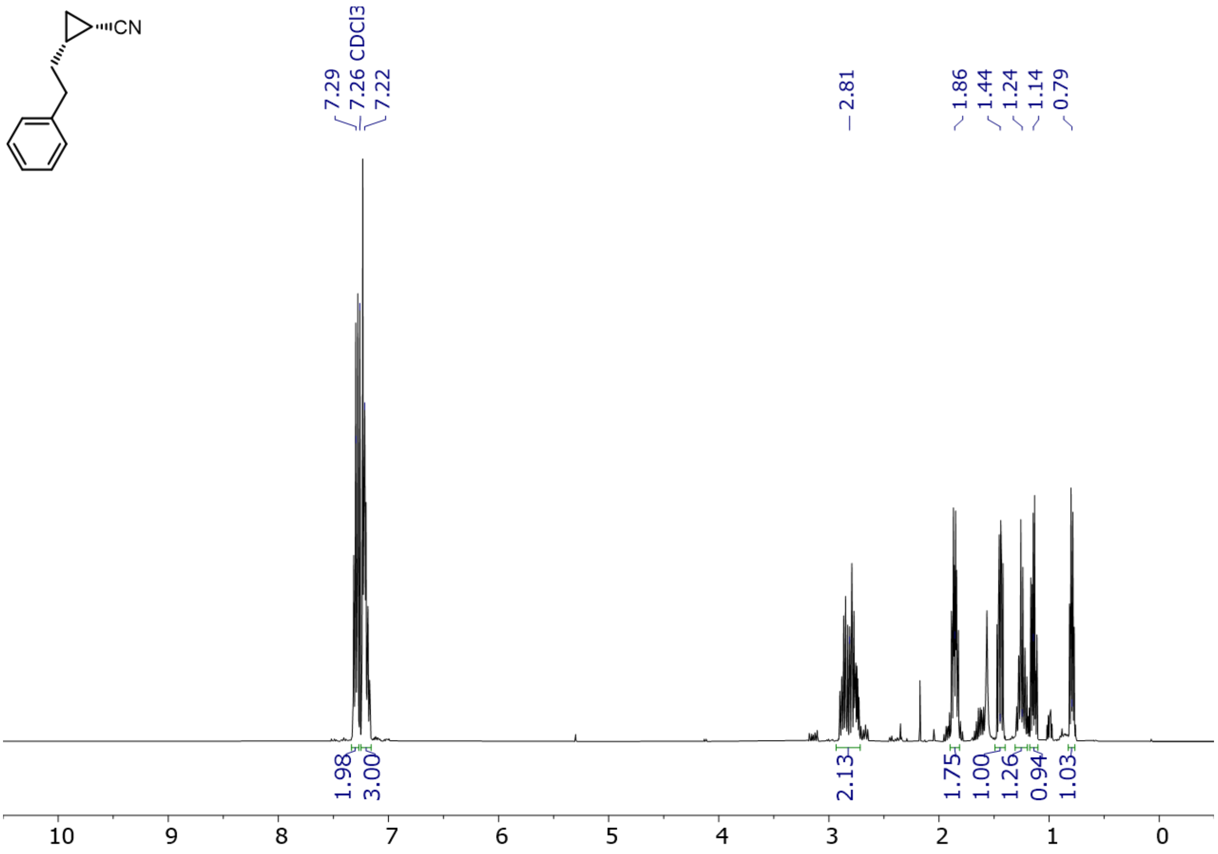

[*cis*-11l] ^{13}C, $CDCl_3$, 101 MHz

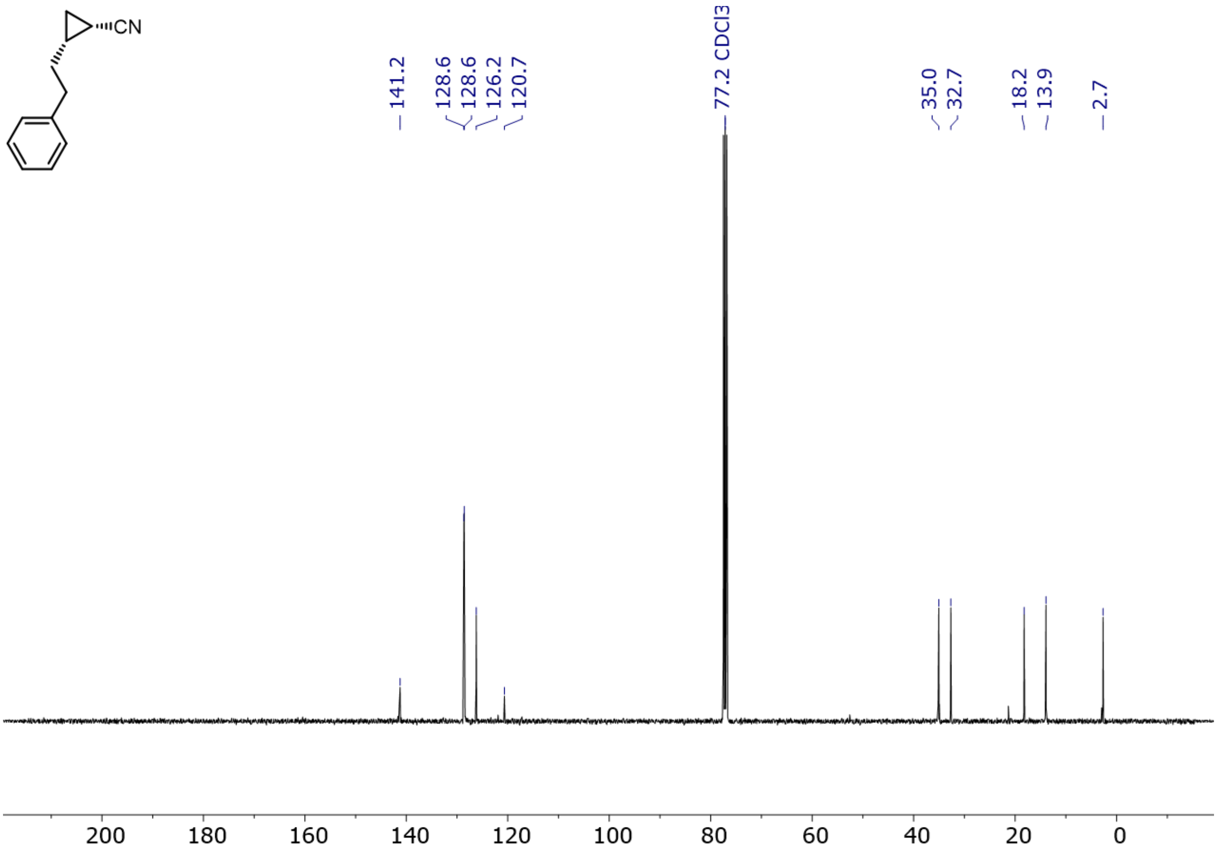

[16] ^{1}H, $CDCl_3$, 400 MHz

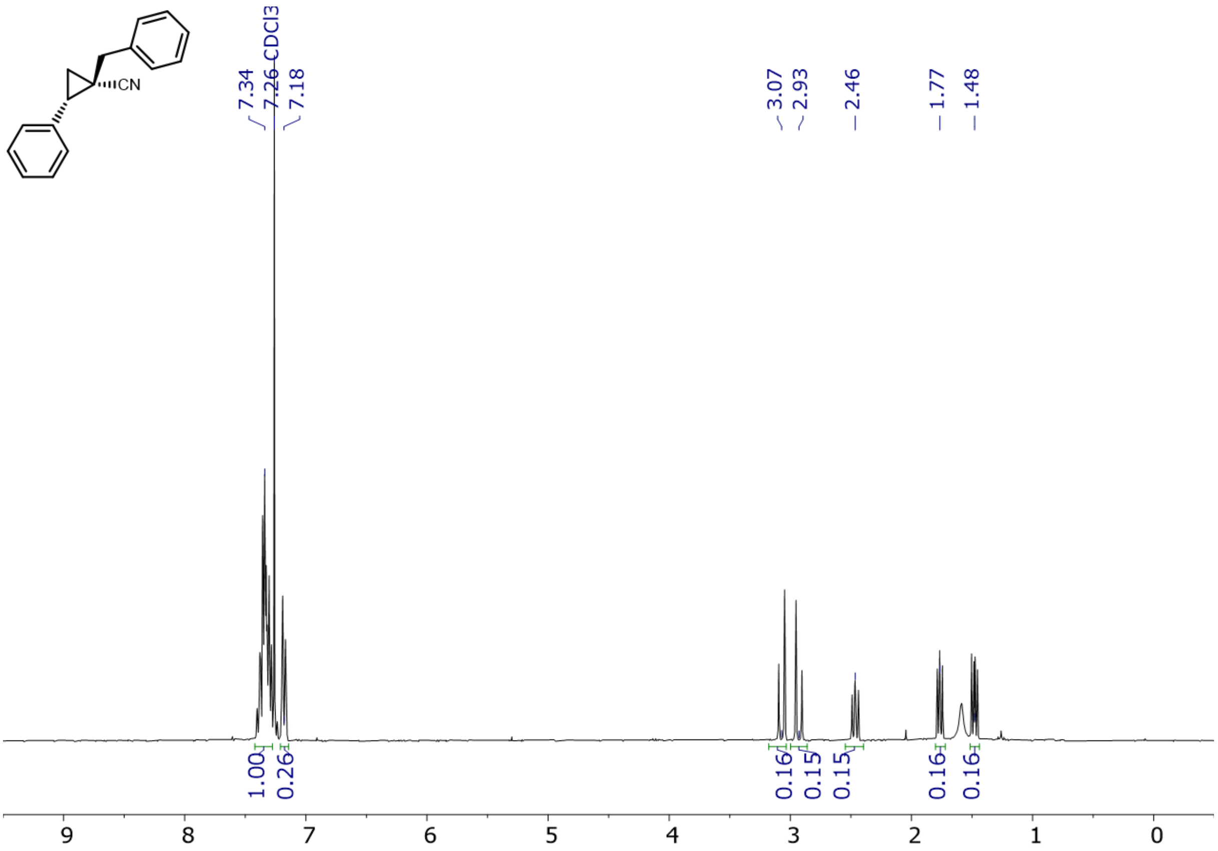

[16] ^{13}C, $CDCl_3$, 101 MHz

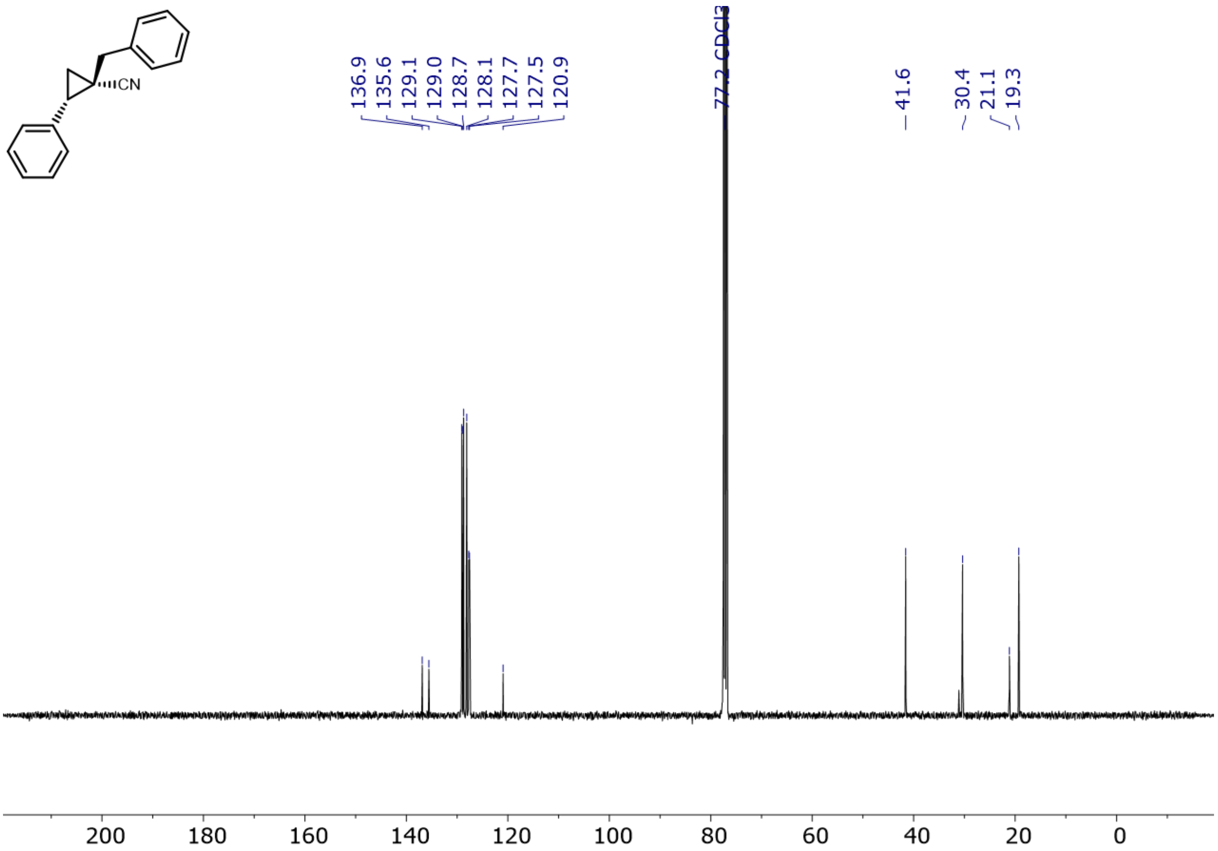

[16] $CDCl_3$, ^{1}H-^{1}H-COSY

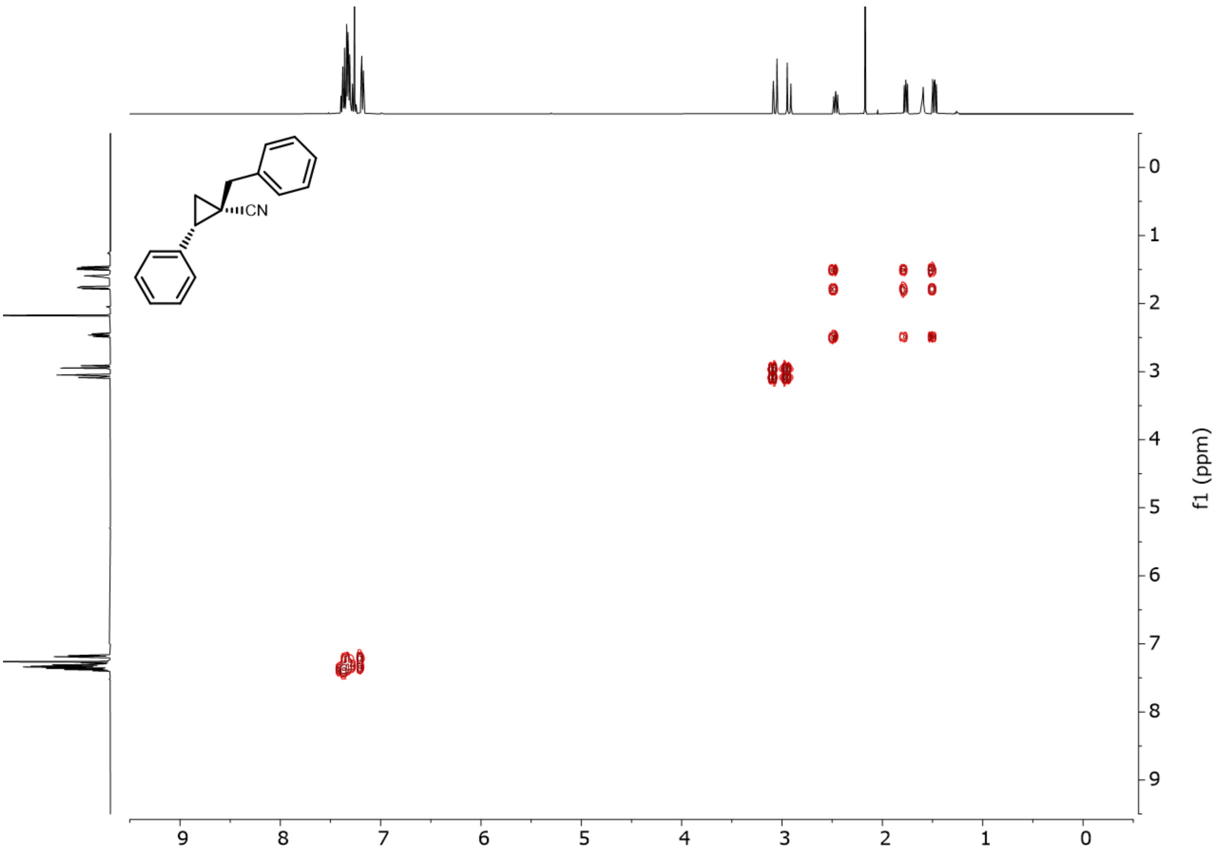

[16] $CDCl_3$, ^{1}H-^{1}H-NOESY

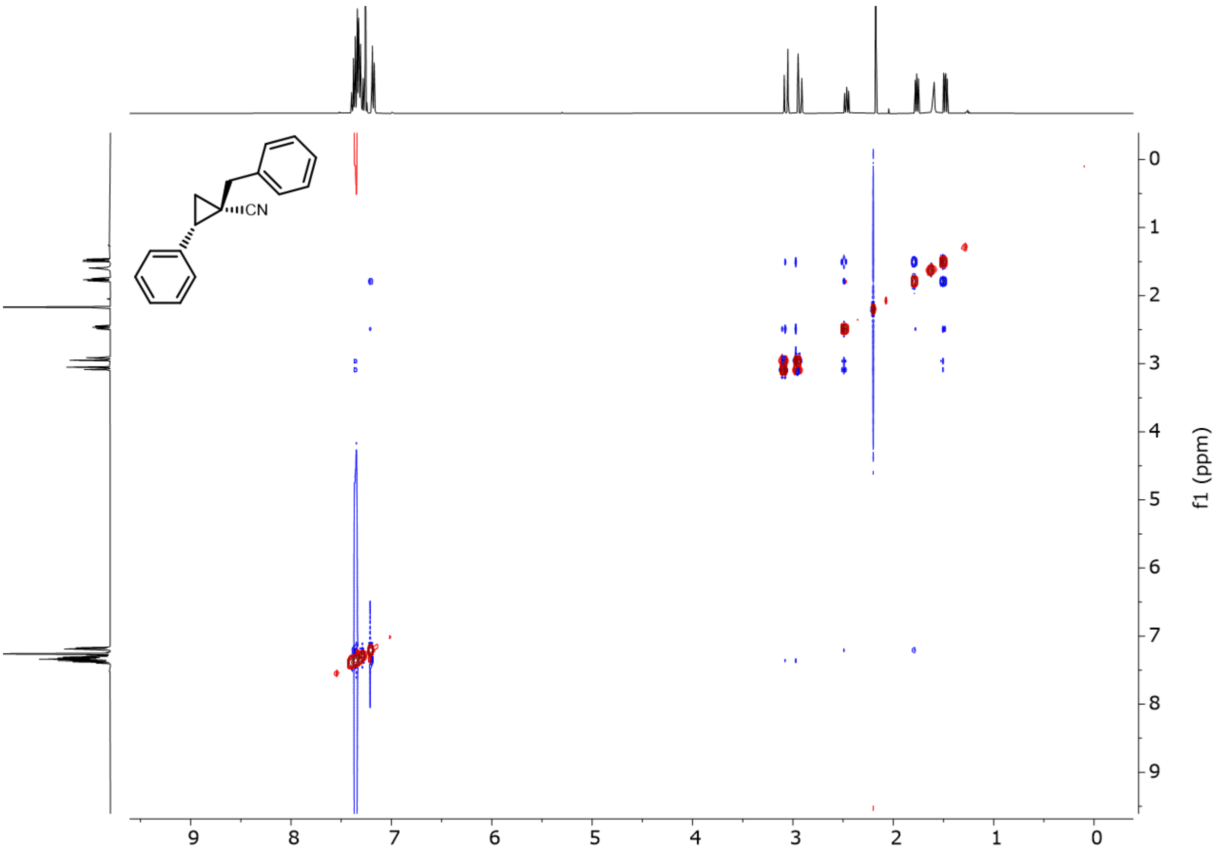

[*trans*-11p] ^{1}H, $CDCl_3$, 400 MHz

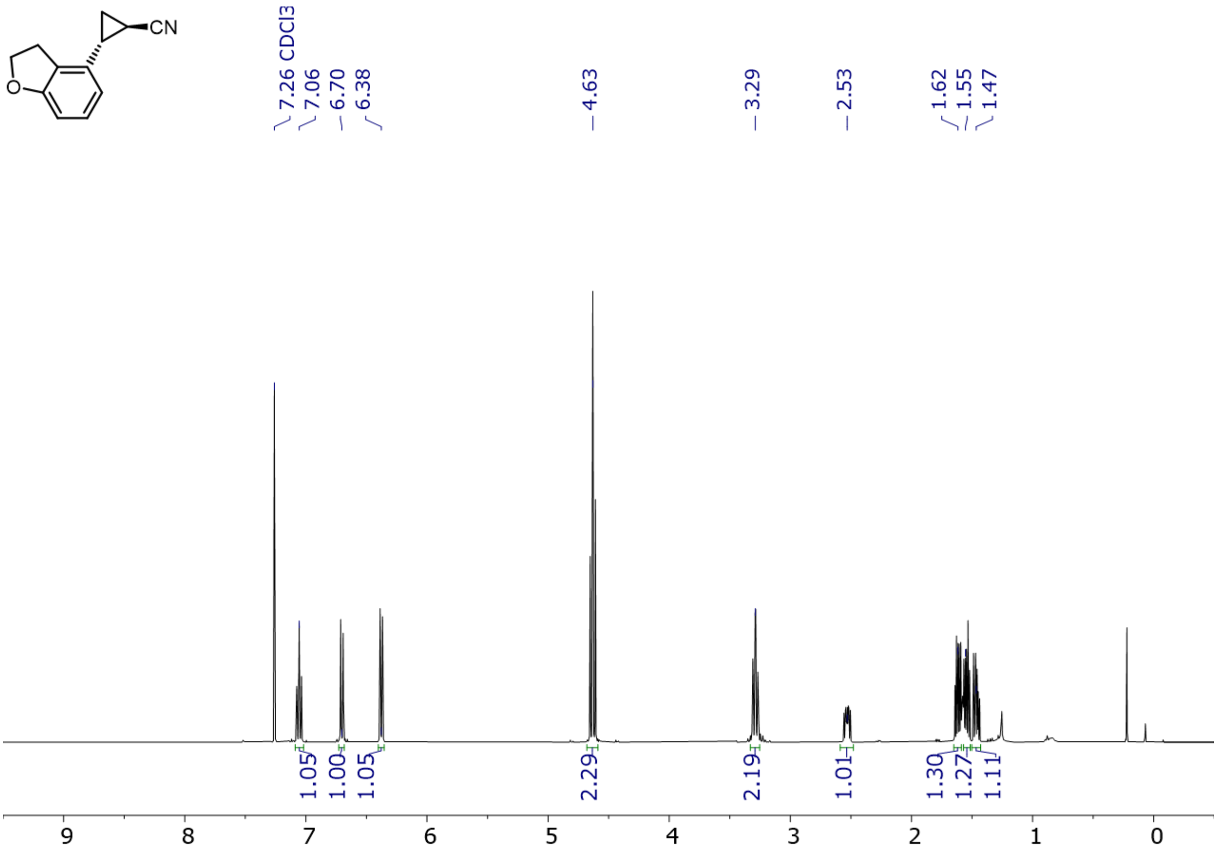

[*trans*-11o] ^{13}C, $CDCl_3$, 101 MHz

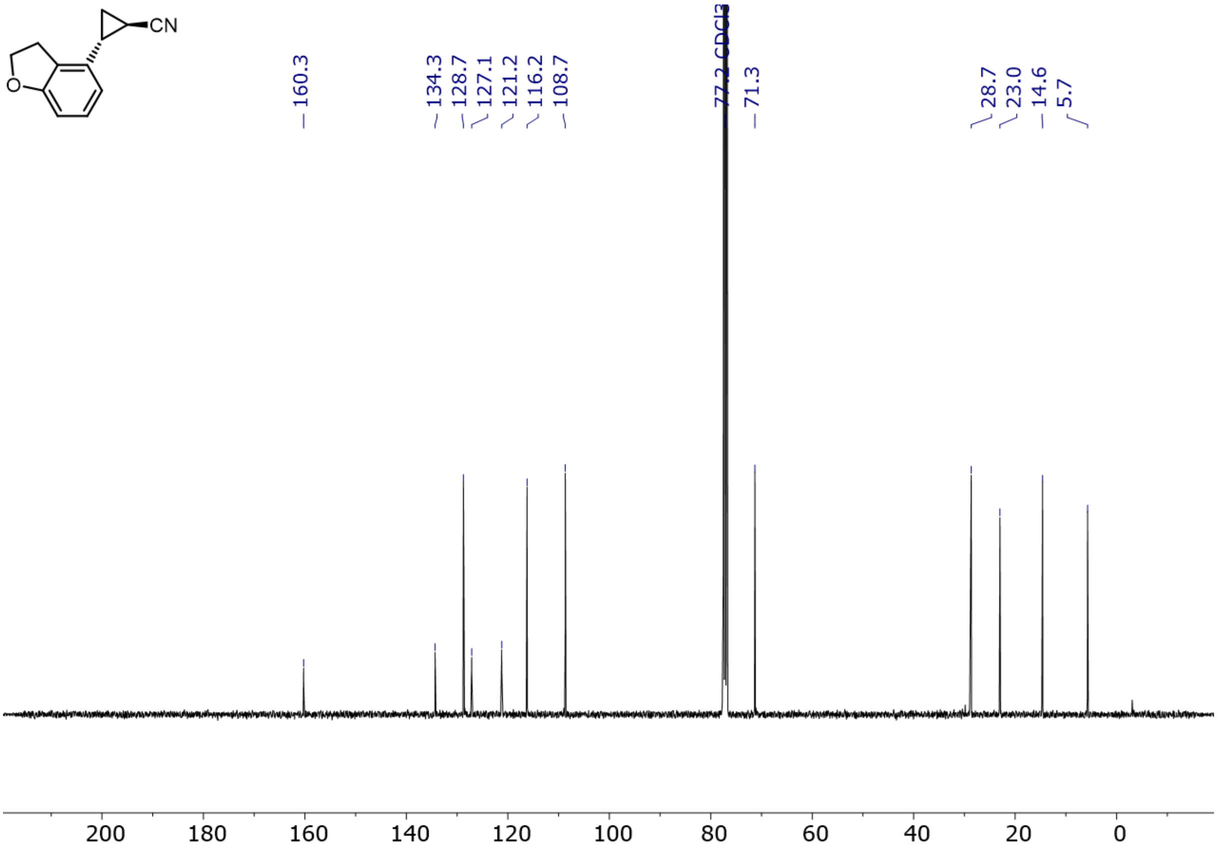

HPLC data

[6a]

top: racemic sample

bottom: enantioenriched sample

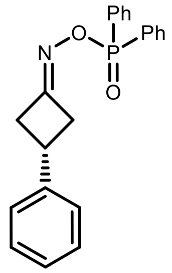

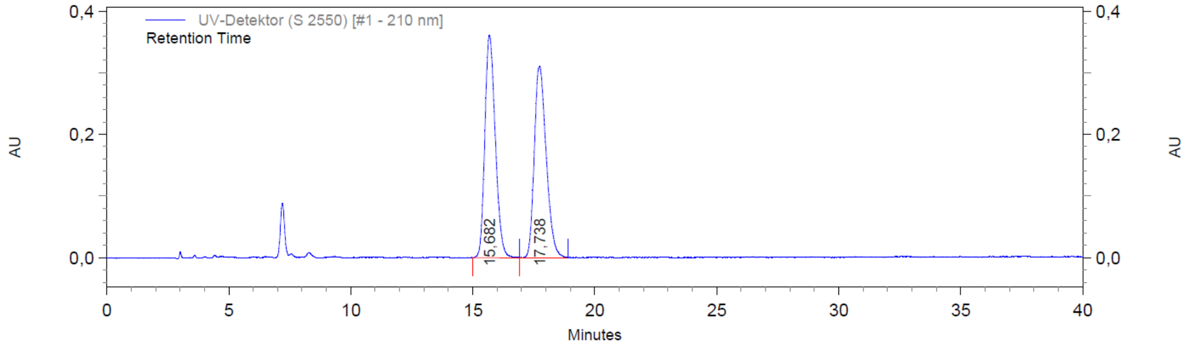

UV-Detektor (S 2550) [#1 - 210 nm] Results

Retention Time	Area	Area %
15,682	10736413	50,47
17,738	10537785	49,53

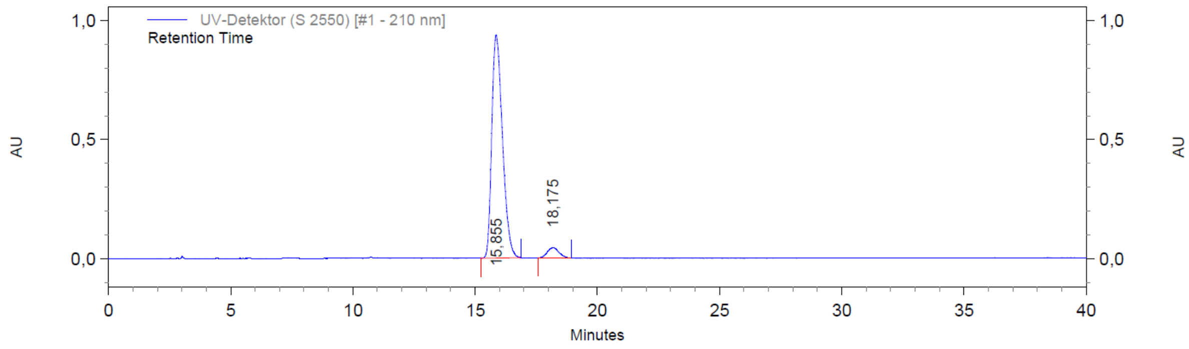

UV-Detektor (S 2550) [#1 - 210 nm] Results

Retention Time	Area	Area %
15,855	28423074	95,19
18,175	1435209	4,81

[6a] *1.0 mmol scale*

top: racemic sample

bottom: enantioenriched sample

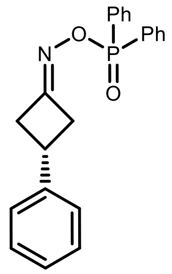

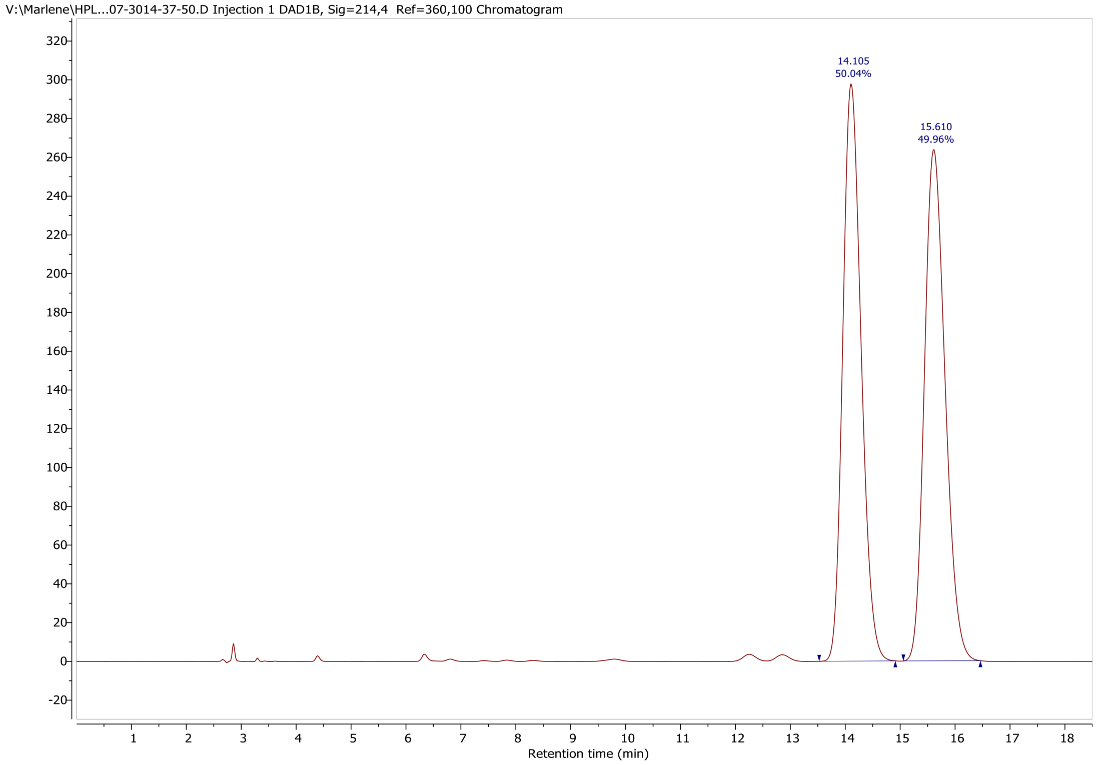

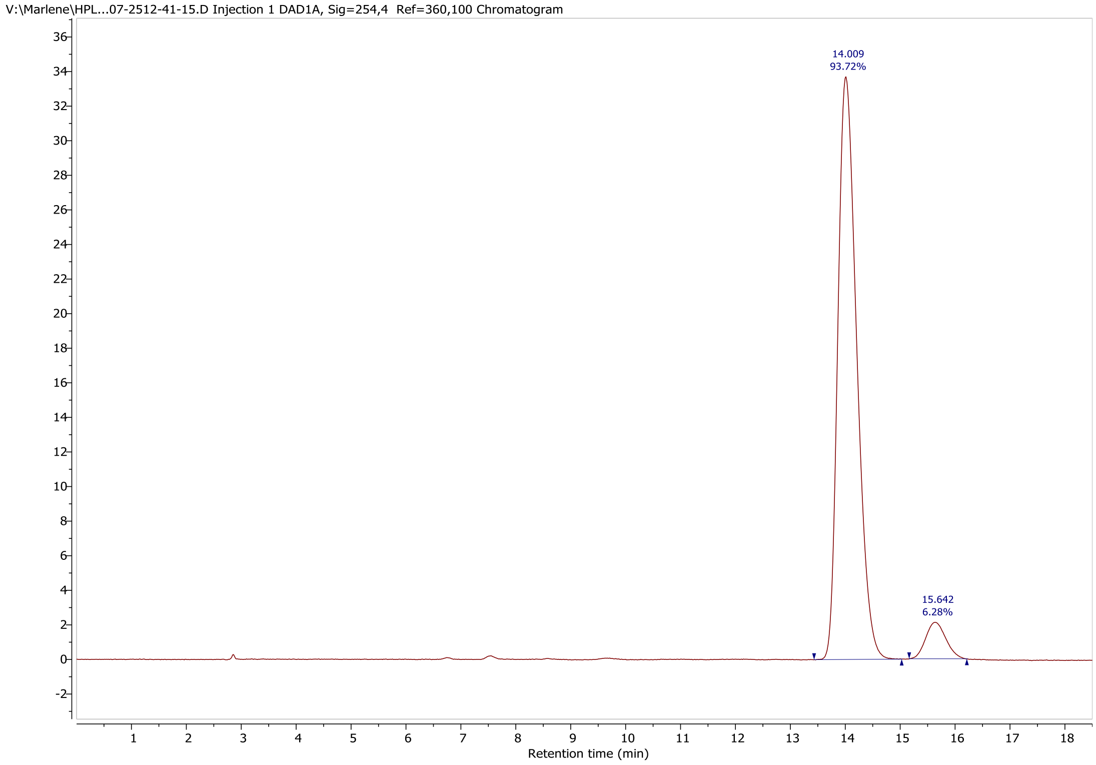

[6b]

top: racemic sample

bottom: enantioenriched sample

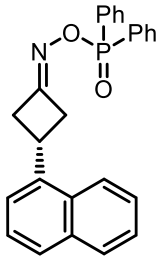

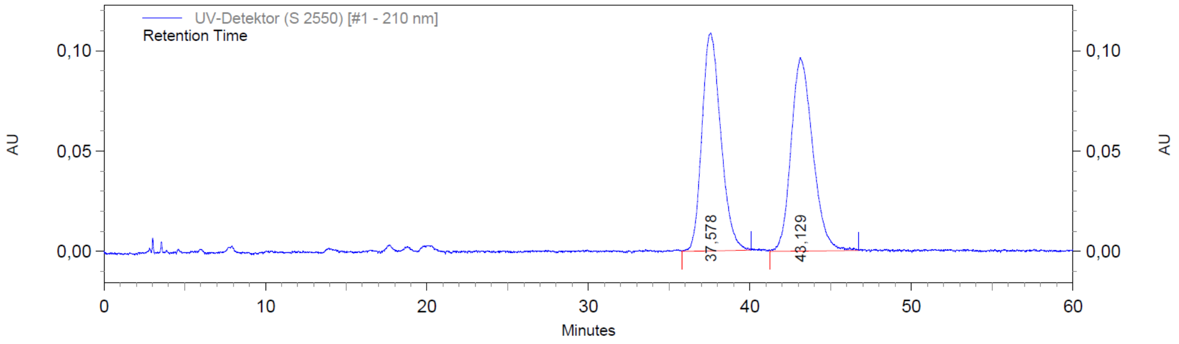

UV-Detektor (S 2550) [#1 - 210 nm] Results

Retention Time	Area	Area %
37,578	8803911	49,50
43,129	8981887	50,50

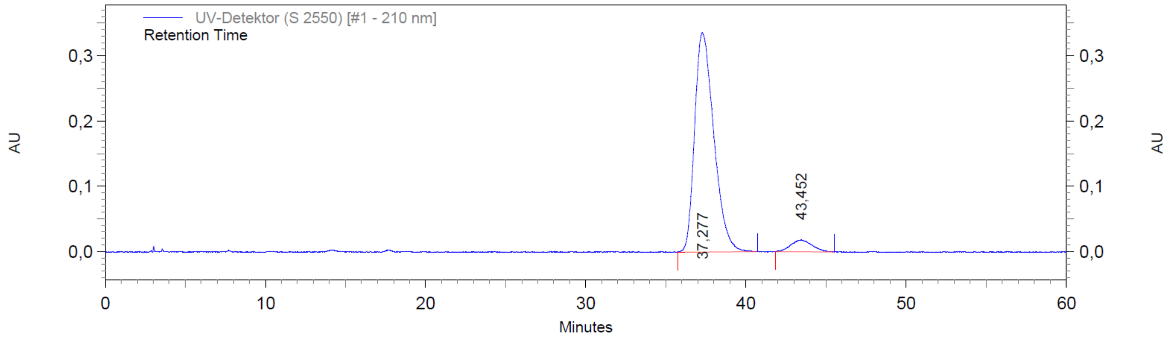

UV-Detektor (S 2550) [#1 - 210 nm] Results

Retention Time	Area	Area %
37,277	28079646	94,31
43,452	1692954	5,69

[6c]

top: racemic sample

bottom: enantioenriched sample

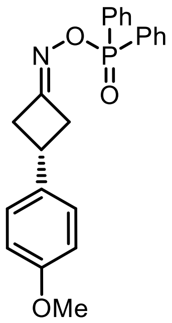

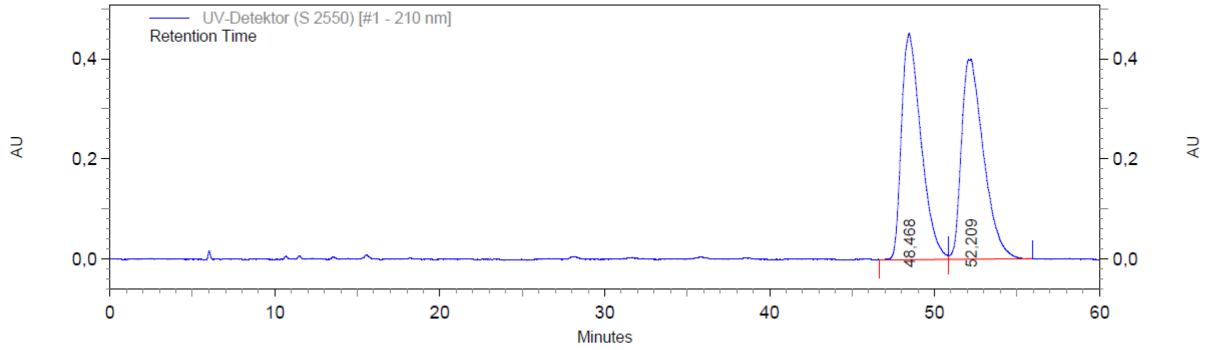

UV-Detektor (S 2550) [#1 - 210 nm] Results

Retention Time	Area	Area %
48,468	37413512	49,92
52,209	37531449	50,08

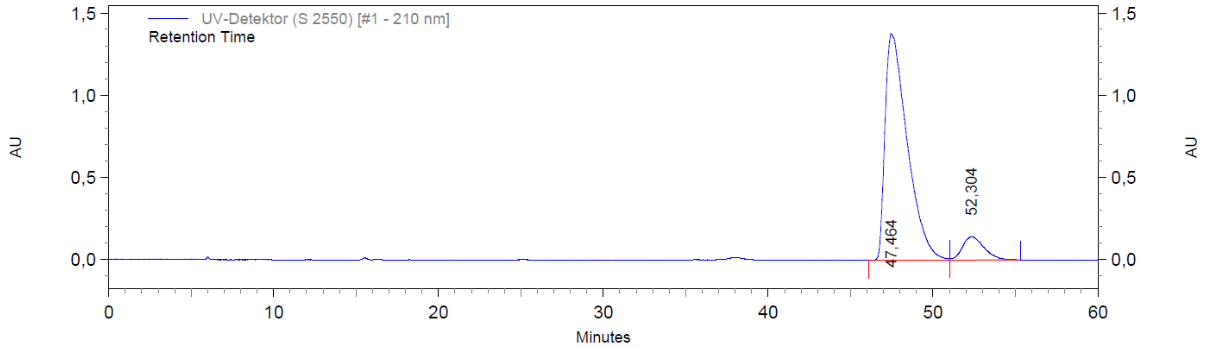

UV-Detektor (S 2550) [#1 - 210 nm] Results

Retention Time	Area	Area %
47,464	129075456	90,81
52,304	13059295	9,19

[6d]

top: racemic sample

bottom: enantioenriched sample

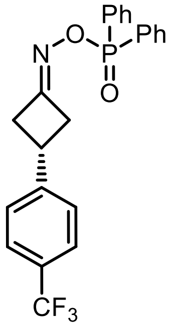

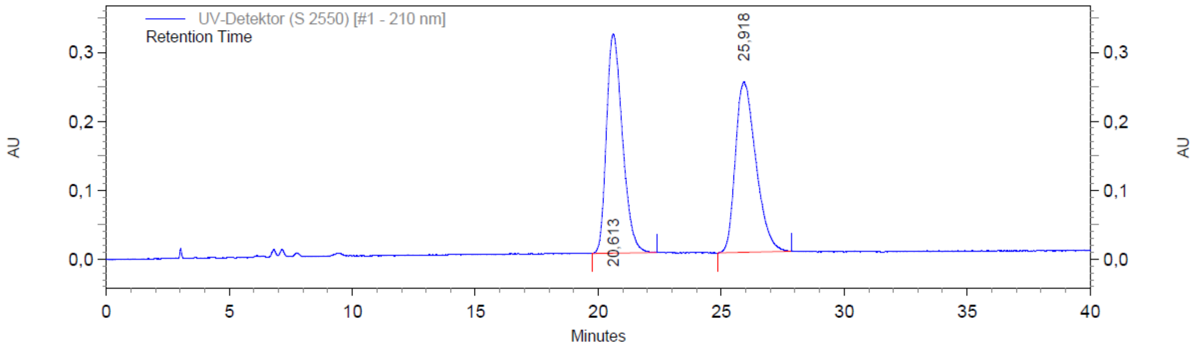

UV-Detektor (S 2550) [#1 - 210 nm] Results

Retention Time	Area	Area %
20,613	14331333	49,95
25,918	14362455	50,05

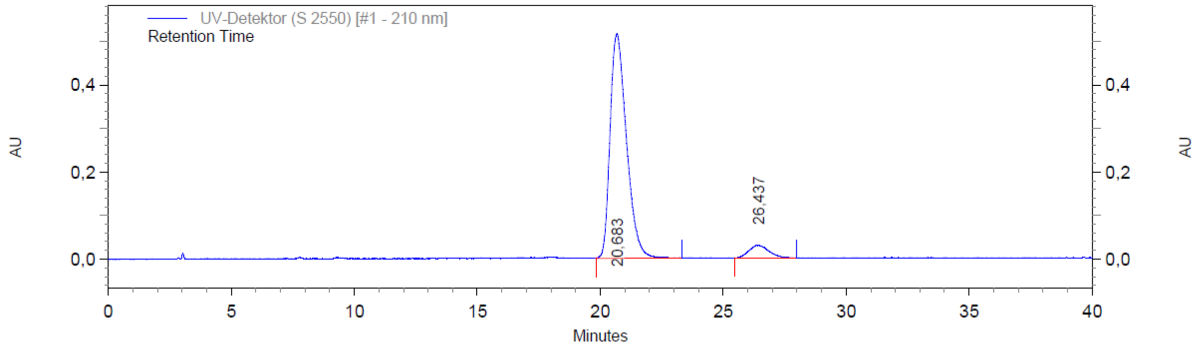

UV-Detektor (S 2550) [#1 - 210 nm] Results

Retention Time	Area	Area %
20,683	23852673	93,40
26,437	1686509	6,60

[6e]

top: racemic sample

bottom: enantioenriched sample

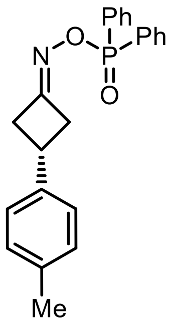

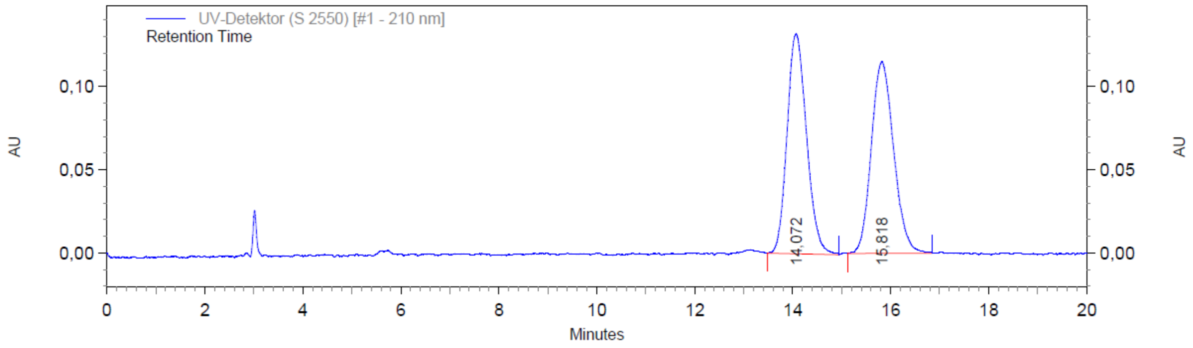

UV-Detektor (S 2550) [#1 - 210 nm] Results

Retention Time	Area	Area %
14,072	3654970	50,00
15,818	3655235	50,00

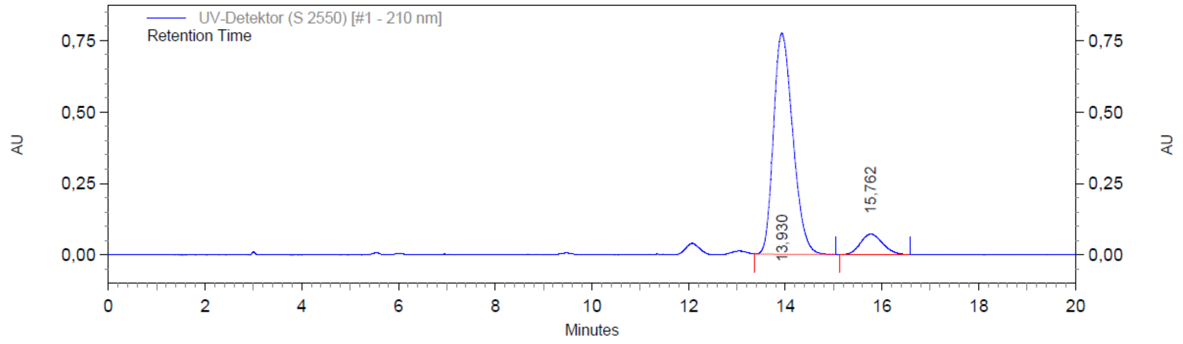

UV-Detektor (S 2550) [#1 - 210 nm] Results

Retention Time	Area	Area %
13,930	21528170	90,51
15,762	2258249	9,49

[6f]

top: racemic sample

bottom: enantioenriched sample

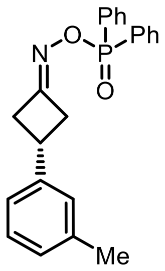

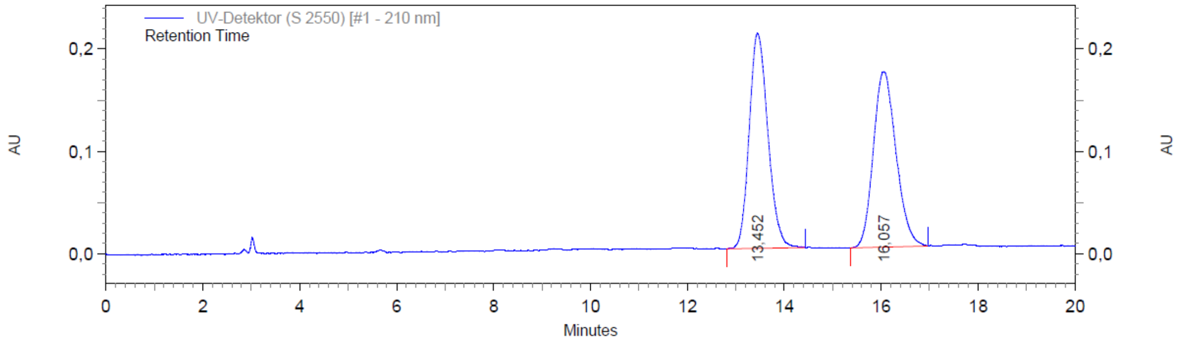

UV-Detektor (S 2550) [#1 - 210 nm] Results

Retention Time	Area	Area %
13,452	5632452	50,26
16,057	5574062	49,74

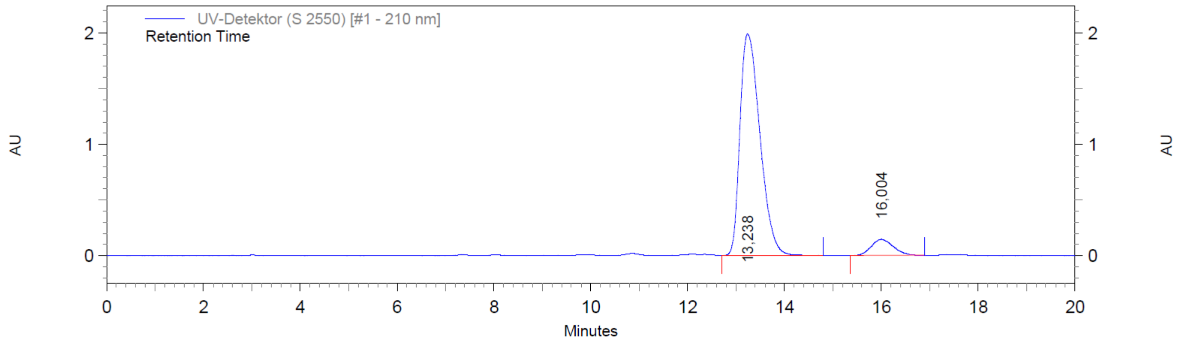

UV-Detektor (S 2550) [#1 - 210 nm] Results

Retention Time	Area	Area %
13,238	57022913	92,57
16,004	4579880	7,43

[6g]

top: racemic sample

bottom: enantioenriched sample

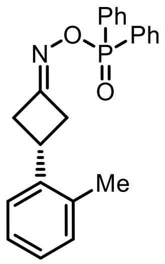

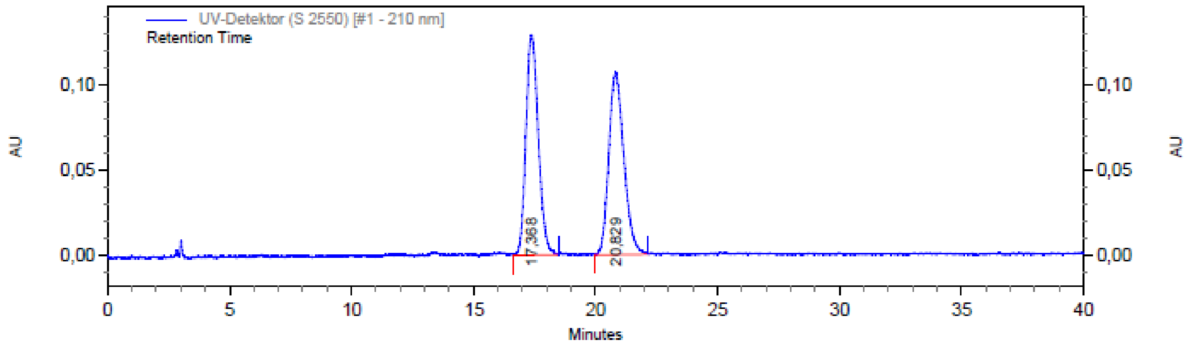

UV-Detektor (S 2550) [#1 - 210 nm] Results

Retention Time	Area	Area %
17,368	4570150	49,75
20,829	4615575	50,25

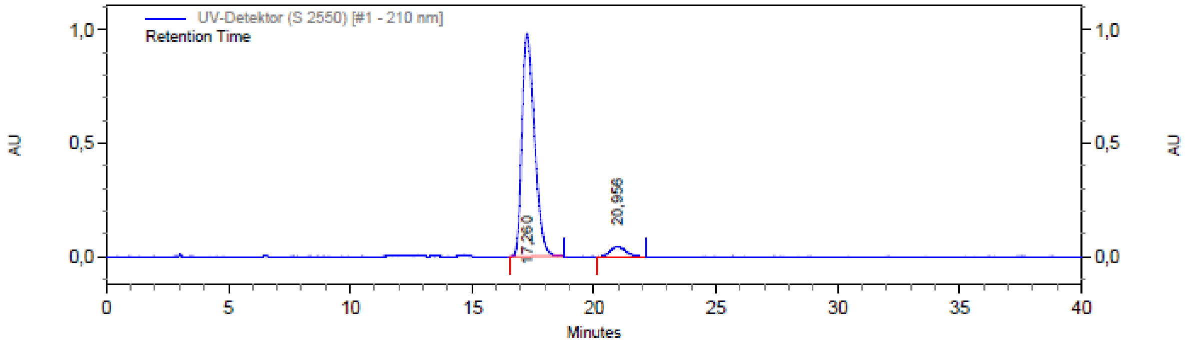

UV-Detektor (S 2550) [#1 - 210 nm] Results

Retention Time	Area	Area %
17,260	35175032	94,97
20,956	1863614	5,03

[6h]

top: racemic sample

bottom: enantioenriched sample

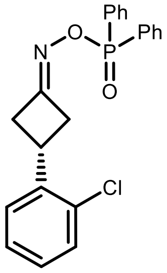

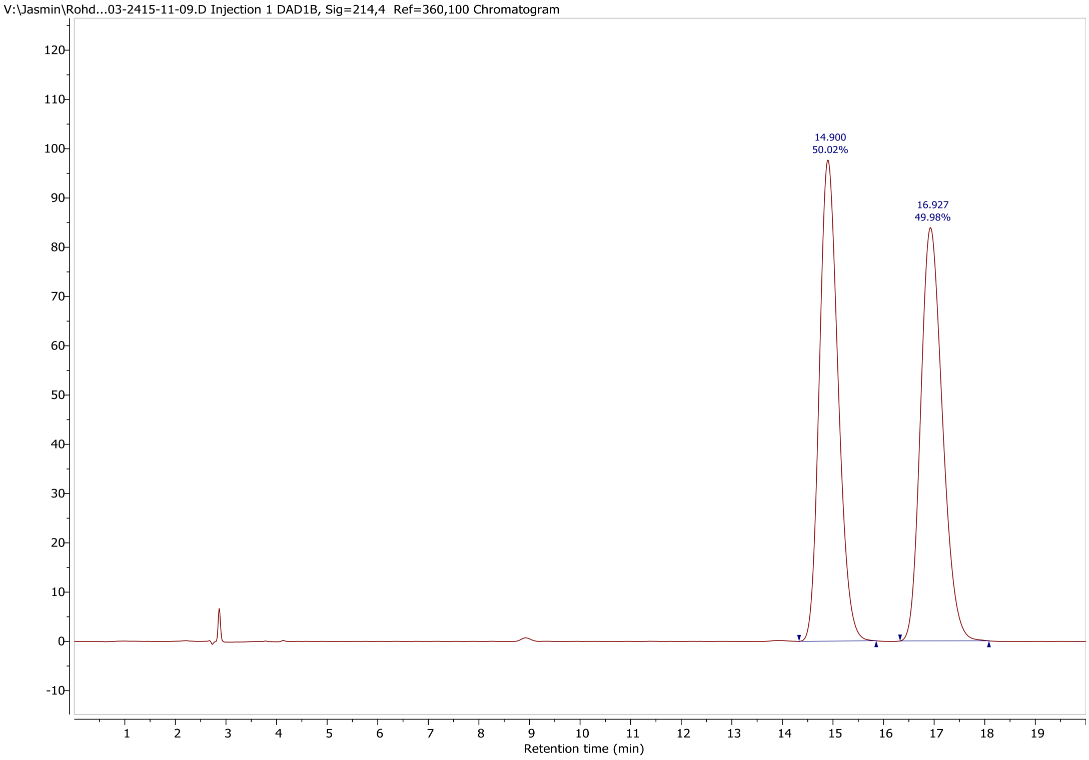

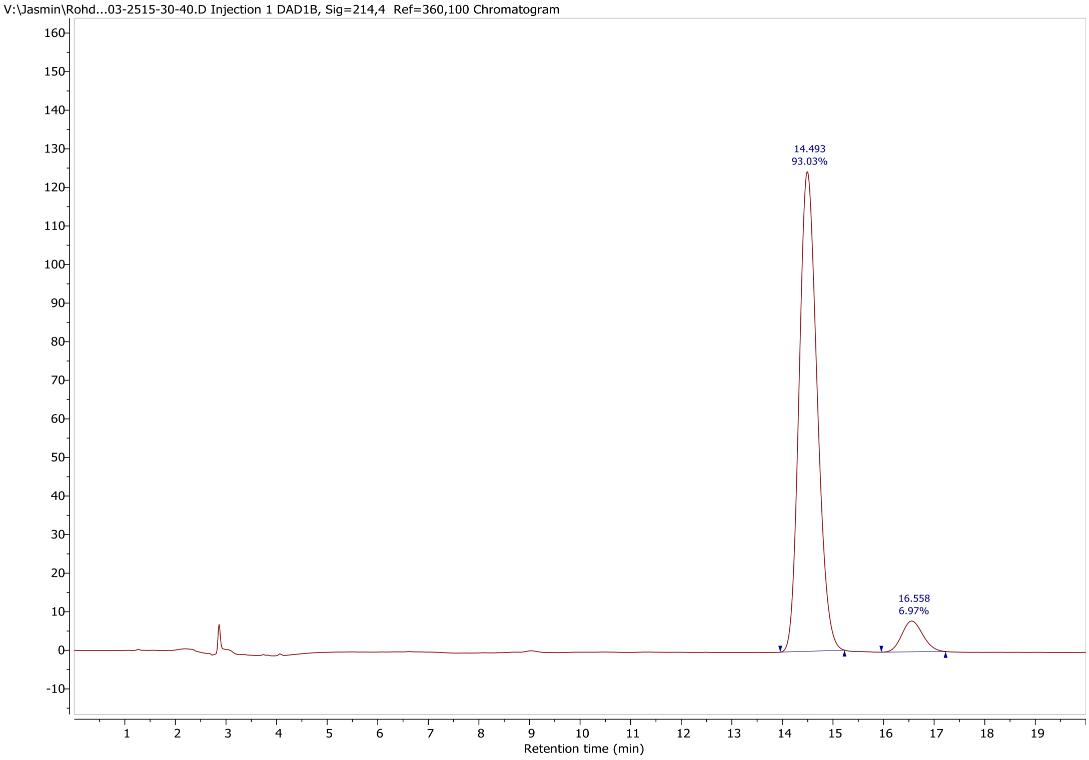

[6i]

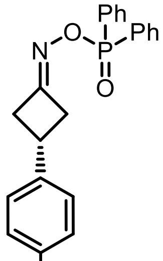

top: racemic sample

bottom: enantioenriched sample

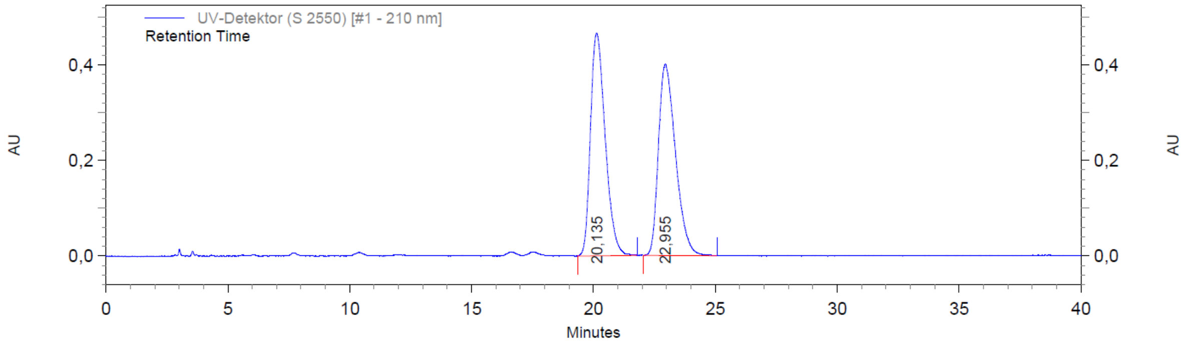

UV-Detektor (S 2550) [#1 - 210 nm] Results

Retention Time	Area	Area %
20,135	19575625	49,98
22,955	19591064	50,02

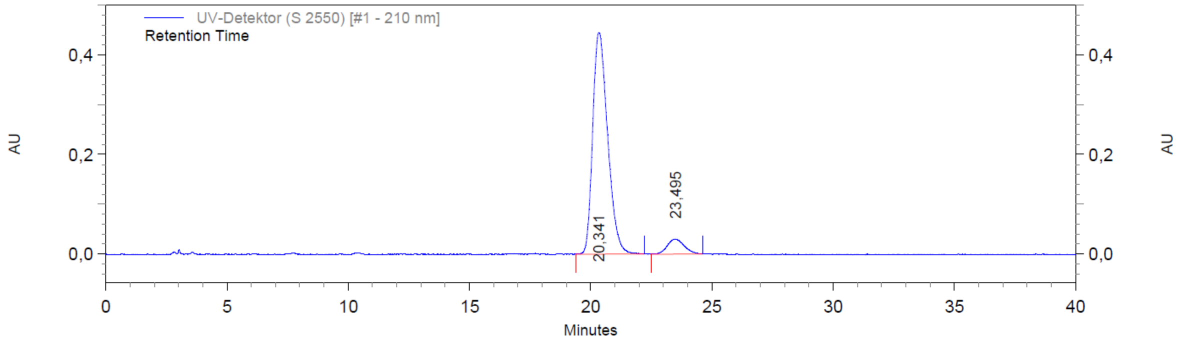

UV-Detektor (S 2550) [#1 - 210 nm] Results

Retention Time	Area	Area %
20,341	19041919	92,72
23,495	1494371	7,28

[6j]

top: racemic sample

bottom: enantioenriched sample

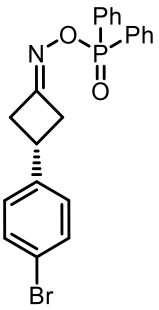

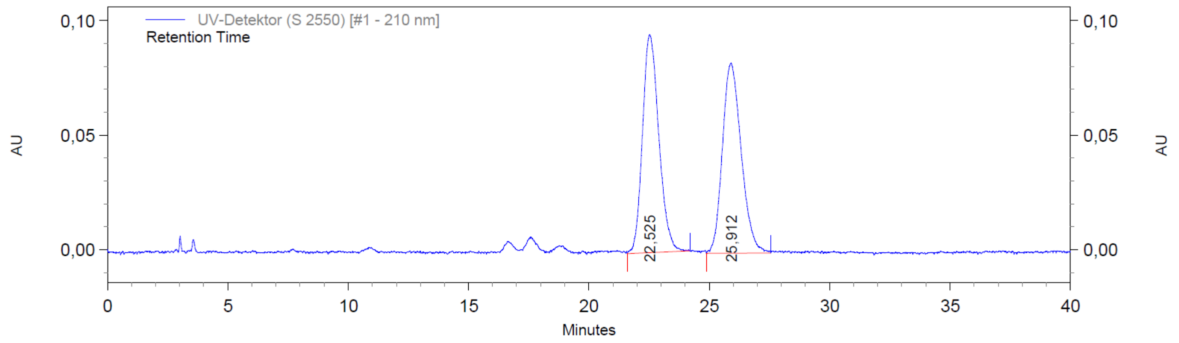

UV-Detektor (S 2550) [#1 - 210 nm] Results

Retention Time	Area	Area %
22,525	4492343	49,91
25,912	4508917	50,09

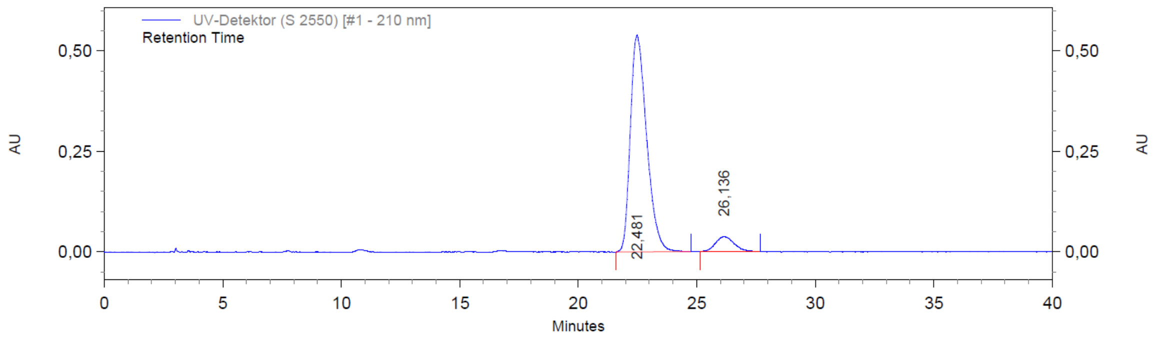

UV-Detektor (S 2550) [#1 - 210 nm] Results

Retention Time	Area	Area %
22,481	25816737	92,51
26,136	2089656	7,49

[6k]

top: racemic sample

bottom: enantioenriched sample

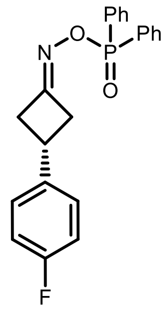

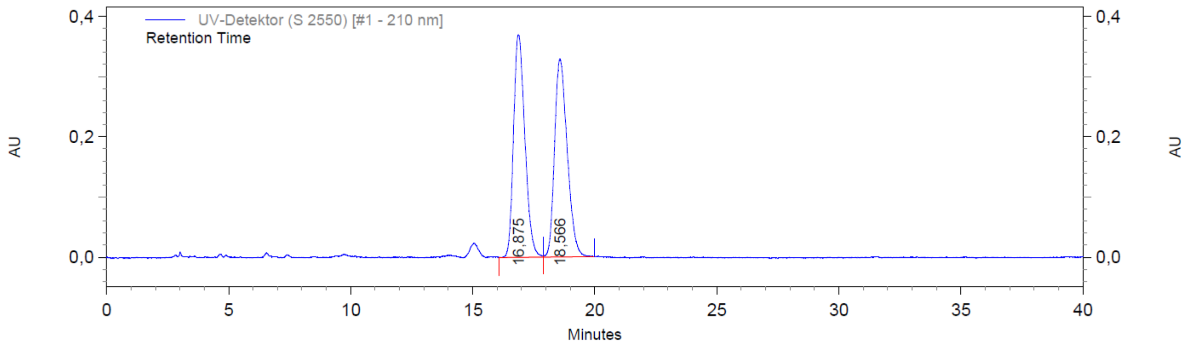

UV-Detektor (S 2550) [#1 - 210 nm] Results

Retention Time	Area	Area %
16,875	11975495	50,17
18,566	11892150	49,83

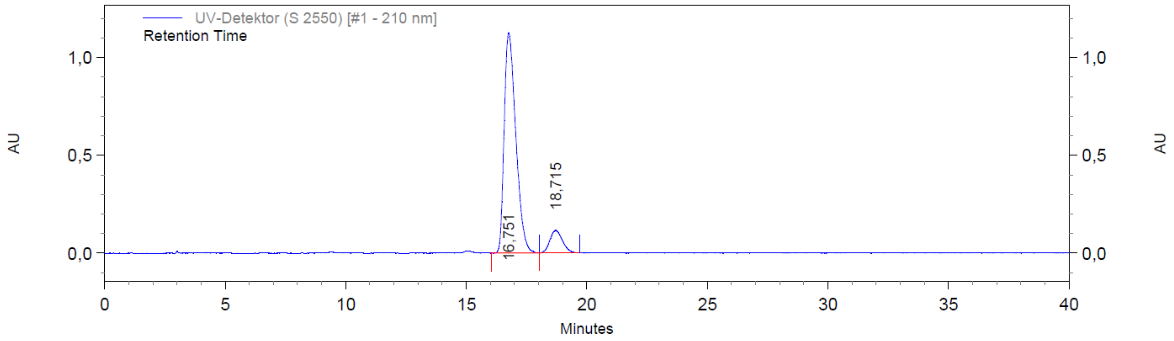

UV-Detektor (S 2550) [#1 - 210 nm] Results

Retention Time	Area	Area %
16,751	37770575	90,07
18,715	4164170	9,93

[6l]

top: racemic sample

bottom: enantioenriched sample

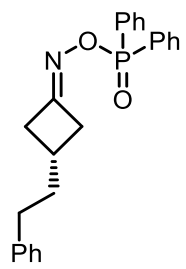

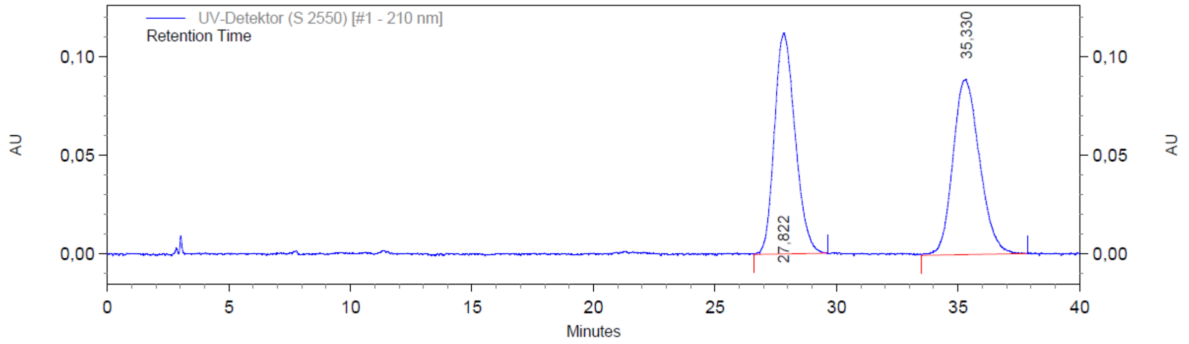

UV-Detektor (S 2550) [#1 - 210 nm] Results

Retention Time	Area	Area %
27,822	6655716	49,49
35,330	6792447	50,51

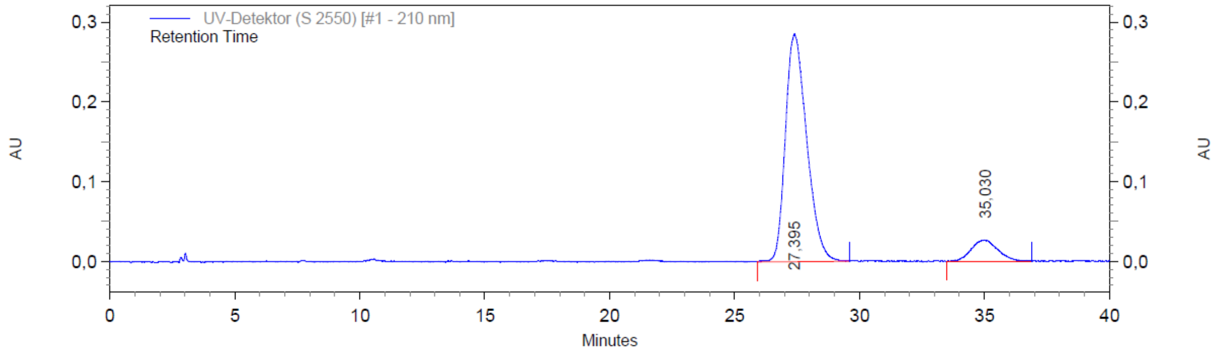

UV-Detektor (S 2550) [#1 - 210 nm] Results

Retention Time	Area	Area %
27,395	16990489	89,24
35,030	2047978	10,76

[6m]

top: racemic sample

bottom: enantioenriched sample

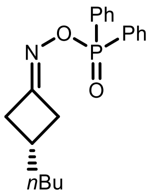

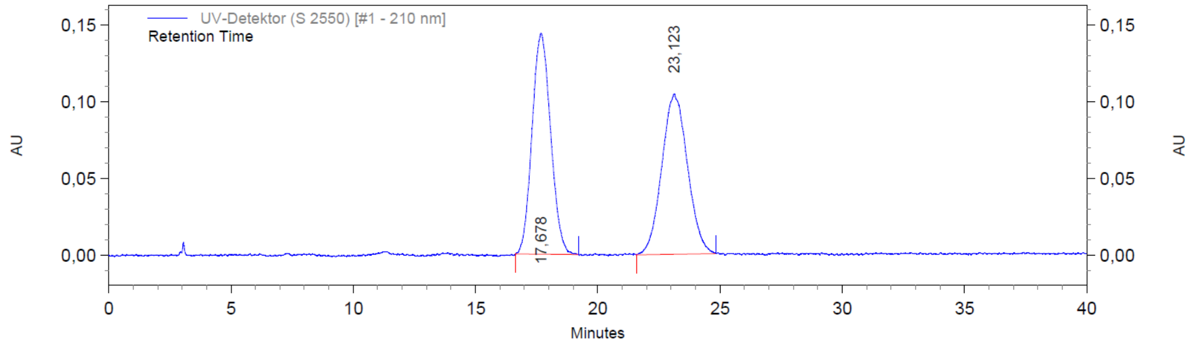

UV-Detektor (S 2550) [#1 - 210 nm] Results

Retention Time	Area	Area %
17,678	7598014	49,88
23,123	7634328	50,12

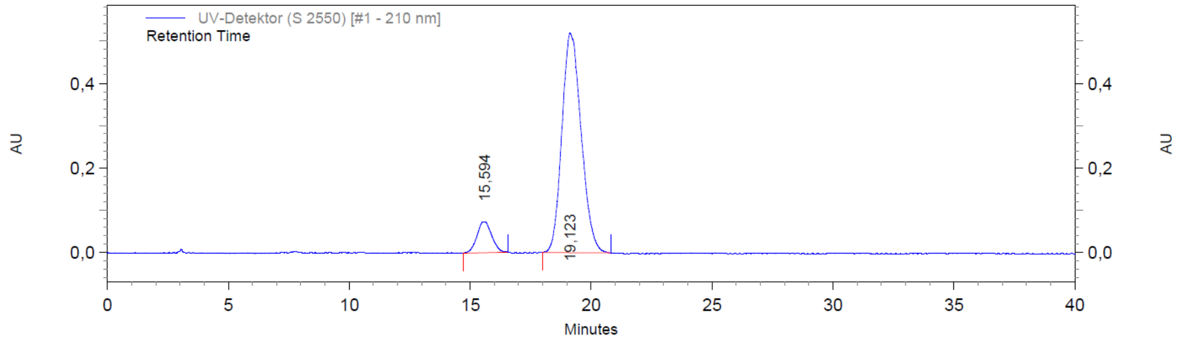

UV-Detektor (S 2550) [#1 - 210 nm] Results

Retention Time	Area	Area %
15,594	3089478	9,67
19,123	28870319	90,33

[6n]

top: racemic sample

bottom: enantioenriched sample

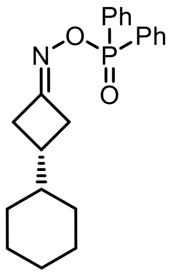

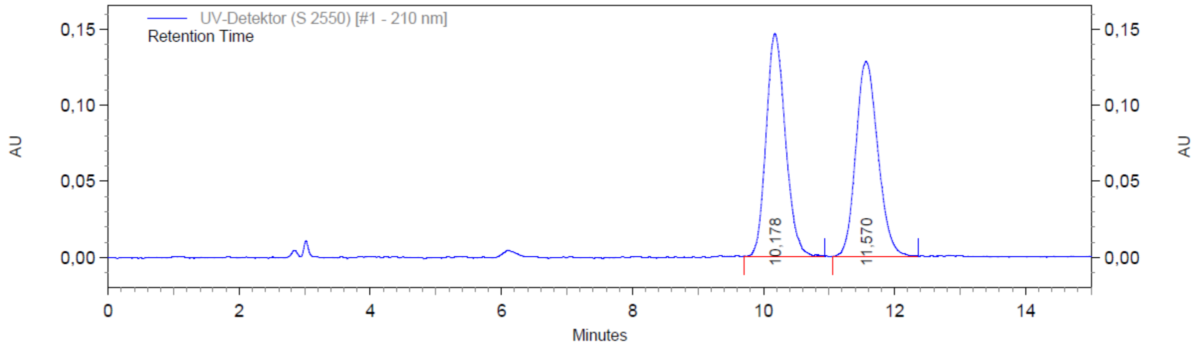

UV-Detektor (S 2550) [#1 - 210 nm] Results

Retention Time	Area	Area %
10,178	3011903	49,93
11,570	3020311	50,07

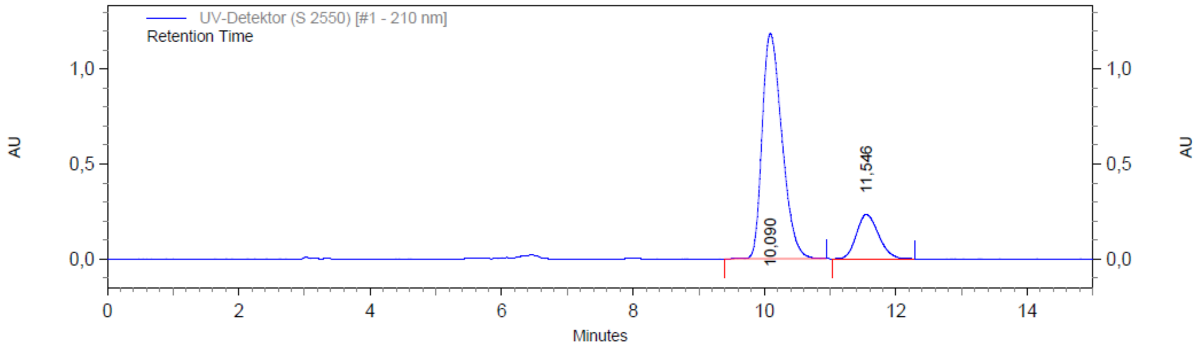

UV-Detektor (S 2550) [#1 - 210 nm] Results

Retention Time	Area	Area %
10,090	24910565	82,04
11,546	5452748	17,96

[6o]

top: racemic sample

bottom: enantioenriched sample

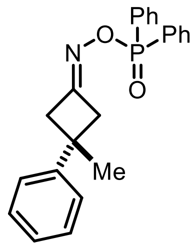

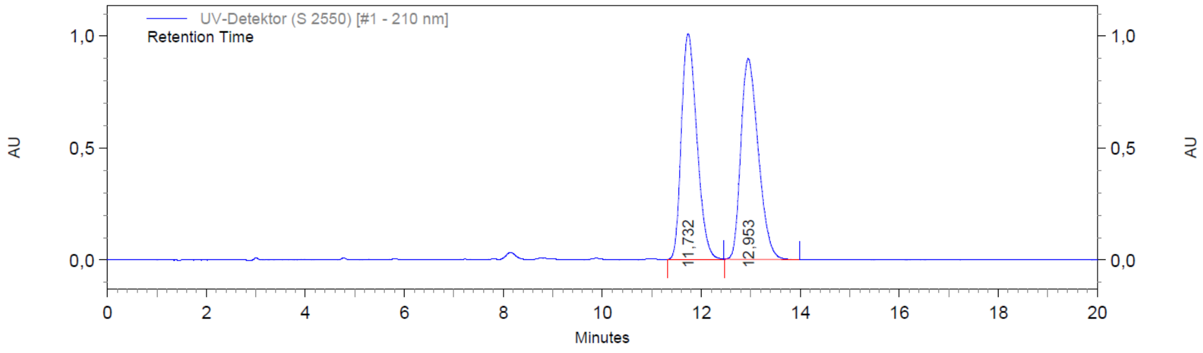

UV-Detektor (S 2550) [#1 - 210 nm] Results

Retention Time	Area	Area %
11,732	21870375	49,87
12,953	21982384	50,13

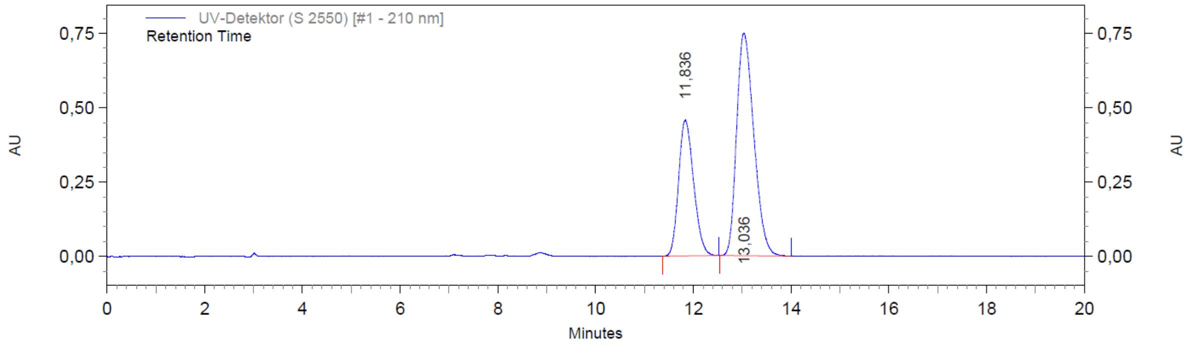

UV-Detektor (S 2550) [#1 - 210 nm] Results

Retention Time	Area	Area %
11,836	9791061	34,77
13,036	18371877	65,23

[*cis*-11a]

top: racemic sample

bottom: enantioenriched sample

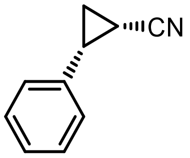

V:\Marlene\HPL...09-1117-15-34.D Injection 1 DAD1B, Sig=214,4 Ref=360,100 Chromatogram

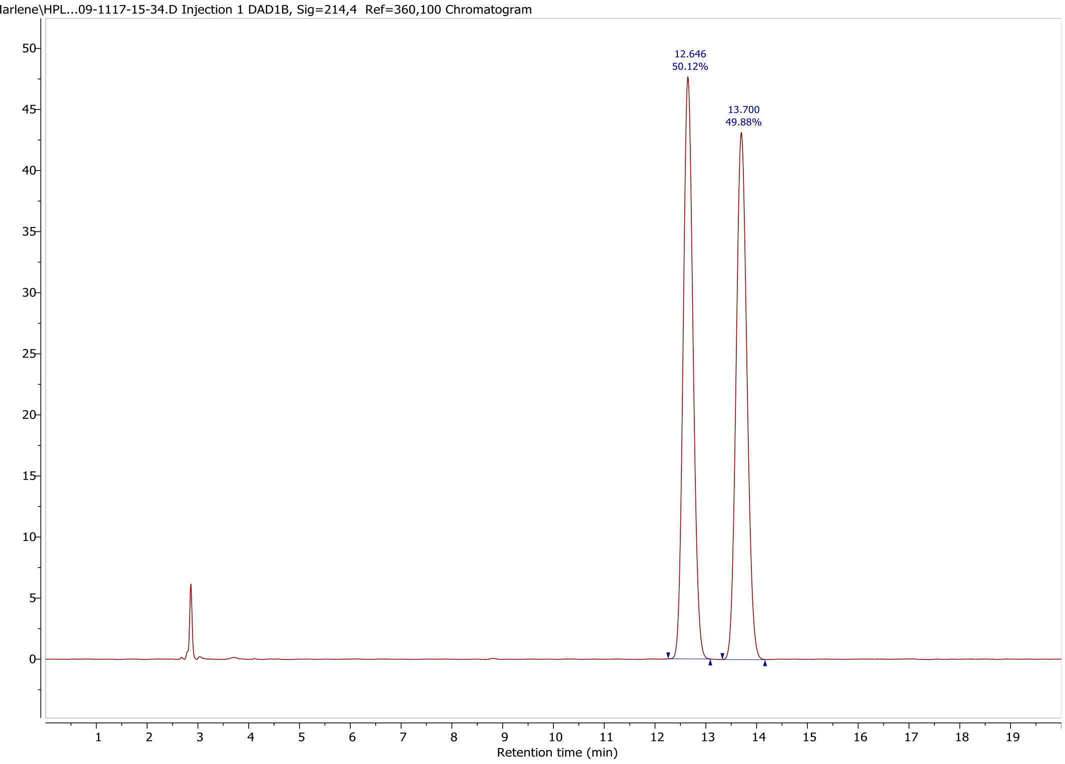

V:\Marlene\HPL...09-1117-37-30.D Injection 1 DAD1B, Sig=214,4 Ref=360,100 Chromatogram

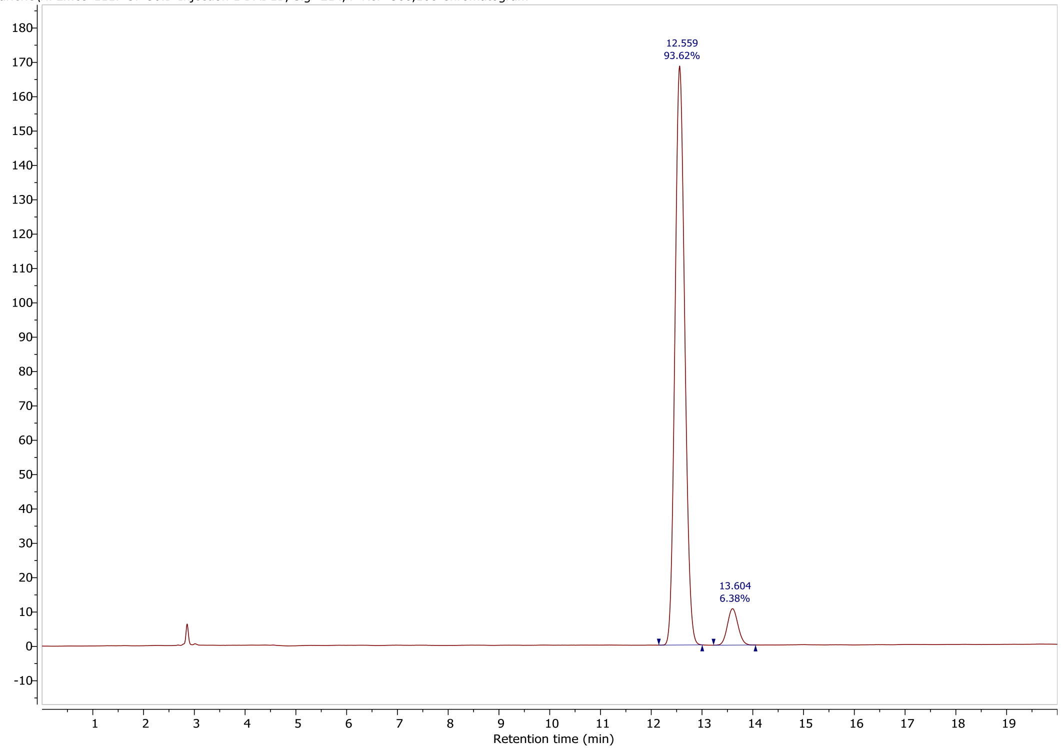

[*ent-cis*-11a]

top: racemic sample

bottom: enantioenriched sample

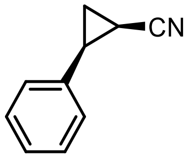

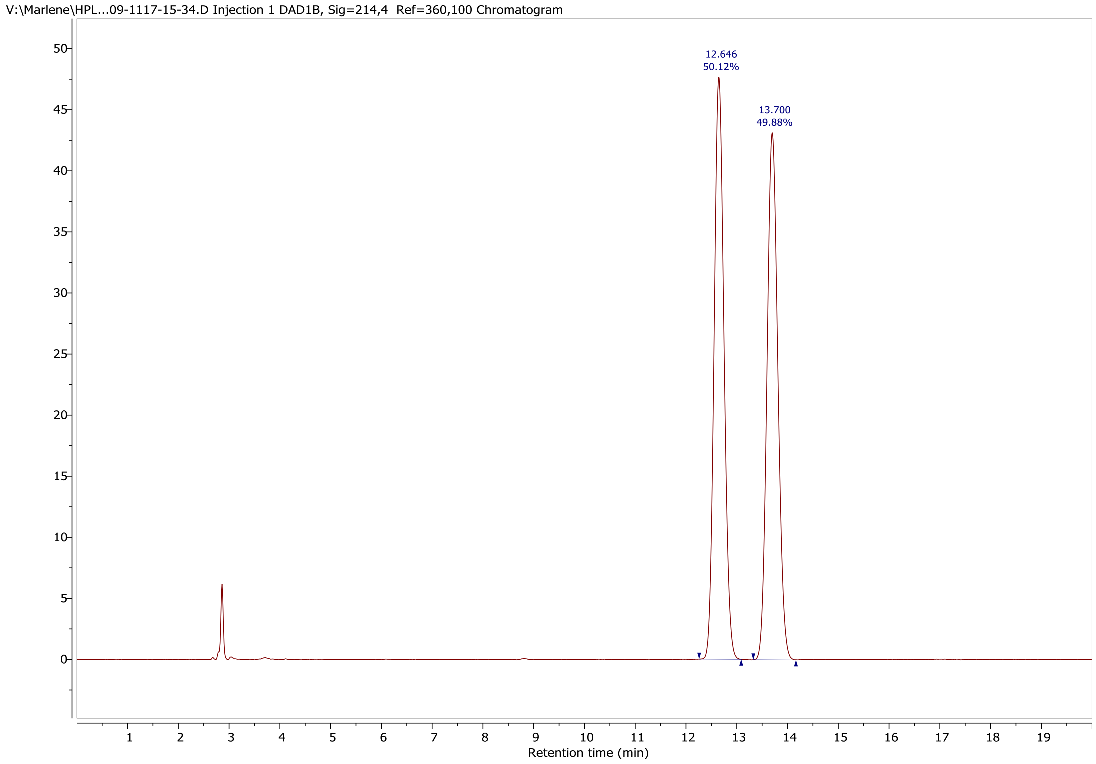

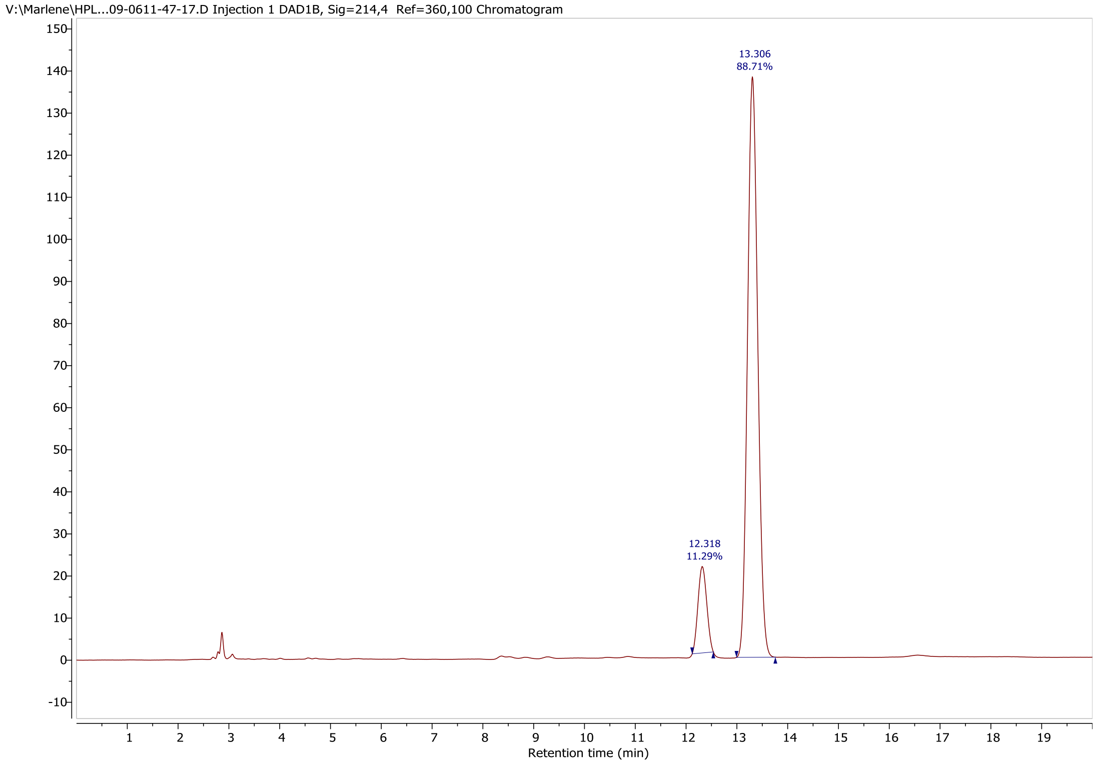

[*trans*-11a]

top: racemic sample

bottom: enantioenriched sample

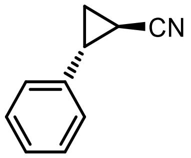

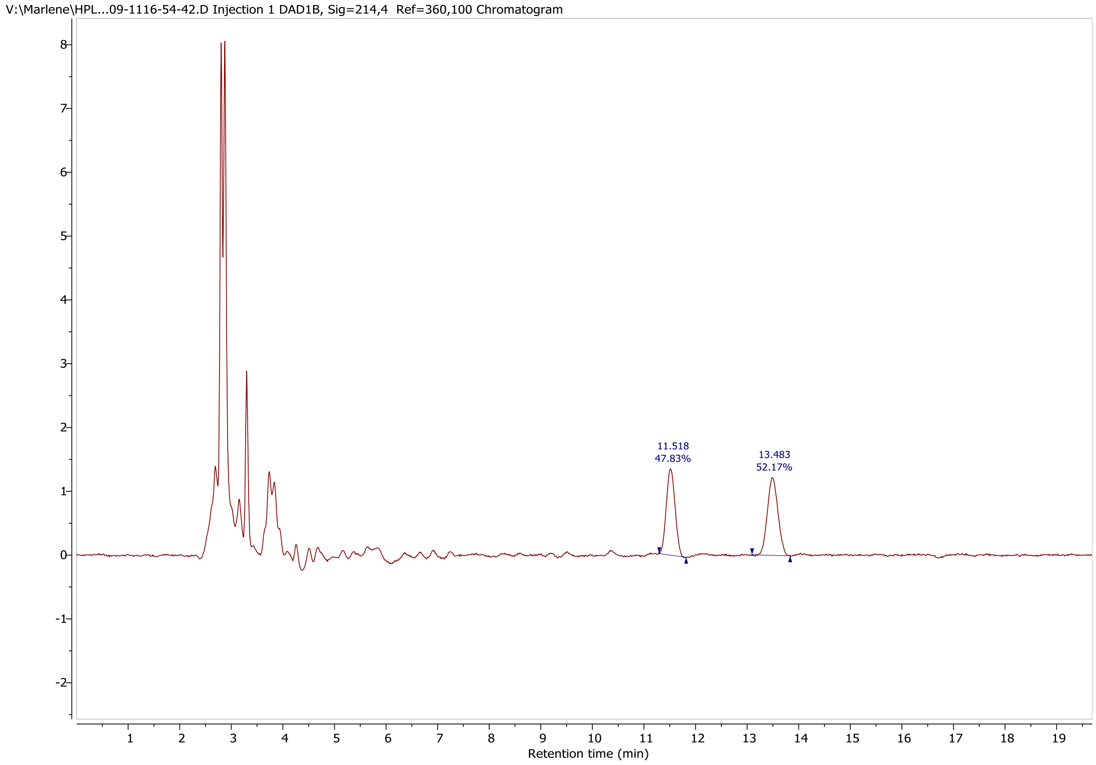

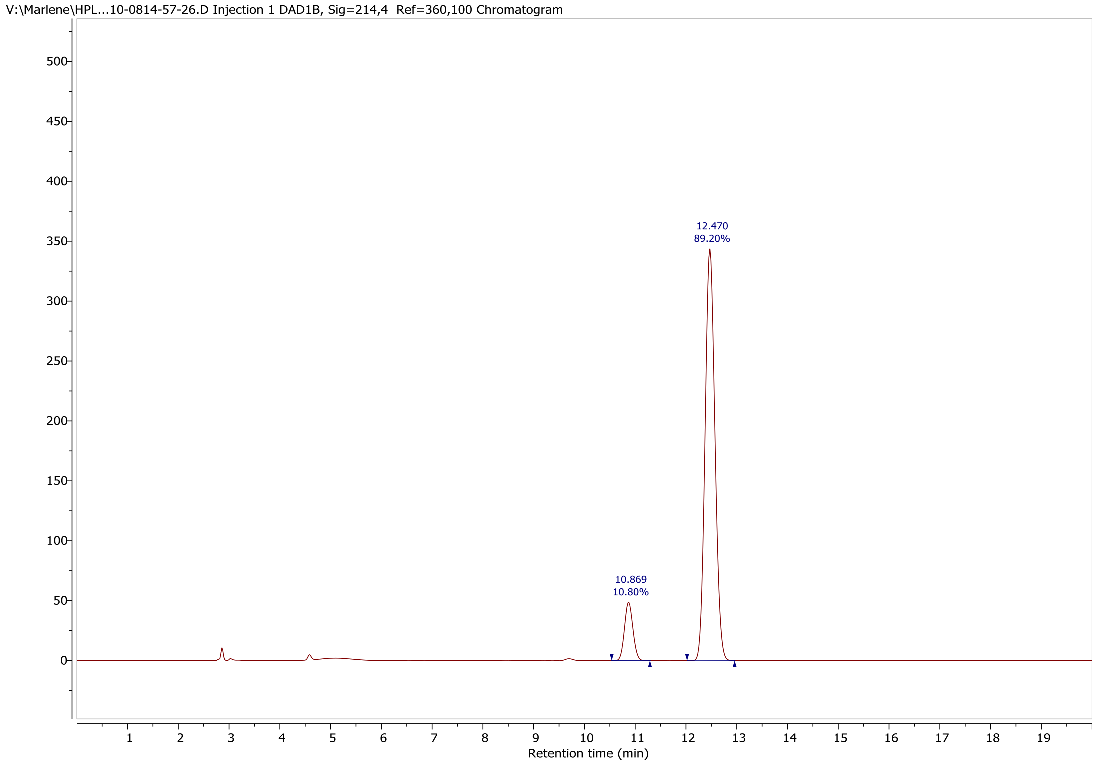

[*ent*-*trans*-11a]

top: racemic sample

bottom: enantioenriched sample

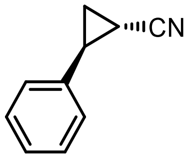

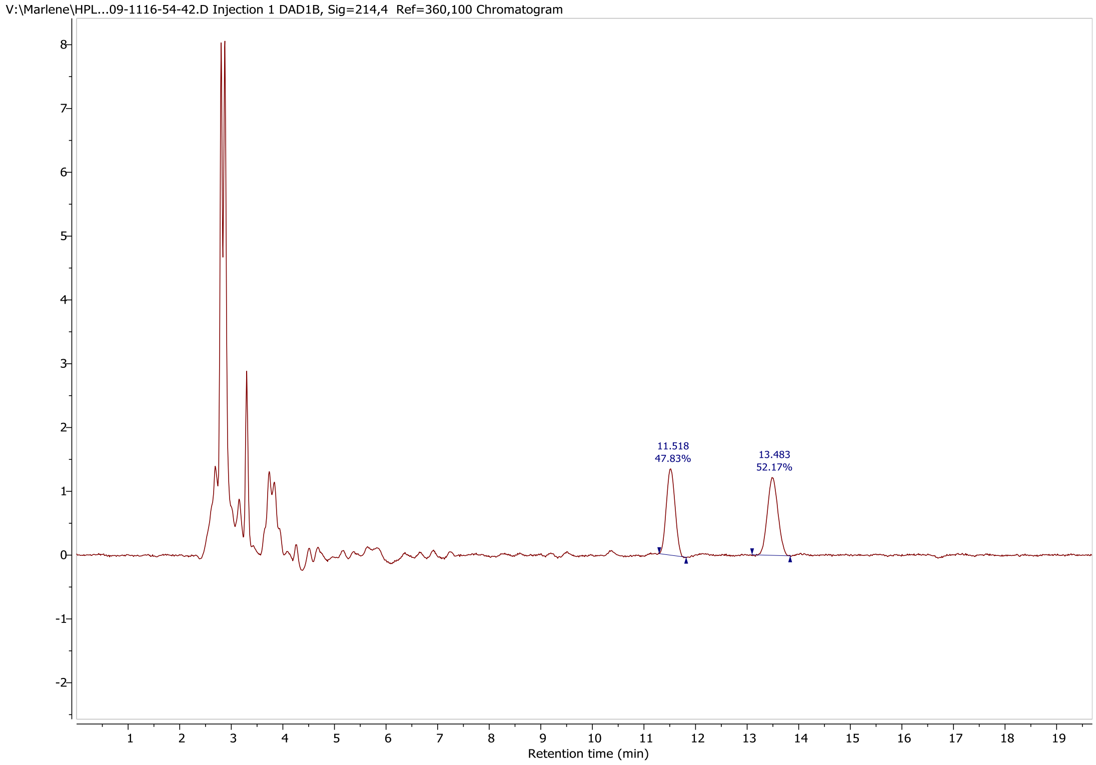

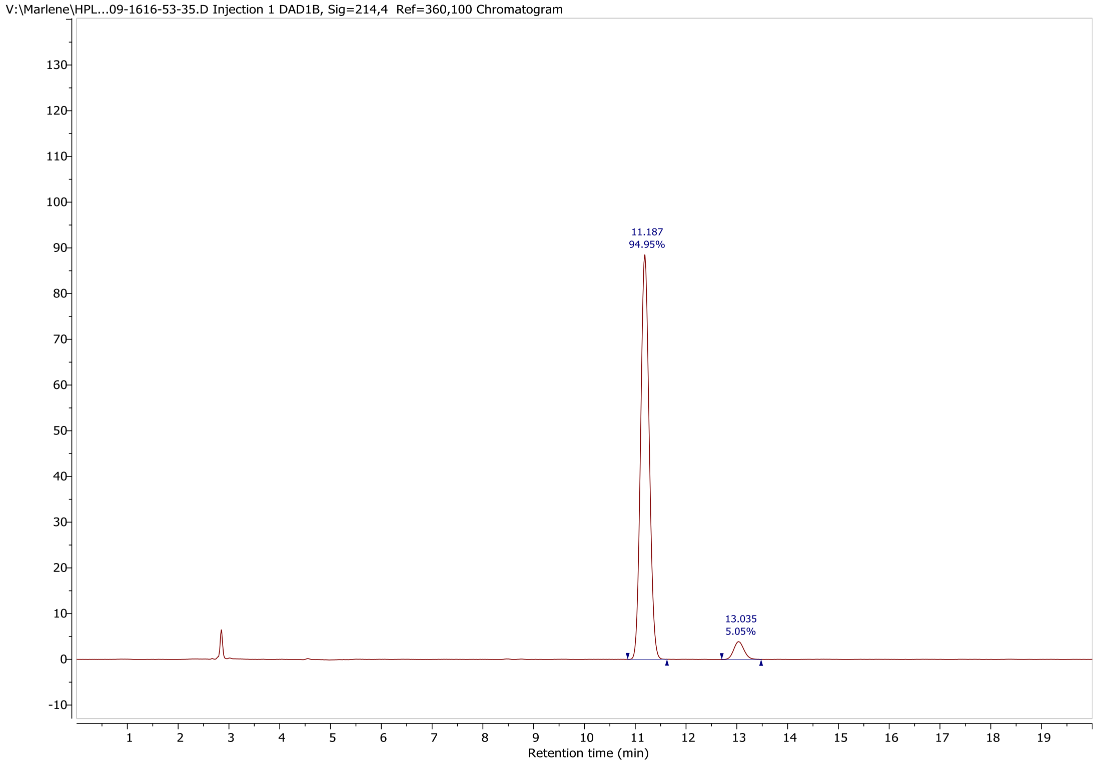

[*cis*-11c]

top: racemic sample

bottom: enantioenriched sample

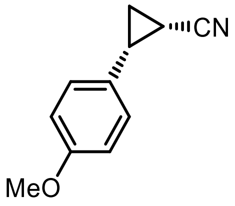

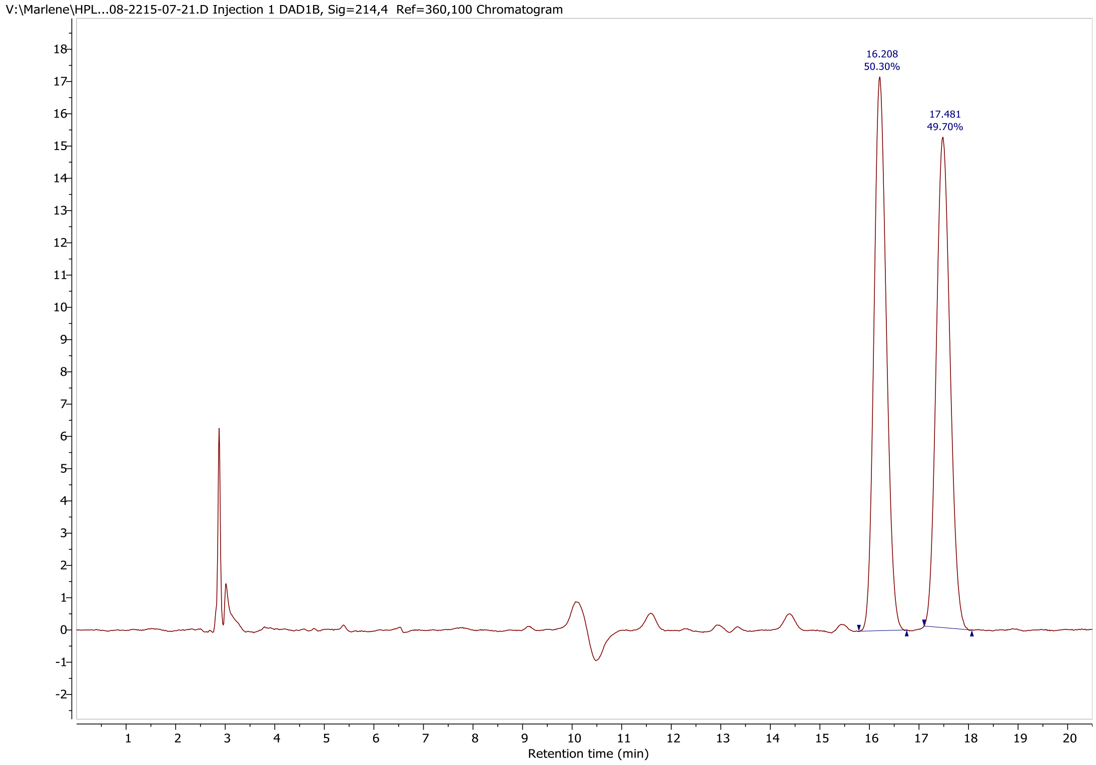

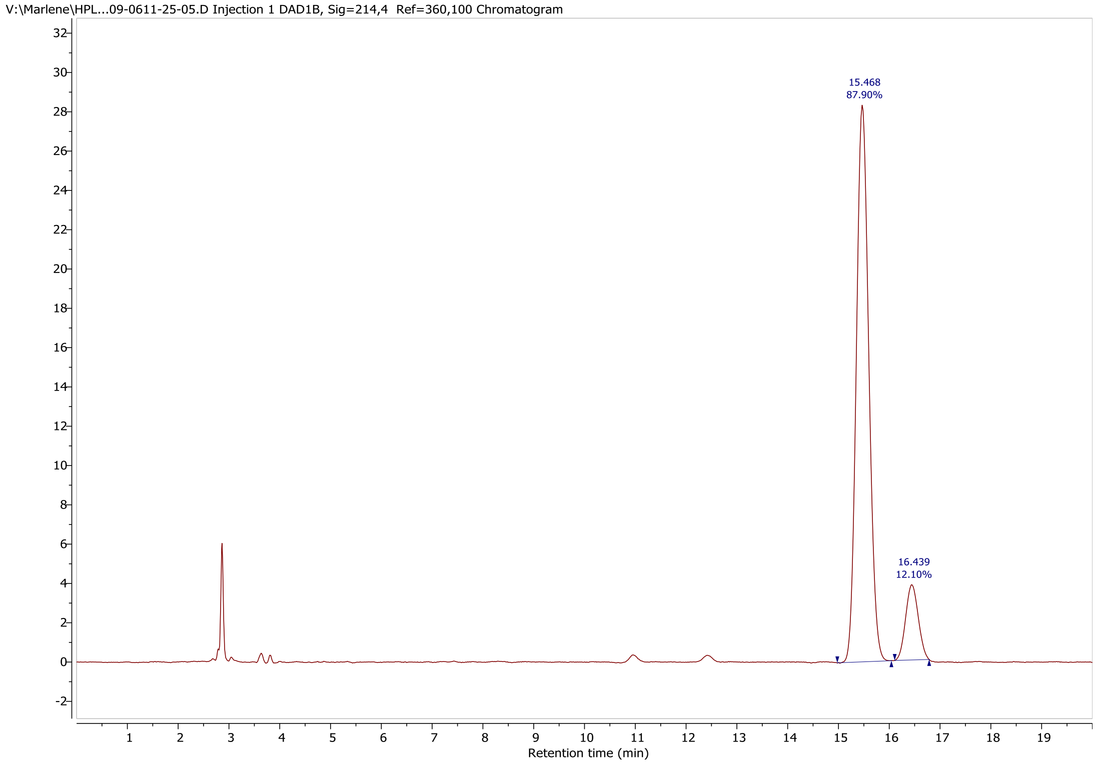

[*cis*-11d]

top: racemic sample

bottom: enantioenriched sample

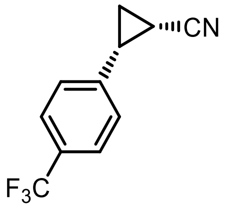

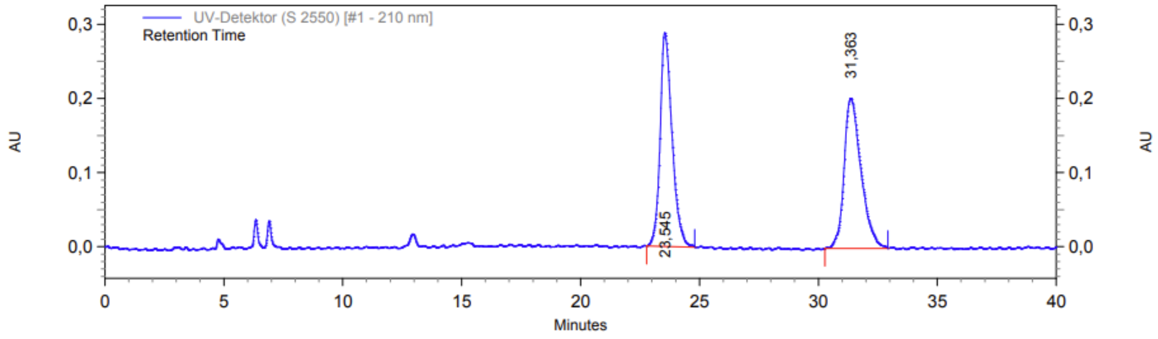

UV-Detektor (S 2550) [#1 - 210 nm] Results

Retention Time	Area	Area %
23,545	10100908	50,19
31,363	10023513	49,81

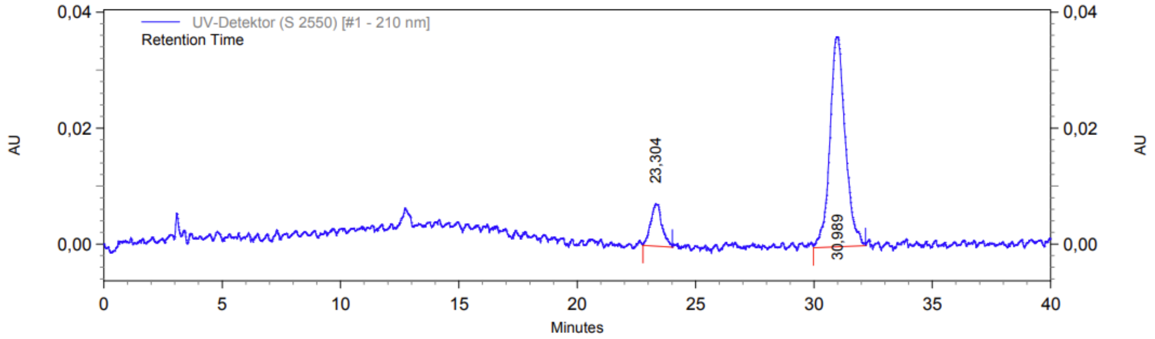

UV-Detektor (S 2550) [#1 - 210 nm] Results

Retention Time	Area	Area %
23,304	241609	12,89
30,989	1633116	87,11

[*cis*-11f]

top: racemic sample

bottom: enantioenriched sample

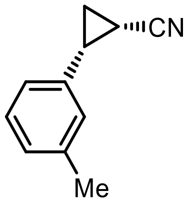

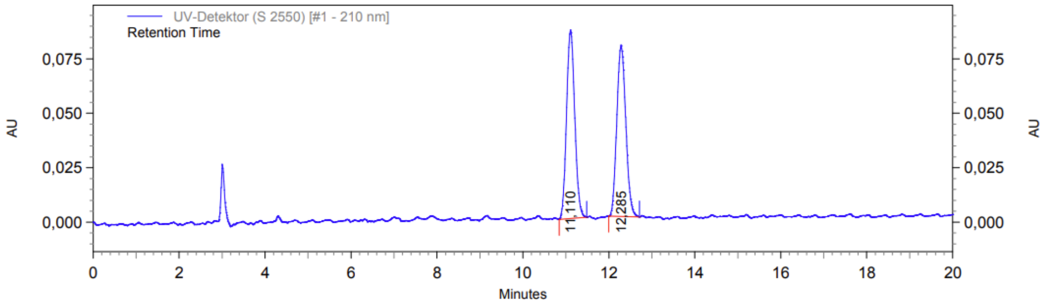

UV-Detektor (S 2550) [#1 - 210 nm] Results

Retention Time	Area	Area %
11,110	1152930	50,20
12,285	1143939	49,80

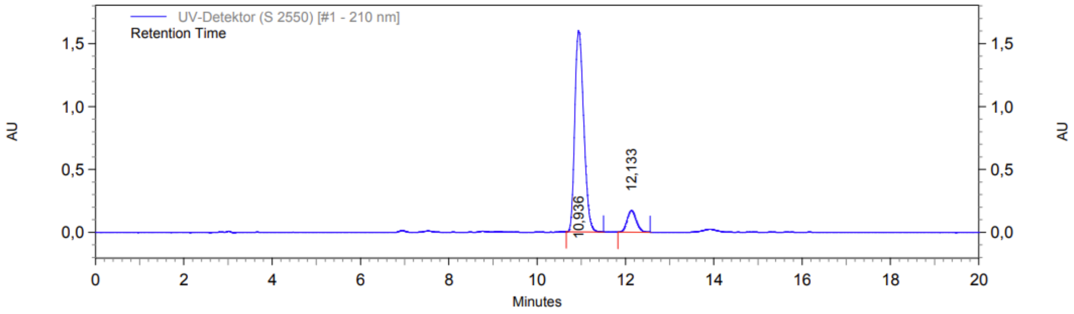

UV-Detektor (S 2550) [#1 - 210 nm] Results

Retention Time	Area	Area %
10,936	22497692	90,11
12,133	2467996	9,89

[*trans*-11f]

top: racemic sample

bottom: enantioenriched sample

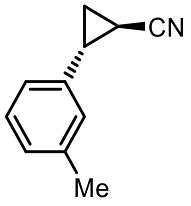

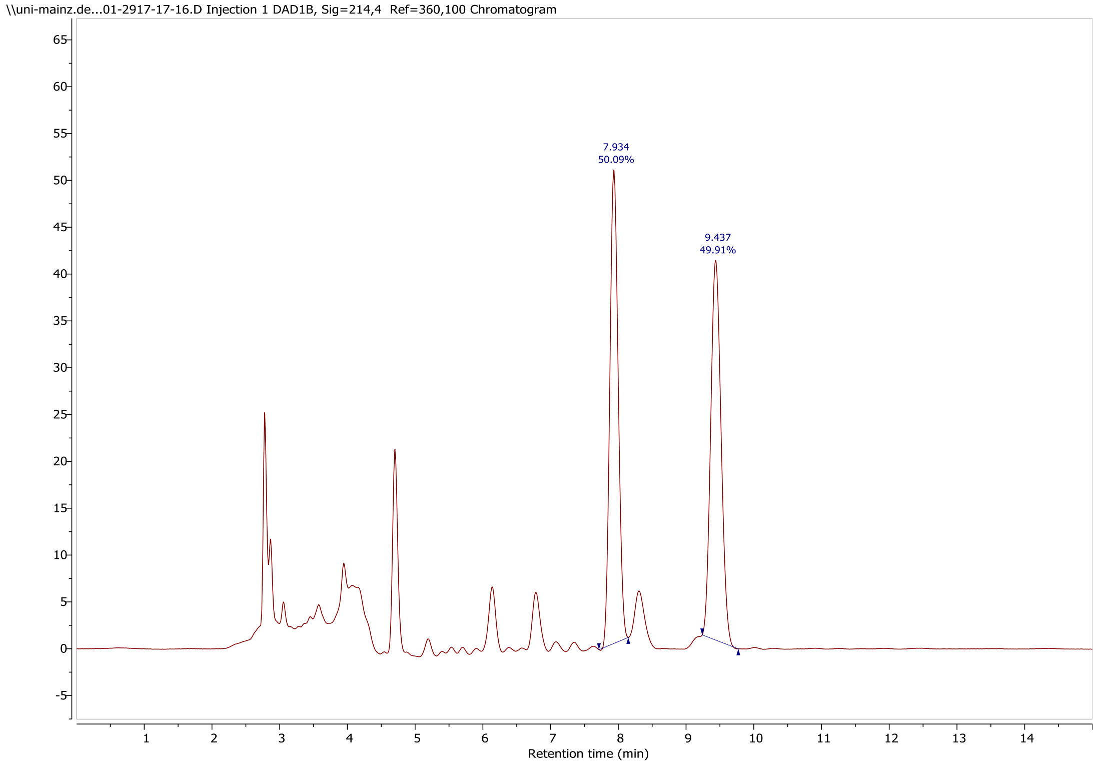

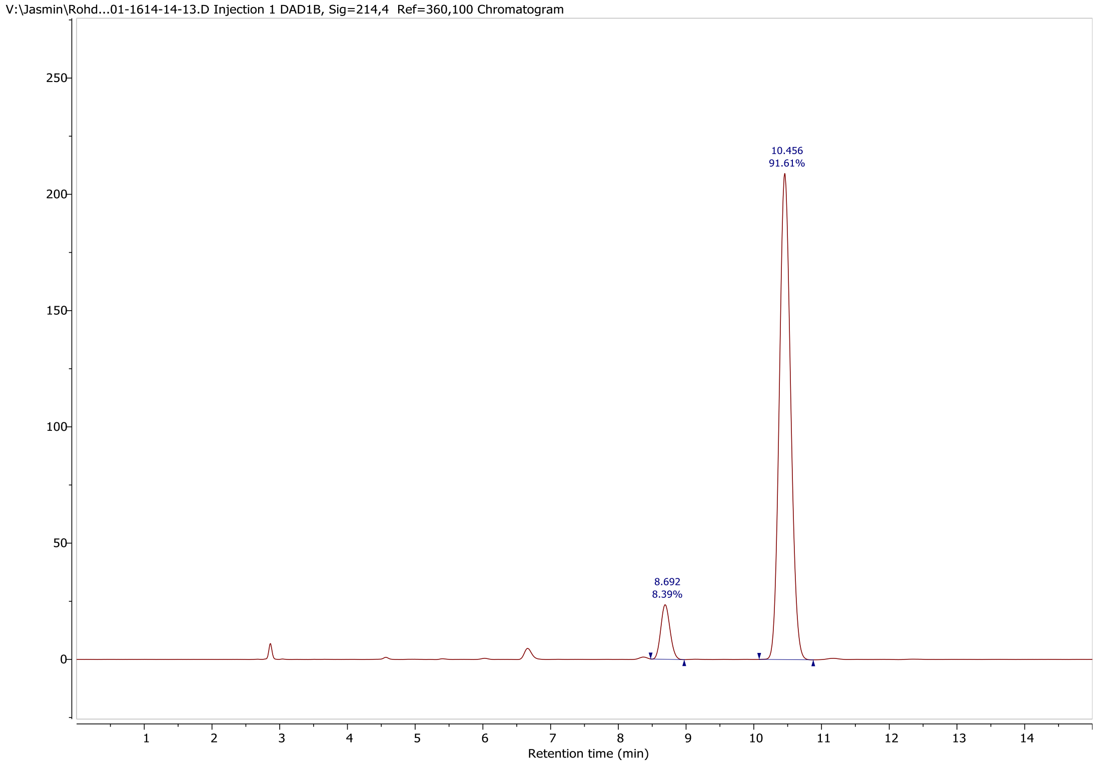

[*cis*-11g]

top: racemic sample

bottom: enantioenriched sample

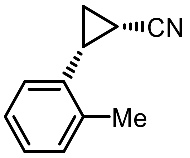

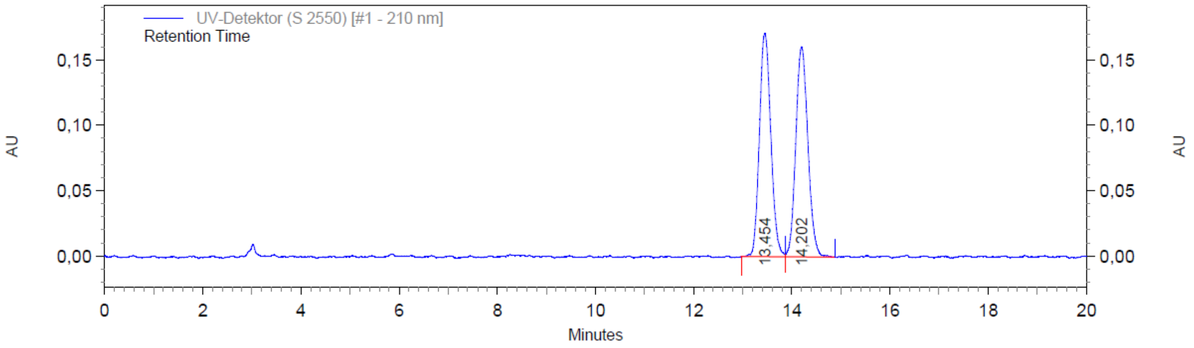

UV-Detektor (S 2550) [#1 - 210 nm] Results

Retention Time	Area	Area %
13,454	2753872	50,01
14,202	2752623	49,99

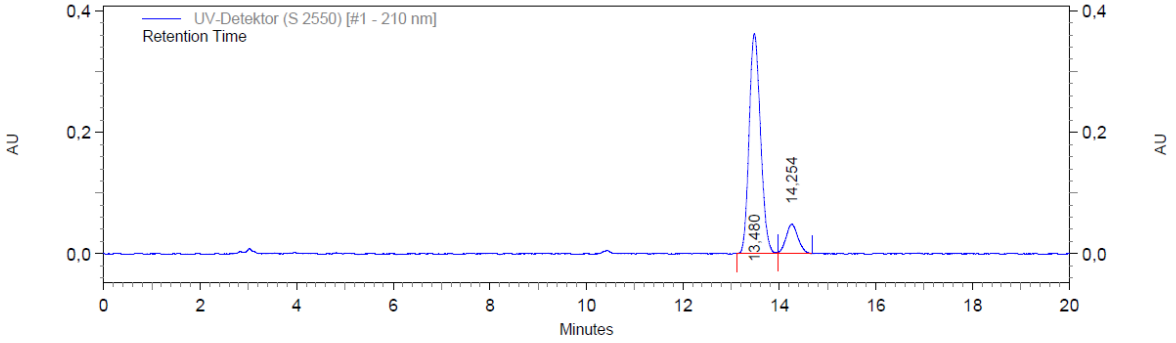

UV-Detektor (S 2550) [#1 - 210 nm] Results

Retention Time	Area	Area %
13,480	5927355	87,66
14,254	834649	12,34

[***trans*-11g**]

top: racemic sample

bottom: enantioenriched sample

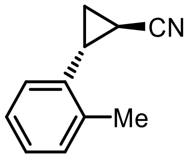

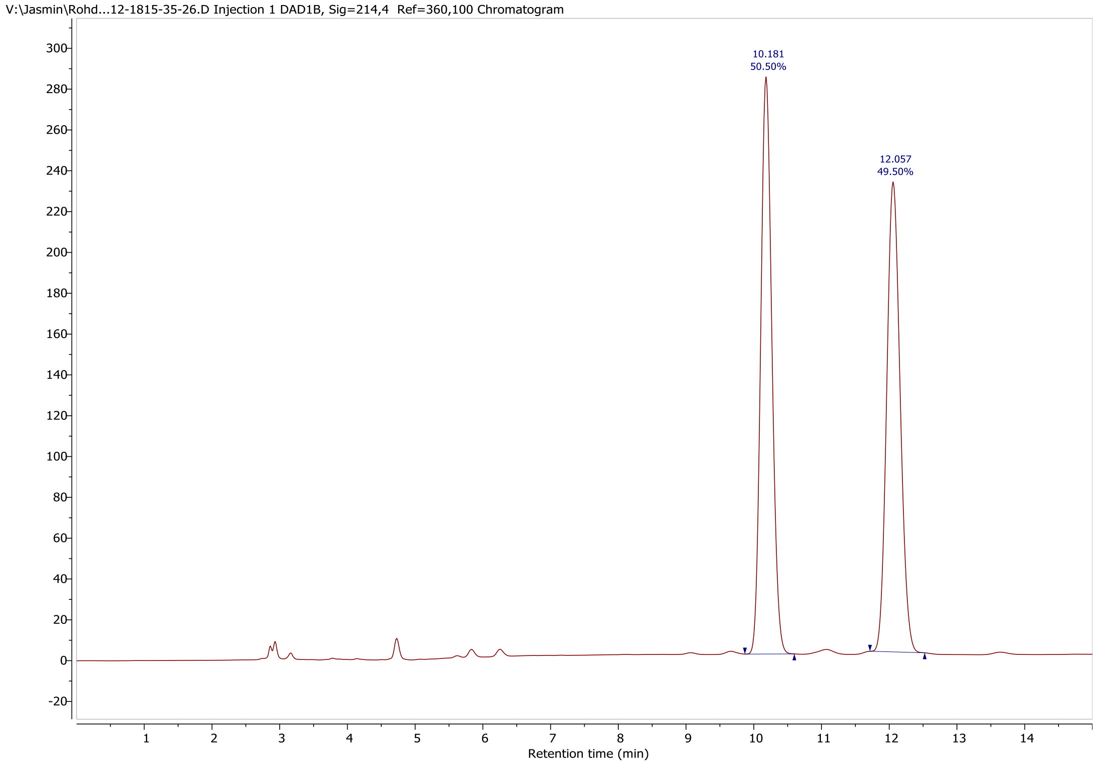

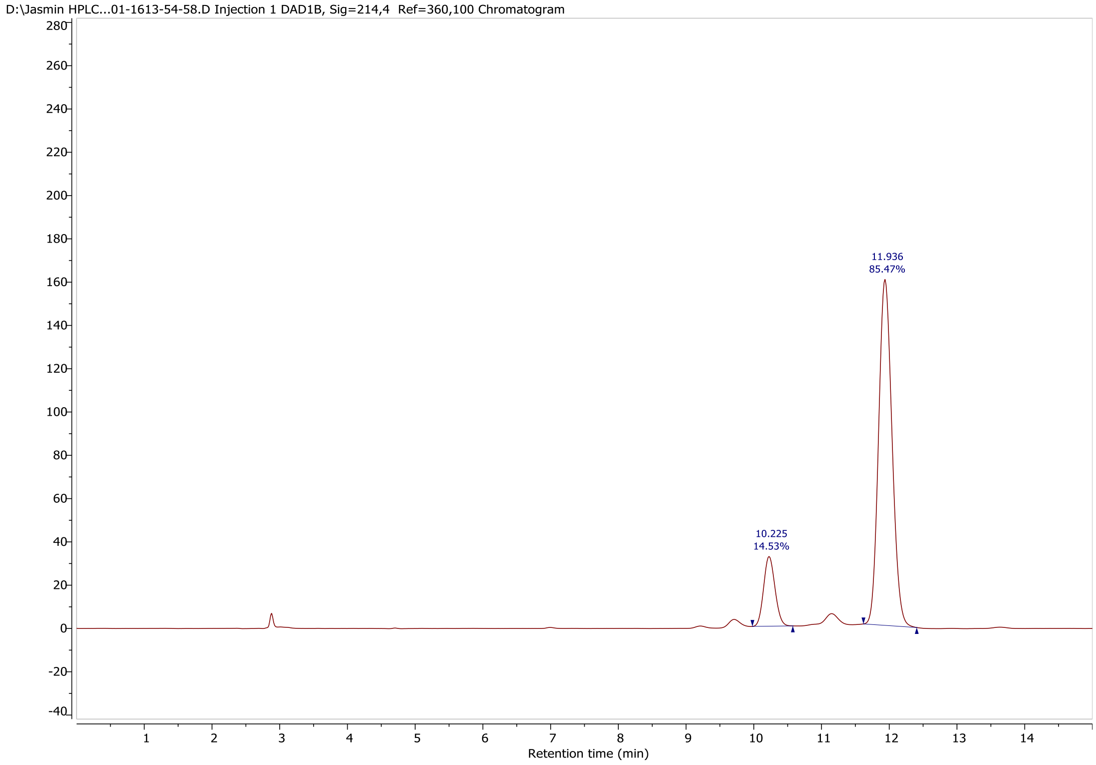

[*cis*-11i]

top: racemic sample

bottom: enantioenriched sample

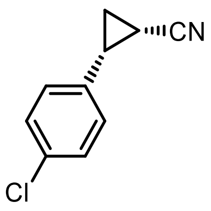

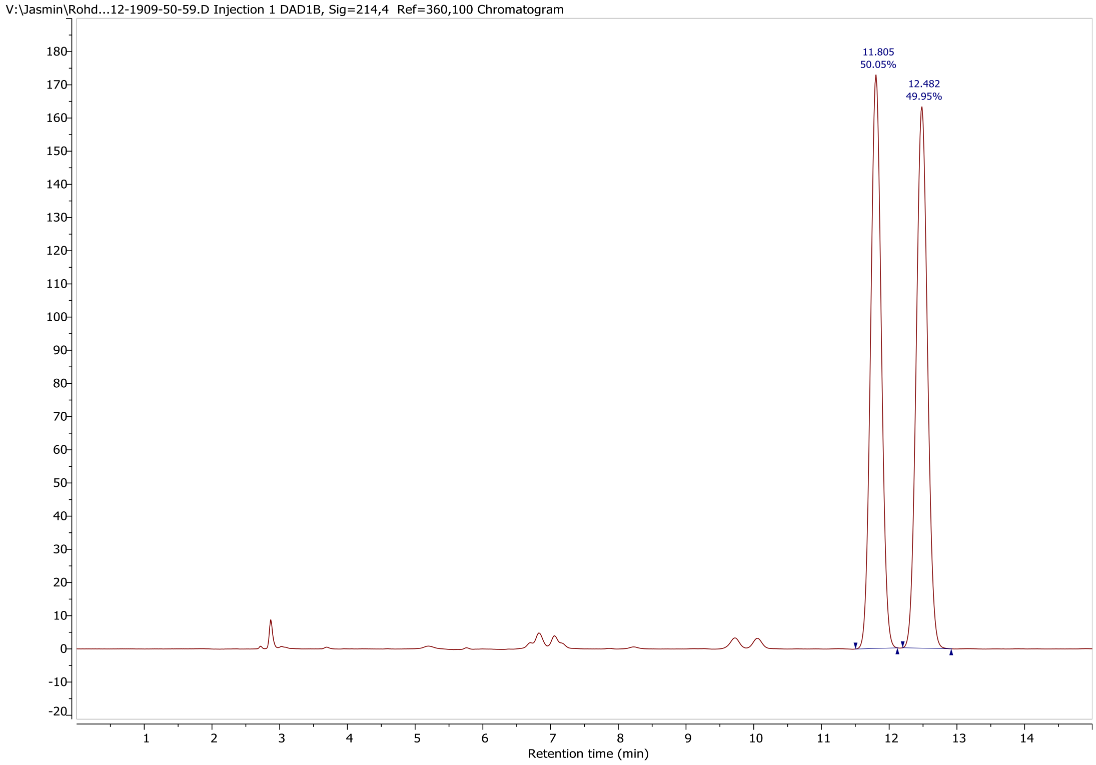

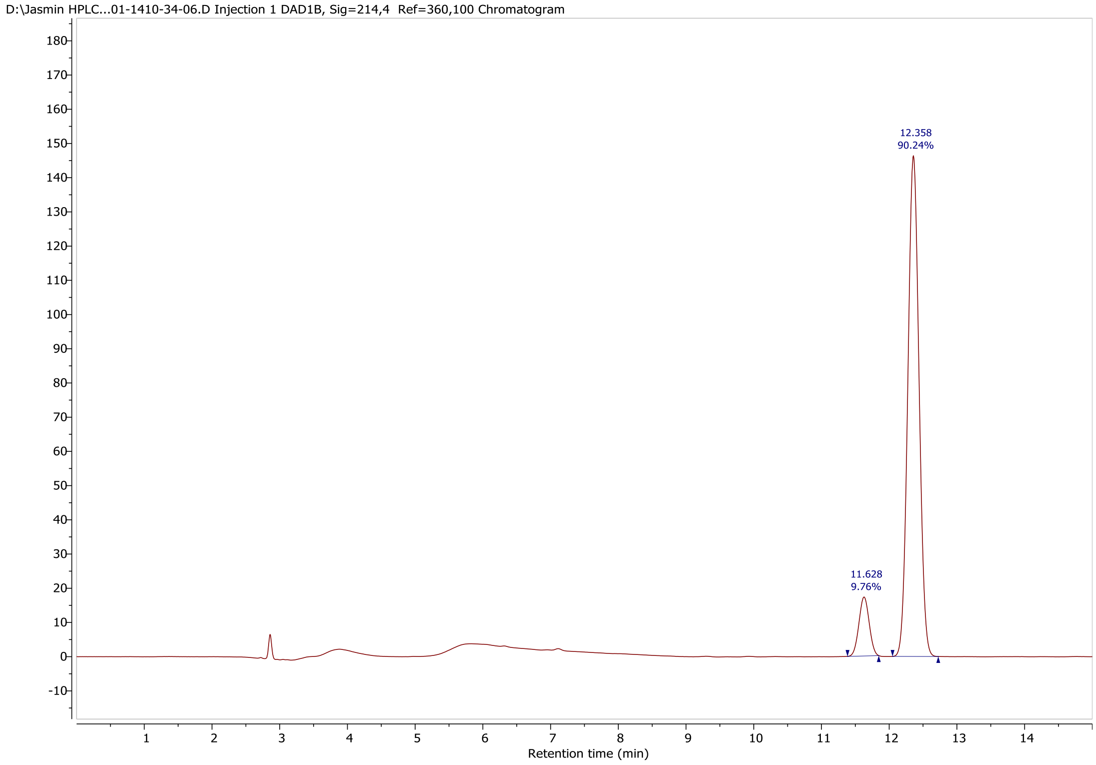

[*cis*-11k]

top: racemic sample

bottom: enantioenriched sample

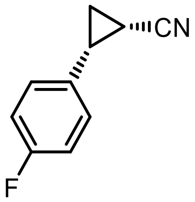

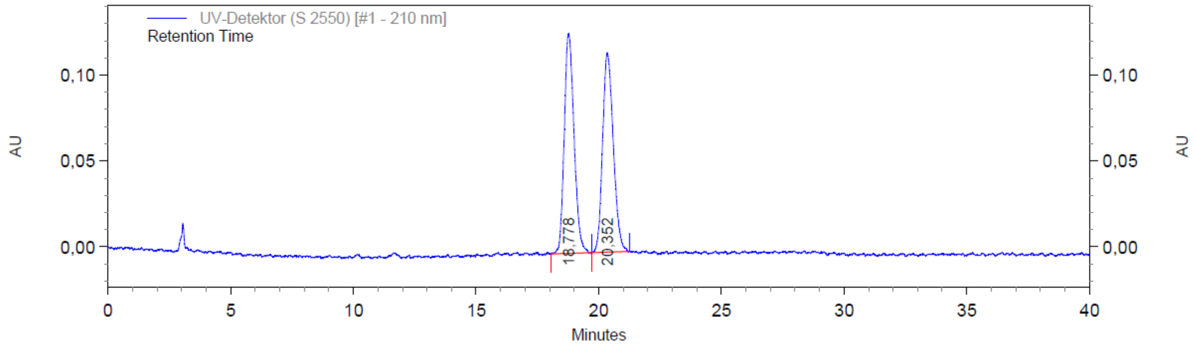

UV-Detektor (S 2550) [#1 - 210 nm] Results

Retention Time	Area	Area %
18,778	3553159	50,28
20,352	3513609	49,72

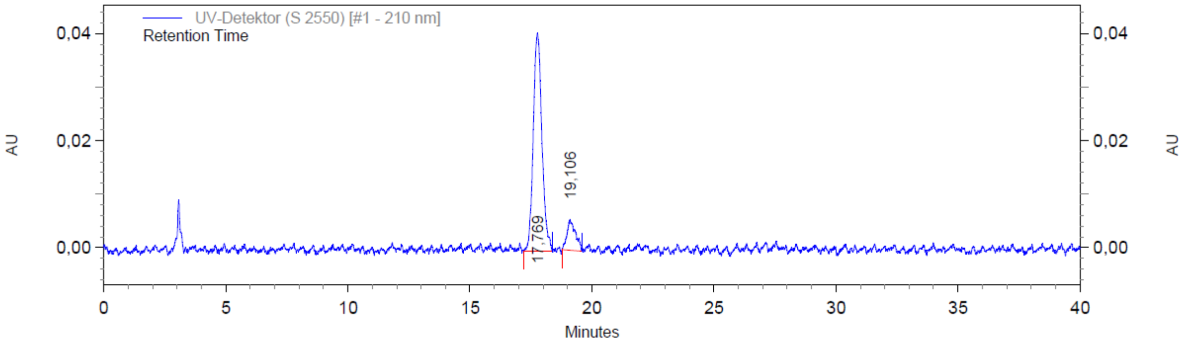

UV-Detektor (S 2550) [#1 - 210 nm] Results

Retention Time	Area	Area %
17,769	998878	87,71
19,106	140021	12,29

[*cis*-11l]

top: racemic sample

bottom: enantioenriched sample

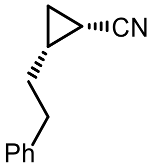

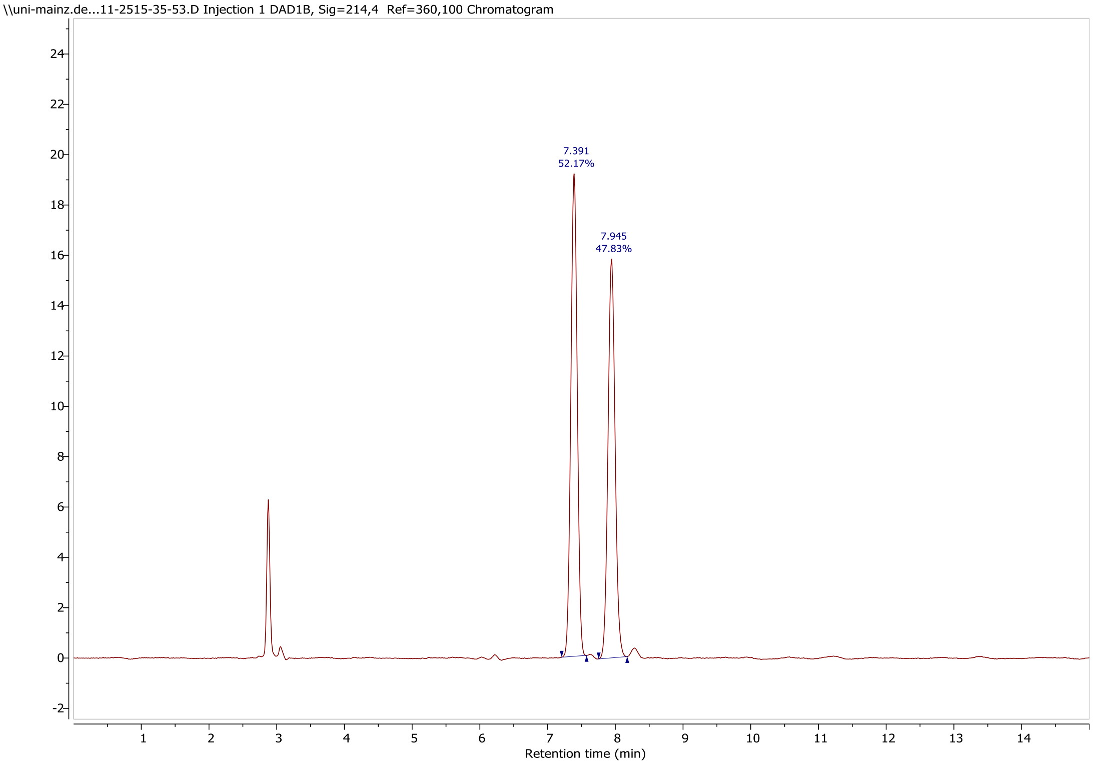

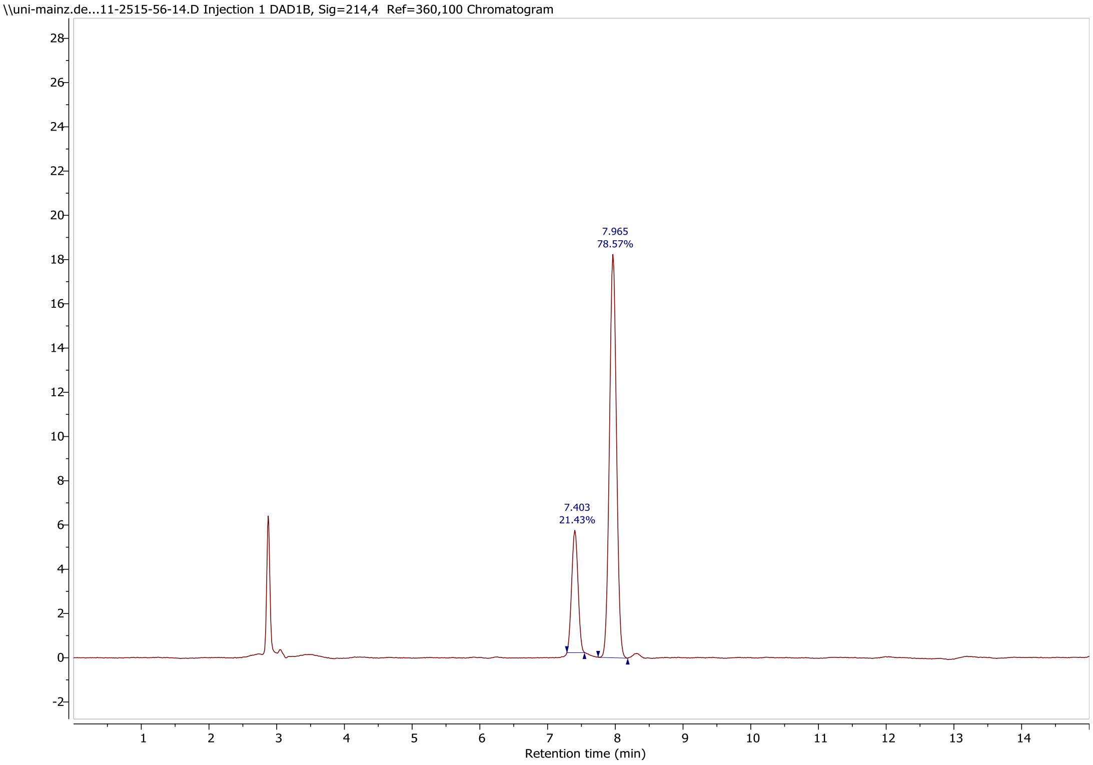

[16]

top: racemic sample

bottom: enantioenriched sample

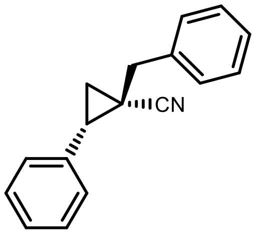

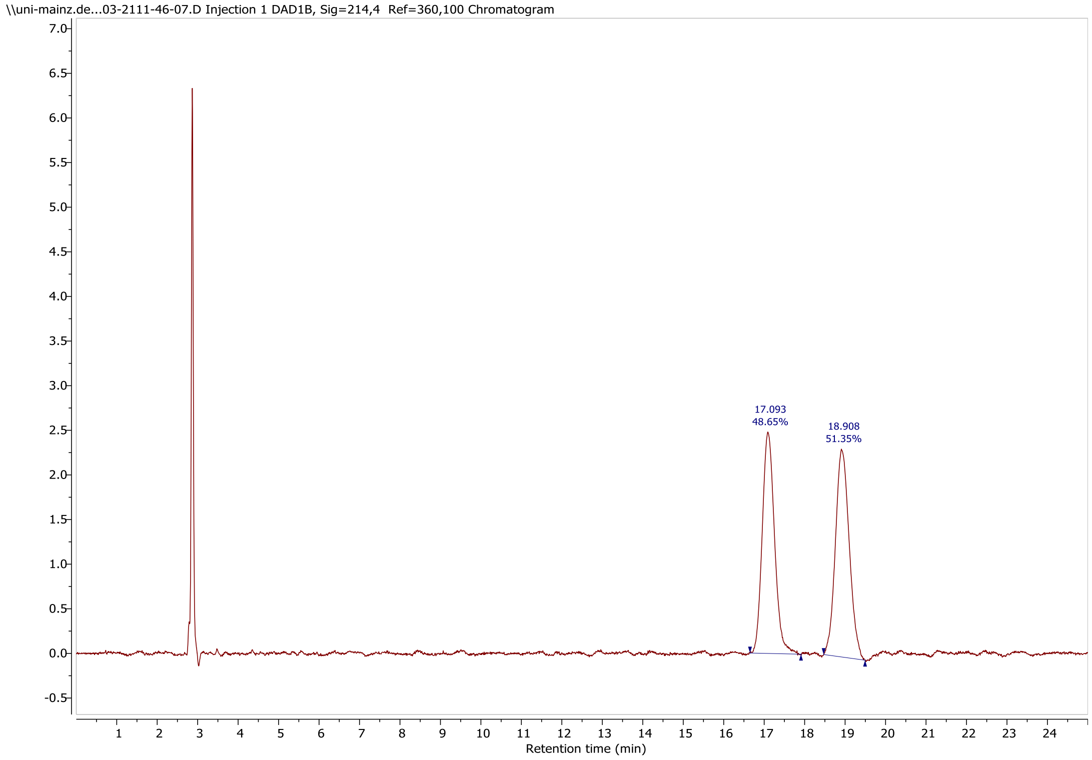

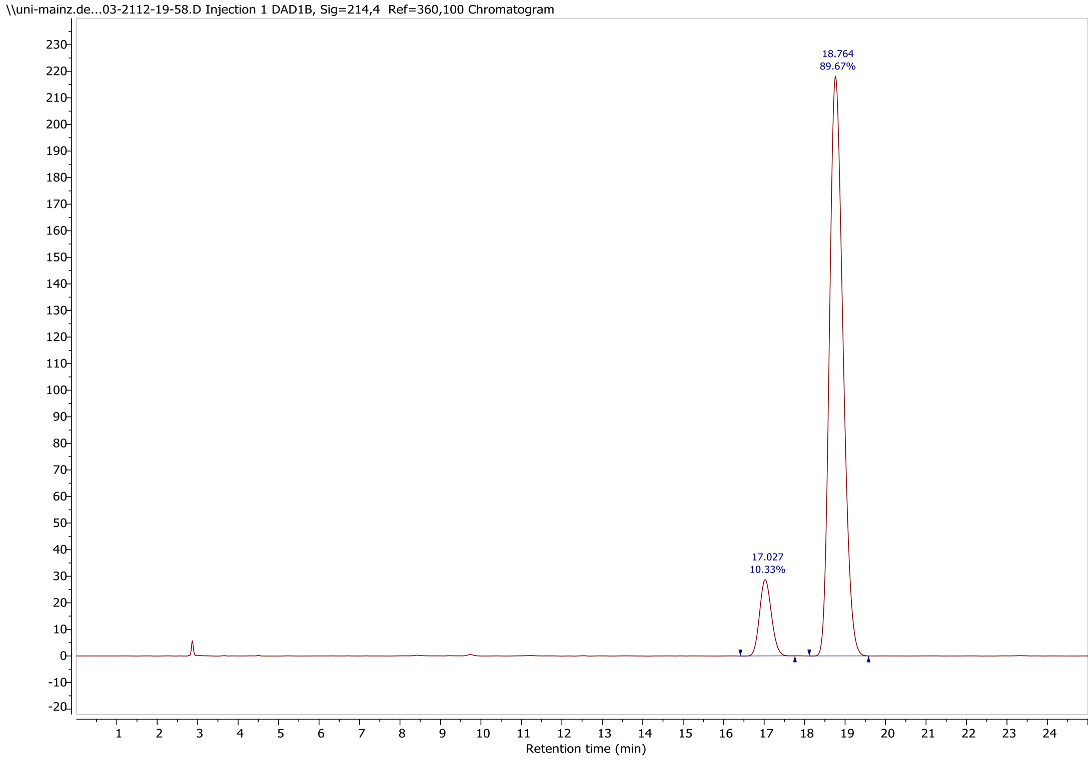

[*trans*-11p]

top: racemic sample

bottom: enantioenriched sample

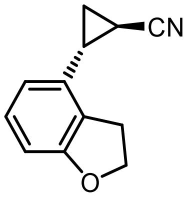

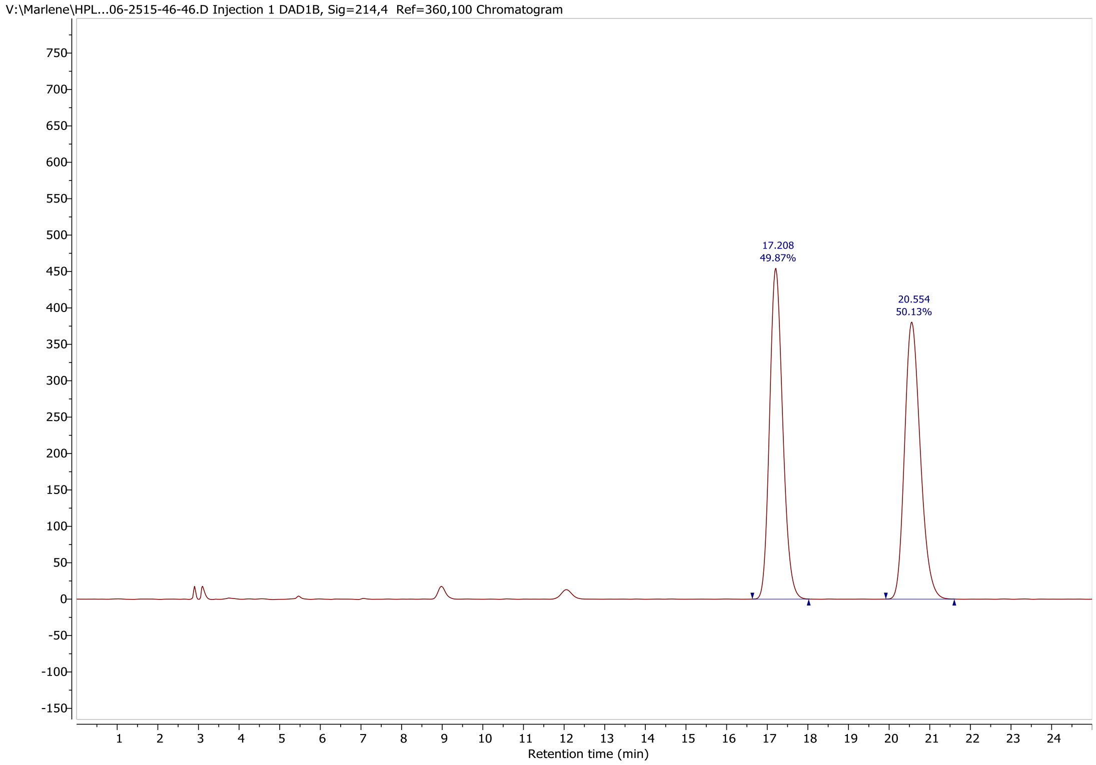

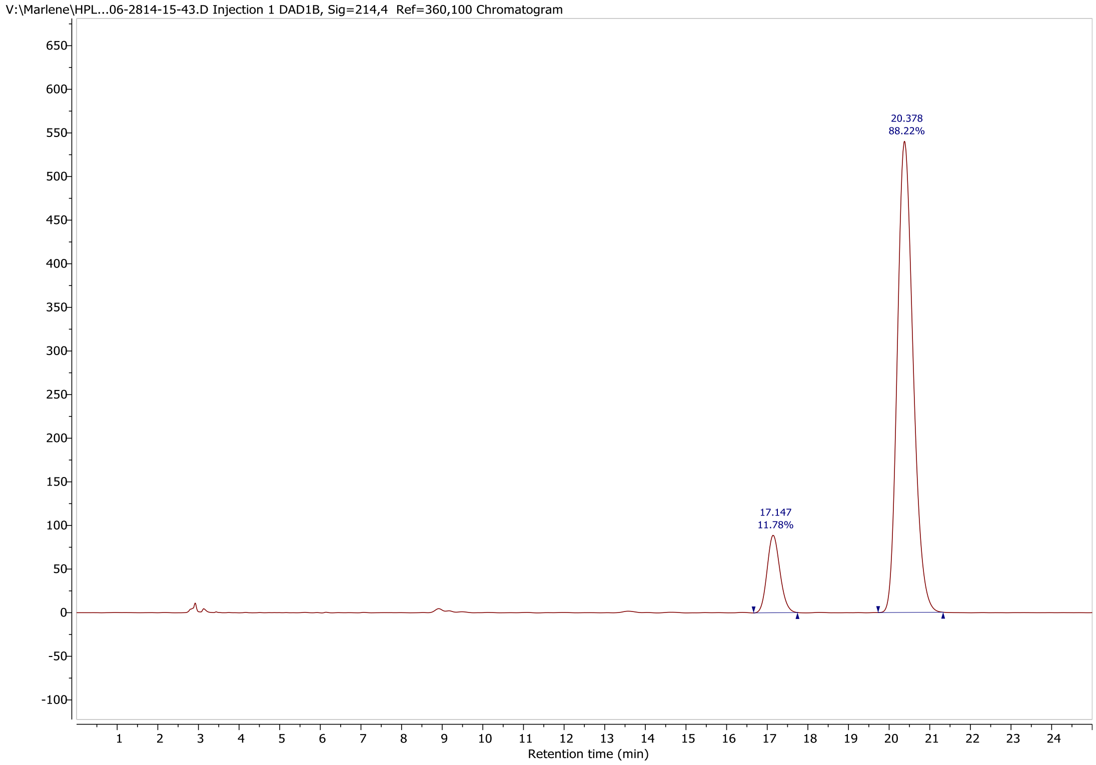

Supplement: Supplementary file 1 — Supporting Information [file ANIE-64-e202503056-s004.pdf]
